# Logit models, the area under receiver characteristic curves, sensitivity, and specificity for Co-enrollment density in college networks dataset.

**DOI:** 10.1016/j.dib.2021.107509

**Published:** 2021-10-26

**Authors:** Eric Leonardo Huerta-Manzanilla, Matthew W. Ohland, Manuel Toledano-Ayala, Juan Carlos Jáuregui-Correa

**Affiliations:** a Universidad Autónoma de Querétaro, México; b Purdue University, United States

**Keywords:** Student retention in college, Logistic regression in education, Receiver operating characteristics roc curves, Social network relations strength, Sensitivity and specificity

## Abstract

This article describes the data related to co-enrollment density (CD), a new network clustering index, that can predict persistence and graduation. The data hold the raw results and charts obtained with the algorithm for CD introduced in ``Co-Enrollment Density Predicts Engineering Students' Persistence and Graduation: College Networks and Logistic Regression Analysis.'' There are data for eight institutions that show CD as a predictor for graduation at four years, graduation at six years, and ever graduated. The files were processed using *R* to estimate CD at one, two, three, and four years. Logistic regression models, receiver operating characteristic curves, specificity, sensitivity, and cut-off points were estimated for each model. The *R* code to reproduce the metanalysis for the summary data is included. The displays for the logistic regression models, receiver operating characteristic curves, density curves for classes, models, and parameters are included.

**Specifications Table**

**Table:** 

Subject	Education
Specific subject area	Engineering education
Type of data	Table Chart
How data were acquired	The original enrollment records were obtained from The Multiple-Institution Database for Investigating Engineering Longitudinal Development (MIDFIELD) [1]. The records were filtered for students who declared engineering as a major and institutions that reported course sections. The records obtained were for 68293 students. These records were analyzed with the methodology proposed in “Co-Enrollment Density Predicts Engineering Students' First-Year Persistence and Graduation at Four and Six Years: College Networks and Logistic Regression Analysis.”
Data format	Raw data (XLSX) Code (R)
Parameters for data collection	Eight institutions and 68293 students were selected for the analysis. The records were processed, and the results of the research are presented in the included data. The data were collected from records for more than one million students, and filtered for those who declared engineering as a major.
Description of data collection	The MIDFIELD database holds more than one million students at the time of the study; after filtering the records, CD was calculated yearly from one to four years. Binary variables for persistence and graduation were fitted to CD with logistic regression. Logistic regression model parameters were consolidated for graduation at four, six, and ever in the table presented in this paper, along with the charts and raw results for the logistic regression analysis, receiver operating characteristic curve, and area under the curve.
Data source location	Country: USA Primary source: MIDFIELD
Data accessibility	With the article.
Related research article	Huerta-Manzanilla, E. L., Ohland, M. W., & Peniche-Vera, R. (2020). Co-Enrollment Density Predicts Engineering Students' Persistence and Graduation: College Networks and Logistic Regression Analysis. https://doi.org/10.1016/j.stueduc.2021.101025.

**Value of the Data**

• These data include co-enrollment density (CD), a novel index for college networks, and its cut-off points based on logistic regression models that predict graduation at four, six years, and ever.
• Researchers and educational practitioners involved in the study of the persistence of engineering students may take advantage of these examples.
• CD is a novel college network metric derived from standard enrollment records, known to predict graduation. The data provide examples of how network indexes derived from student records may be used to analyze educational outcomes like retention.
• The data and code could be re-used to develop comparisons between groups, which is demonstrated by comparing the area under the receiver curve, specificity, sensitivity, and cut-off points by the predictor. In addition, the code allows its replication with the data included and with other similar data. The charts obtained will consist of box plots along with Dunn's pairwise tests.
• The researchers interested in the related article may confirm the findings reported there. In addition, they will have access to the entire set of charts (Logistic regression, area under the receiver operating curve, and Youden's cut-off estimation) and models (regression coefficient tests and Youden's approximations) [10].
• The explanations on the methods used to derive co-enrollment density from academic records may help to replicate its estimation with academic records that include course and student-enrollment information.

## 1 Data Description

This data set holds the raw results for evaluating co-enrollment density (CD) in engineering college networks. CD is the logarithm base 2 of co-enrollment frequency: the total count of students enrolling in the same courses during their studies.

Co-enrollment frequencies are the sum of rows or columns in a students’ adjacency matrix. An adjacency matrix is a squared matrix with a column and a row per enrolled student. The intersections value one when two students co-enrolled and zero when not, except the diagonal that holds the total count of courses enrolled per student. Therefore, the sum of any column or row, less the value in the diagonal equals the co-enrollment frequency. Co-enrollment density is the logarithm base 2 of the co-enrollment frequency (Eq. 1).

(1) $$CD=\log_{2}\left[\sum_{j\in J}\left(x_{ij}\right)-x_{i=J}\right]$$

The critical parameter in this data set is the CD's cut-off point at one to four years. This number is the value for CD that predicts higher odds of graduation. A student with greater CD than the cut-off is likely to graduate, and a student with a lower CD than the cut-off is at risk of not persist. The cut-off points are estimated based on the odds ratios (OR) for each college's model. The logistic regression allows to estimate coefficients in OR units that are the increment in the odds of graduation proportional to the increments in CD. The cut-off points are the CD values that better differentiate potential graduates from students at risk of not persist [10].

The table ``meta.xls'', included along with this paper, shows the parameters for all the models.

Table 1 shows the notations of the columns' headers' identifiers in the raw data table “meta.xls”. A brief description of the content of each column follows. A ``Code Book'' in the Excel file has a short note about each variable, too.

**Table 1:** Notations.

*Ins*	Institution identifier: B, C, D, E, F, H, I, J;
*X*	Predictor: CeD1, CeD2, CeD3, and CeD4;
*Y*	Response: P1, G4, G6, and EG;
*CUT*	Cut-off values for CeD based on Youden's index (j);
Spe=TN/(TN+FP)	Specificity, true negative *TN*=True negatives, *FP*=False positives;
Sen=TP/(TP+FN)	Sensitivity, true fraction *TP*=True positives, *FN*=False negatives;
*AUC*	The area under the receiver operating characteristic curve;
*AIC*	Akaike's entropy-based information criterion;
OR=p(Y)/(1−p(Y))	Odds ratio;
*CI*	Confidence interval at 95%, unless otherwise specified;
*P*	*P*-value *p* < 0.001 (0.001), and specific value for *p*>0.001;
*ROC*	Receiver operating characteristic curve;

The variable ***Ins*** shows an anonymous identifier for each of the eight institutions in the sample. The institutions involved in the study are medium to large American universities that offer four-year engineering degrees. The data in the table belongs to engineering students only.

The variable ***X*** holds the predictor's categories. The column shows the Co-enrollment density identifier for each college. The code's digit indicates how many enrollment years are included in the model, from one to four years (CeD1– CeD4). It is the predictor of the logistic models.

The variable ***Y*** is the response or dependent variable for the logistic models. This variable defines the time to graduation, including four (G4), six years (G6), and ever graduated (EG).

The variable ***CUT*** holds the cut-off values for CD. These were calculated using Youden's index (Fluss et al., 2005). The cut-off defines a parameter to identify students at risk of not graduate, which are the students with lower CD values than the CUT. Students with higher CD values than the cut-off are more likely to graduate.

The column with the header ***Spe*** shows the percentage of specificity or true negative fraction, and the column with header ***Sen*** shows the percentage of sensitivity, the true positive rate.

***AUC*** shows the area under the receiver operating curve for each of the models in the study. The curve is plotted in a chart with 1-Specificity in the x-axis and Sensitivity in the y-axis. AUC measures the logistic regression models' performance across all the classification thresholds for students potentially graduating and those less likely to graduate. The columns ***auc025*** and ***auc975*** are the 95% confidence intervals for the AUC.

***OR*** column holds the odds of graduation for a student given that he or she has CD equals or greater than the ***CUT. ORCI*** is the OR 95% confidence intervals for the odds ratios at the CUT for each model. The last column identified with ***P*** has the statistical significance for each model.

The files have a three-digit identification code that is described in Table 1.

For example, a file identified as 115-cell.TXT is for institution B and CeD1 as a predictor of P1. It contains the summary indexes for the logit model describing the fit of P1 on CeD1. There are nine sets per institution. Each set represents the logit model fit, the receiver operating characteristic curve (ROC), the AUC, the CUTj, the Sen, and Spe for each pair of predictor and response, as indicated in Table 2.

**Table 2:** Three digits code file identifier.

First digit: 1 to 8	The anonymous institutional identifier: B, C, D, E, F, H, I, and J;
Second digit: 1 to 4	The predictor: 1 – CeD1, 2 – CeD2, 3 – CeD3, and 4 – CeD4;
Third digit: 5 to 8	The response: 5 – P1, 6 – G4, 7 – G6, and 8 – EG;

There are seventy-two logit models in total. Each model has a set of six files describing its elements. Table 3 describes the files’ content.

**Table 3:** The responses and predictors for the nine models per institution [9].

Predictor ψ	Response Y	Logit models	Significant
CeD1	P1, G4, G6	24	20
CeD2	G4, G6	16	16
CeD3	G6, EG	16	16
CeD4	G6, EG	16	16

## 2 Experimental Design, Materials and Methods

**Fig. 1:**
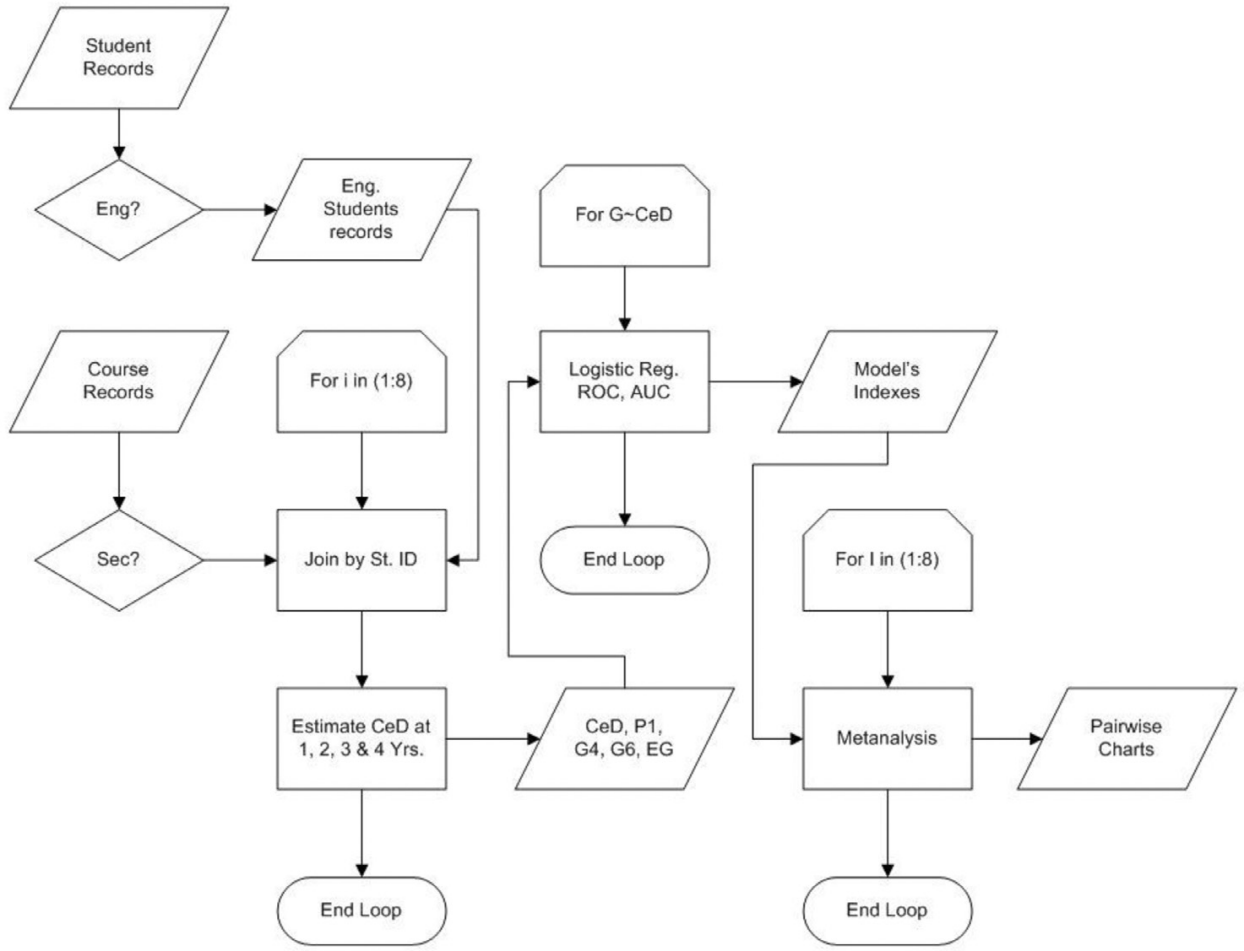
Methods implemented to process the original data. *Note*. The flow chart shows how the original records for students and courses were filtered and joined to get full tables per institution used to estimate CeD and then build the logistic regression models.

Fig. 1 shows the study's design, the datasets taken as inputs, the intermediate process, and the metanalysis. The procedures explained here may help the parties interested in replicating the estimation of co-enrollment density with academic records that include course-student enrollment data.

1. Estimating CeD (First and second column in Fig. 1)

The original data in MIDFIELD were held in two large files in *.sas7bdat format. The students' records were in a 3.3 GB file with 425 columns and 1037778 observations. This file includes variables with some demographic fields and whole college academic records. The course record was a 2.6 GB file with twelve fields and over 33 million observations representing the enrollment records per term, per student. The students' records were filtered for students that declared engineering as a major, and the course records were screened for the institutions that report section data.

The filtered datasets for students and courses were joined by student ID per institution. Each subset was split into subsets per year. These subsets were the base to estimate CeD at the first year, the second year, etc. The results were tables with CeD per year, per student, paired with binomial data for P1, G4, G6, and EG.

1.1. Input: Students and course records in MIDFIELD.
1.2. Output: Table with CeD at 1 to 4 years paired with binomial data for P1, G4, G6, and EG.

2. Computing the logistic regression models and their tests (Third column in Fig. 1)

The logistic regression models and their corresponding tests were calculated from the tables produced in the previous steps. Logistic regression models' coefficients, receiver operating characteristic curves, area under the receiver operating curves, cut-off points for CeD, and a table with coefficients per model were computed. The files obtained as an output of this step are included in the article.

2.1. Input: Table with CeD at 1 to 4 years paired with binomial data for P1, G4, G6, and EG.
2.2. Output: The files are shown in Table 4 and included with this article.

**Table 4:** Files codes and content description.

xxx-Cell.TXT	Model's summary indexes;
xxx-ClassDen.PNG	Density for response categories: 0 – Non-persister, 1 – Persister;
xxx-LogitCurve.PNG	Logit model curve display;
xxx-ROCut.PNG	ROC curves with CUTj points;
xxx-SenSpe.PNG	Sen + Spe charts;
xxx-Summ.TXT	Model's detailed summary;

3. Computing the metanalysis (Fourth column in Fig. 1)

In the previous step, a table with all the logistic regression models’ indexes is produced. The table includes the fields for the variables listed in Table 1, and this table is included with this article. The code may be re-used to make between-group analysis with data that include group and response fields. The variable used for the grouping was the predictor (X) in the data contained in this article. The R code to process the results to get the metanalysis is presented below:

The file called meta.xlsx contains the raw data summary to prepare the group comparison charts for the metanalysis. Table 4 shows the variables in the file. A script in R [2] will process the XLSX file and produce group comparison charts to analyze the dataset results. The steps of the code are the following:

3.1. Setting the libraries, and the folders

For the metanalysis, the libraries needed are: {pacman} [3], {ggstatplot} [4], {ggplot2} [5], {ggthemes} [6], and {readxl} [7].

#———————————————————————

# Libraries

# The reader is encourage to use the {pacman} library for p_load()

# libraries instead of the more common library(), or require().

if (!require("pacman")) {

install.packages("pacman")

library(pacman)

} #Install {pacman} if it is not yet in the computer.

p_load(ggstatsplot, #Produce the charts: ggbetweenstats();

folderfun, #Set a folder function: setff();

ggplot2, #Allows to save the charts: ggsave();

ggthemes, #Select predefined themes: theme_hc();

readxl) #Read MS Excel files: read_excel();

#———————————————————————

3.2. Reading data from disk

This code defines a function to access the folder. This example is called "meta," where the input file will be read from, and the charts will be saved. The folder should exist as a subfolder within the folder where the R environment containing the code is located.

#———————————————————————

# folders

setff("io", "meta") #Set a subfolder meta to hold the files

# Data

meta <- read_excel(ffio("meta.xlsx"))

factores <- c("Ins", "Y", "X")

meta[,factores] <- lapply(meta[,factores], factor)

met <- meta[(meta$CUT > 2),] #Screen out non significant models

#———————————————————————

3.3. The function to save the chart to disk

This function saves a jpeg chart to disk. It takes the name of the chart in R in "x" and the file's name in "y." This list should include the file extension and be in character format.

#————————————————

# Function to save the charts to disk

sgr <- function(x,y){ggsave(filename = ffio(x),

width = 10,

height= 6,

plot = y,

device = "jpeg",

dpi = 320,

units = 'in')}

#————————————————

3.4. The code to produce and save the charts

This code generates the chart for the predictor and response, adds the title, and names the axis.

#—————————————

# plot OR by X

or <- ggbetweenstats(

data = met,

x = X,

y = OR,

mean.ci = T,

type = "np",

bf.message = T,

results.subtitle = F,

outlier.tagging = T,

outlier.label = Ins,

ggtheme = theme_hc(base_size = 14),

xlab = "Predictor",

ylab = 'OR',

caption = FALSE,

pairwise.comparisons = T,

plot.type = "box",

messages = FALSE

); sgr("8-ORbyX.jpg",or)

#—————————————

The code and the tables may produce similar charts to the ones included in the related research article. It is also an example for the comparative analysis of other continuous variables by categorical group variables. However, we believe that the data's main contribution is the cut-off values for co-enrollment density in the main data table.

## Ethics Statement

The data has no personal information neither institutional references that may compromise the privacy of any parties. It is entirely anonymous; therefore, no ethical implication should be declared.

## CRediT authorship contribution statement

**Eric Leonardo Huerta-Manzanilla:** Methodology, Software, Investigation, Formal analysis, Writing – original draft. **Matthew W. Ohland:** Conceptualization, Data curation, Writing – review & editing. **Manuel Toledano-Ayala:** Funding acquisition. **Juan Carlos Jáuregui-Correa:** Funding acquisition.

## Declaration of Competing Interest

The authors declare that they have no known competing for financial interests or personal relationships which have, or could be perceived to have, influenced the work reported in this article.

## Appendix Supplementary materials

Image, application 1
