# Supplementary figures and images for: Logit models, the area under receiver characteristic curves, sensitivity, and specificity for Co-enrollment density in college networks dataset (part 2 of 2)
Source: Data Brief. 2021 Oct 26;39:107509. doi: 10.1016/j.dib.2021.107509 (PMC8573127; doi:10.1016/j.dib.2021.107509)

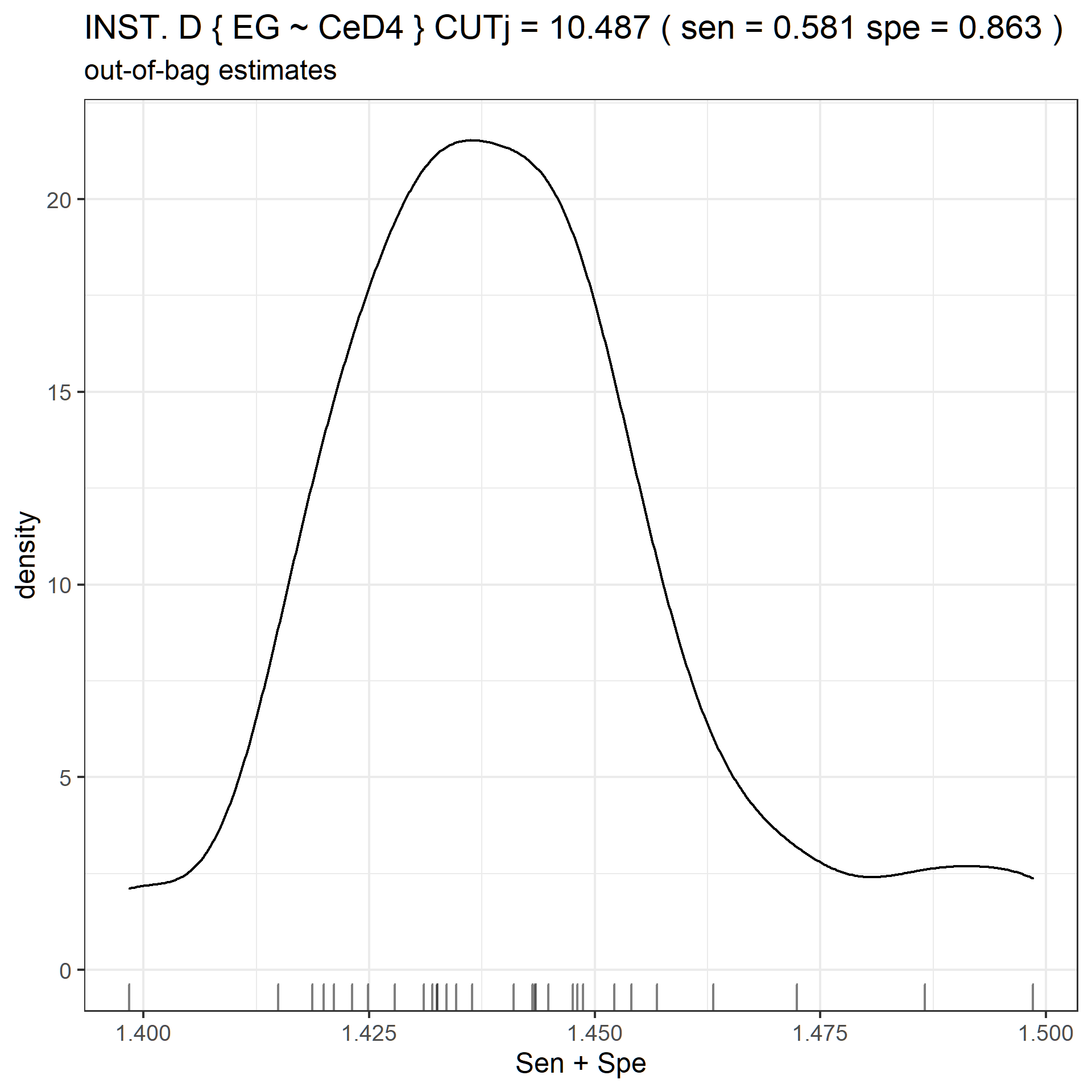

Supplement: Supplementary file 1 [file mmc1.zip › SupplementaryMaterials/348-SenSpe.png]

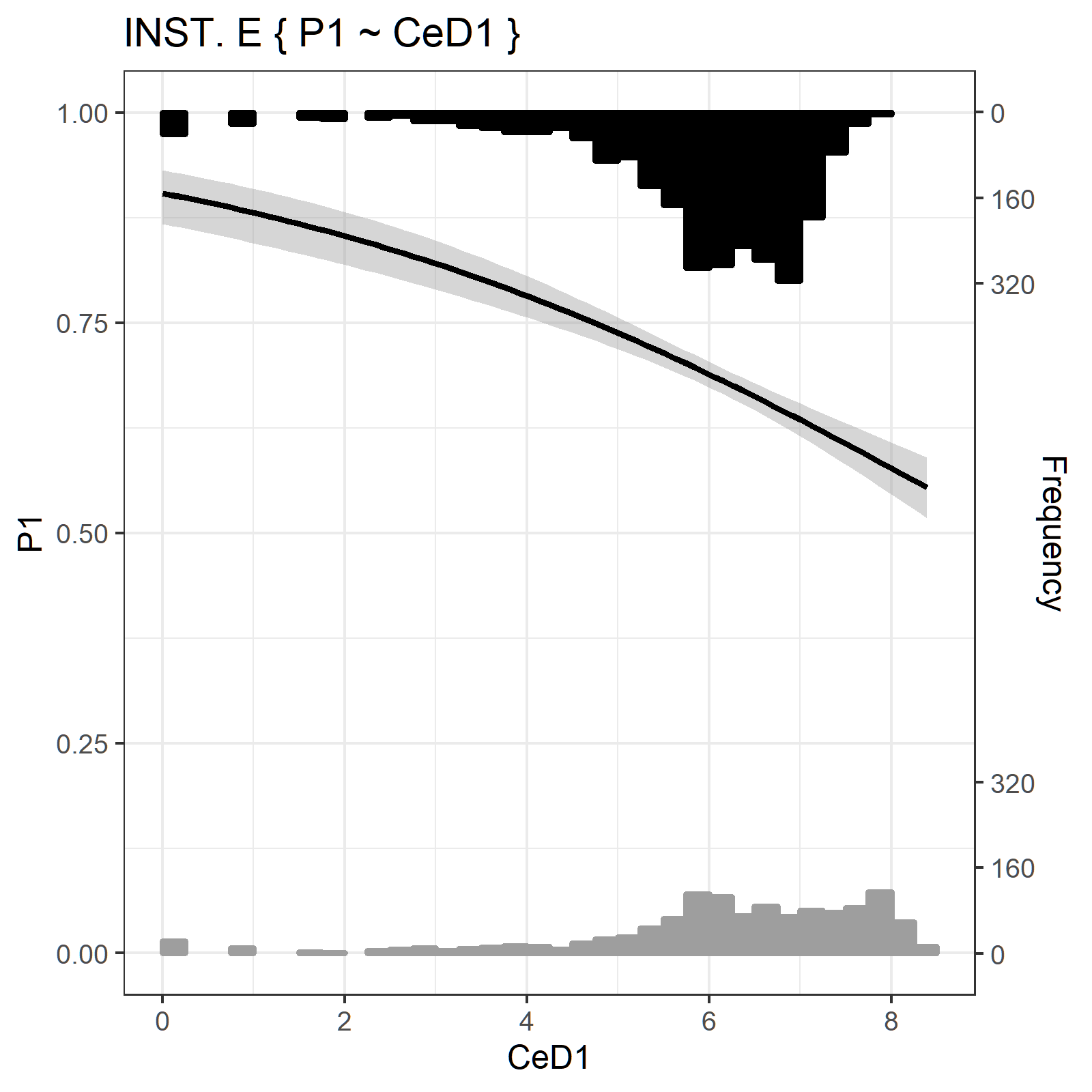

Supplement: Supplementary file 1 [file mmc1.zip › SupplementaryMaterials/415-LogitCurve.png]

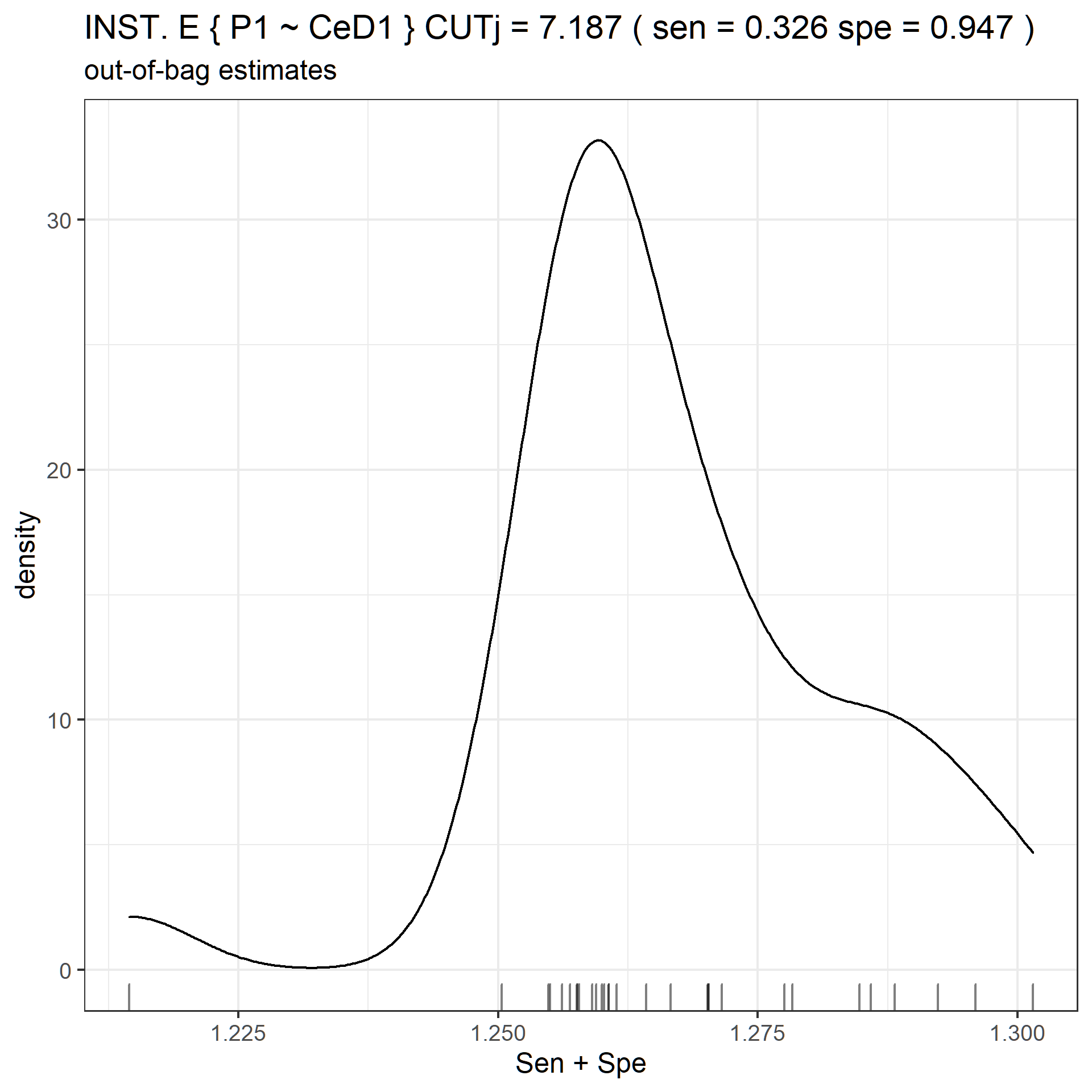

Supplement: Supplementary file 1 [file mmc1.zip › SupplementaryMaterials/415-SenSpe.png]

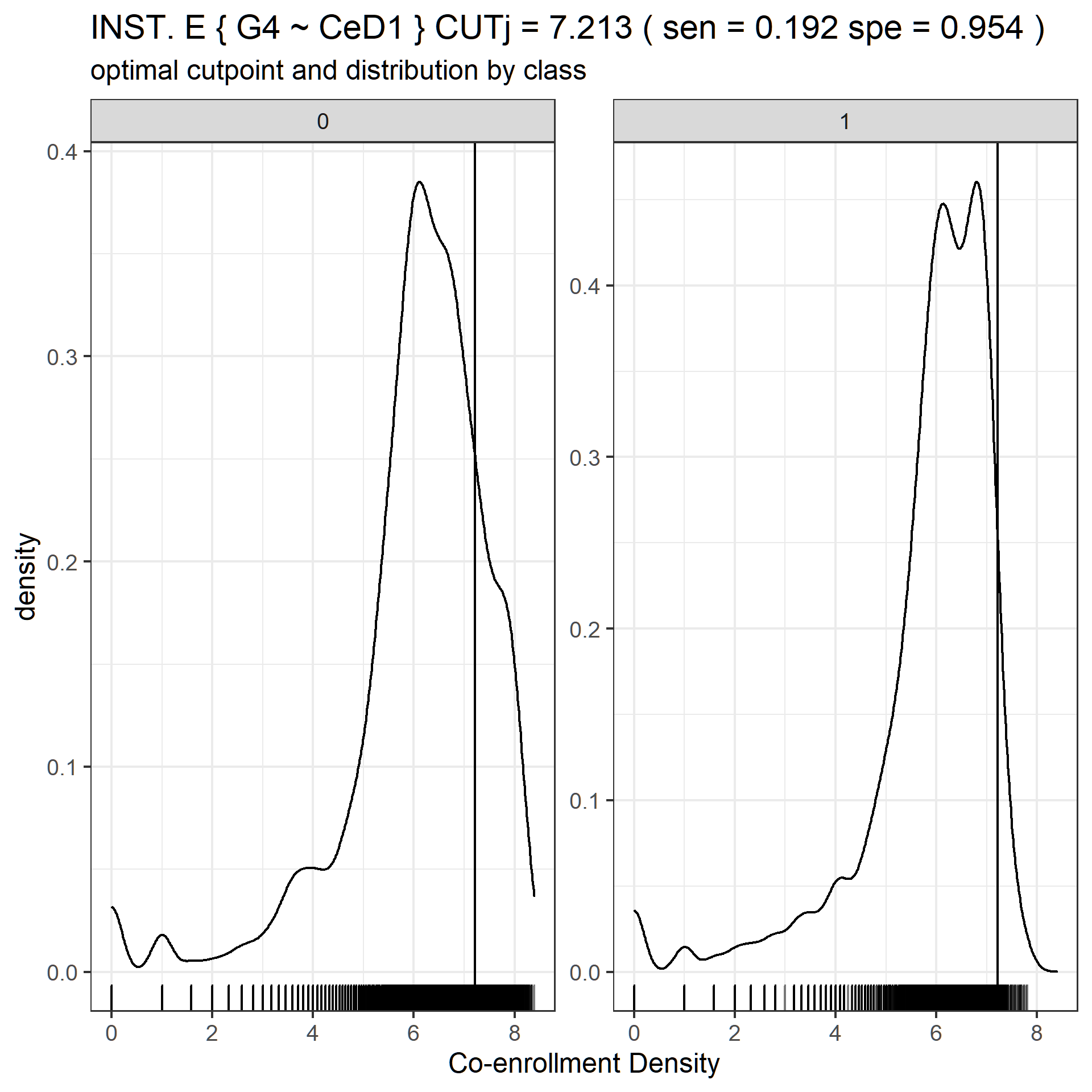

Supplement: Supplementary file 1 [file mmc1.zip › SupplementaryMaterials/416-ClassDen.png]

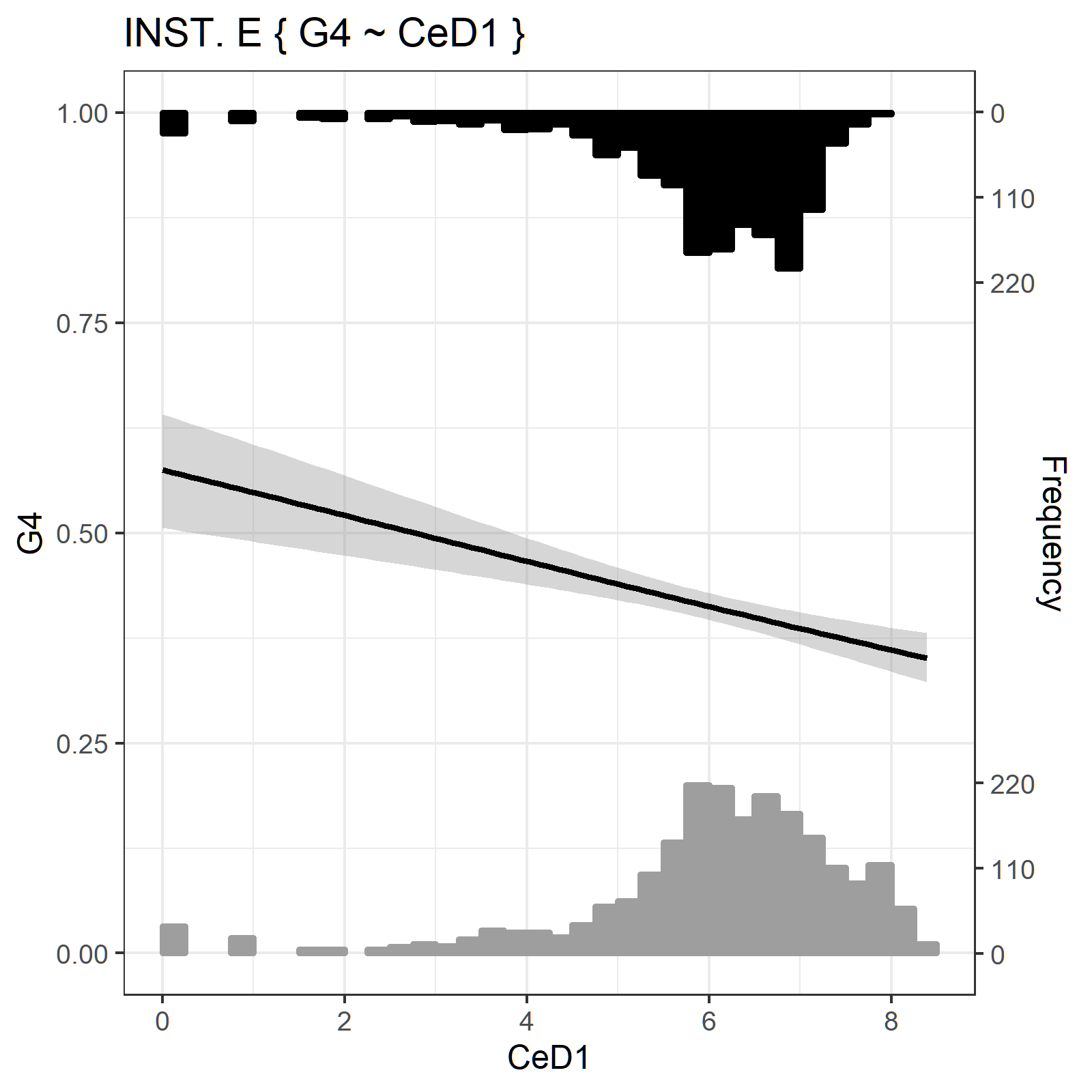

Supplement: Supplementary file 1 [file mmc1.zip › SupplementaryMaterials/416-LogitCurve.png]

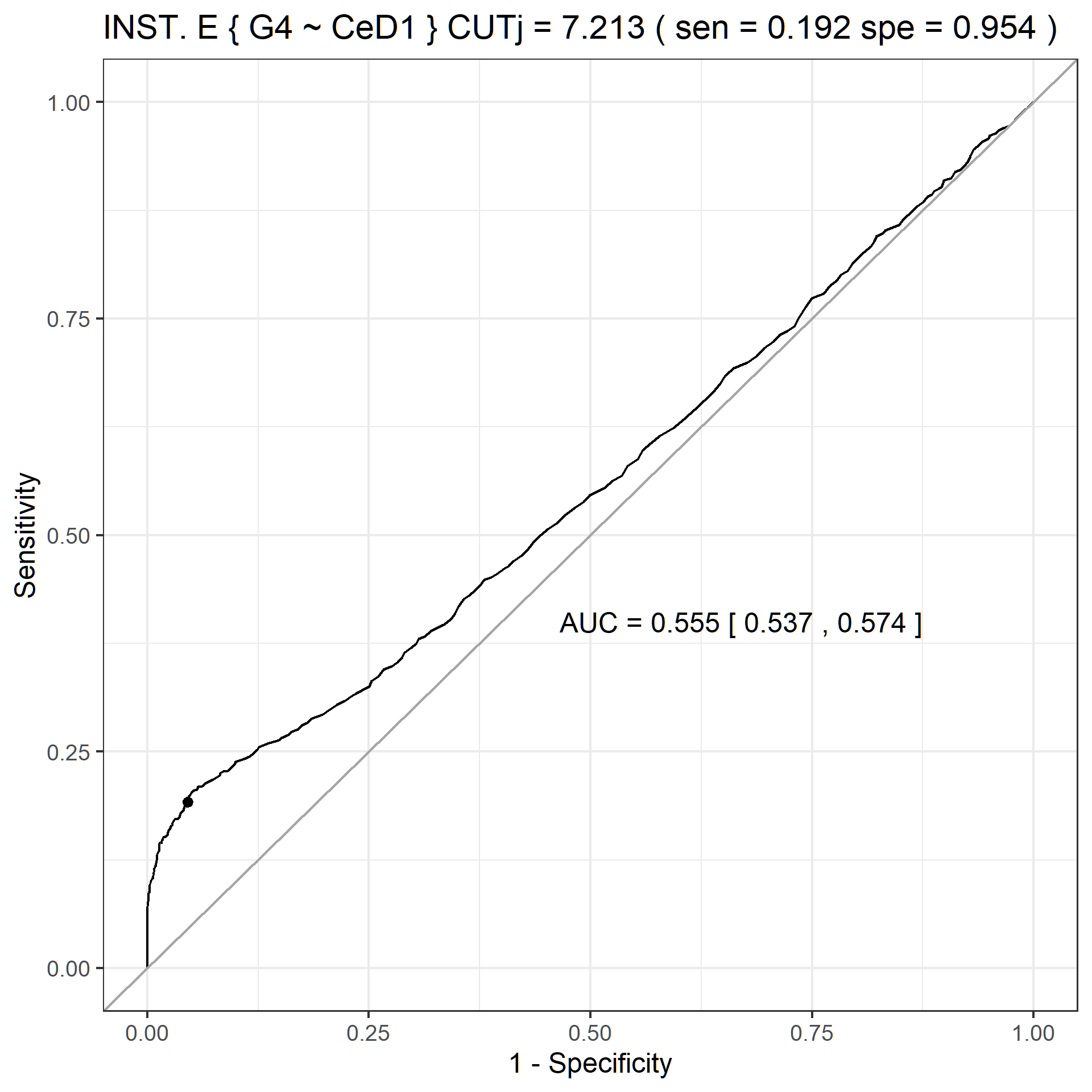

Supplement: Supplementary file 1 [file mmc1.zip › SupplementaryMaterials/416-ROCut.png]

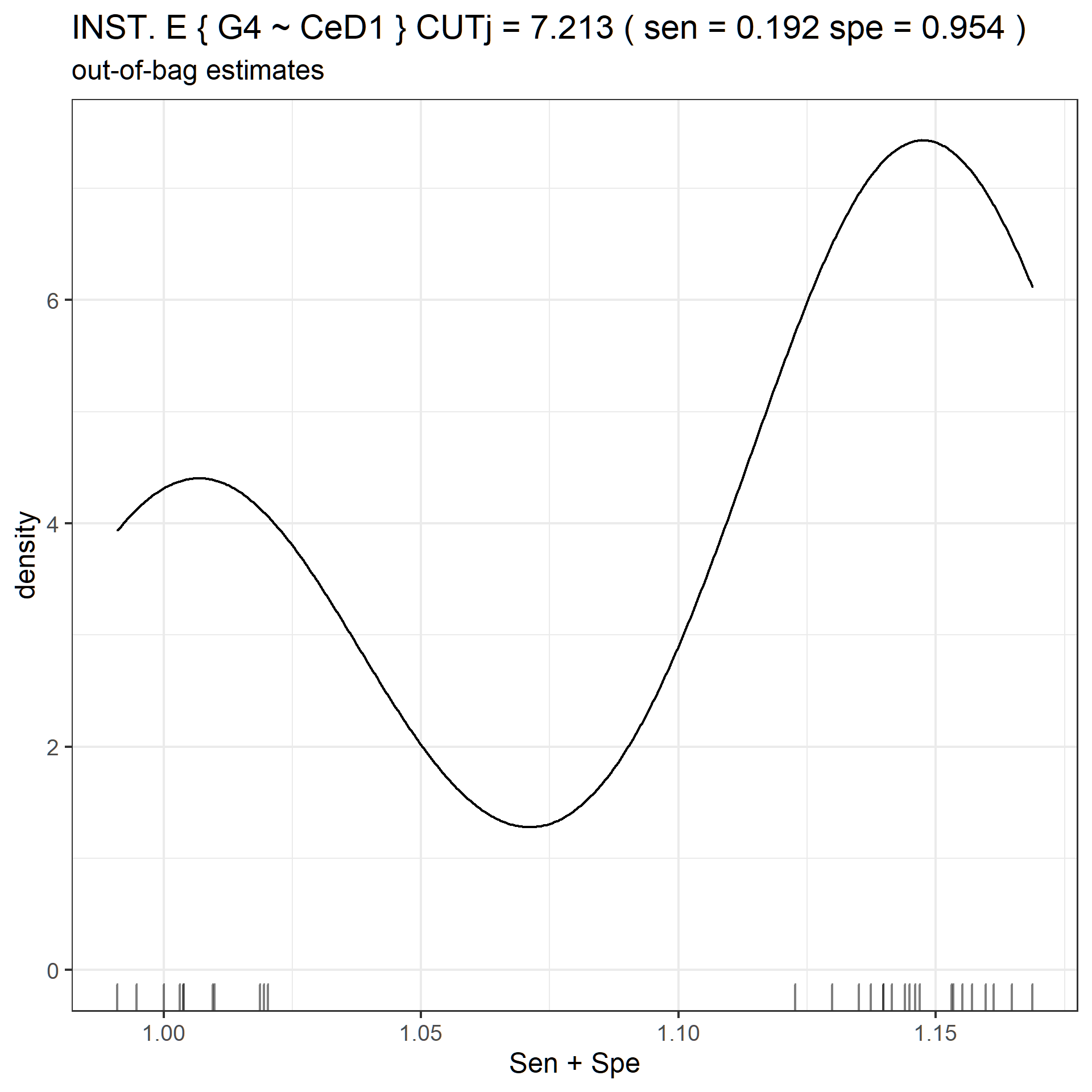

Supplement: Supplementary file 1 [file mmc1.zip › SupplementaryMaterials/416-SenSpe.png]

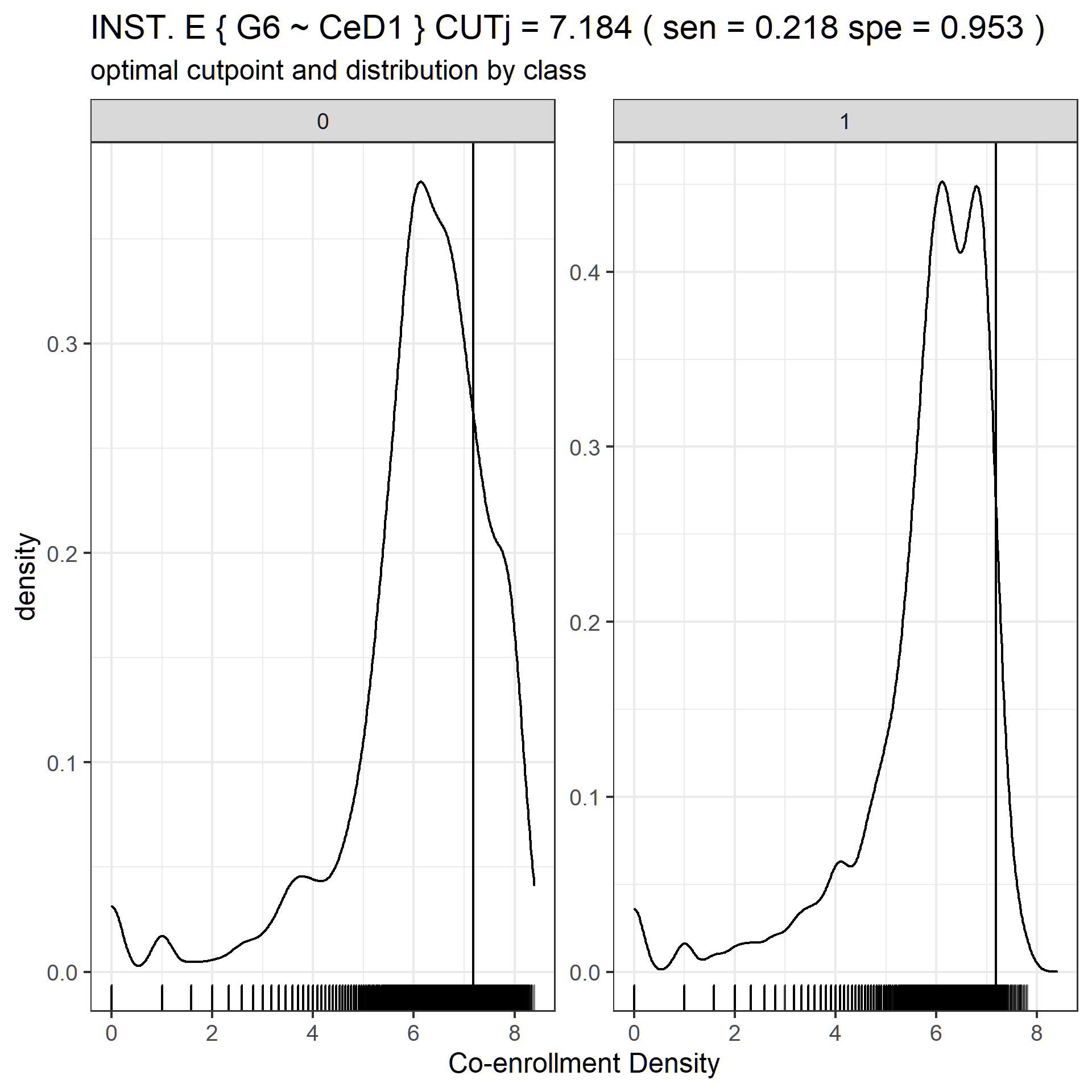

Supplement: Supplementary file 1 [file mmc1.zip › SupplementaryMaterials/417-ClassDen.png]

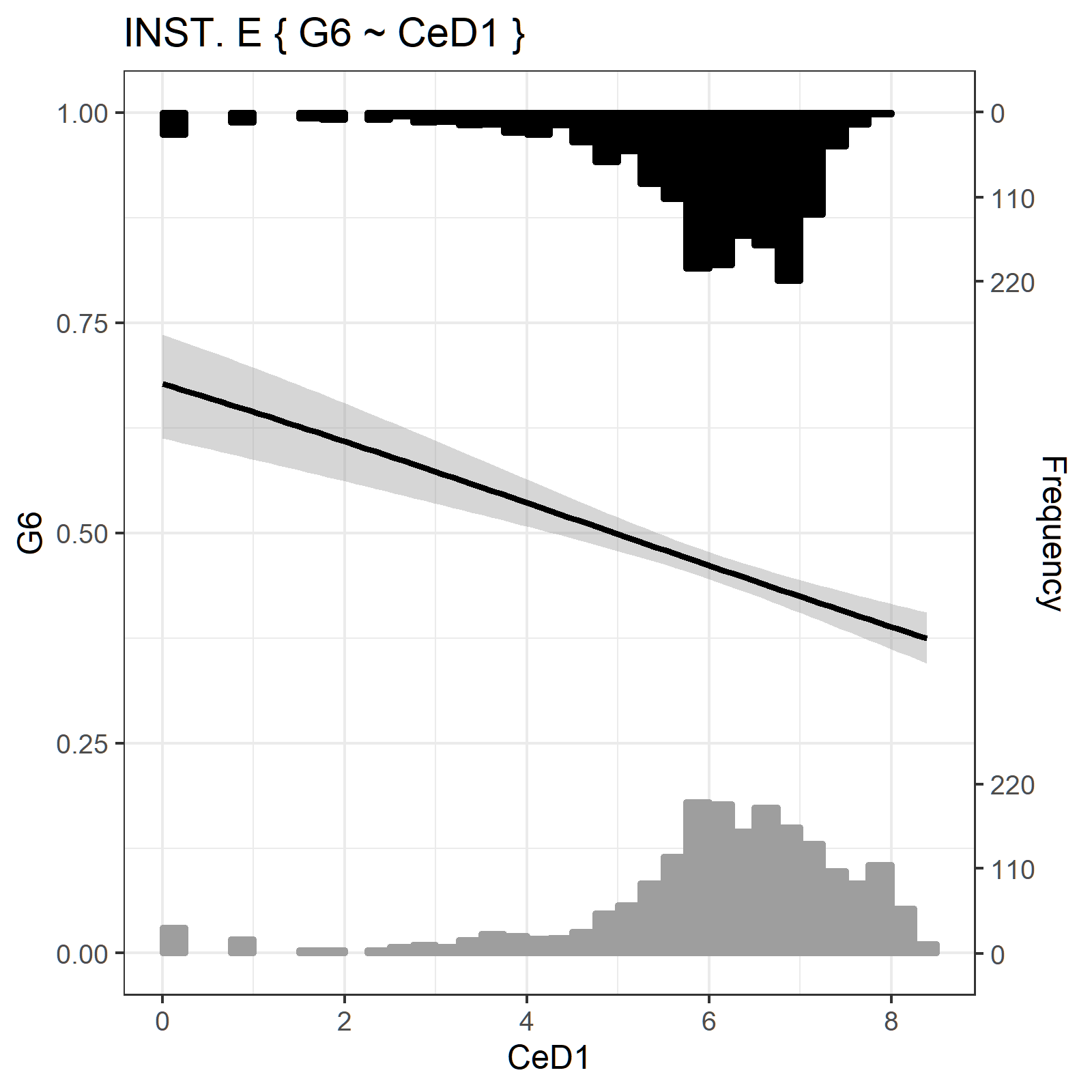

Supplement: Supplementary file 1 [file mmc1.zip › SupplementaryMaterials/417-LogitCurve.png]

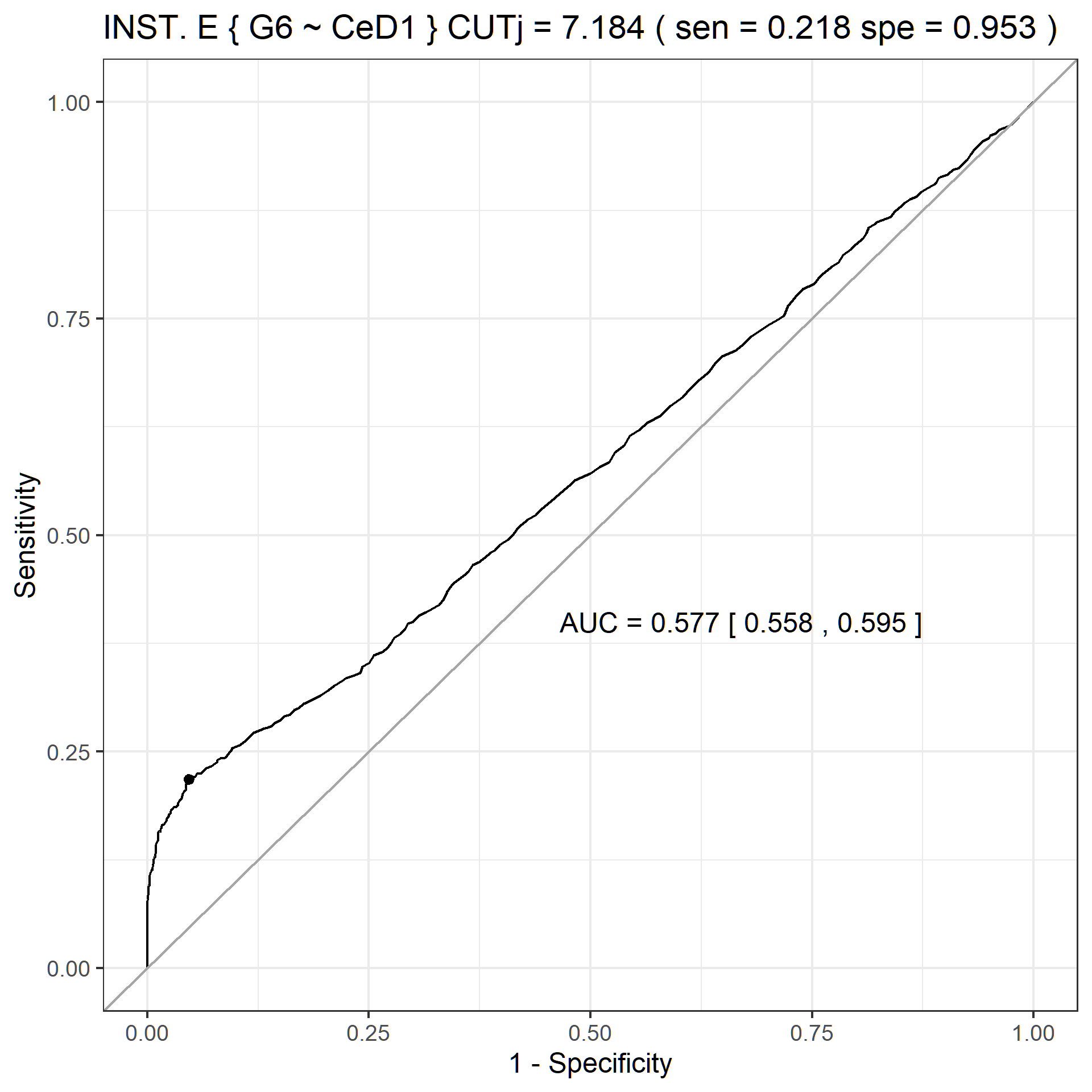

Supplement: Supplementary file 1 [file mmc1.zip › SupplementaryMaterials/417-ROCut.png]

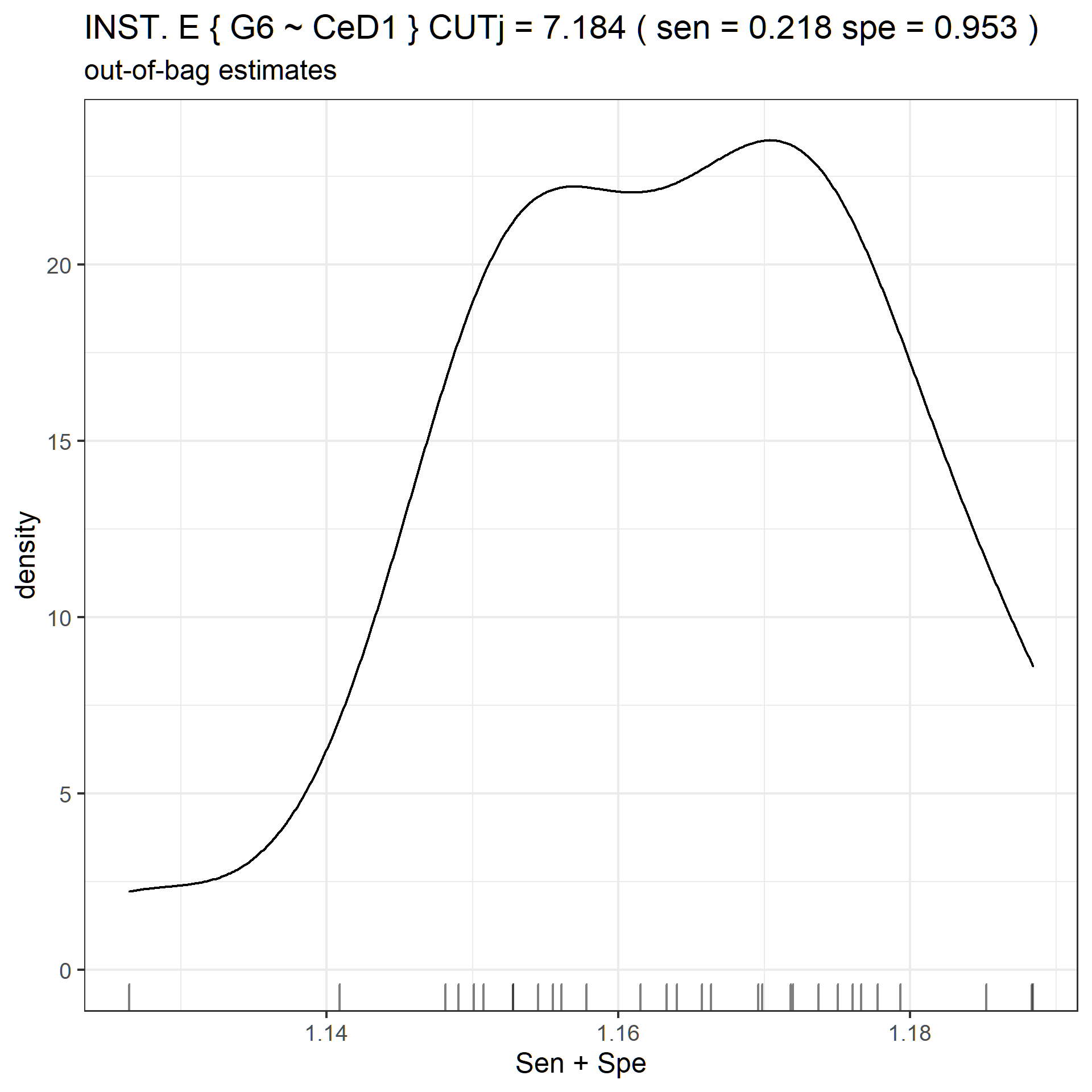

Supplement: Supplementary file 1 [file mmc1.zip › SupplementaryMaterials/417-SenSpe.png]

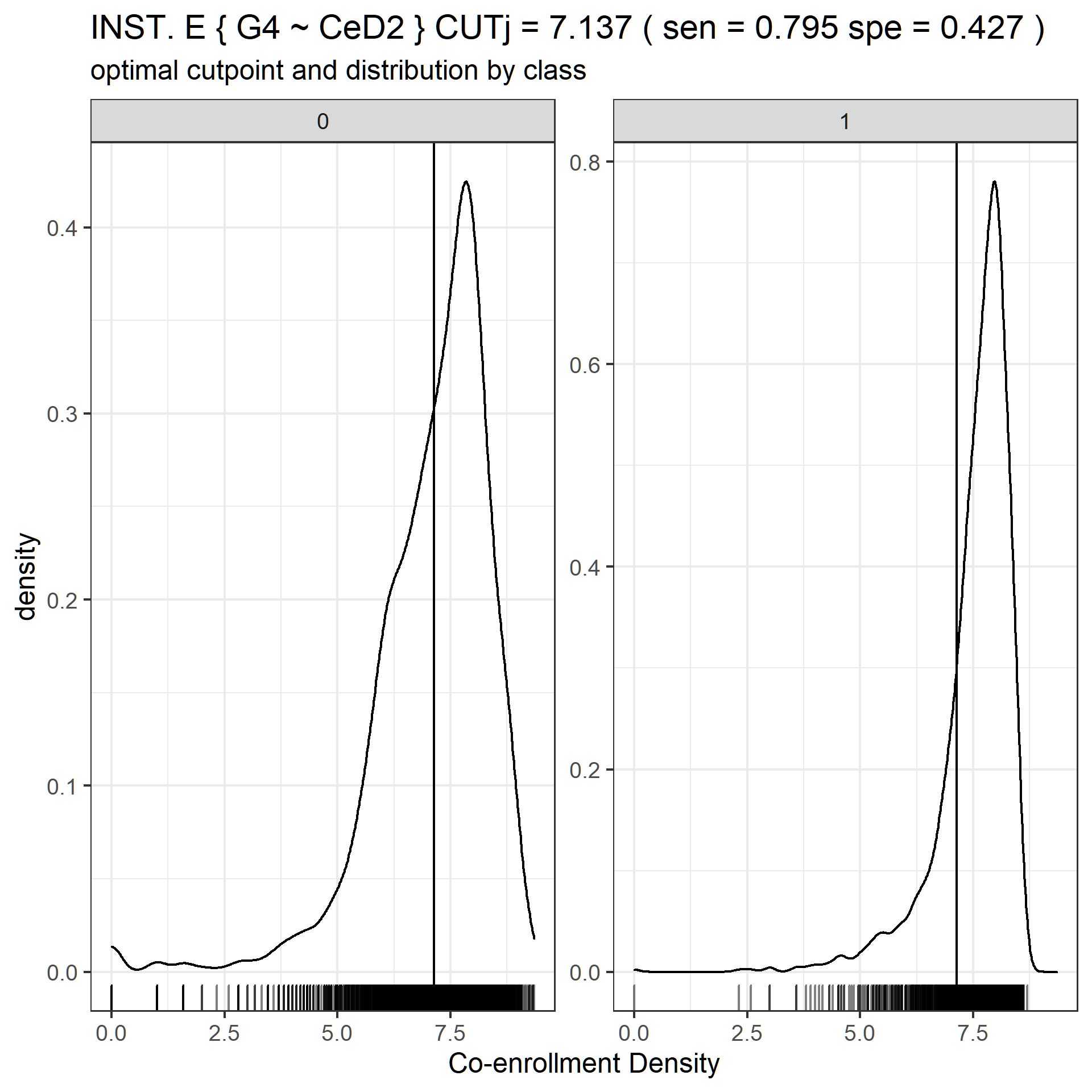

Supplement: Supplementary file 1 [file mmc1.zip › SupplementaryMaterials/426-ClassDen.png]

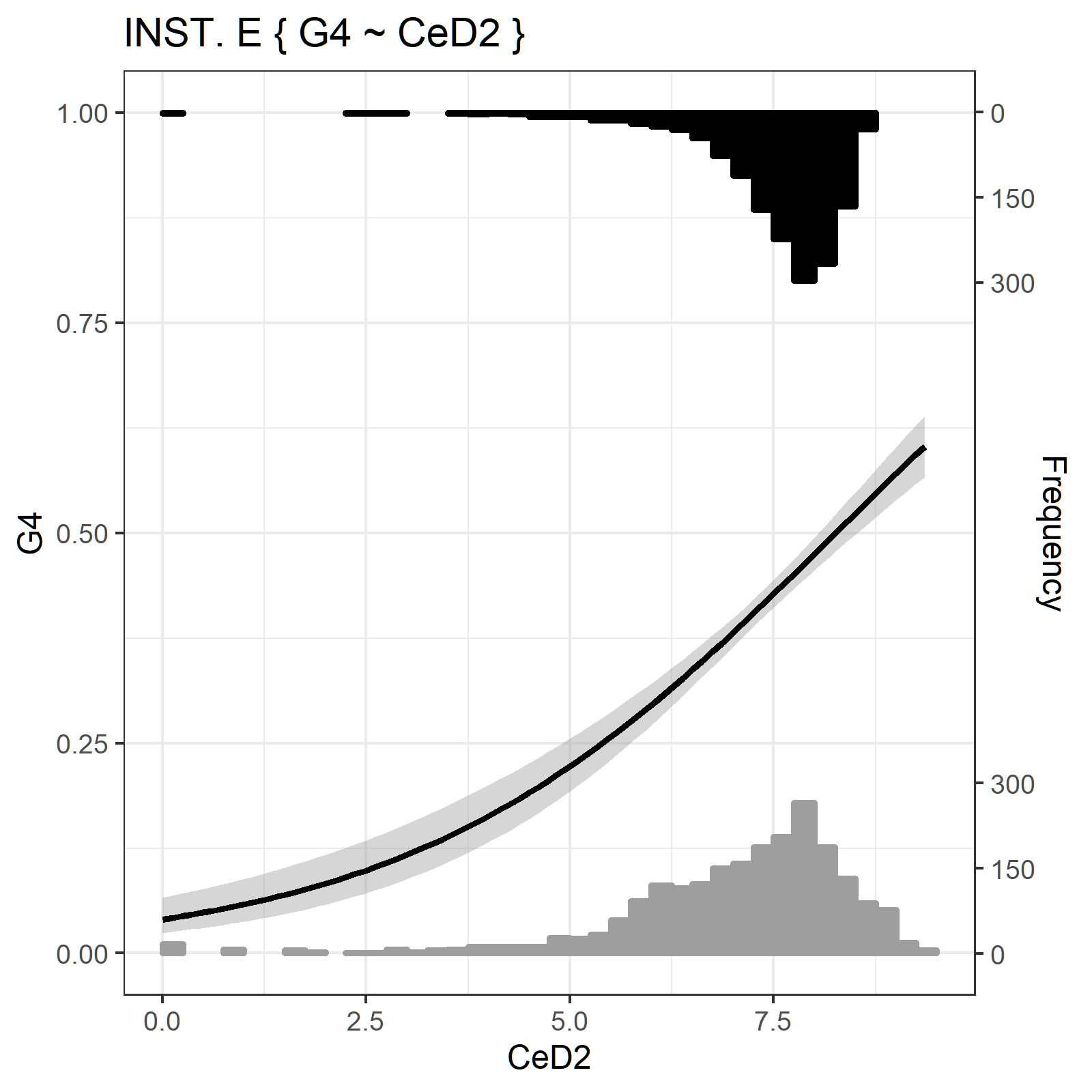

Supplement: Supplementary file 1 [file mmc1.zip › SupplementaryMaterials/426-LogitCurve.png]

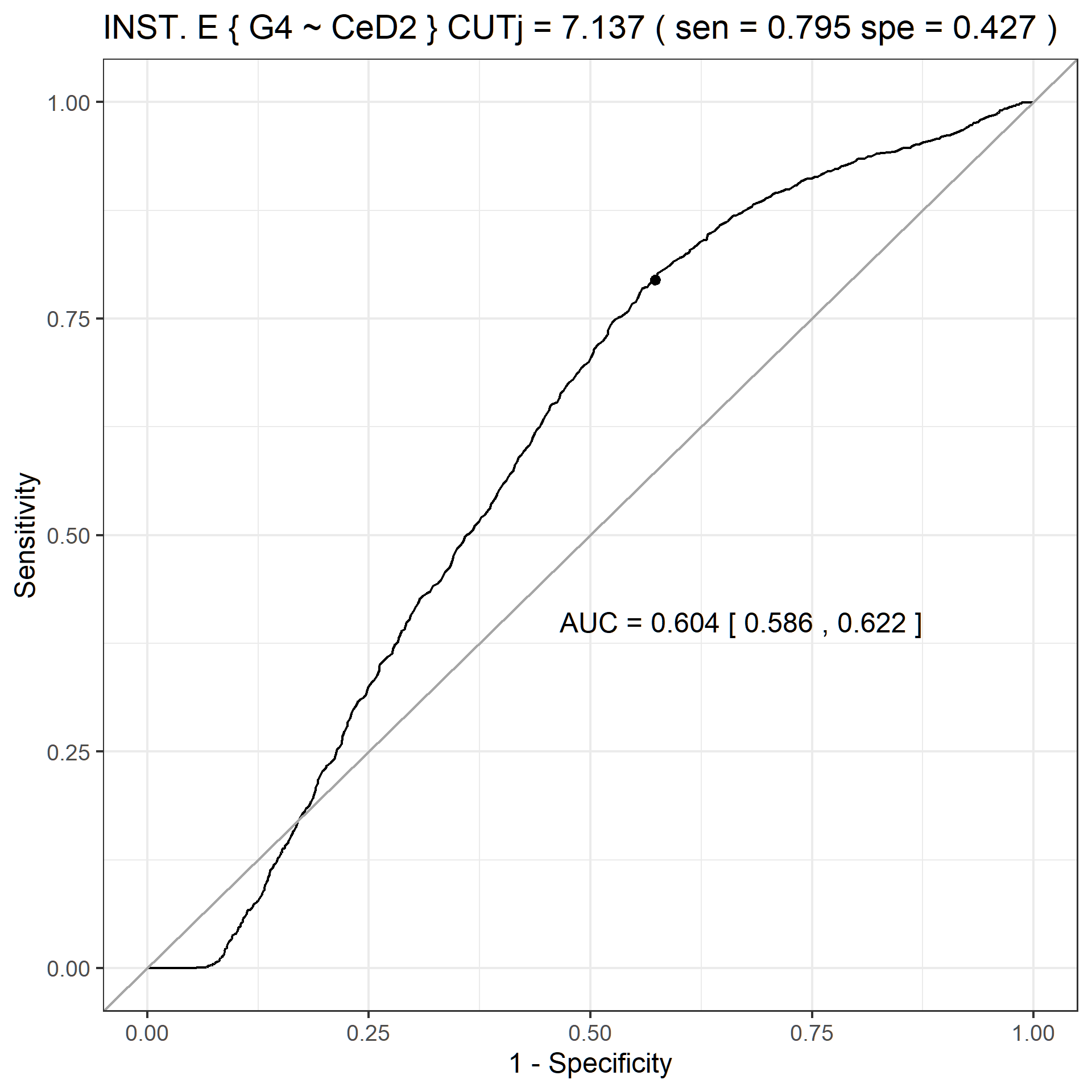

Supplement: Supplementary file 1 [file mmc1.zip › SupplementaryMaterials/426-ROCut.png]

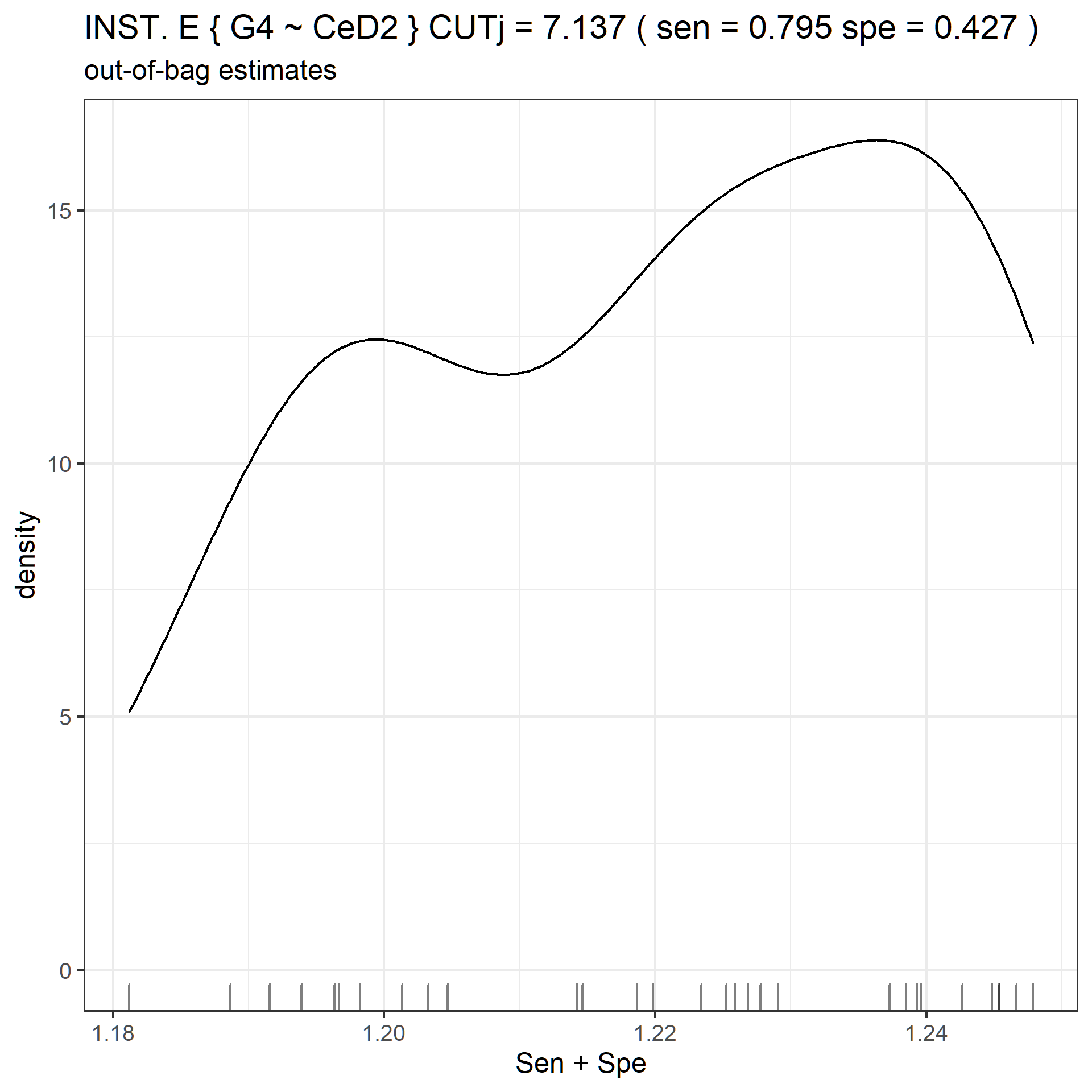

Supplement: Supplementary file 1 [file mmc1.zip › SupplementaryMaterials/426-SenSpe.png]

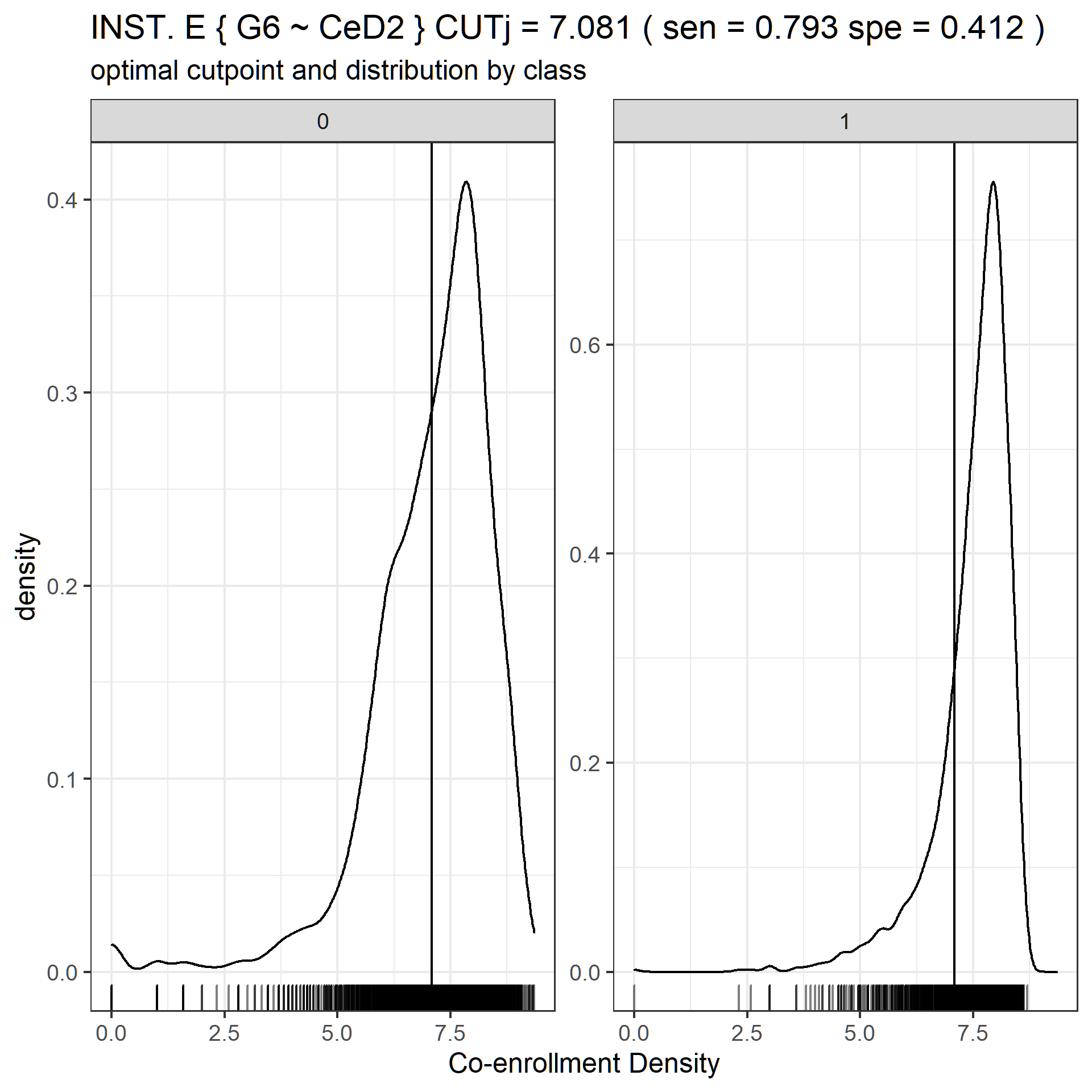

Supplement: Supplementary file 1 [file mmc1.zip › SupplementaryMaterials/427-ClassDen.png]

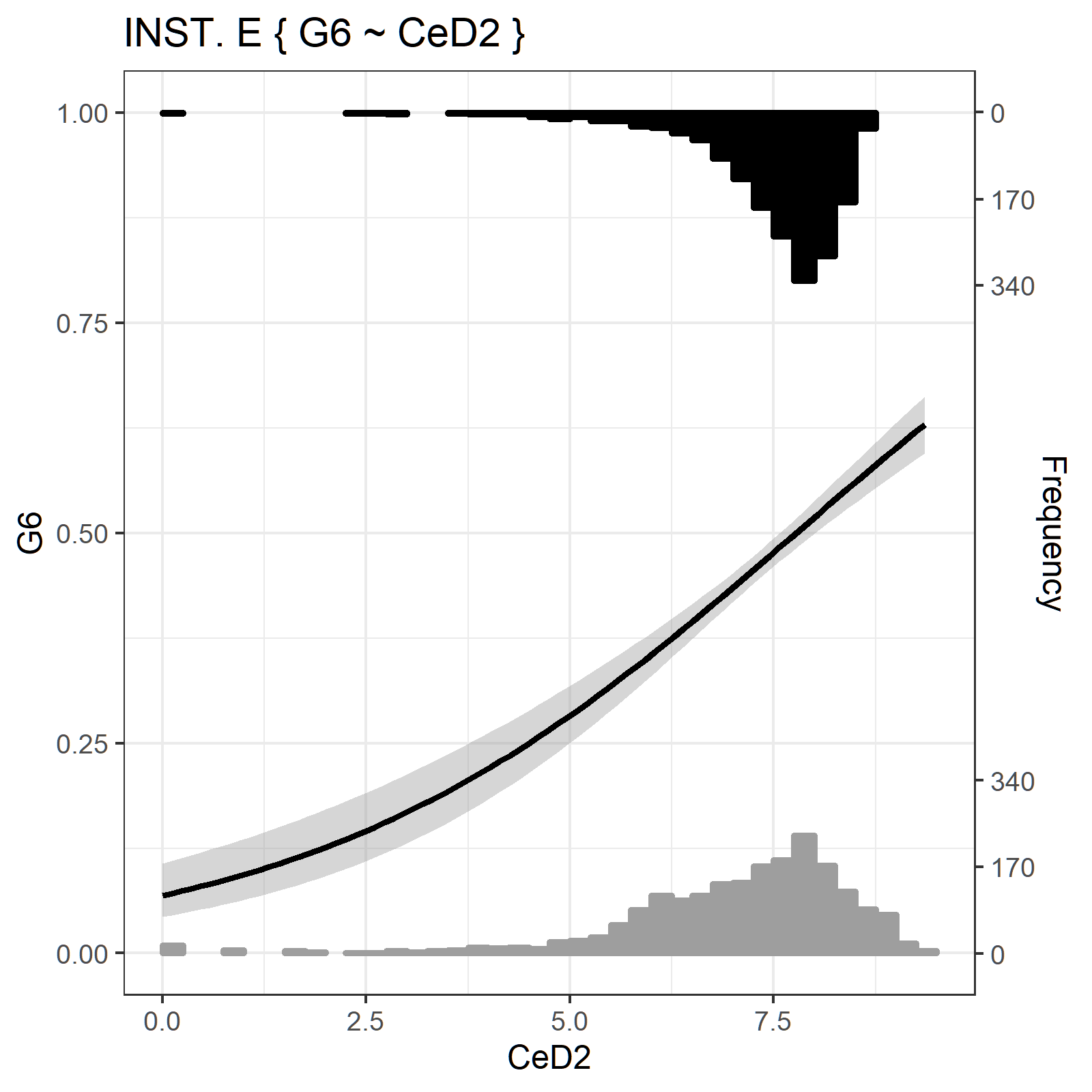

Supplement: Supplementary file 1 [file mmc1.zip › SupplementaryMaterials/427-LogitCurve.png]

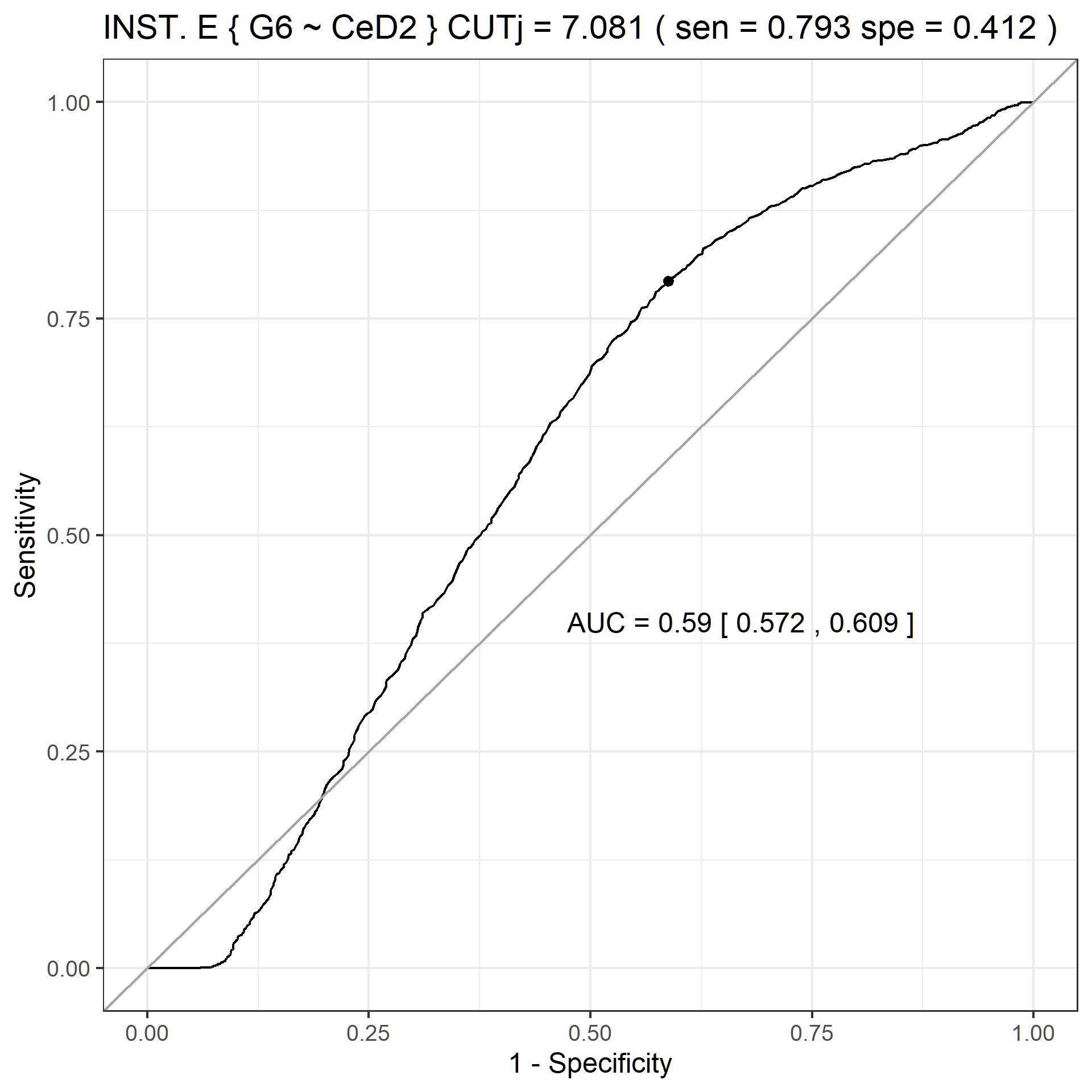

Supplement: Supplementary file 1 [file mmc1.zip › SupplementaryMaterials/427-ROCut.png]

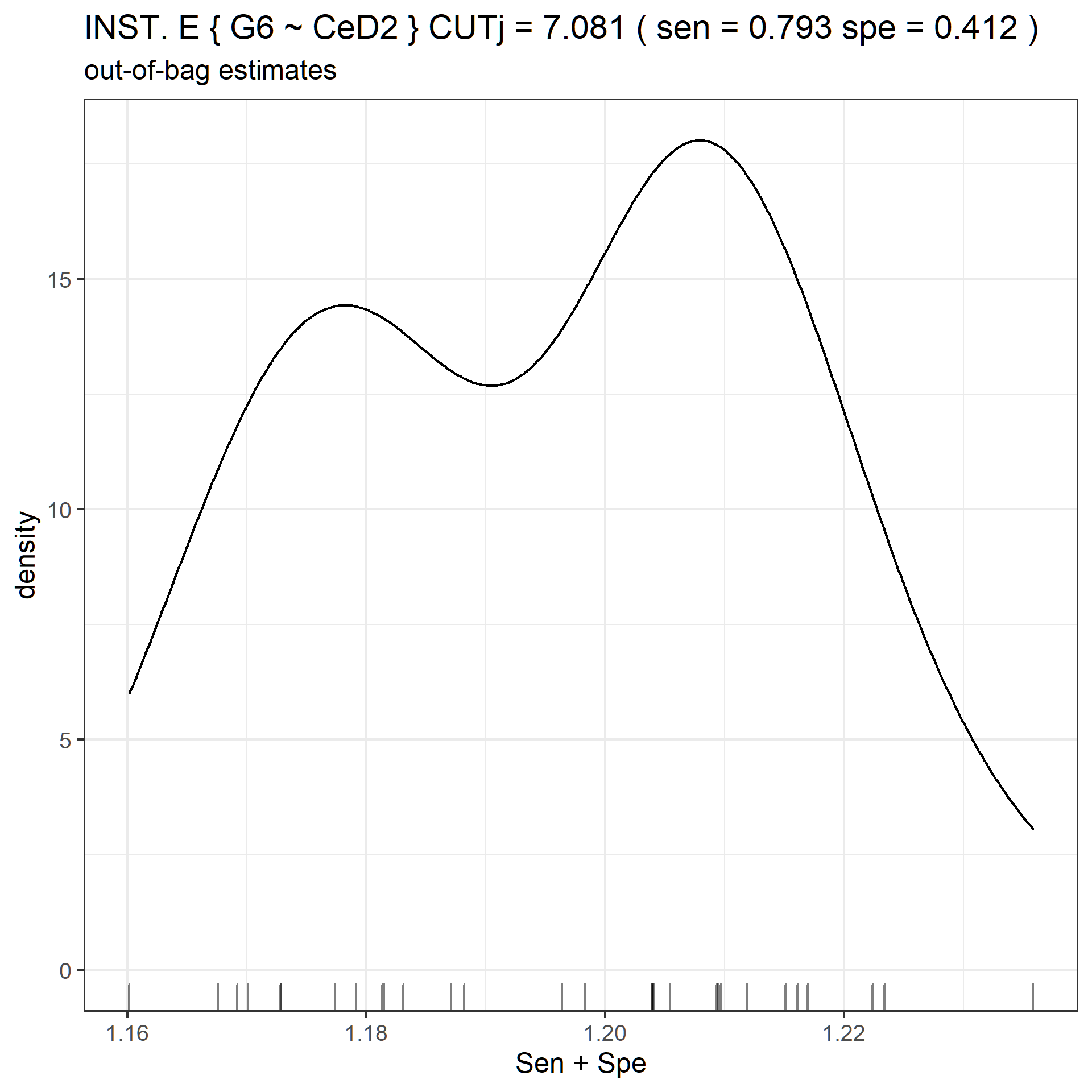

Supplement: Supplementary file 1 [file mmc1.zip › SupplementaryMaterials/427-SenSpe.png]

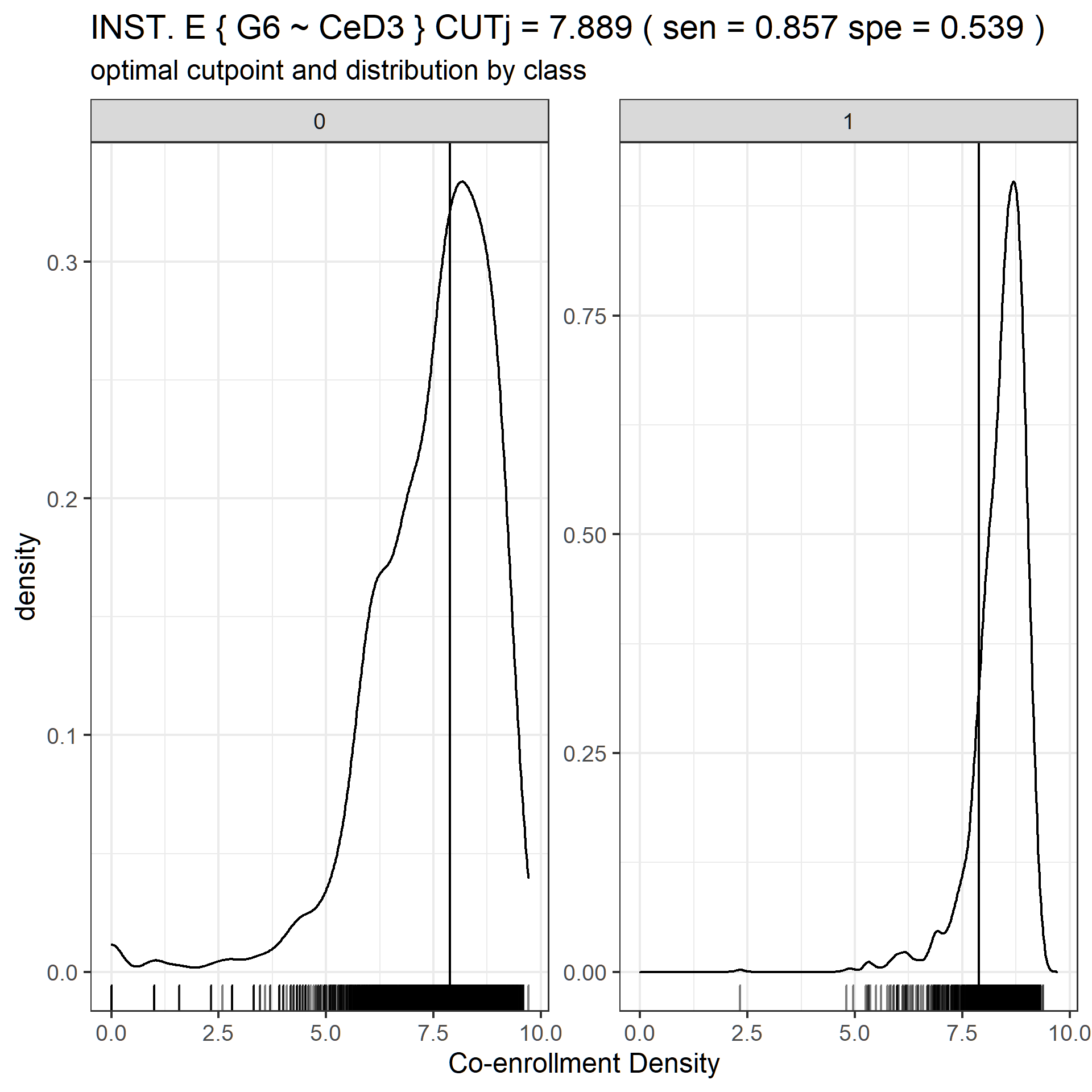

Supplement: Supplementary file 1 [file mmc1.zip › SupplementaryMaterials/437-ClassDen.png]

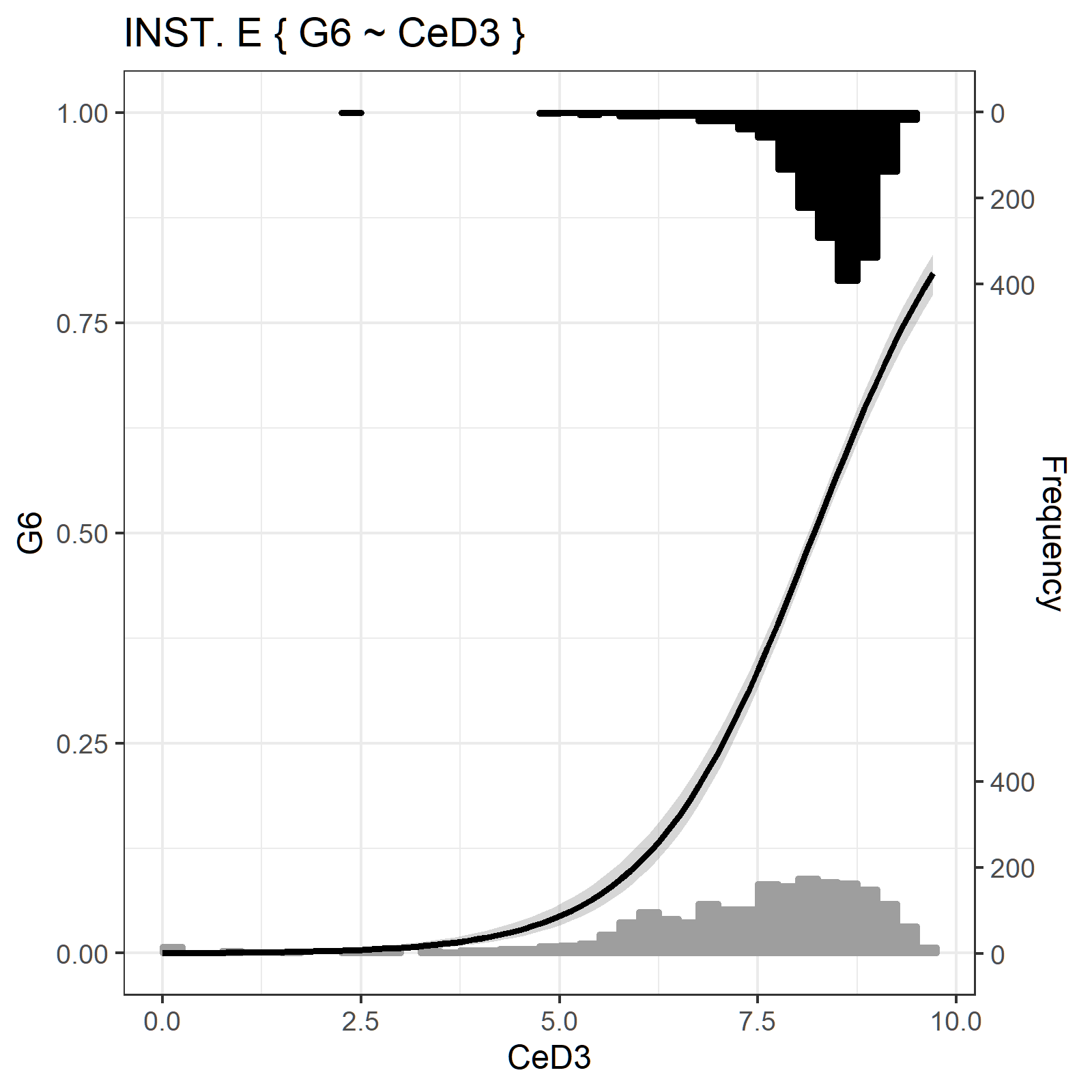

Supplement: Supplementary file 1 [file mmc1.zip › SupplementaryMaterials/437-LogitCurve.png]

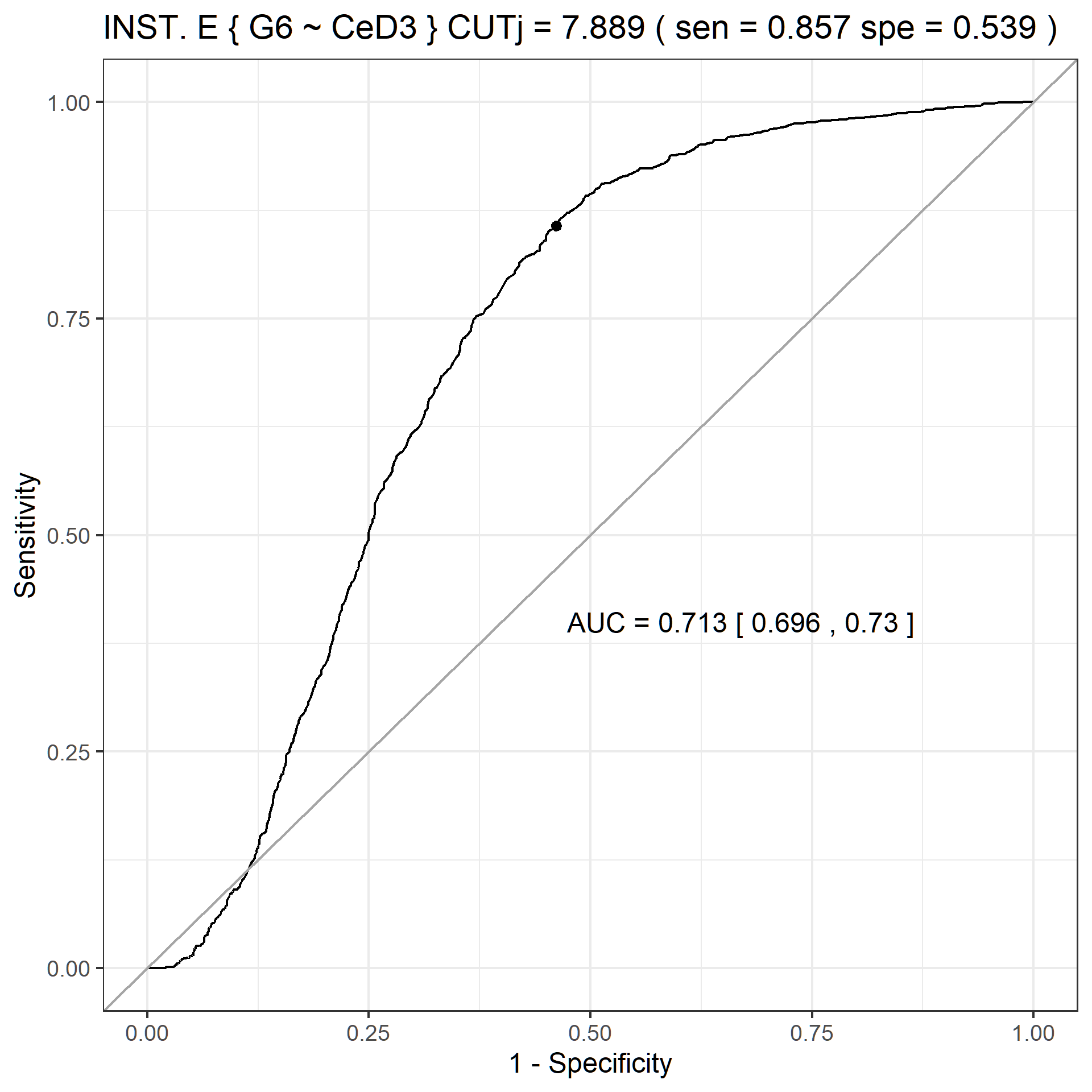

Supplement: Supplementary file 1 [file mmc1.zip › SupplementaryMaterials/437-ROCut.png]

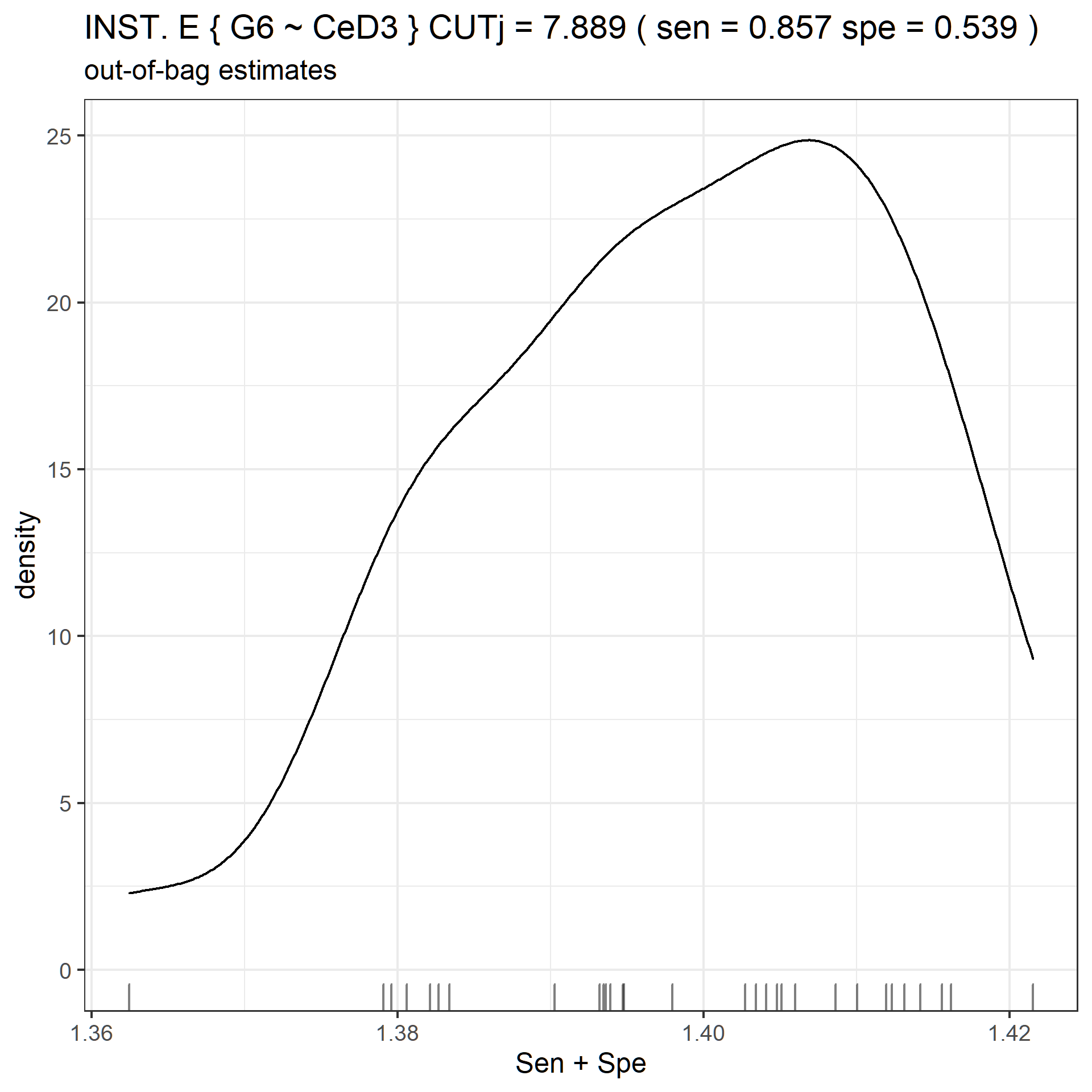

Supplement: Supplementary file 1 [file mmc1.zip › SupplementaryMaterials/437-SenSpe.png]

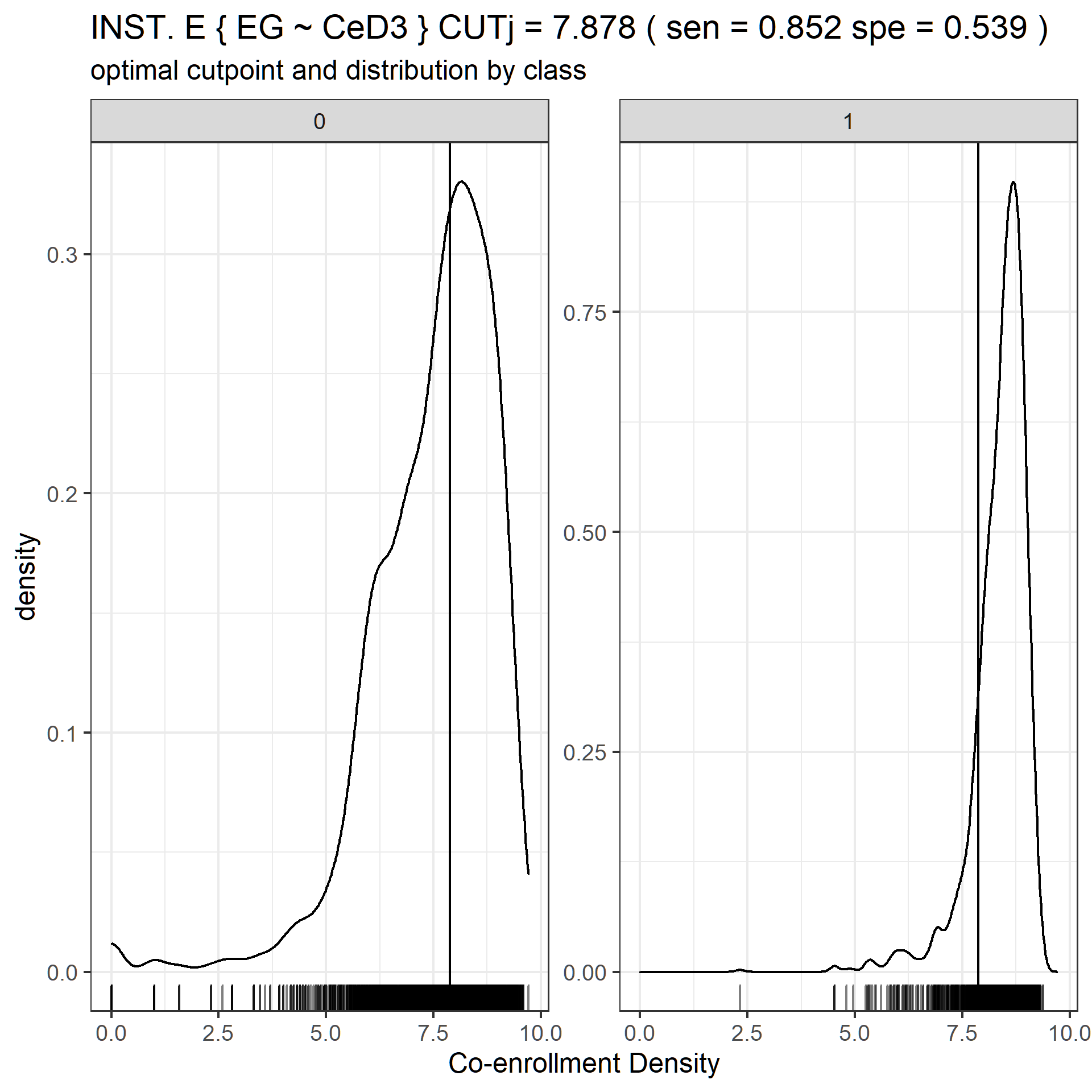

Supplement: Supplementary file 1 [file mmc1.zip › SupplementaryMaterials/438-ClassDen.png]

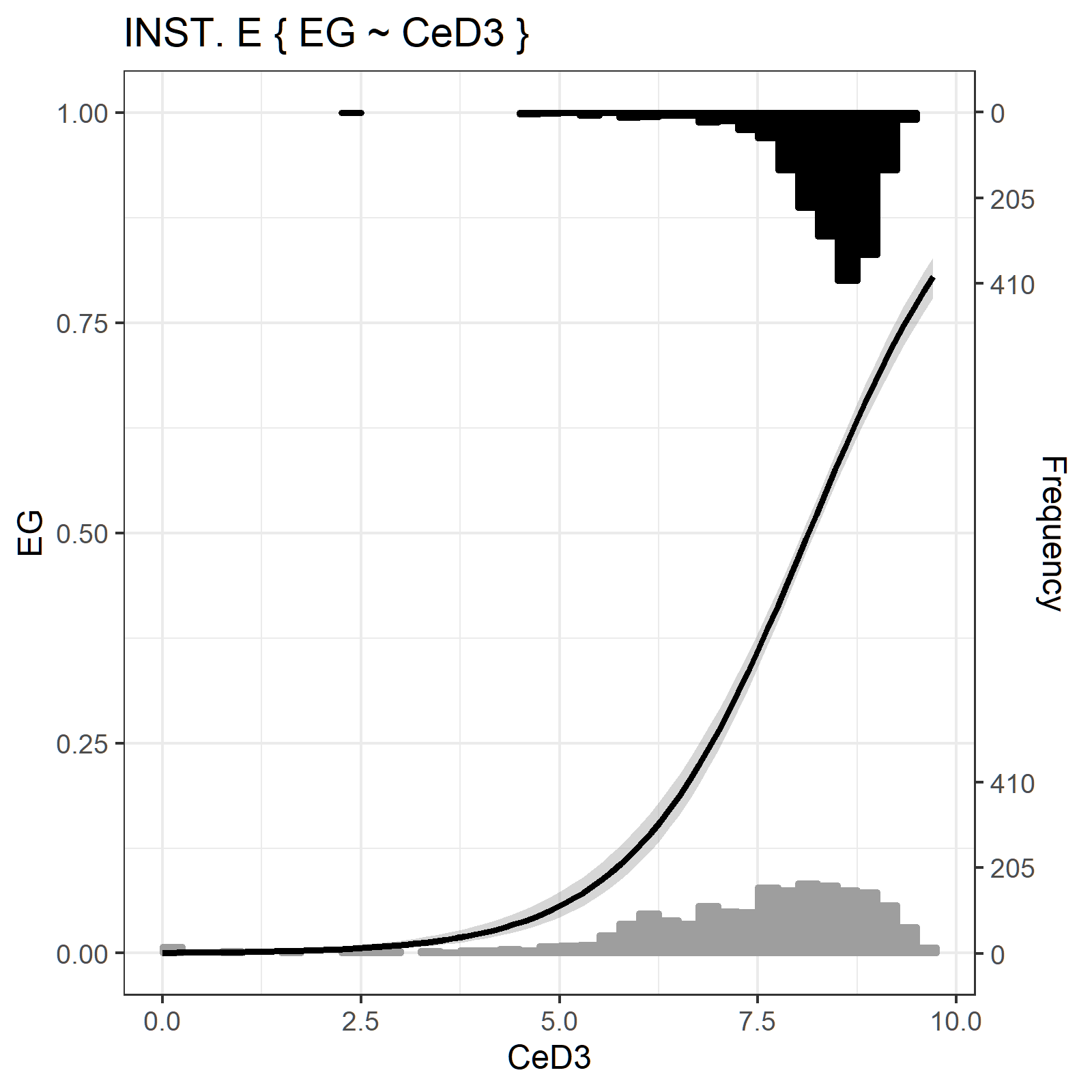

Supplement: Supplementary file 1 [file mmc1.zip › SupplementaryMaterials/438-LogitCurve.png]

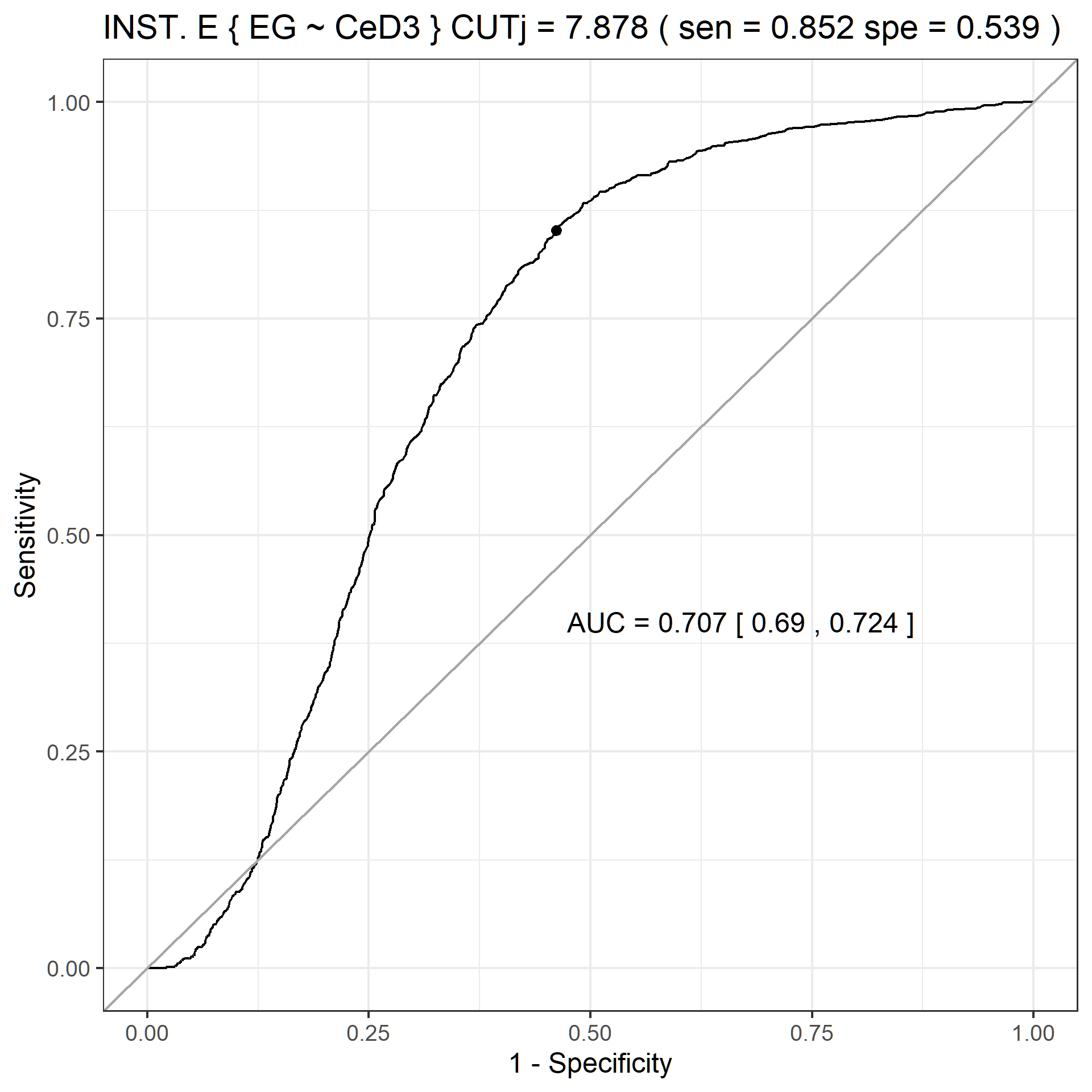

Supplement: Supplementary file 1 [file mmc1.zip › SupplementaryMaterials/438-ROCut.png]

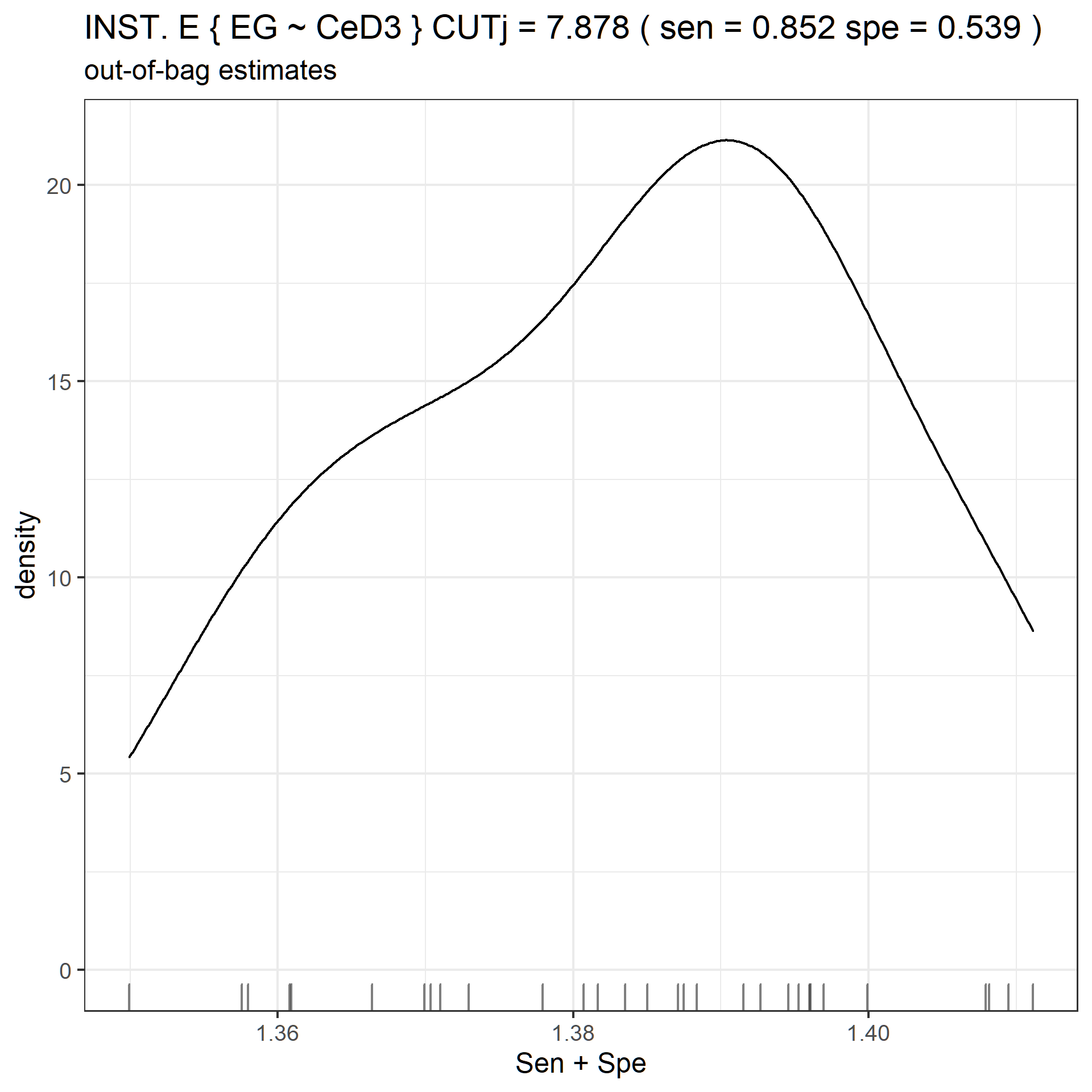

Supplement: Supplementary file 1 [file mmc1.zip › SupplementaryMaterials/438-SenSpe.png]

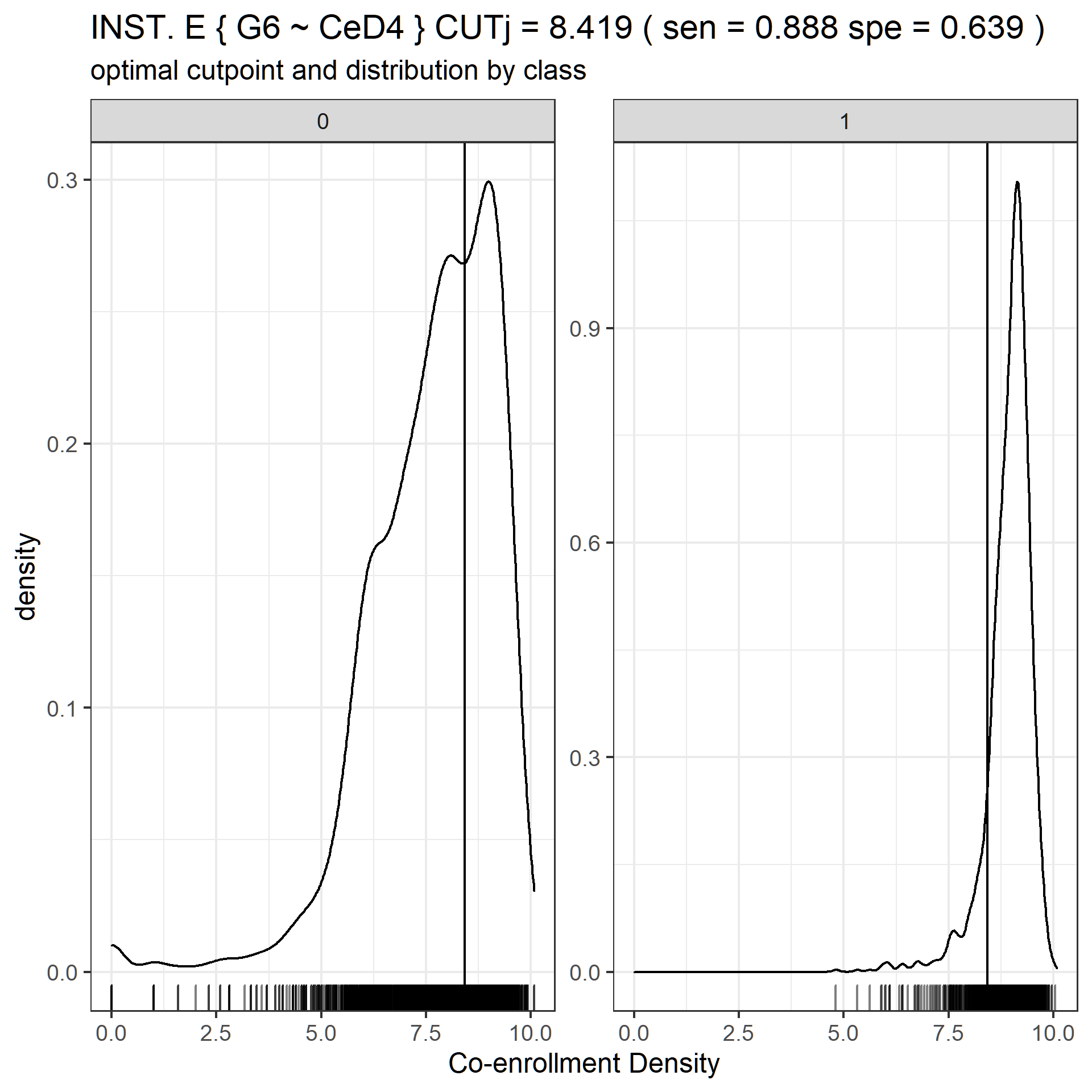

Supplement: Supplementary file 1 [file mmc1.zip › SupplementaryMaterials/447-ClassDen.png]

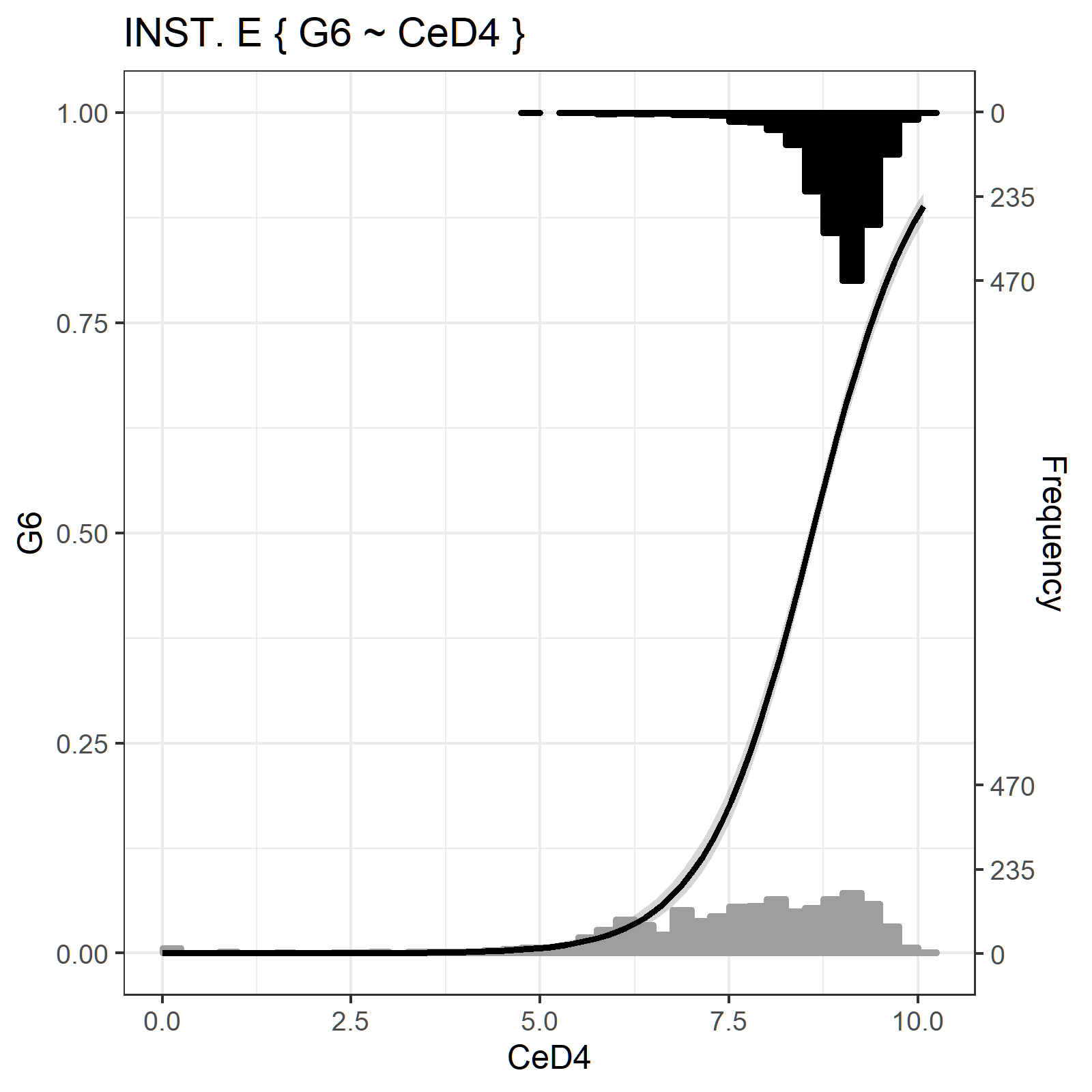

Supplement: Supplementary file 1 [file mmc1.zip › SupplementaryMaterials/447-LogitCurve.png]

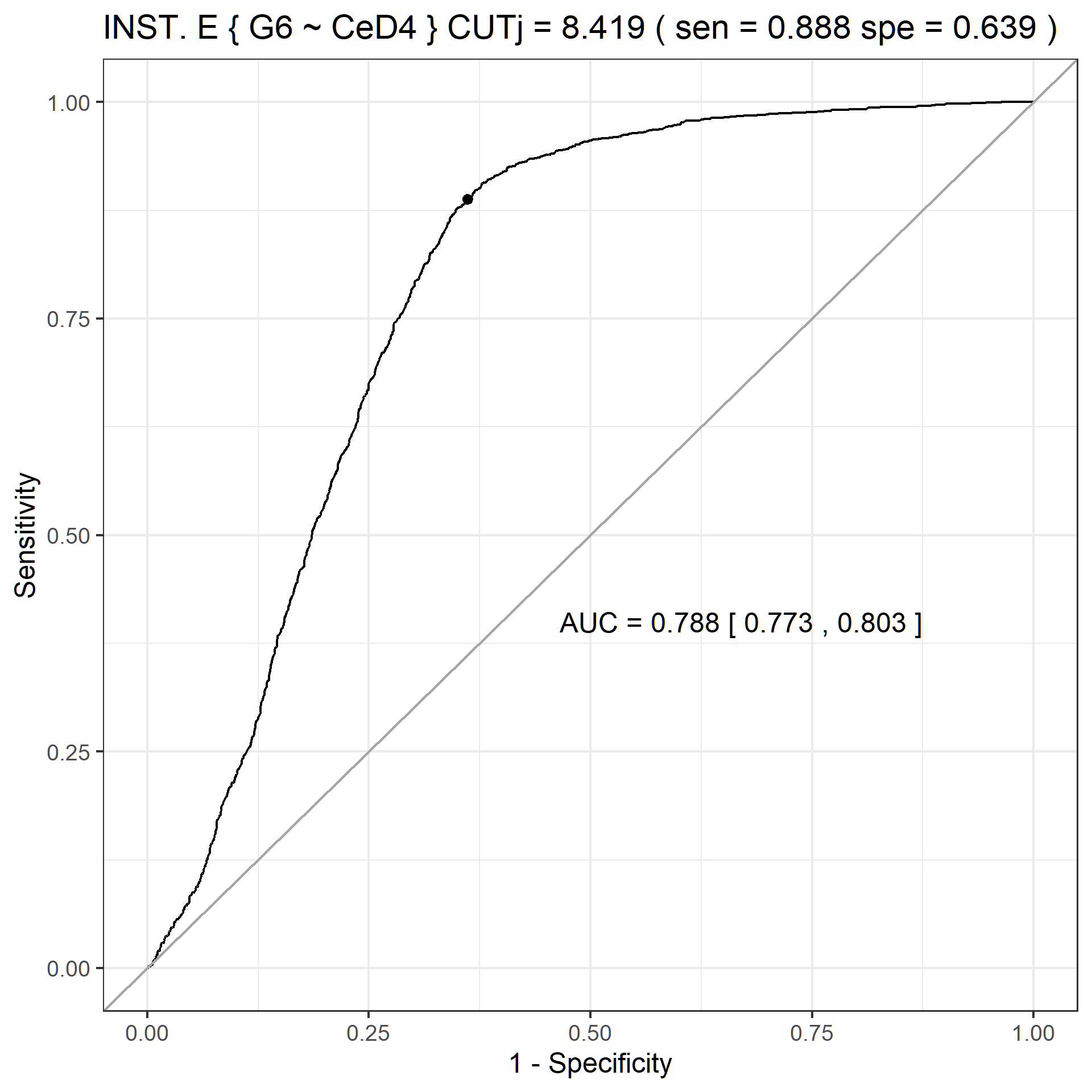

Supplement: Supplementary file 1 [file mmc1.zip › SupplementaryMaterials/447-ROCut.png]

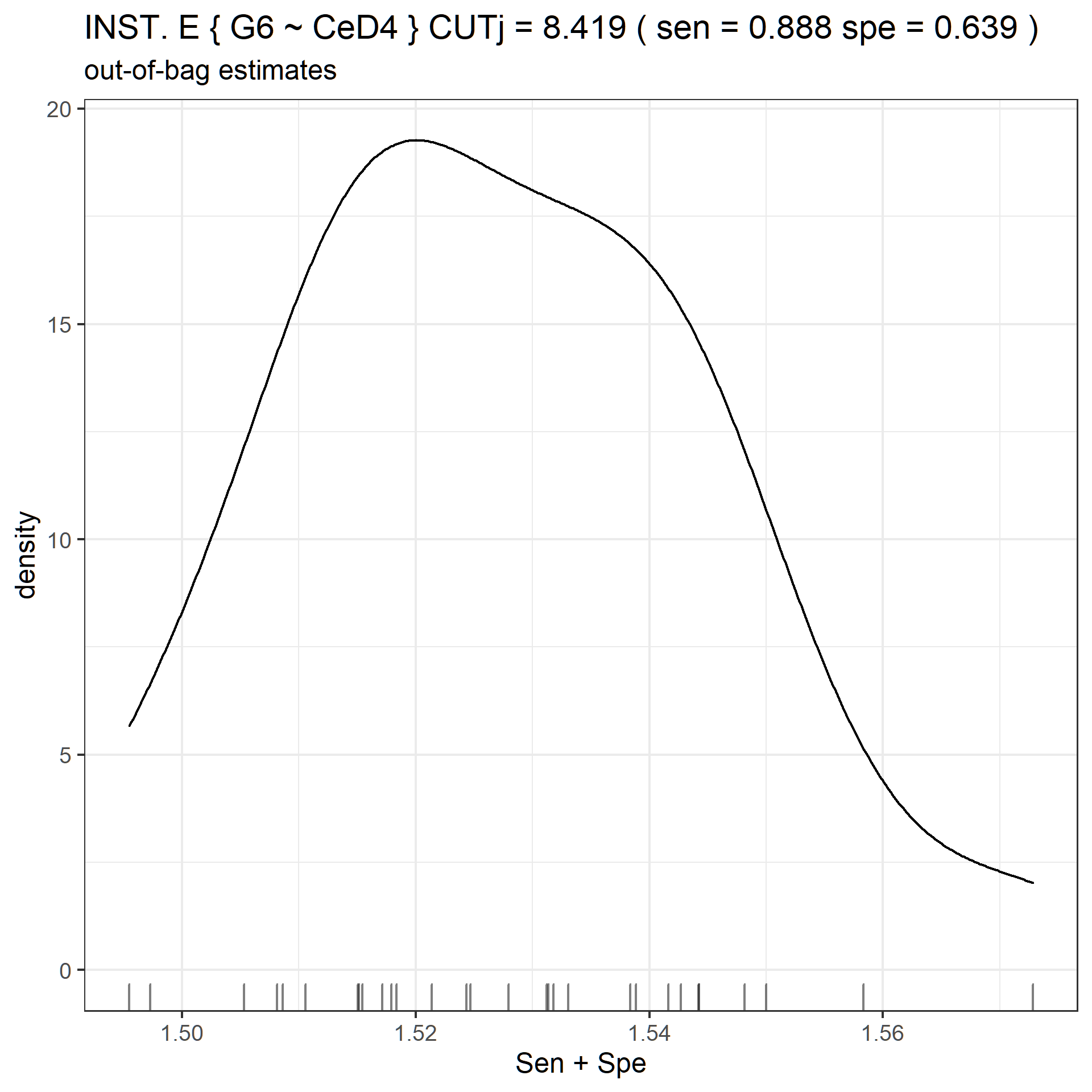

Supplement: Supplementary file 1 [file mmc1.zip › SupplementaryMaterials/447-SenSpe.png]
