# Supplementary figures and images for: Logit models, the area under receiver characteristic curves, sensitivity, and specificity for Co-enrollment density in college networks dataset (part 1 of 2)
Source: Data Brief. 2021 Oct 26;39:107509. doi: 10.1016/j.dib.2021.107509 (PMC8573127; doi:10.1016/j.dib.2021.107509)

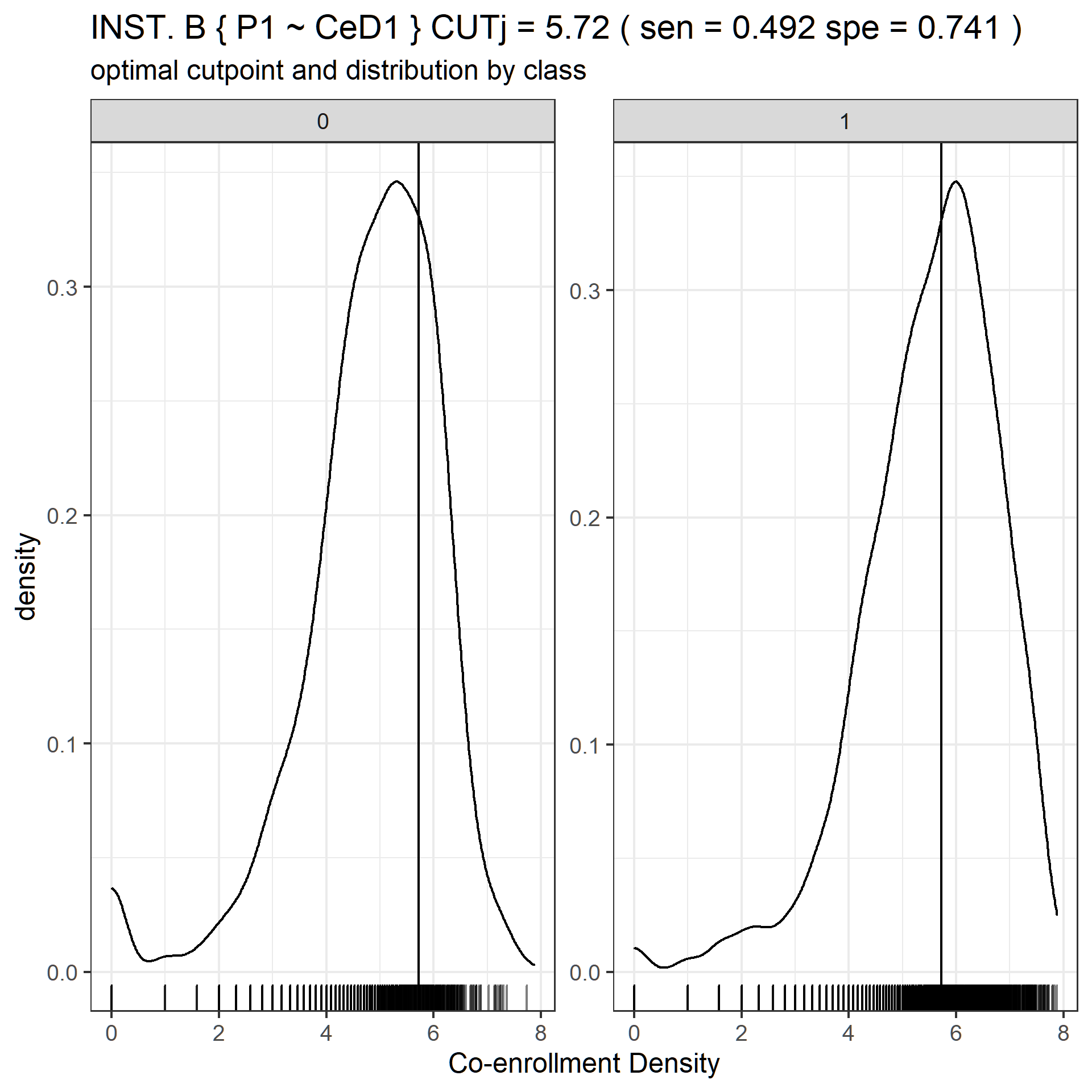

Supplement: Supplementary file 1 [file mmc1.zip › SupplementaryMaterials/115-ClassDen.png]

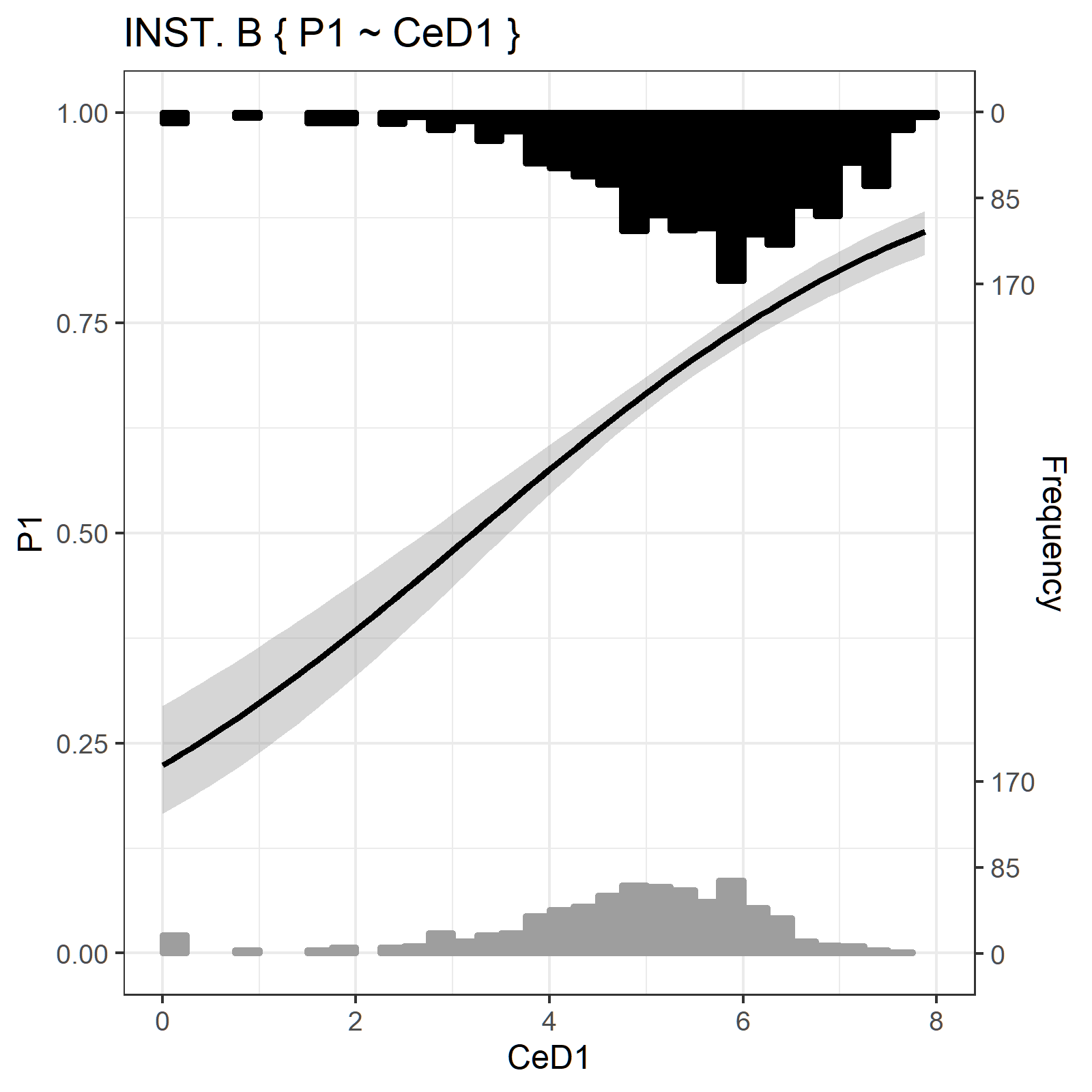

Supplement: Supplementary file 1 [file mmc1.zip › SupplementaryMaterials/115-LogitCurve.png]

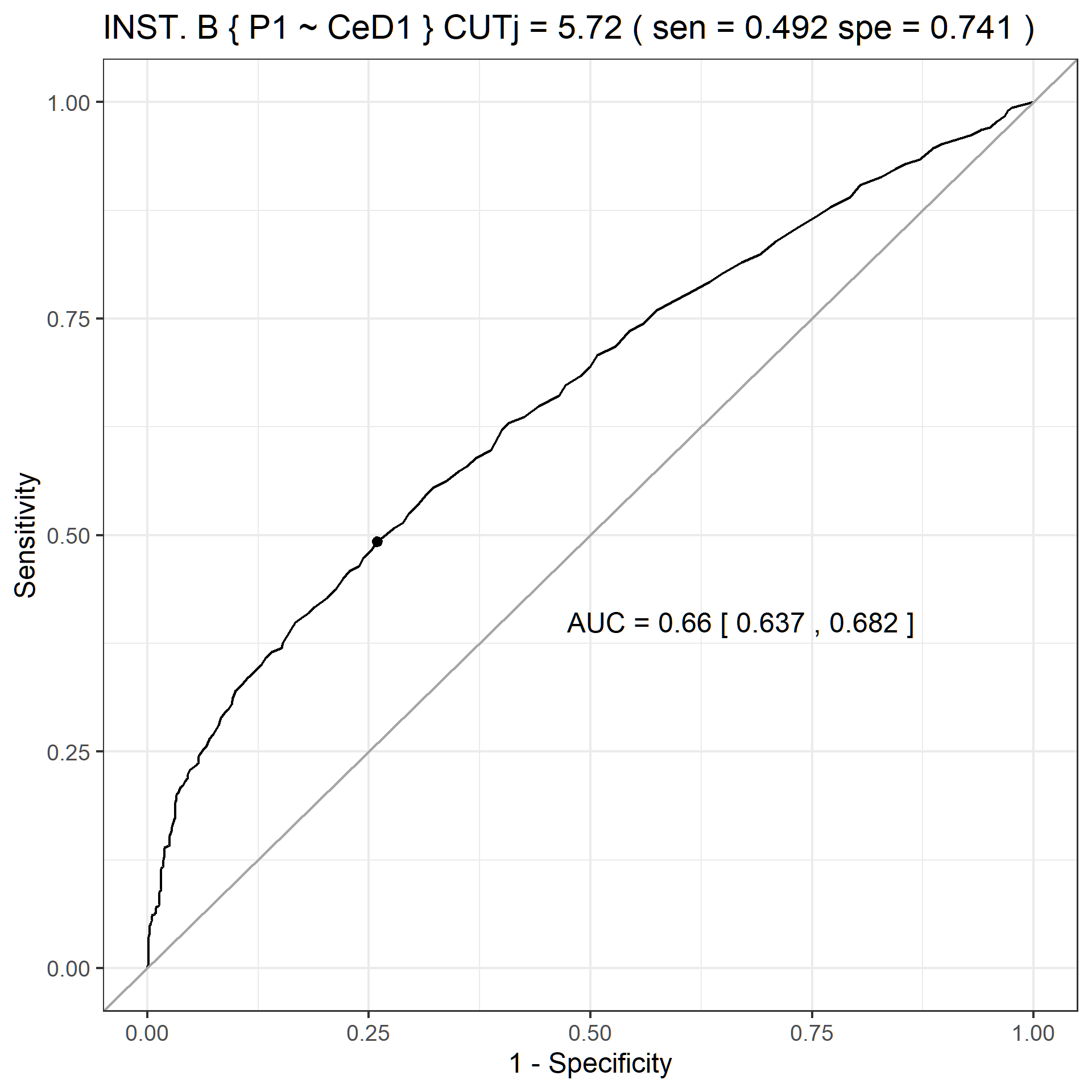

Supplement: Supplementary file 1 [file mmc1.zip › SupplementaryMaterials/115-ROCut.png]

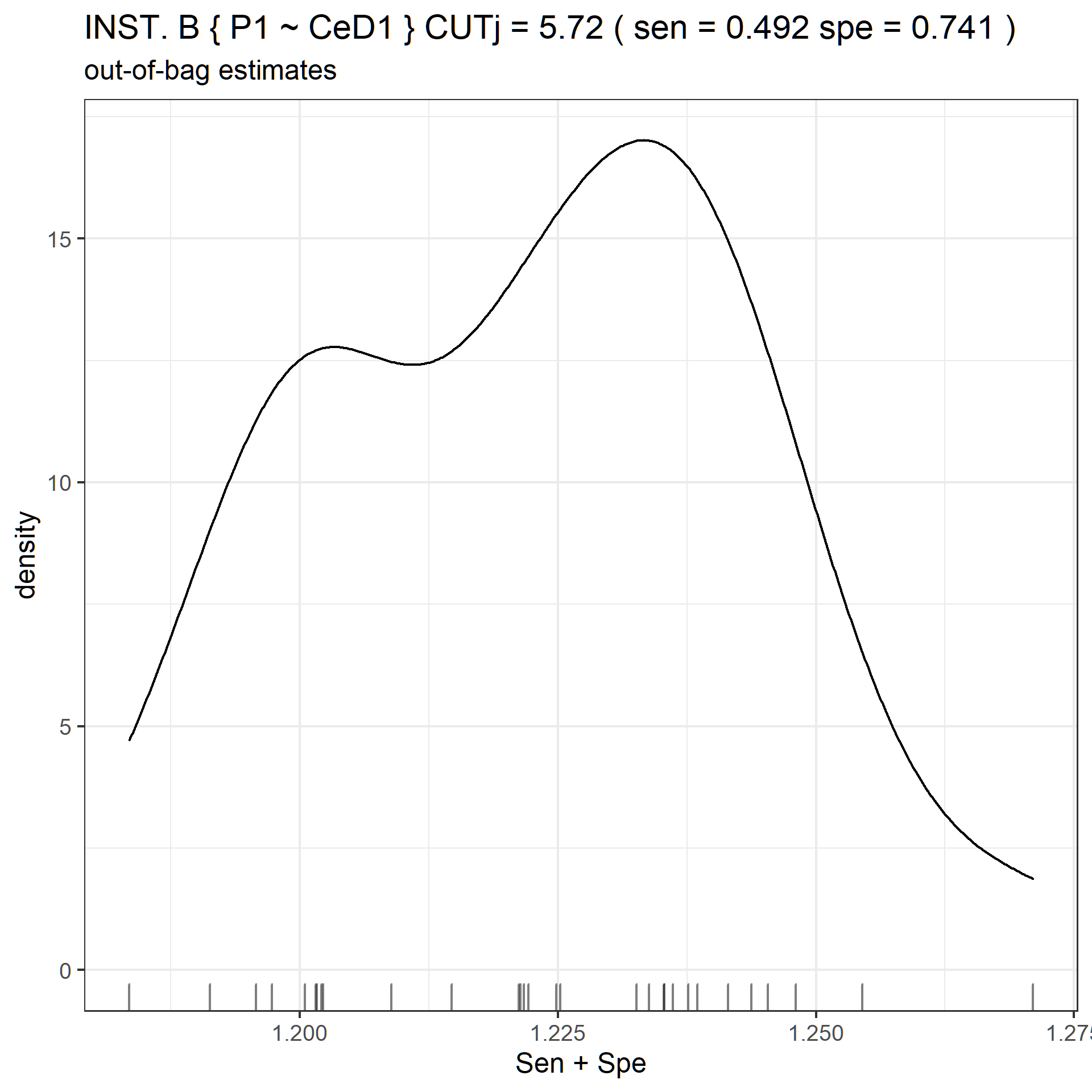

Supplement: Supplementary file 1 [file mmc1.zip › SupplementaryMaterials/115-SenSpe.png]

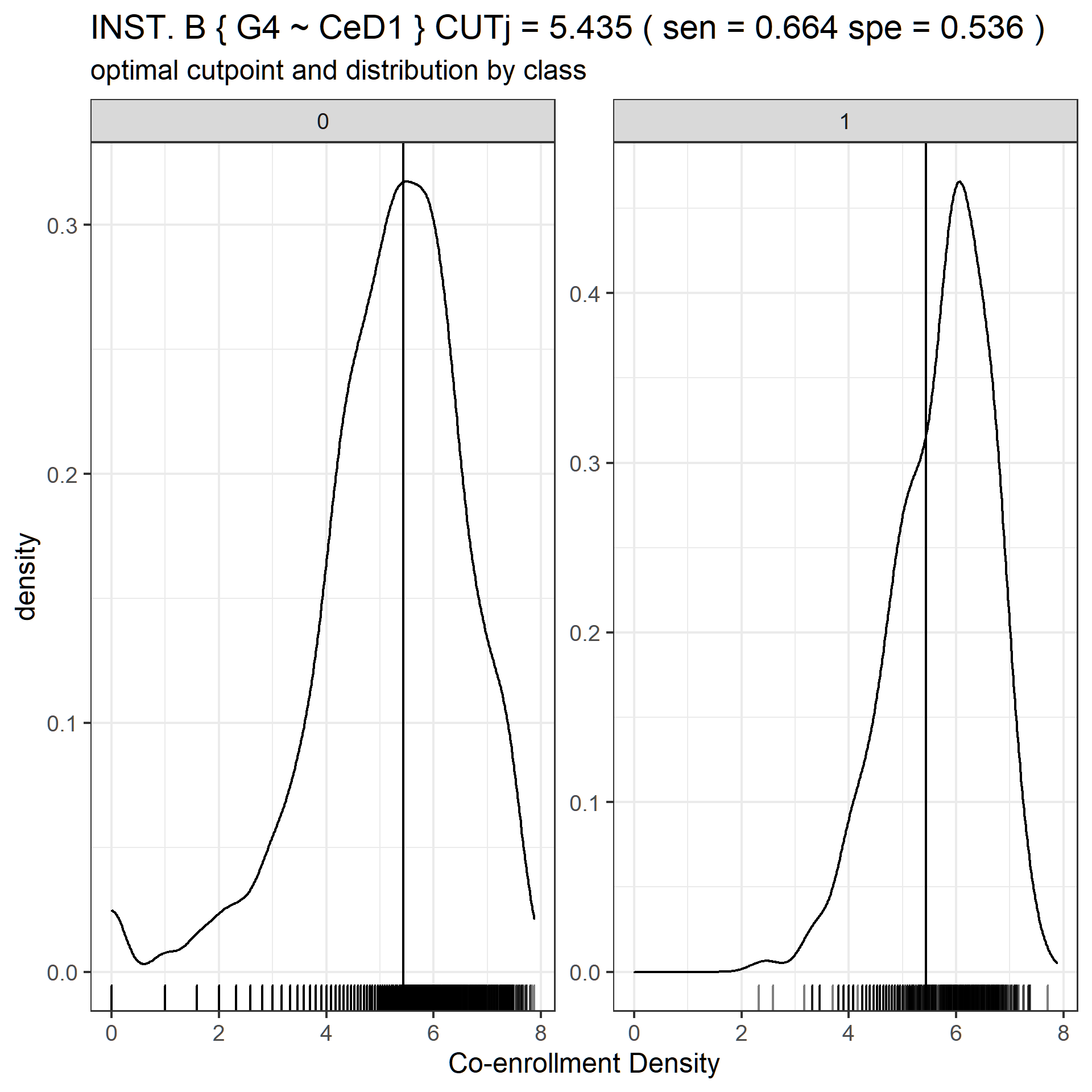

Supplement: Supplementary file 1 [file mmc1.zip › SupplementaryMaterials/116-ClassDen.png]

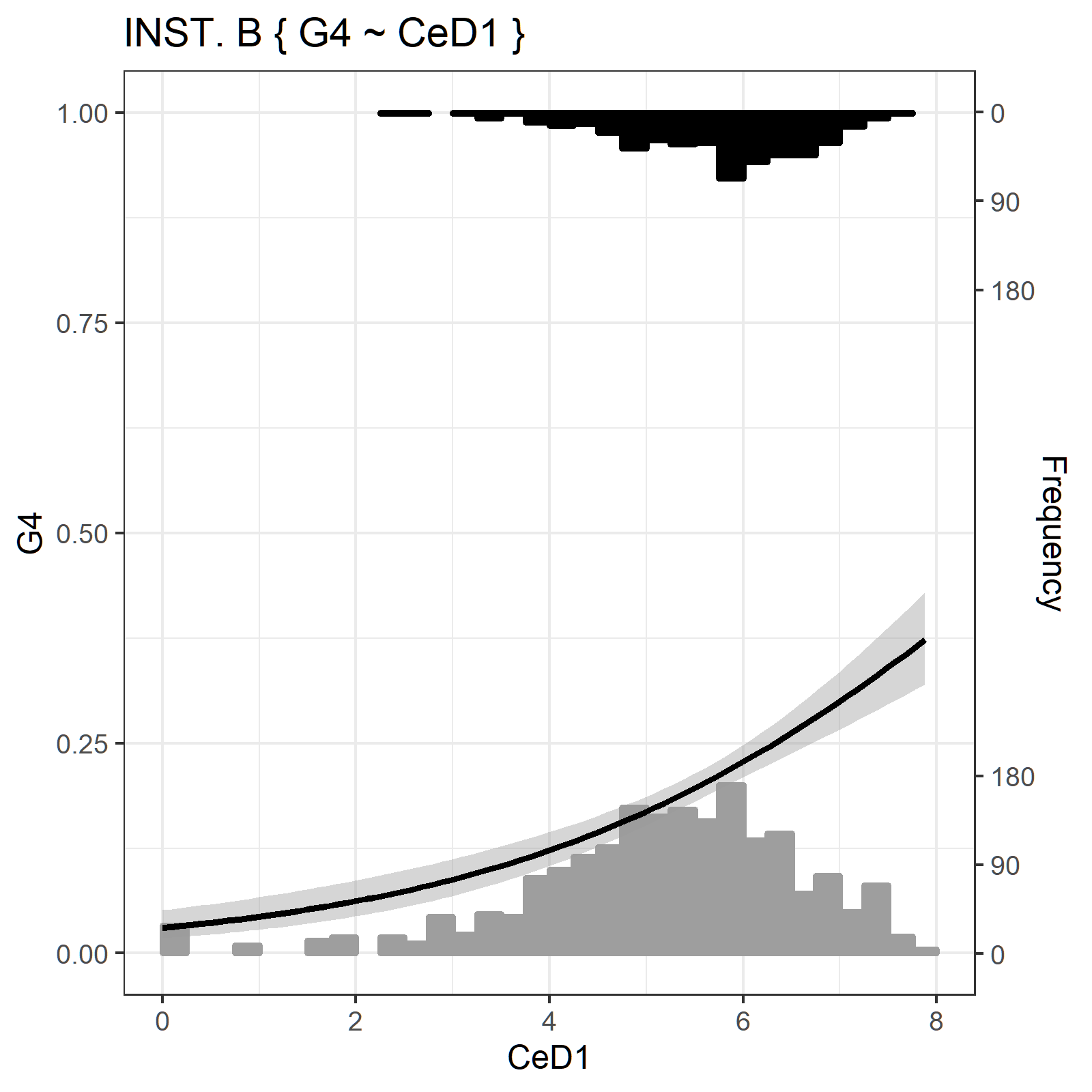

Supplement: Supplementary file 1 [file mmc1.zip › SupplementaryMaterials/116-LogitCurve.png]

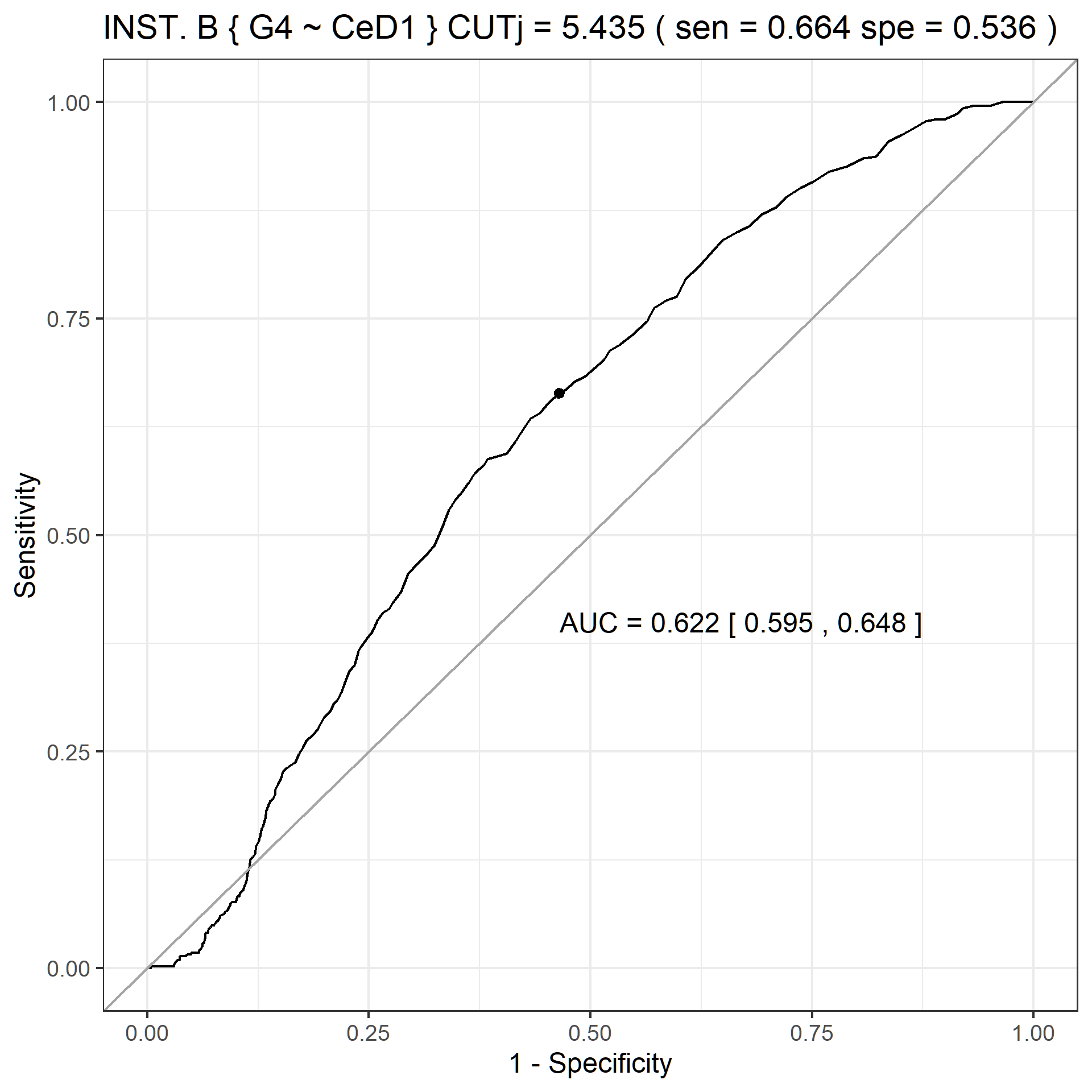

Supplement: Supplementary file 1 [file mmc1.zip › SupplementaryMaterials/116-ROCut.png]

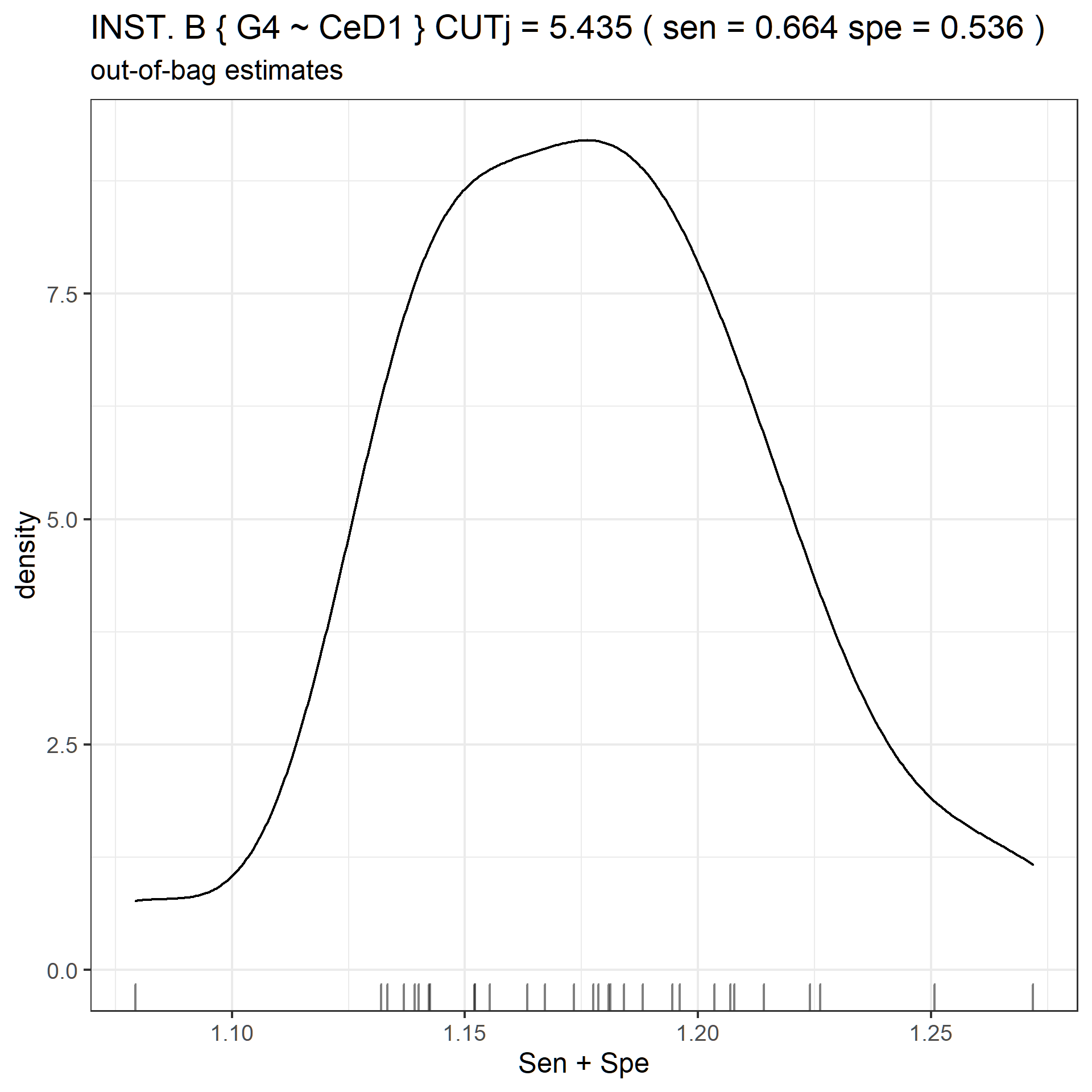

Supplement: Supplementary file 1 [file mmc1.zip › SupplementaryMaterials/116-SenSpe.png]

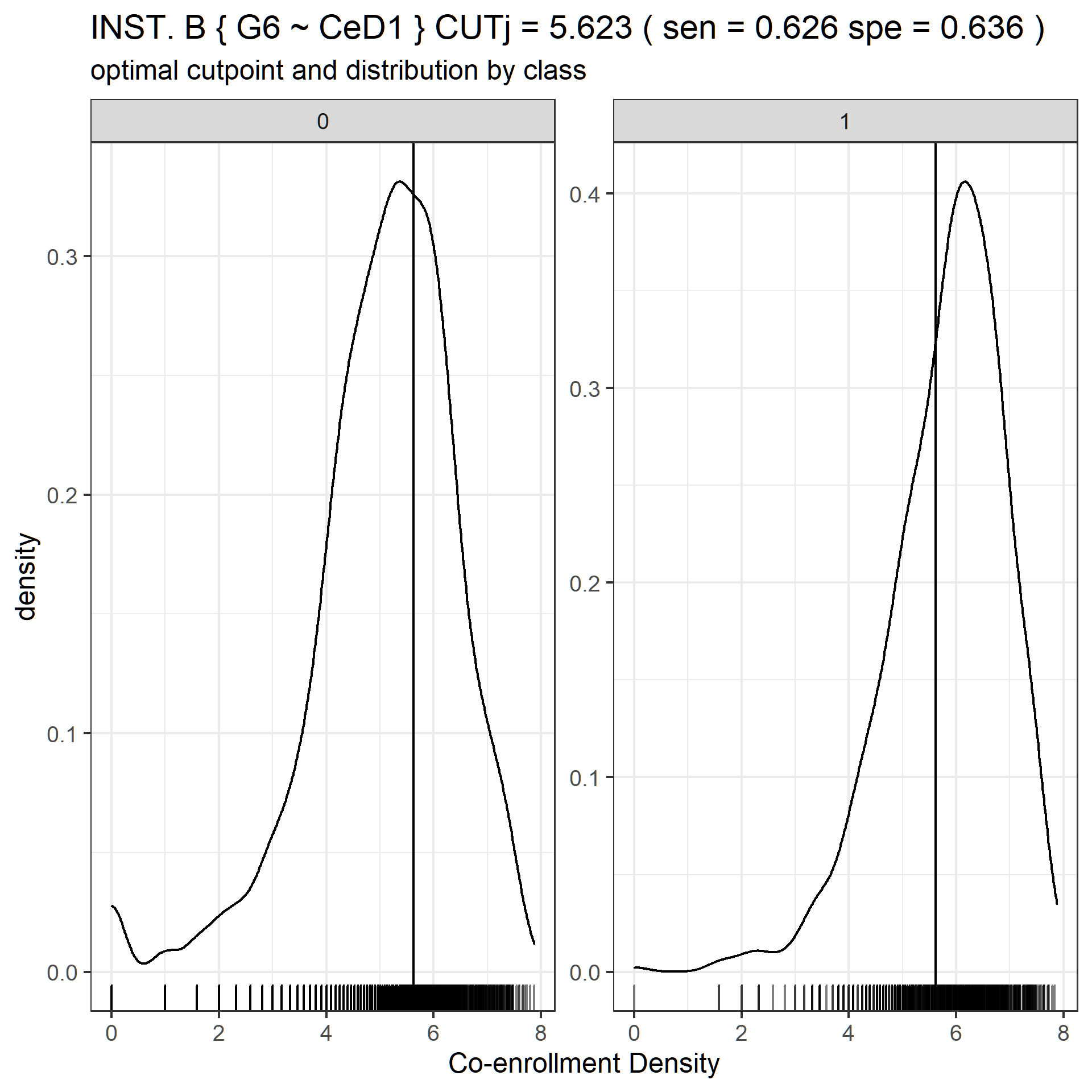

Supplement: Supplementary file 1 [file mmc1.zip › SupplementaryMaterials/117-ClassDen.png]

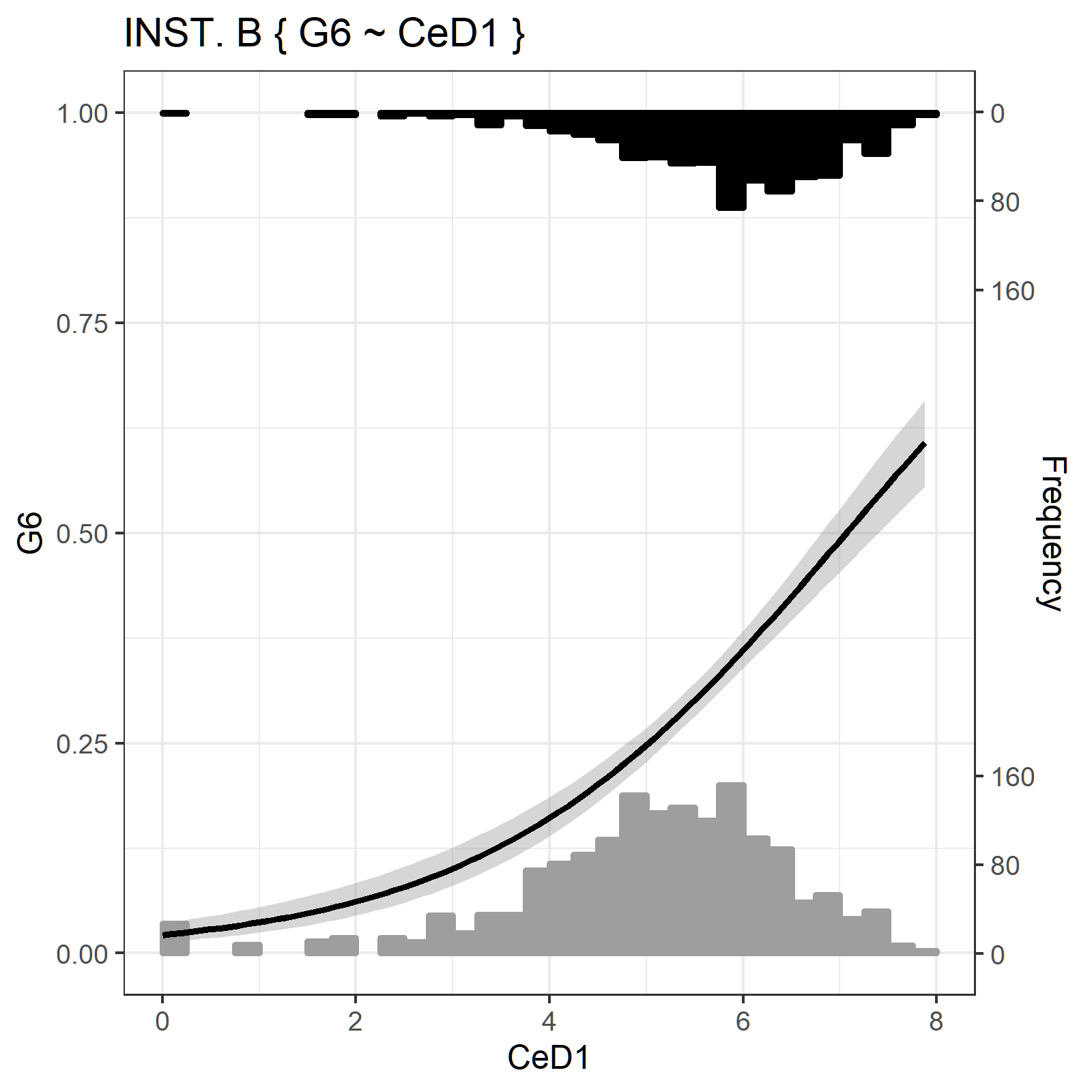

Supplement: Supplementary file 1 [file mmc1.zip › SupplementaryMaterials/117-LogitCurve.png]

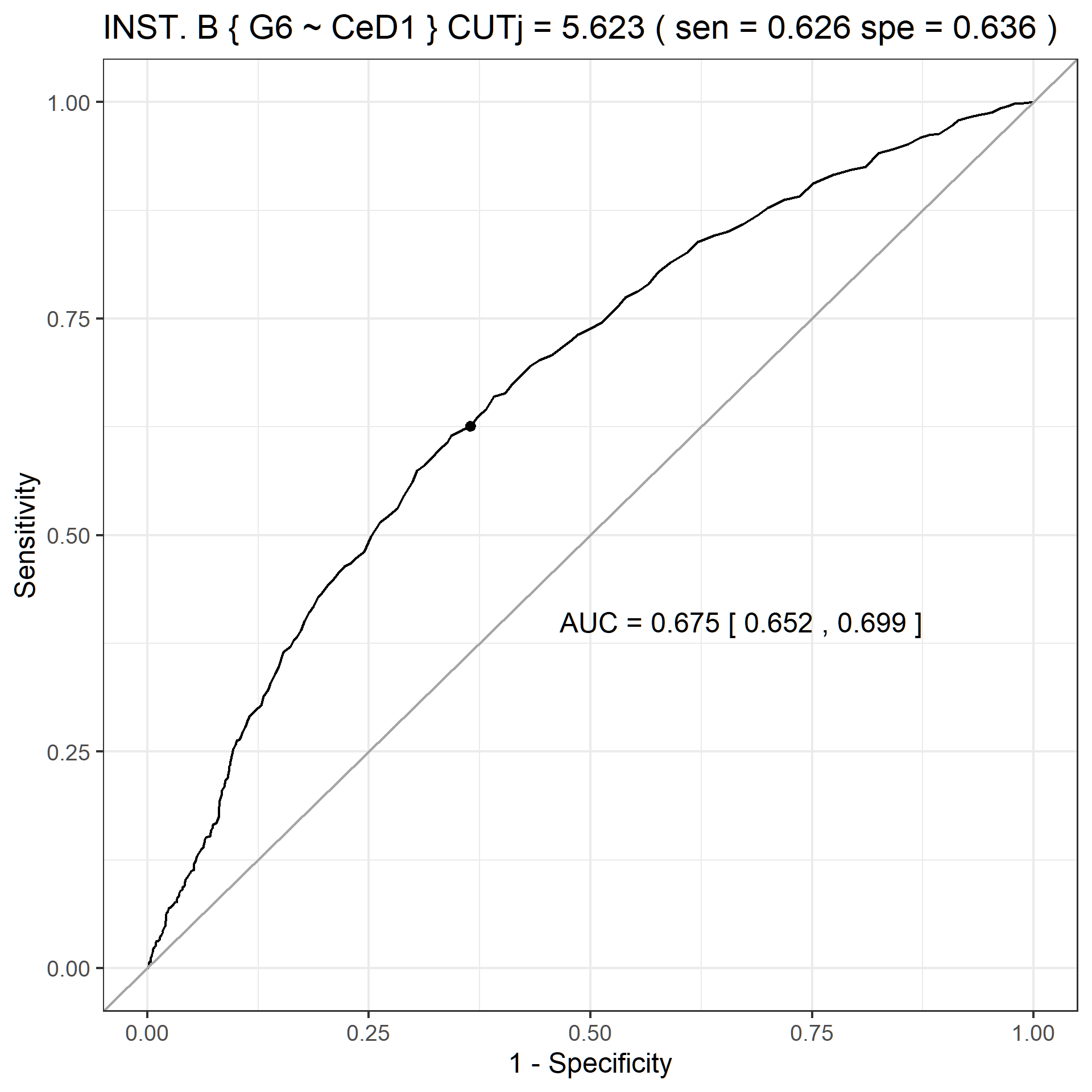

Supplement: Supplementary file 1 [file mmc1.zip › SupplementaryMaterials/117-ROCut.png]

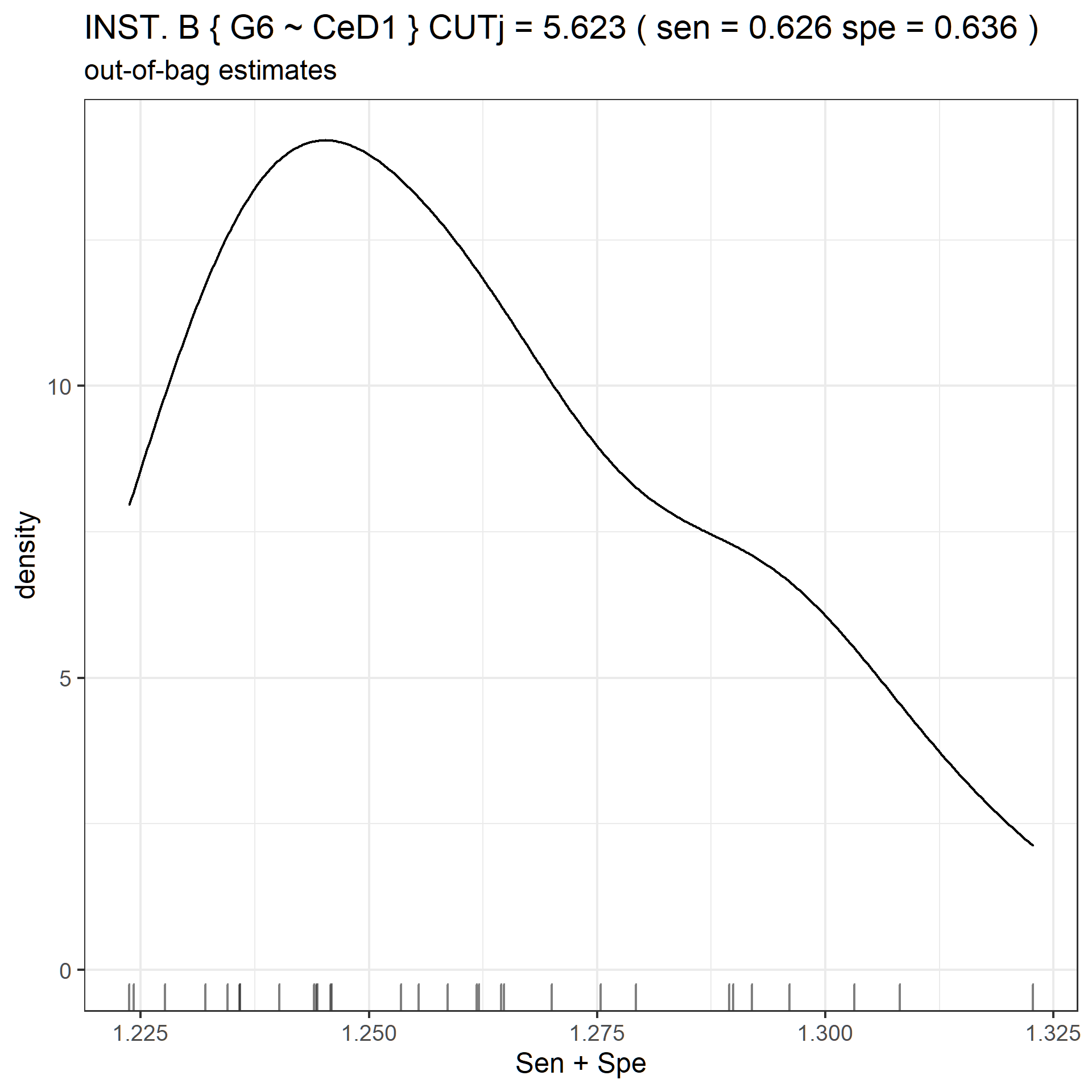

Supplement: Supplementary file 1 [file mmc1.zip › SupplementaryMaterials/117-SenSpe.png]

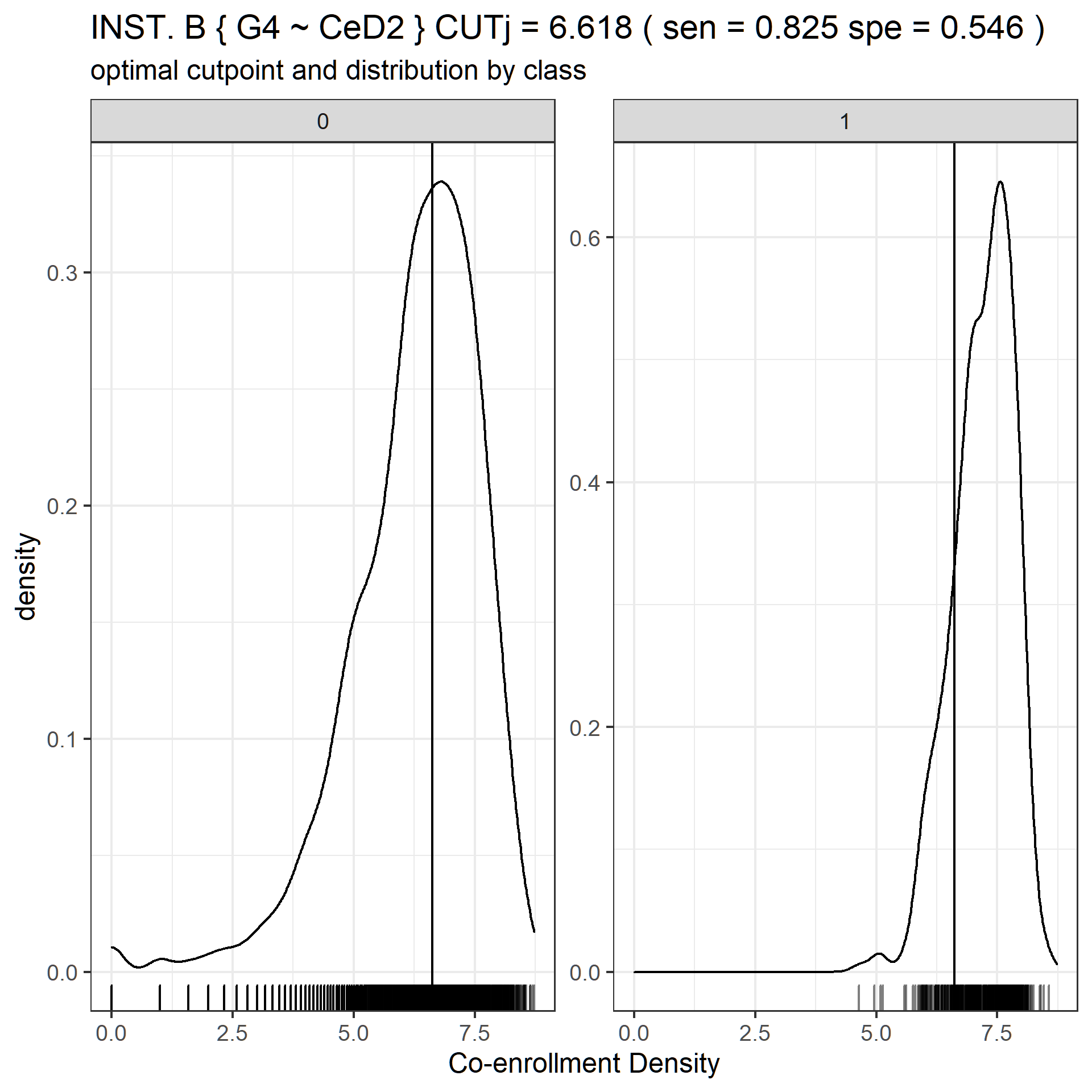

Supplement: Supplementary file 1 [file mmc1.zip › SupplementaryMaterials/126-ClassDen.png]

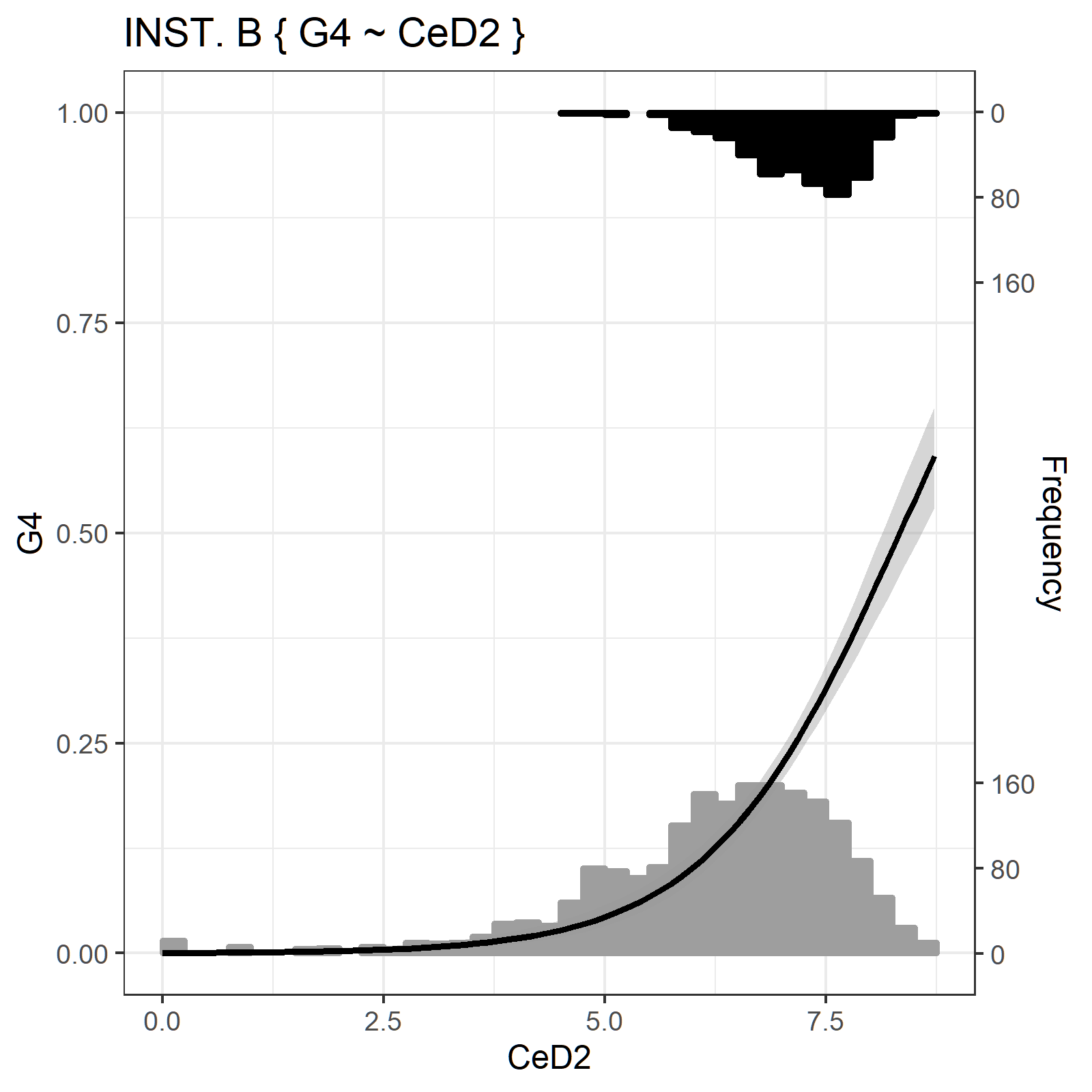

Supplement: Supplementary file 1 [file mmc1.zip › SupplementaryMaterials/126-LogitCurve.png]

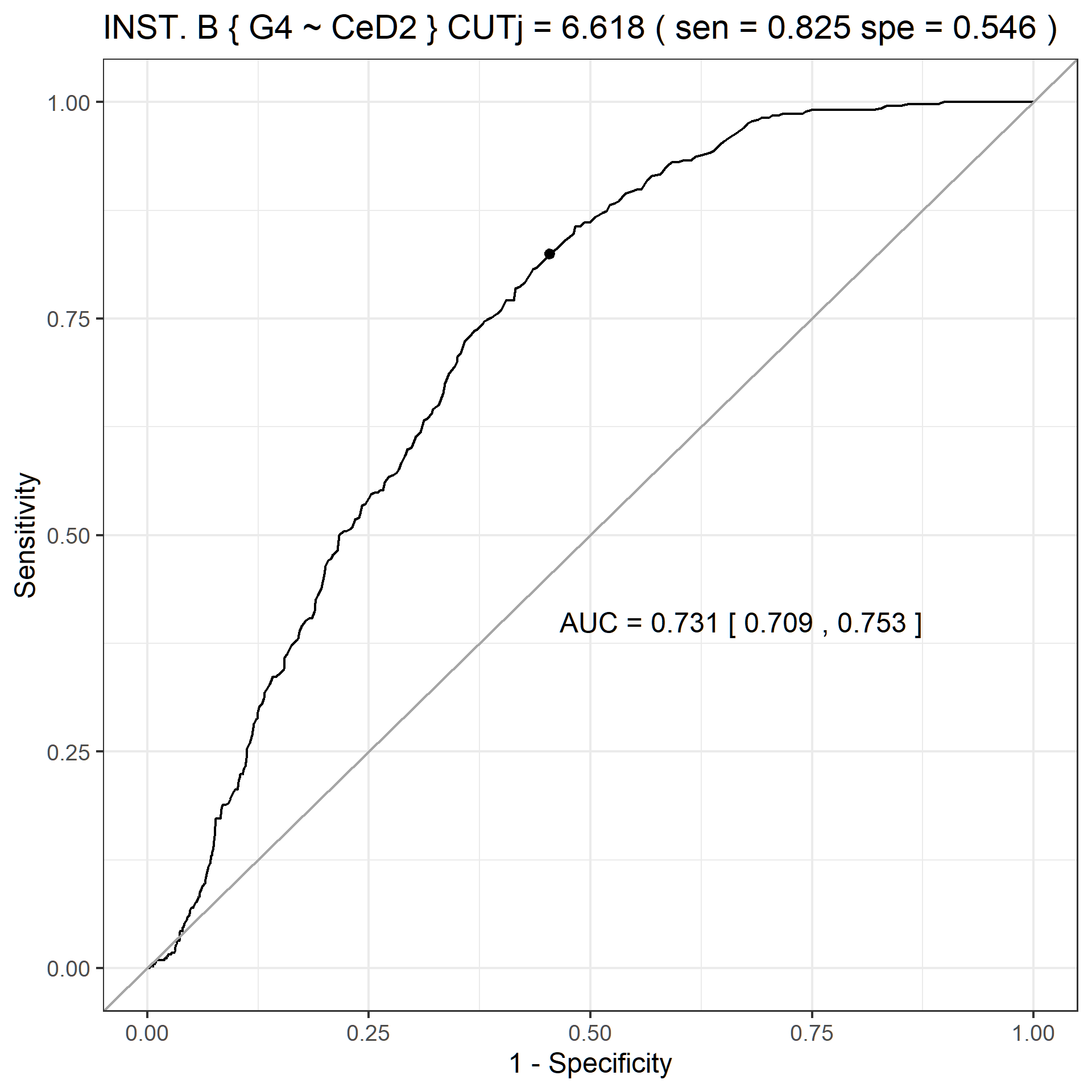

Supplement: Supplementary file 1 [file mmc1.zip › SupplementaryMaterials/126-ROCut.png]

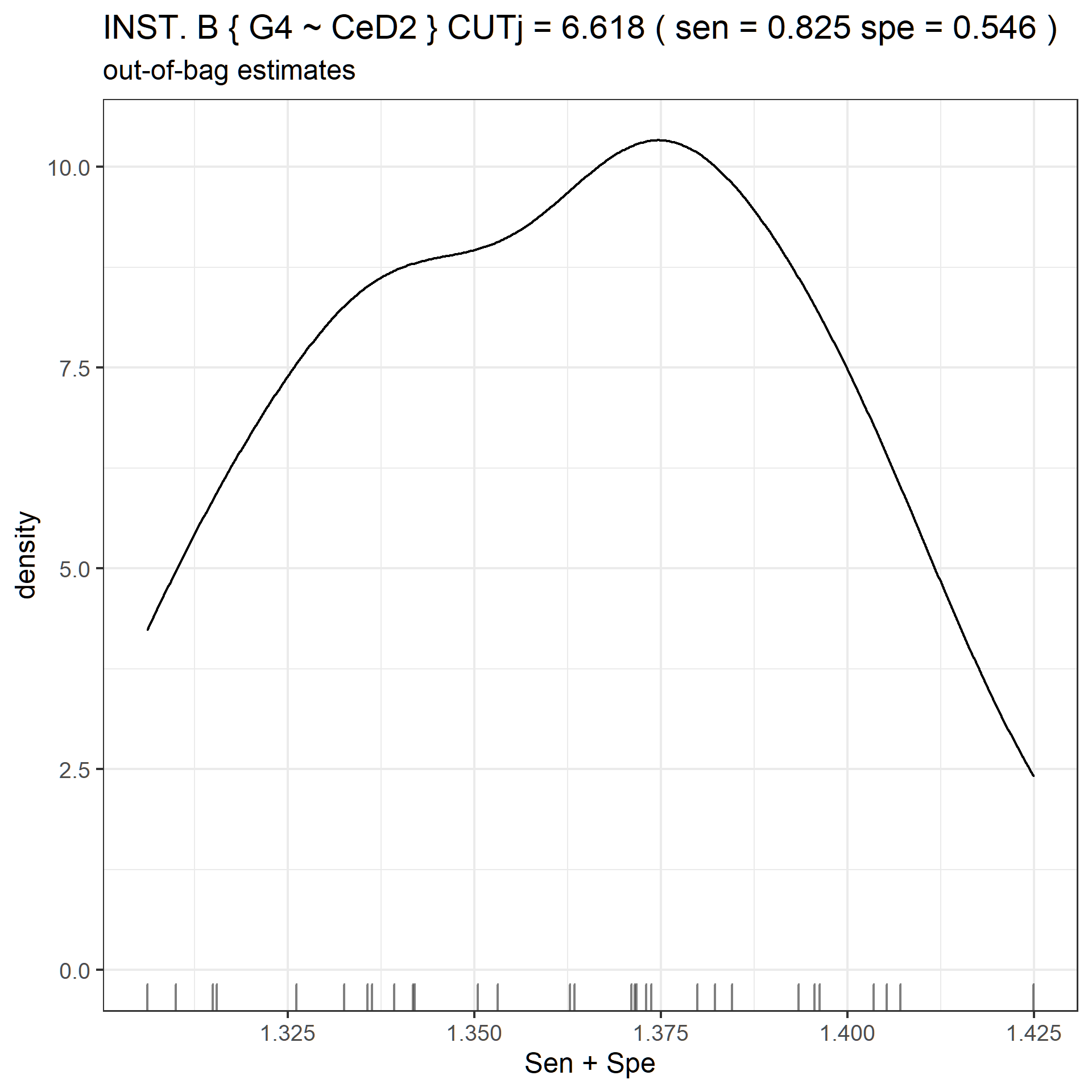

Supplement: Supplementary file 1 [file mmc1.zip › SupplementaryMaterials/126-SenSpe.png]

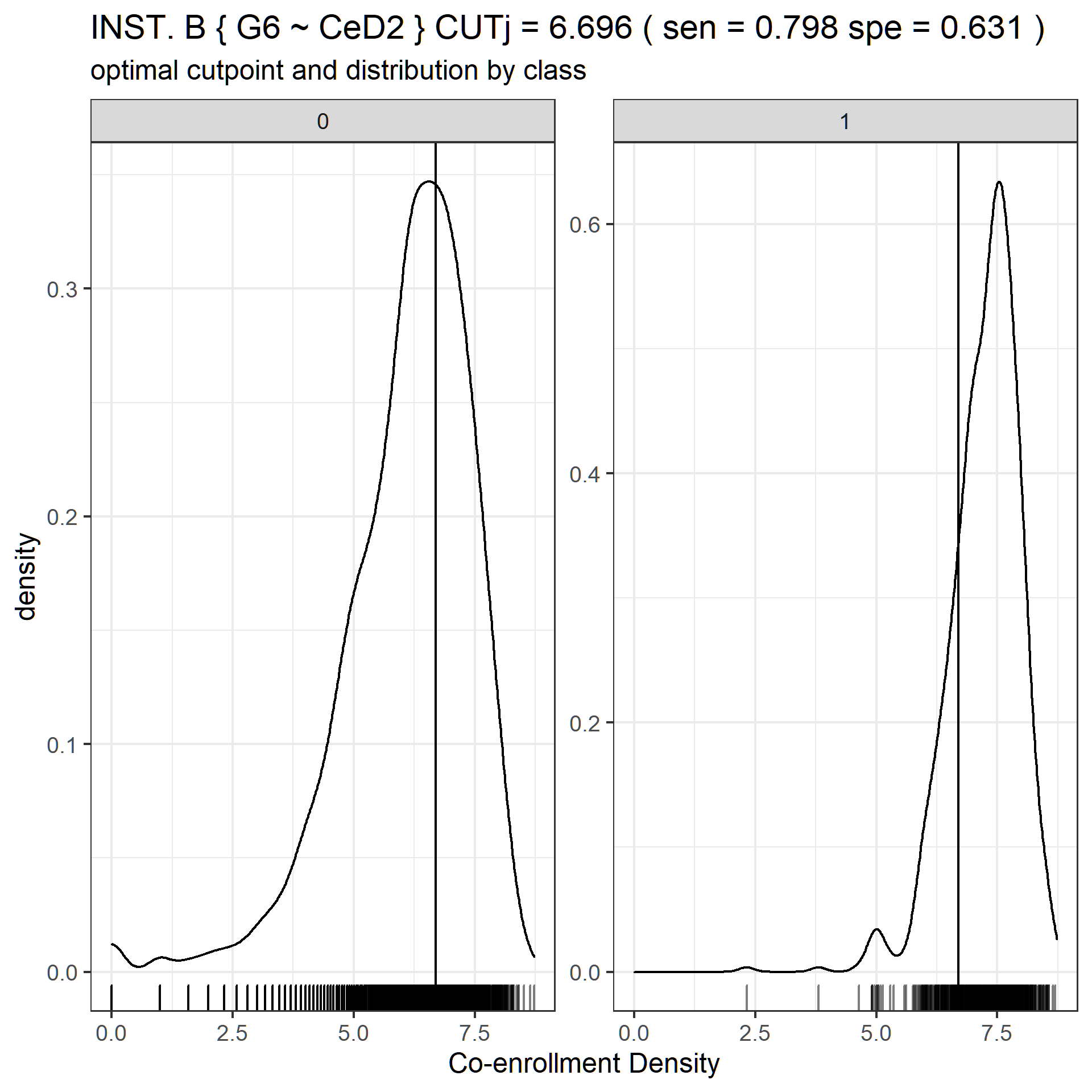

Supplement: Supplementary file 1 [file mmc1.zip › SupplementaryMaterials/127-ClassDen.png]

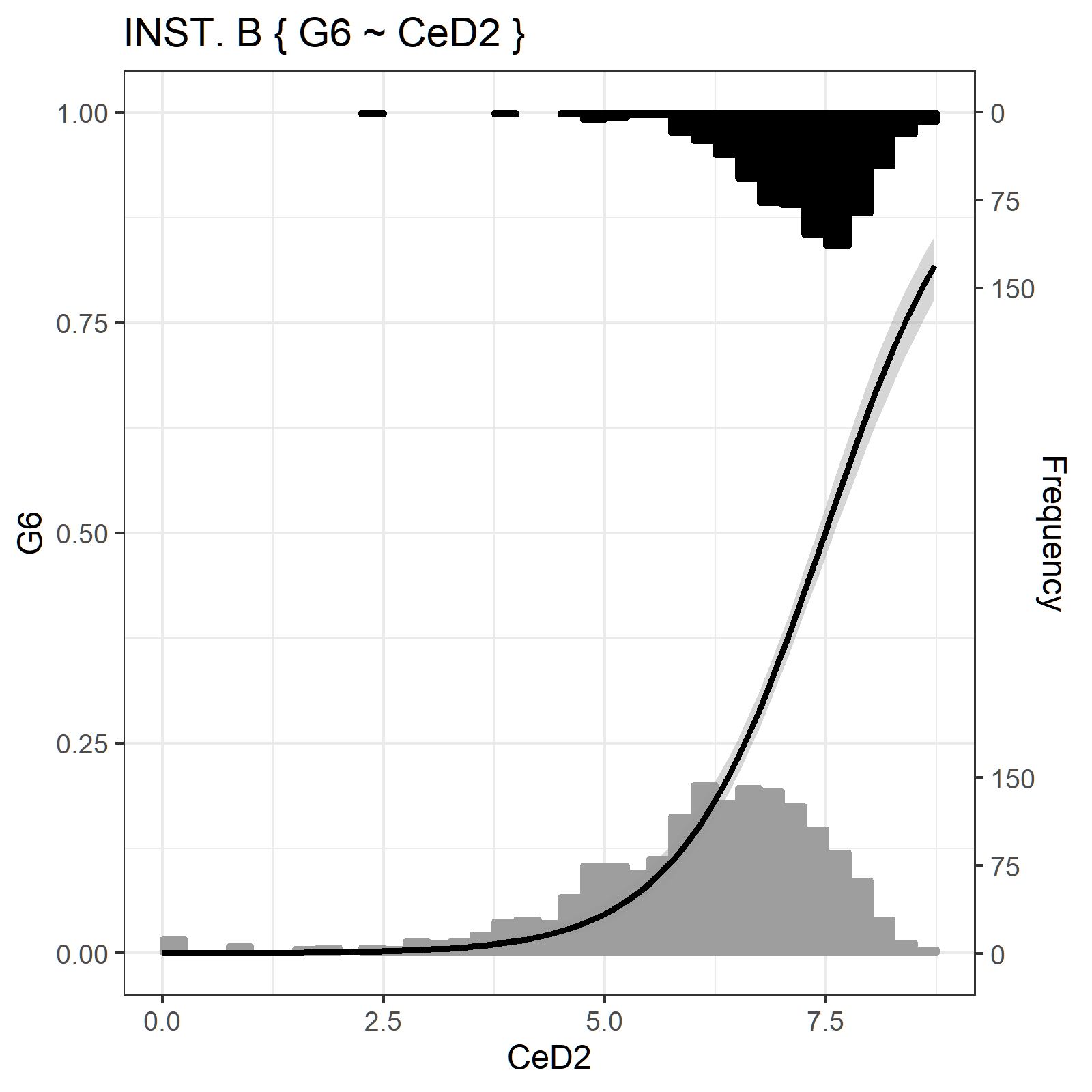

Supplement: Supplementary file 1 [file mmc1.zip › SupplementaryMaterials/127-LogitCurve.png]

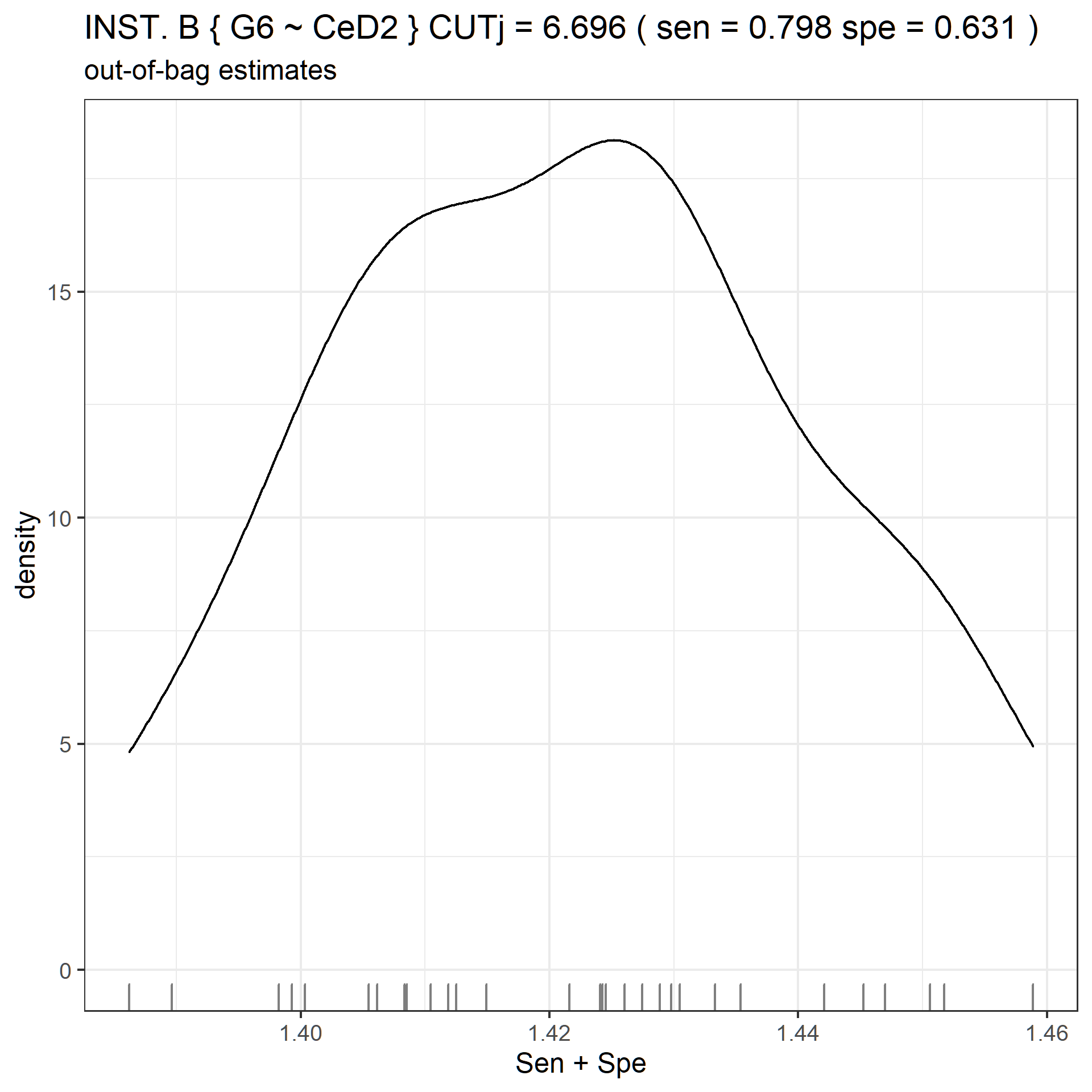

Supplement: Supplementary file 1 [file mmc1.zip › SupplementaryMaterials/127-SenSpe.png]

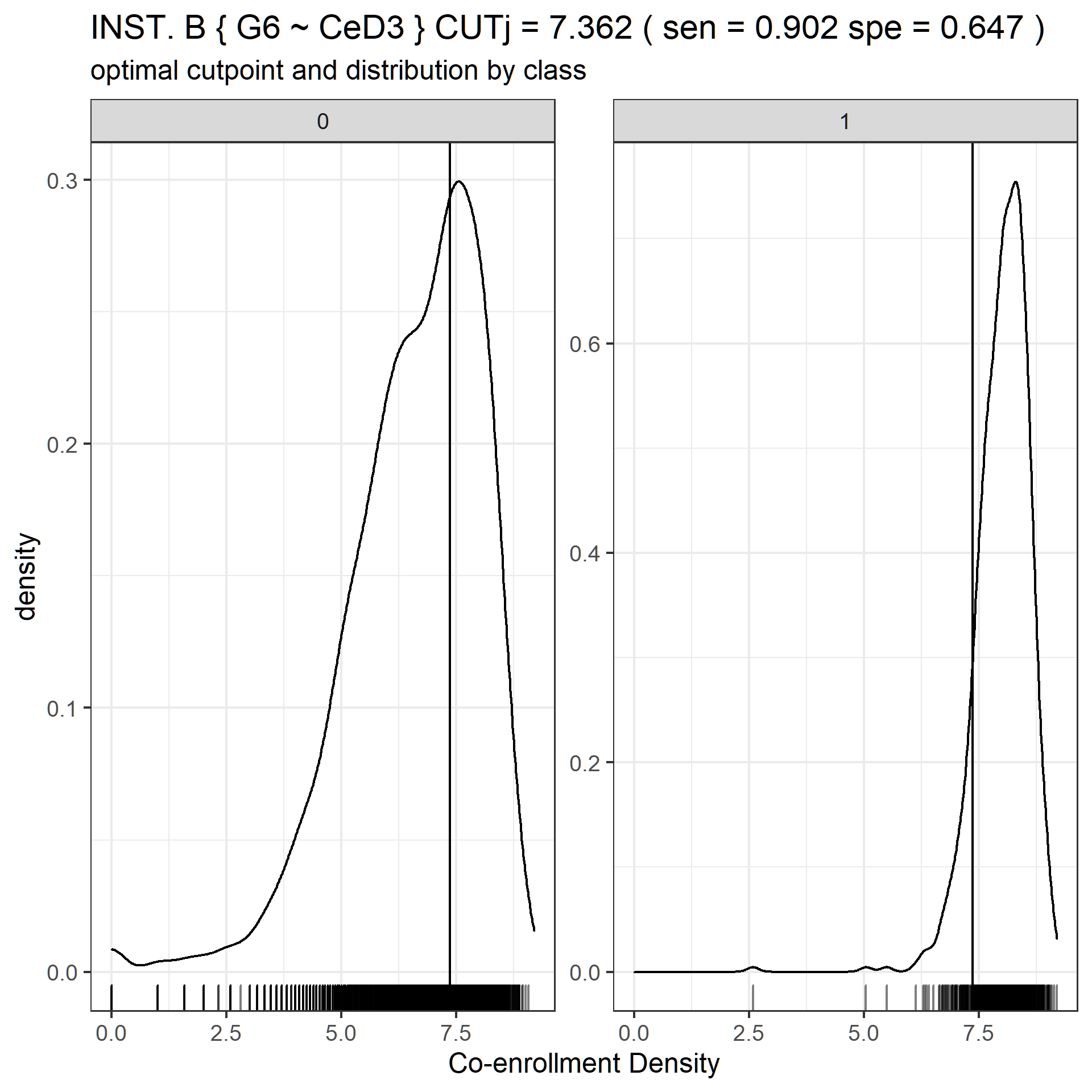

Supplement: Supplementary file 1 [file mmc1.zip › SupplementaryMaterials/137-ClassDen.png]

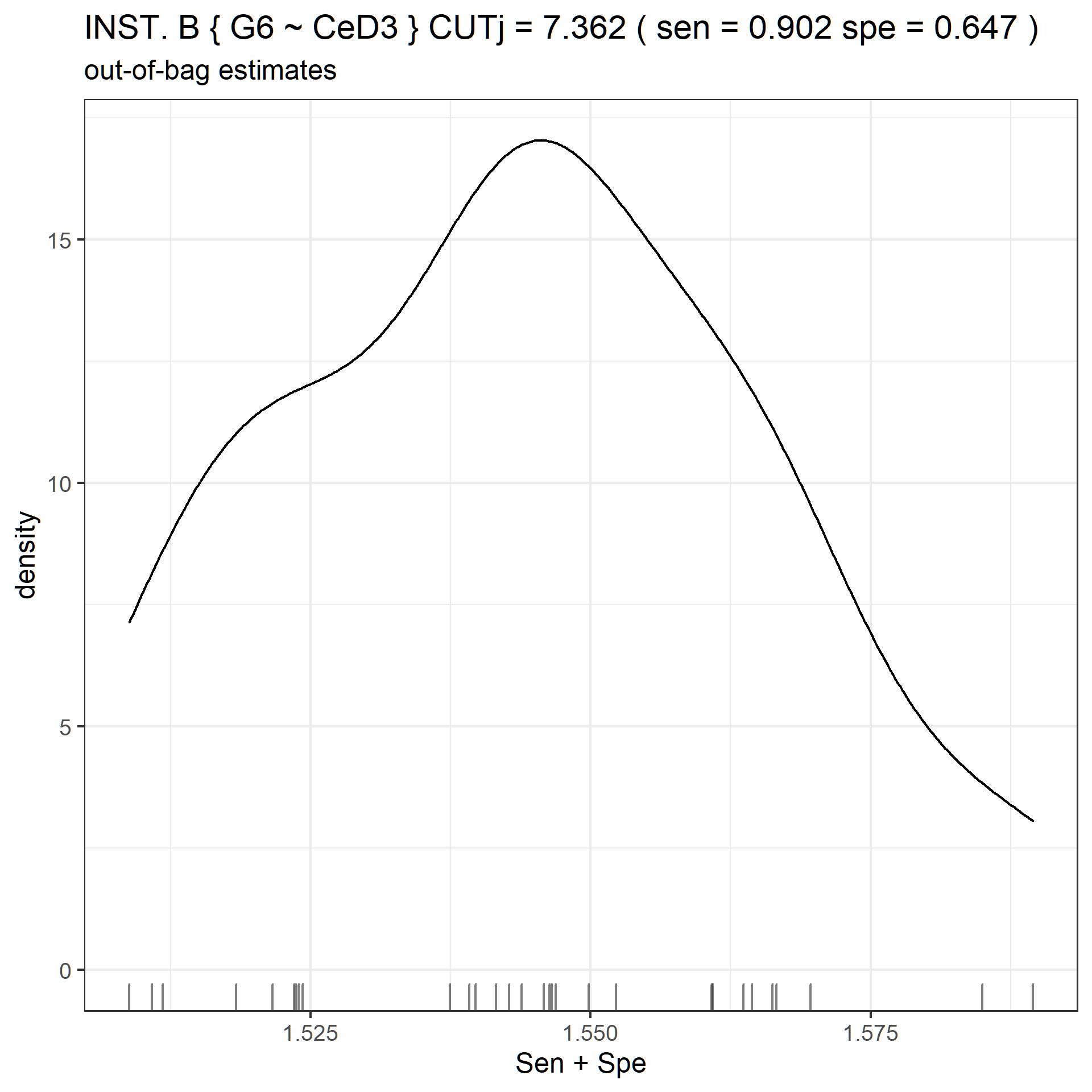

Supplement: Supplementary file 1 [file mmc1.zip › SupplementaryMaterials/137-SenSpe.png]

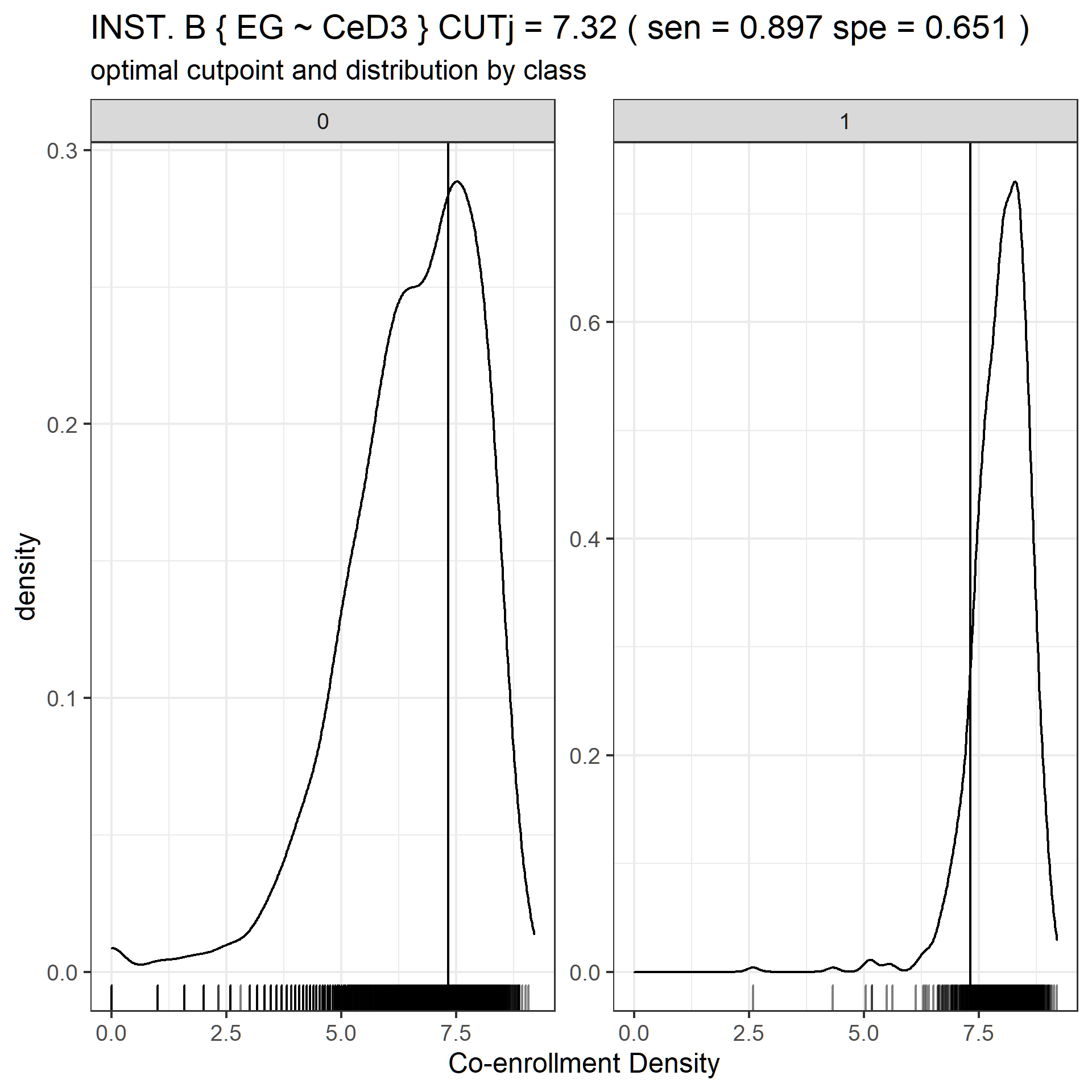

Supplement: Supplementary file 1 [file mmc1.zip › SupplementaryMaterials/138-ClassDen.png]

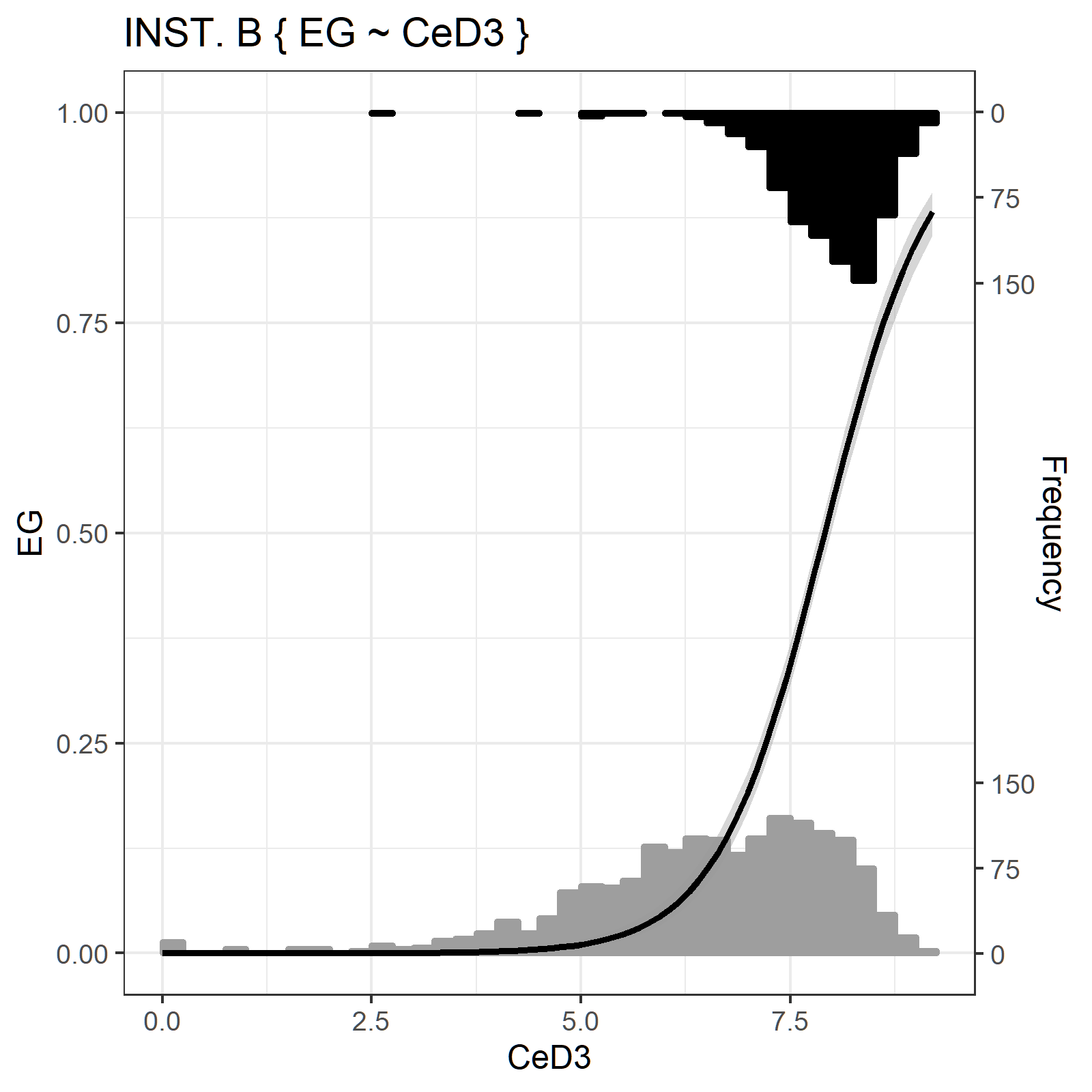

Supplement: Supplementary file 1 [file mmc1.zip › SupplementaryMaterials/138-LogitCurve.png]

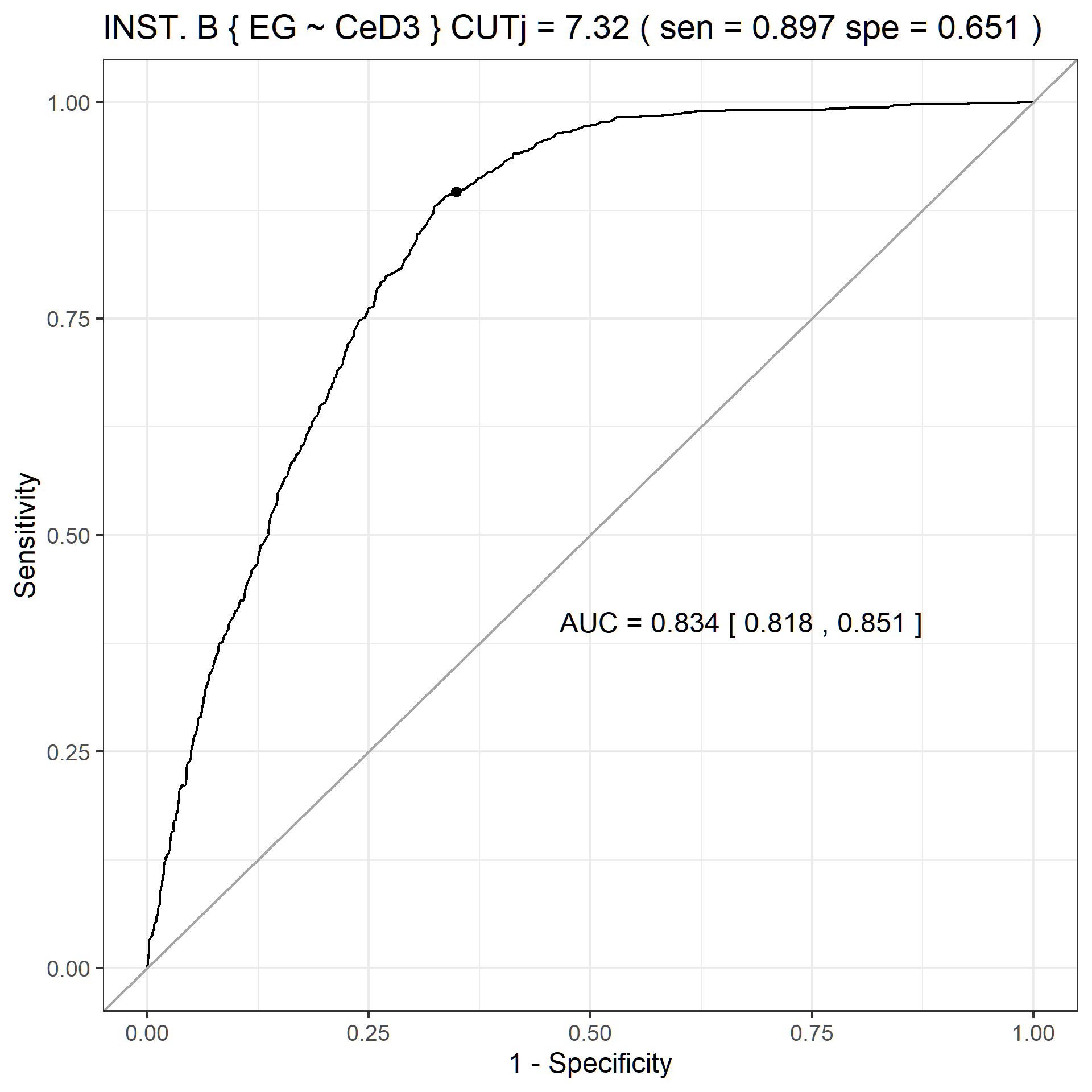

Supplement: Supplementary file 1 [file mmc1.zip › SupplementaryMaterials/138-ROCut.png]

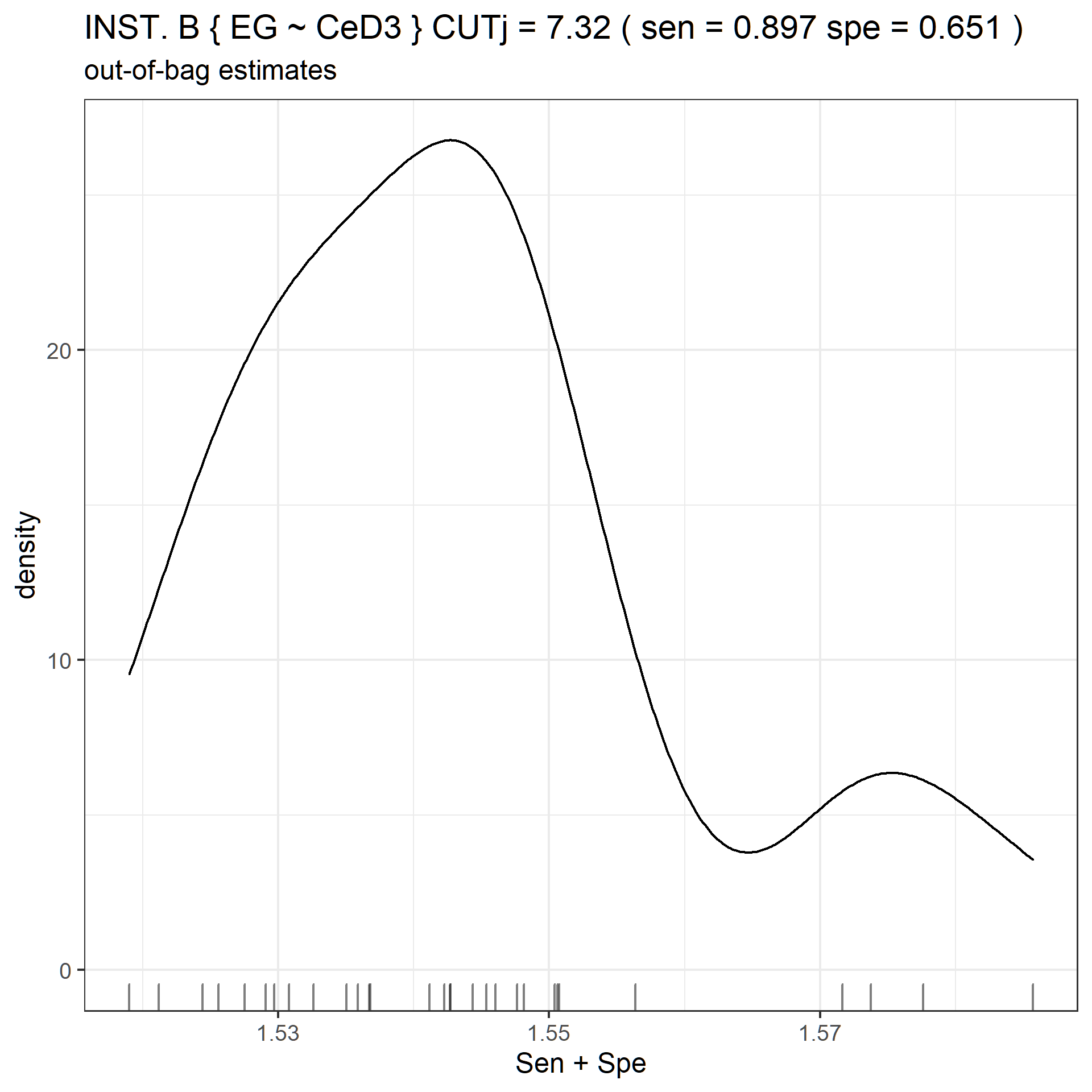

Supplement: Supplementary file 1 [file mmc1.zip › SupplementaryMaterials/138-SenSpe.png]

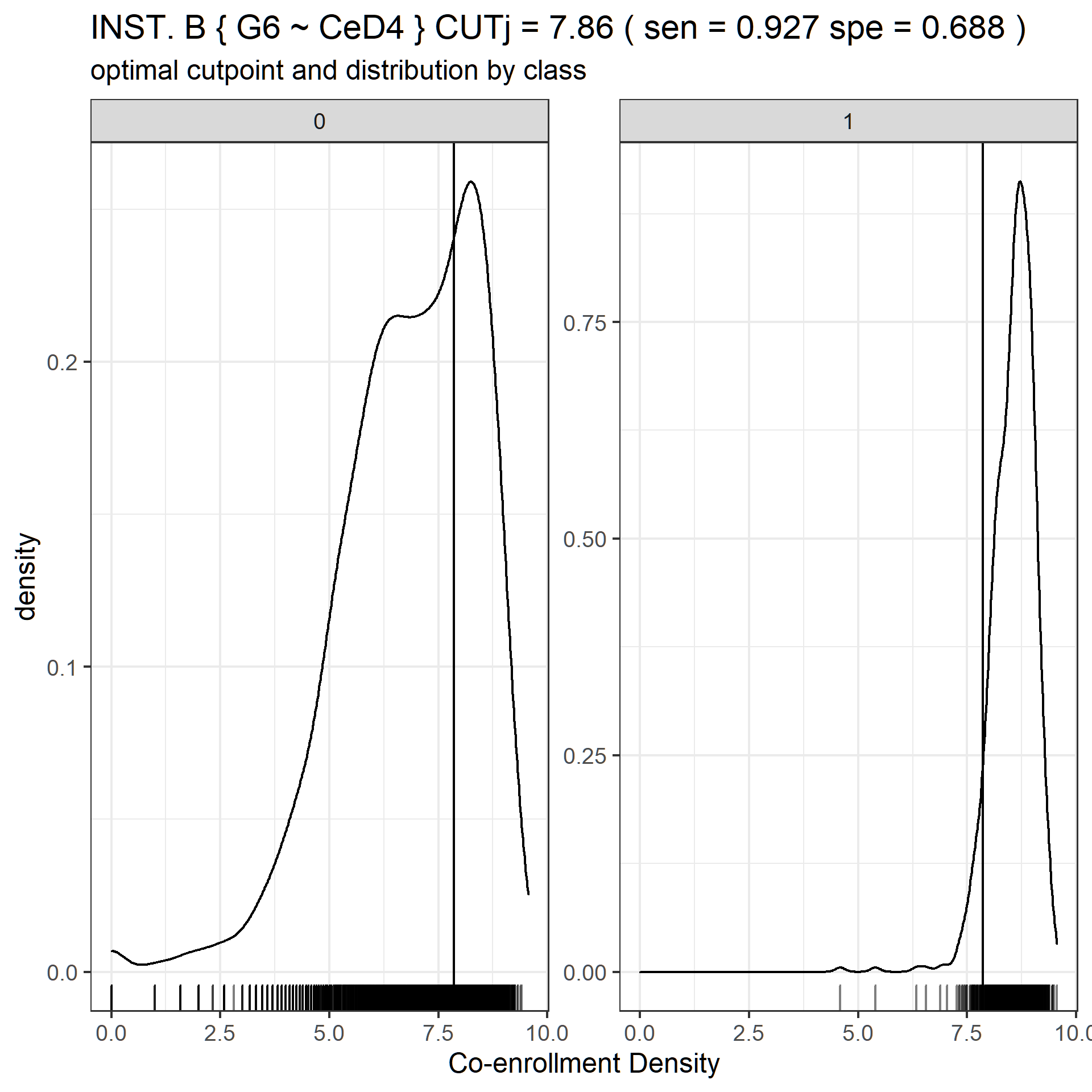

Supplement: Supplementary file 1 [file mmc1.zip › SupplementaryMaterials/147-ClassDen.png]

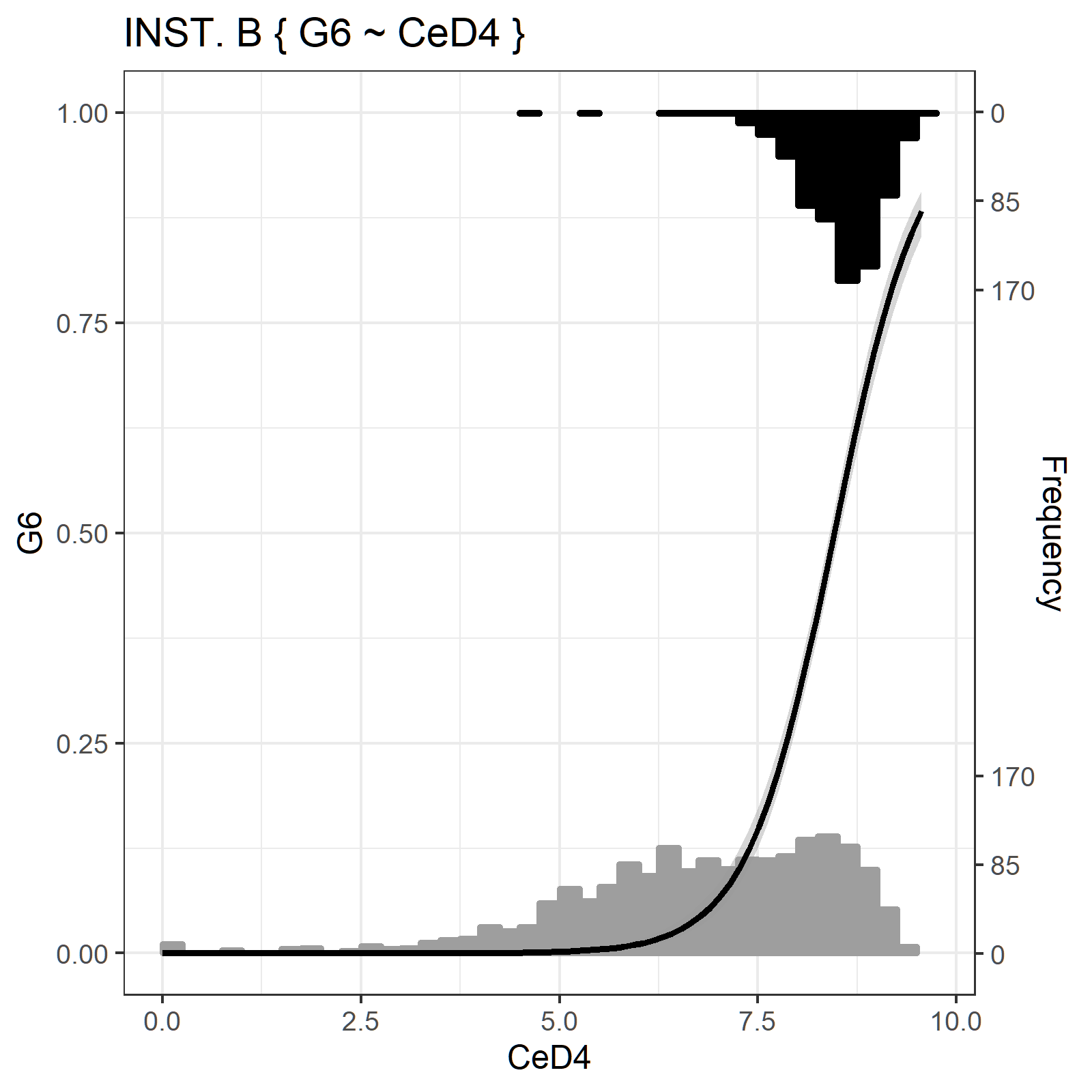

Supplement: Supplementary file 1 [file mmc1.zip › SupplementaryMaterials/147-LogitCurve.png]

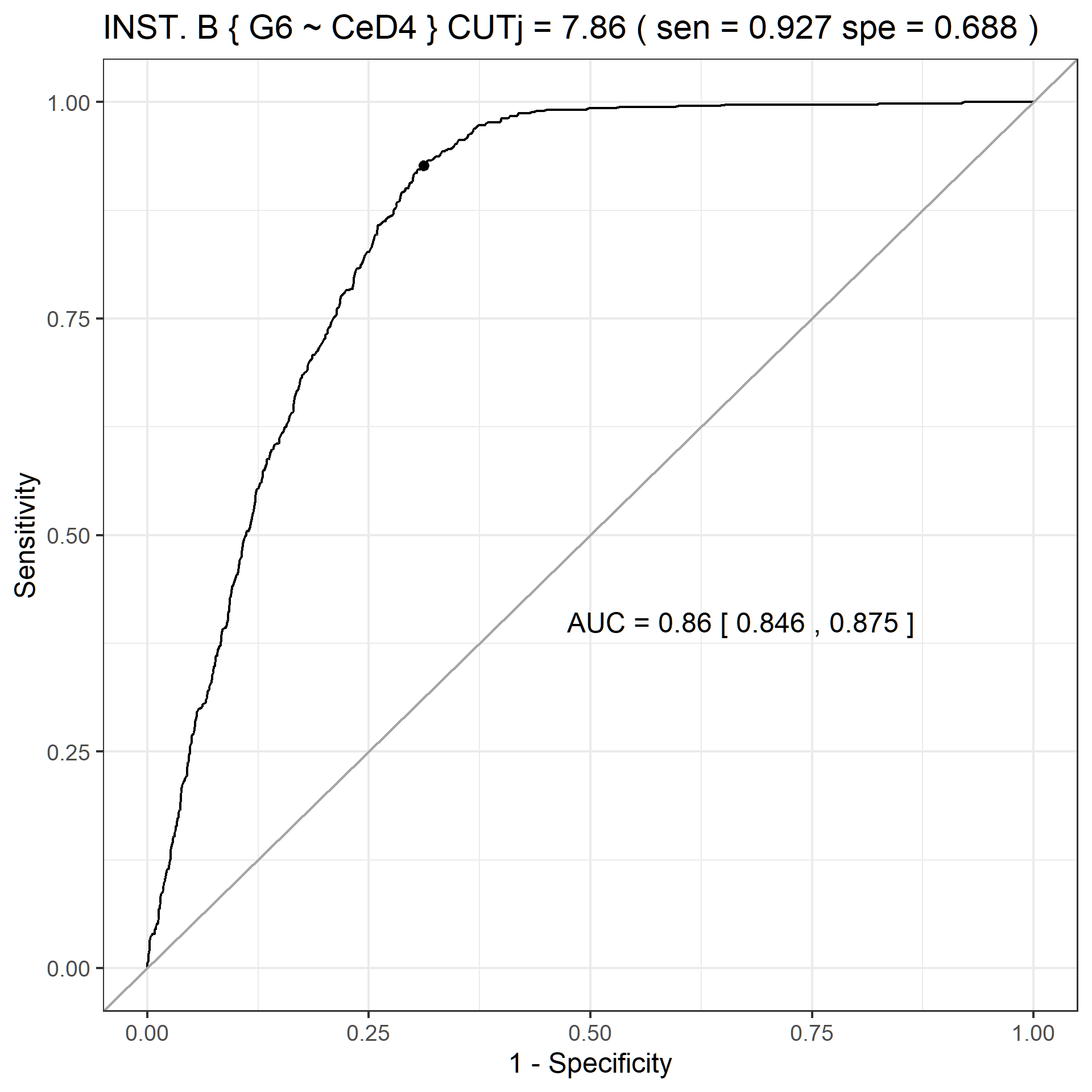

Supplement: Supplementary file 1 [file mmc1.zip › SupplementaryMaterials/147-ROCut.png]

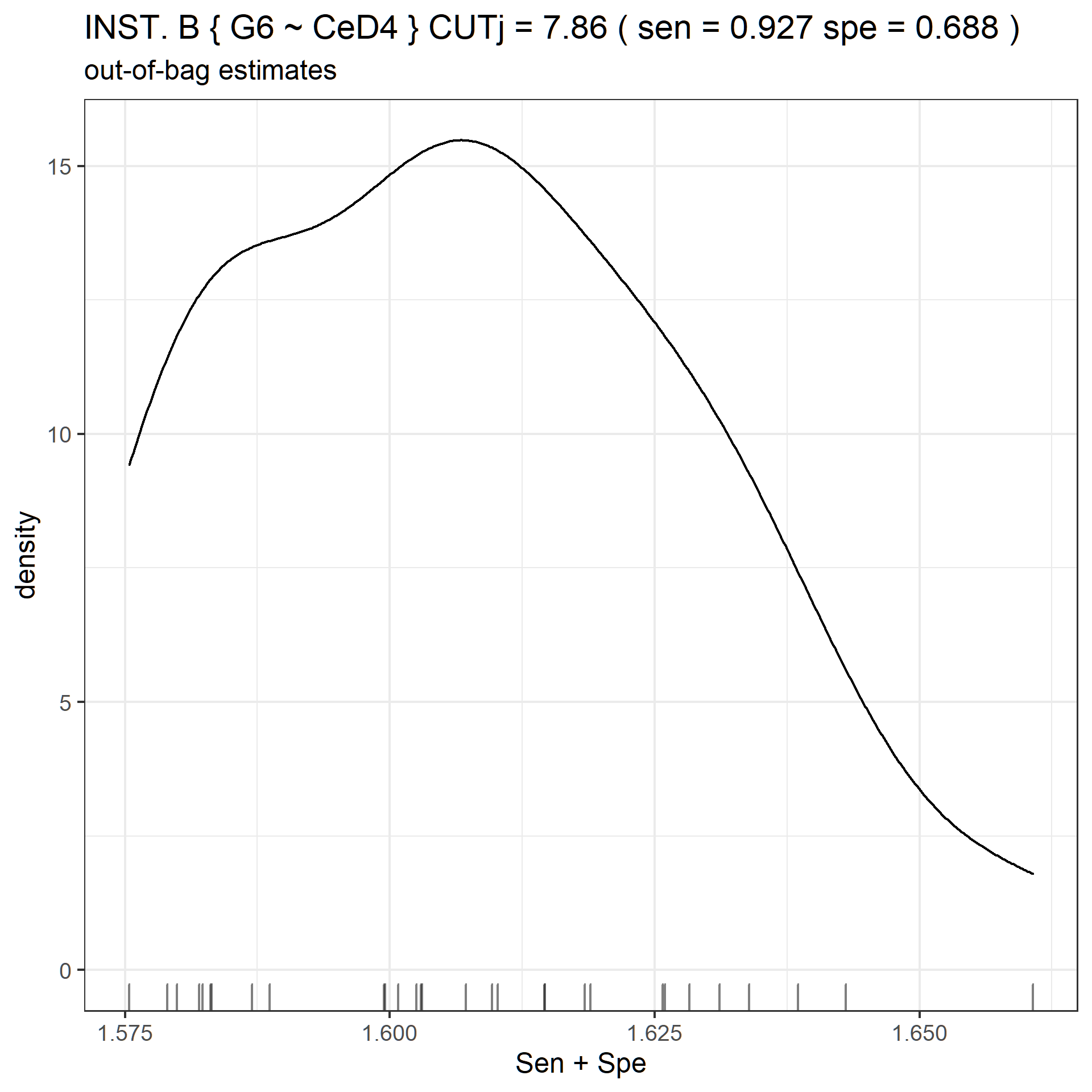

Supplement: Supplementary file 1 [file mmc1.zip › SupplementaryMaterials/147-SenSpe.png]

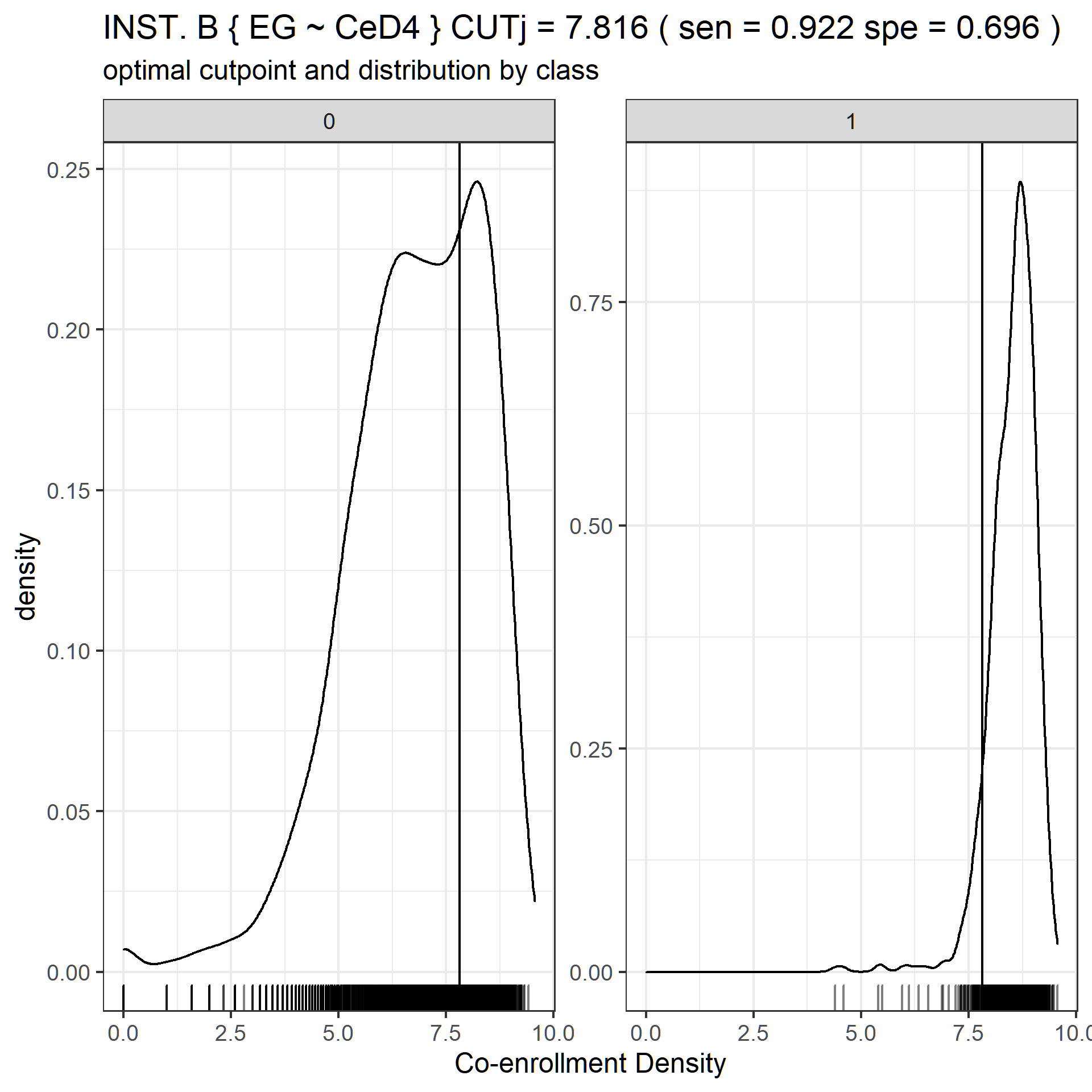

Supplement: Supplementary file 1 [file mmc1.zip › SupplementaryMaterials/148-ClassDen.png]

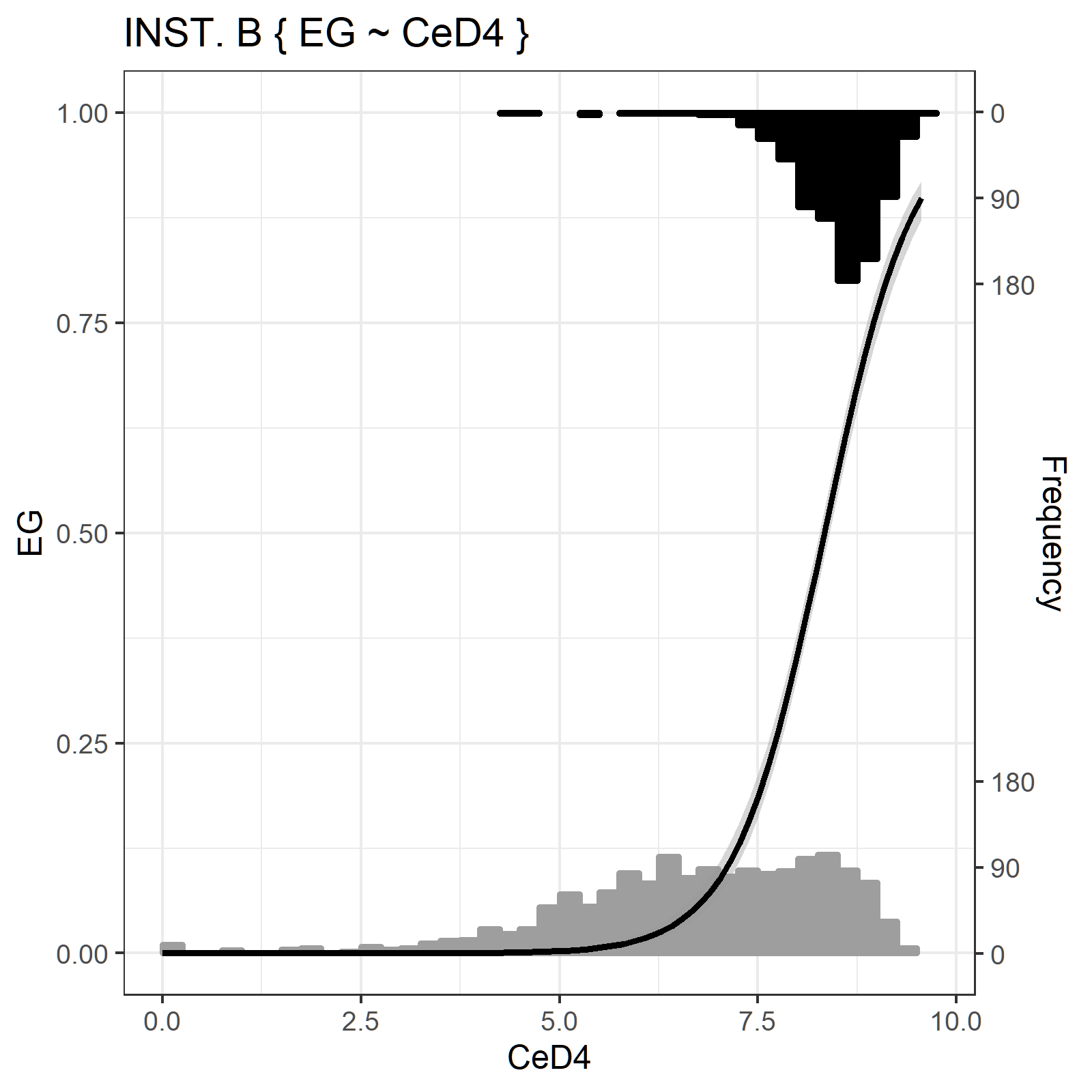

Supplement: Supplementary file 1 [file mmc1.zip › SupplementaryMaterials/148-LogitCurve.png]

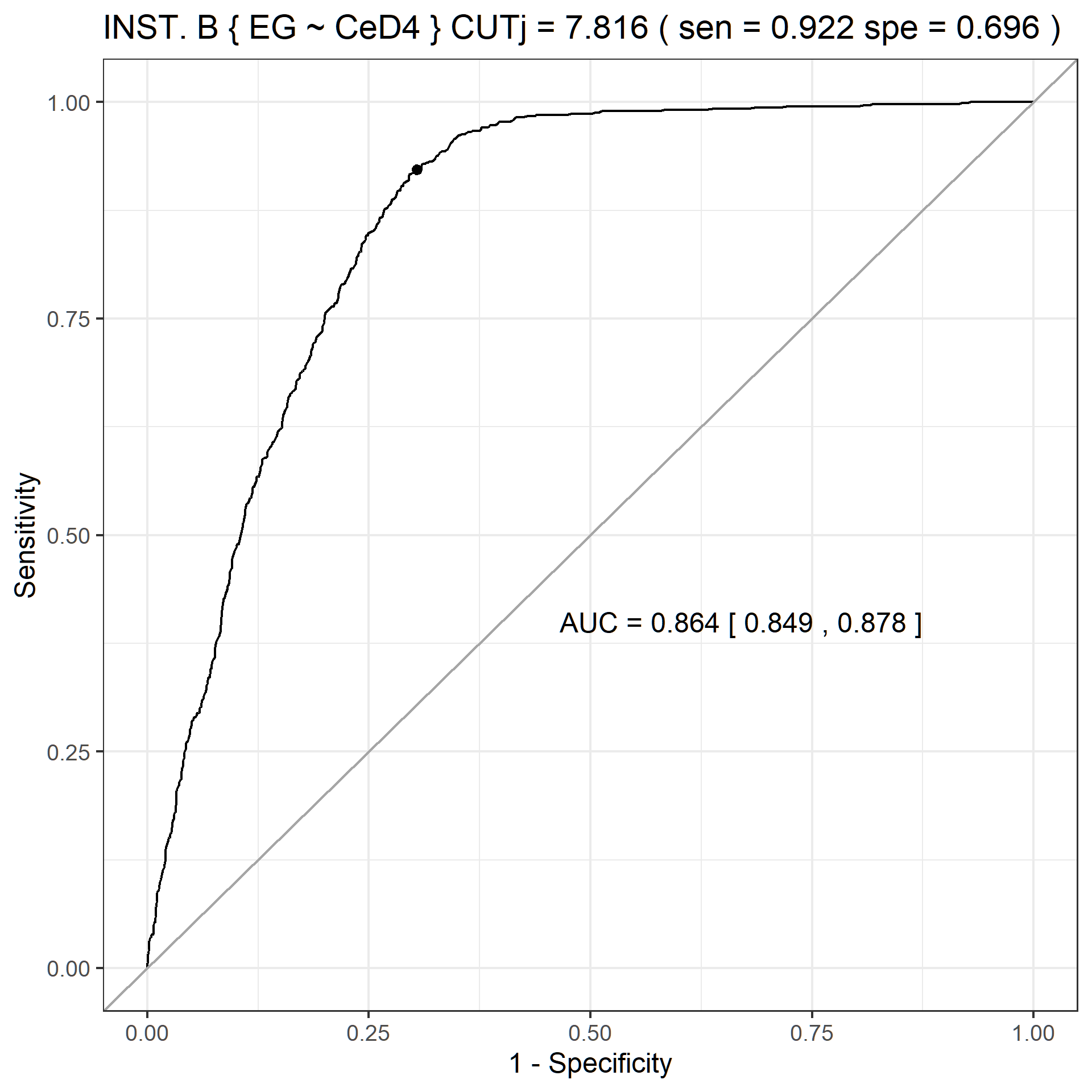

Supplement: Supplementary file 1 [file mmc1.zip › SupplementaryMaterials/148-ROCut.png]

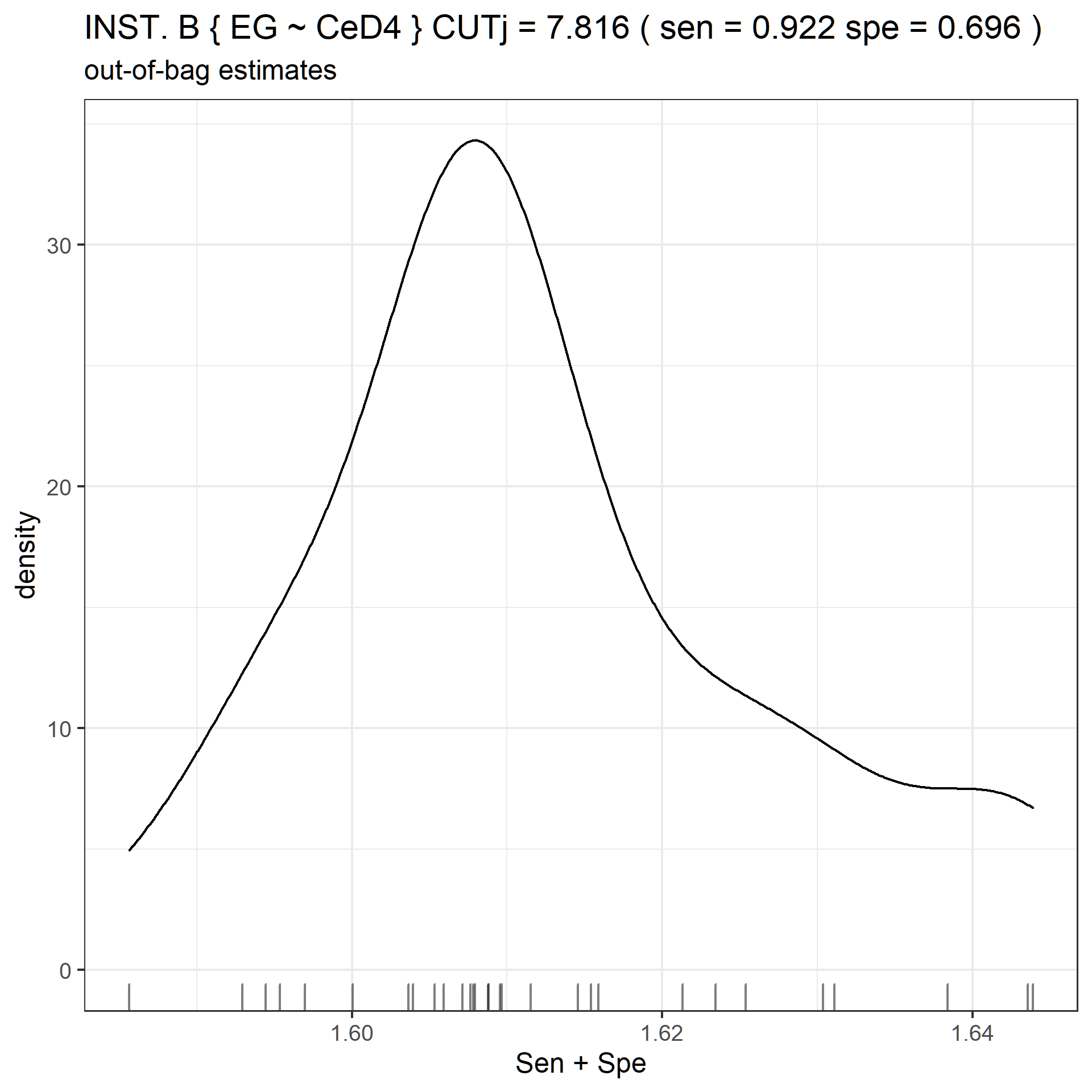

Supplement: Supplementary file 1 [file mmc1.zip › SupplementaryMaterials/148-SenSpe.png]

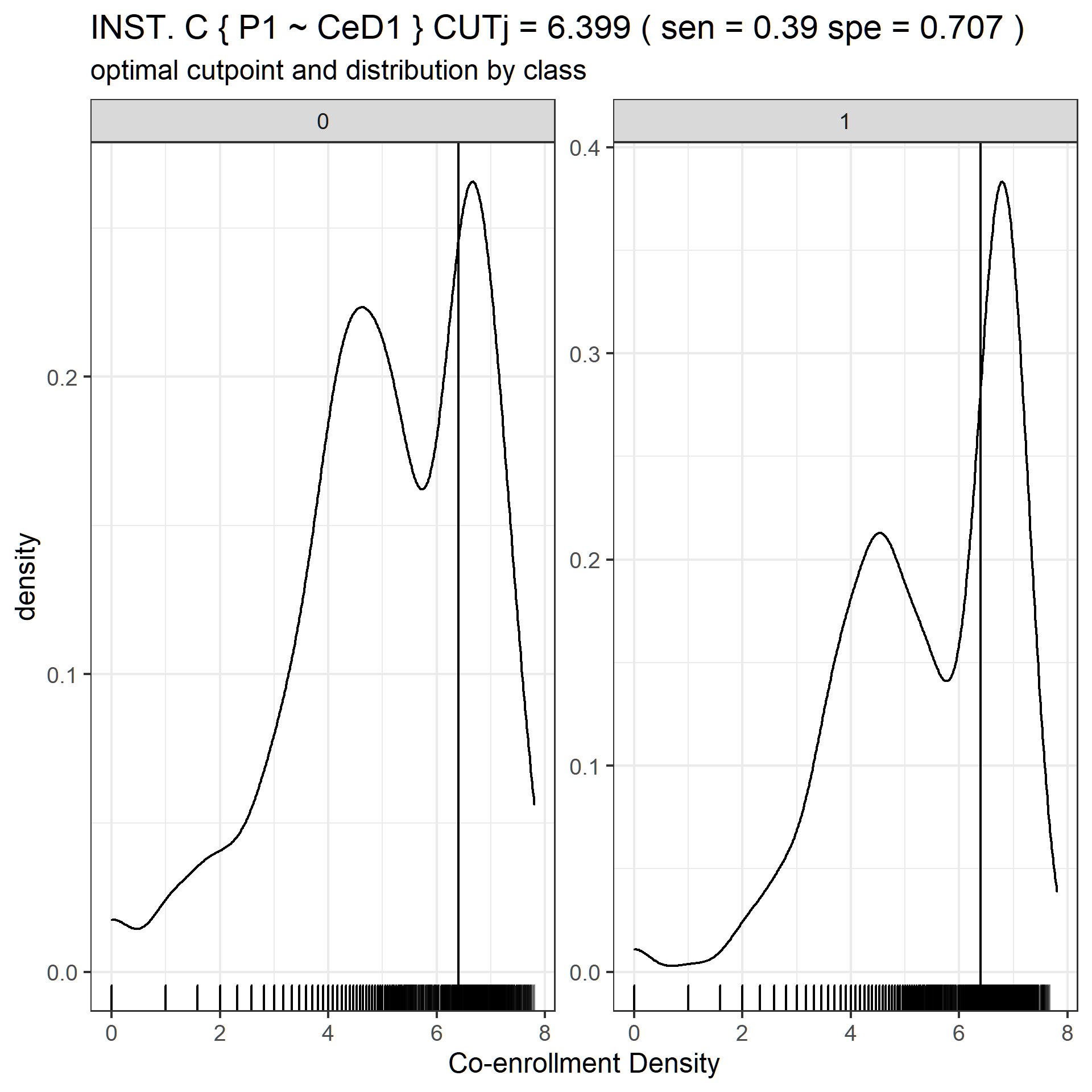

Supplement: Supplementary file 1 [file mmc1.zip › SupplementaryMaterials/215-ClassDen.png]

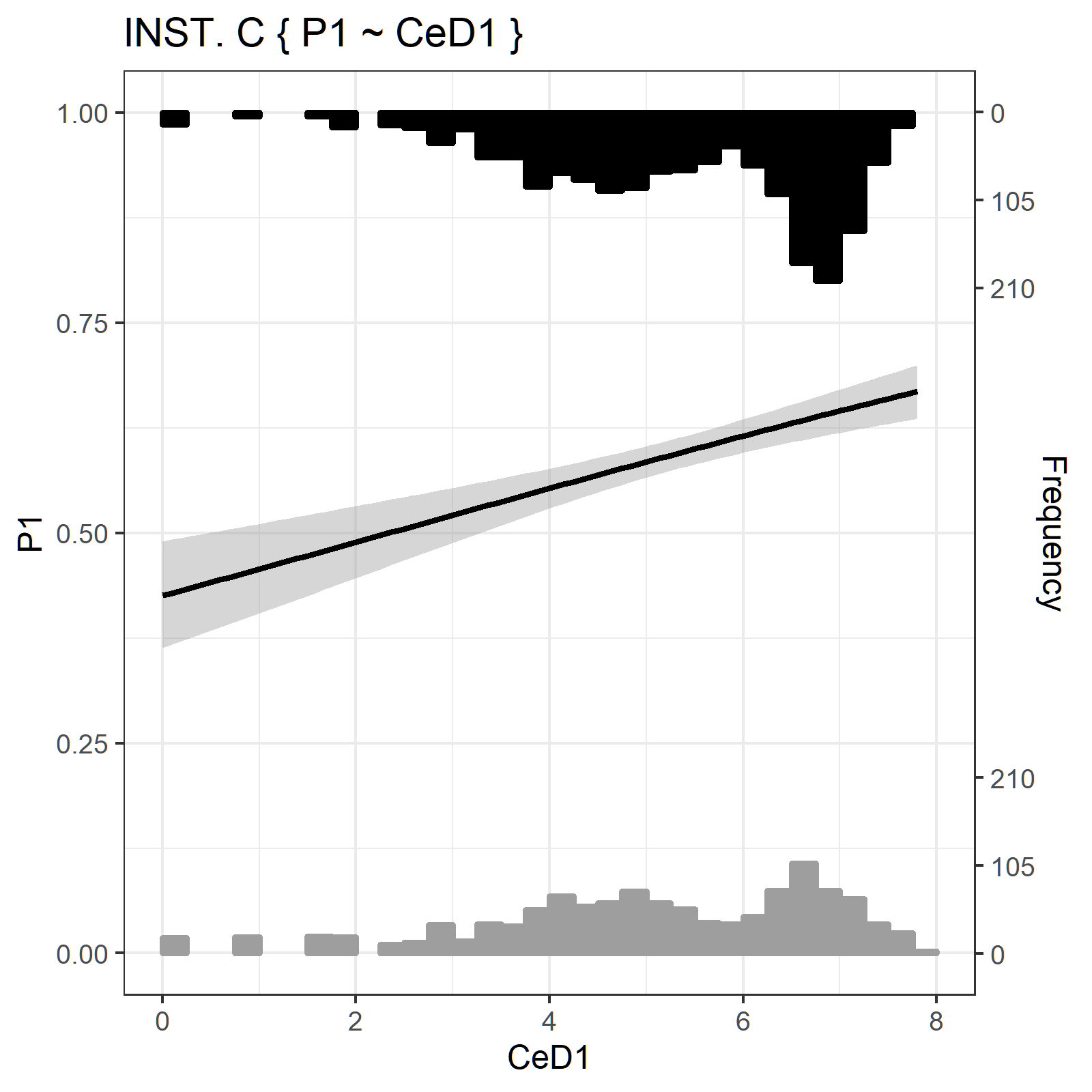

Supplement: Supplementary file 1 [file mmc1.zip › SupplementaryMaterials/215-LogitCurve.png]

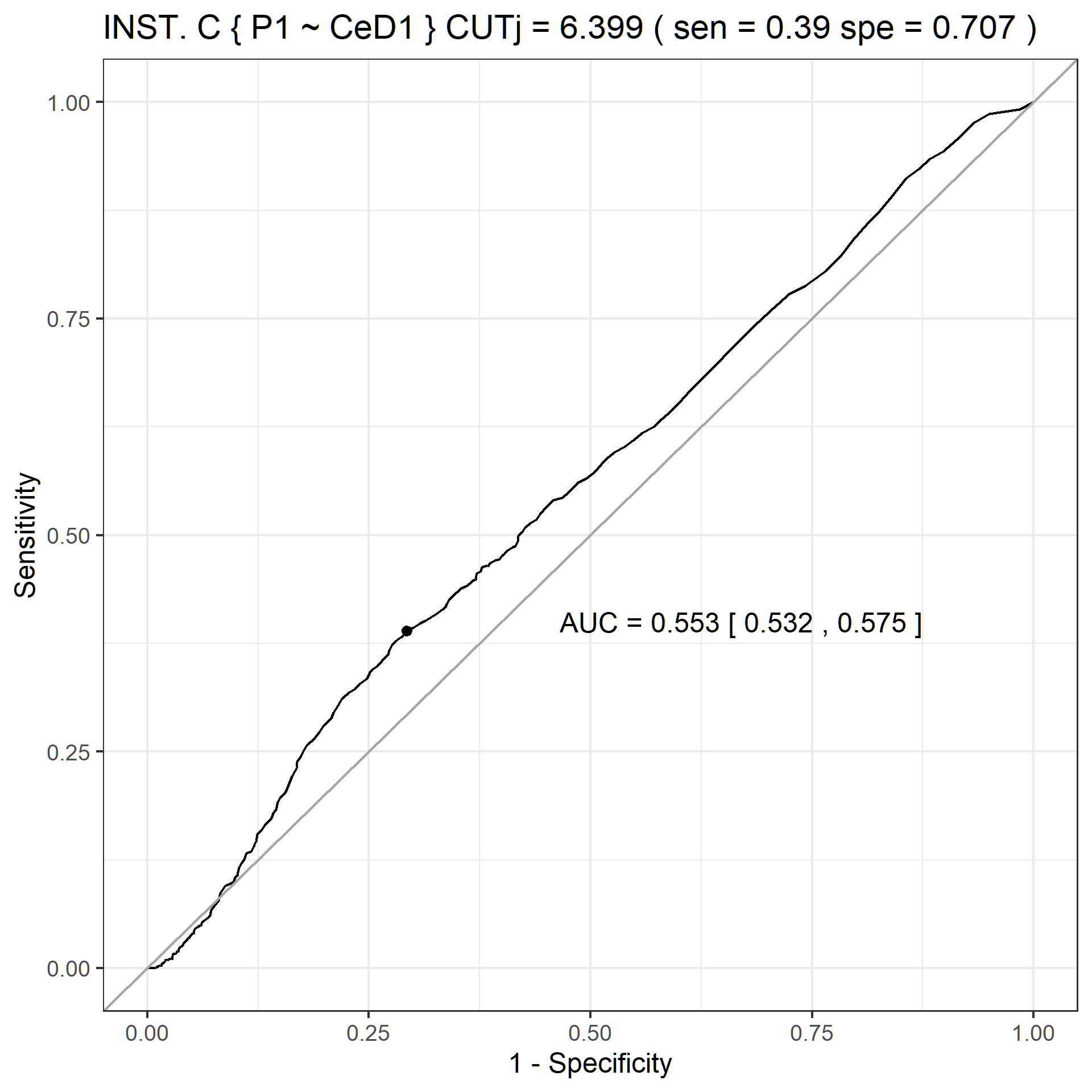

Supplement: Supplementary file 1 [file mmc1.zip › SupplementaryMaterials/215-ROCut.png]

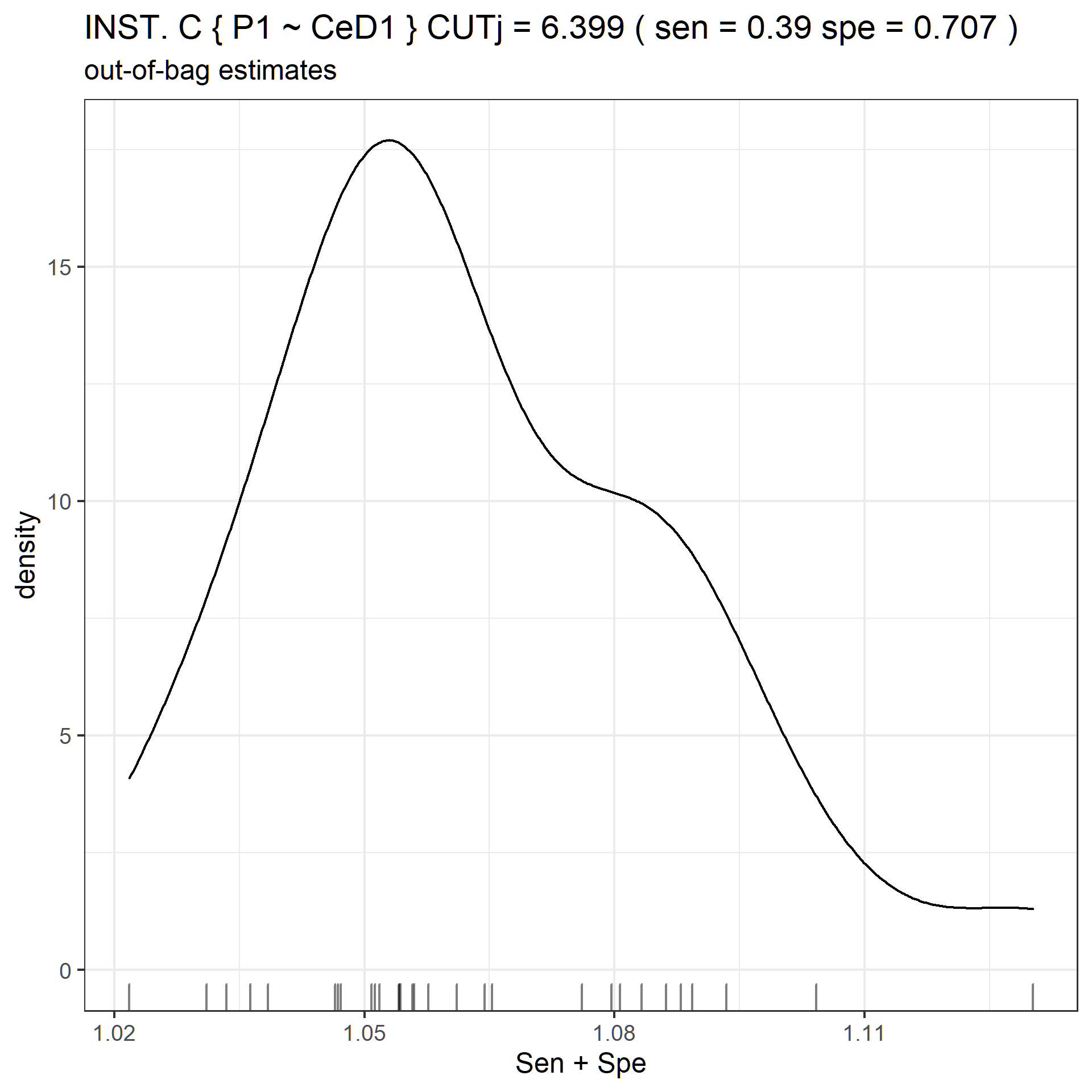

Supplement: Supplementary file 1 [file mmc1.zip › SupplementaryMaterials/215-SenSpe.png]

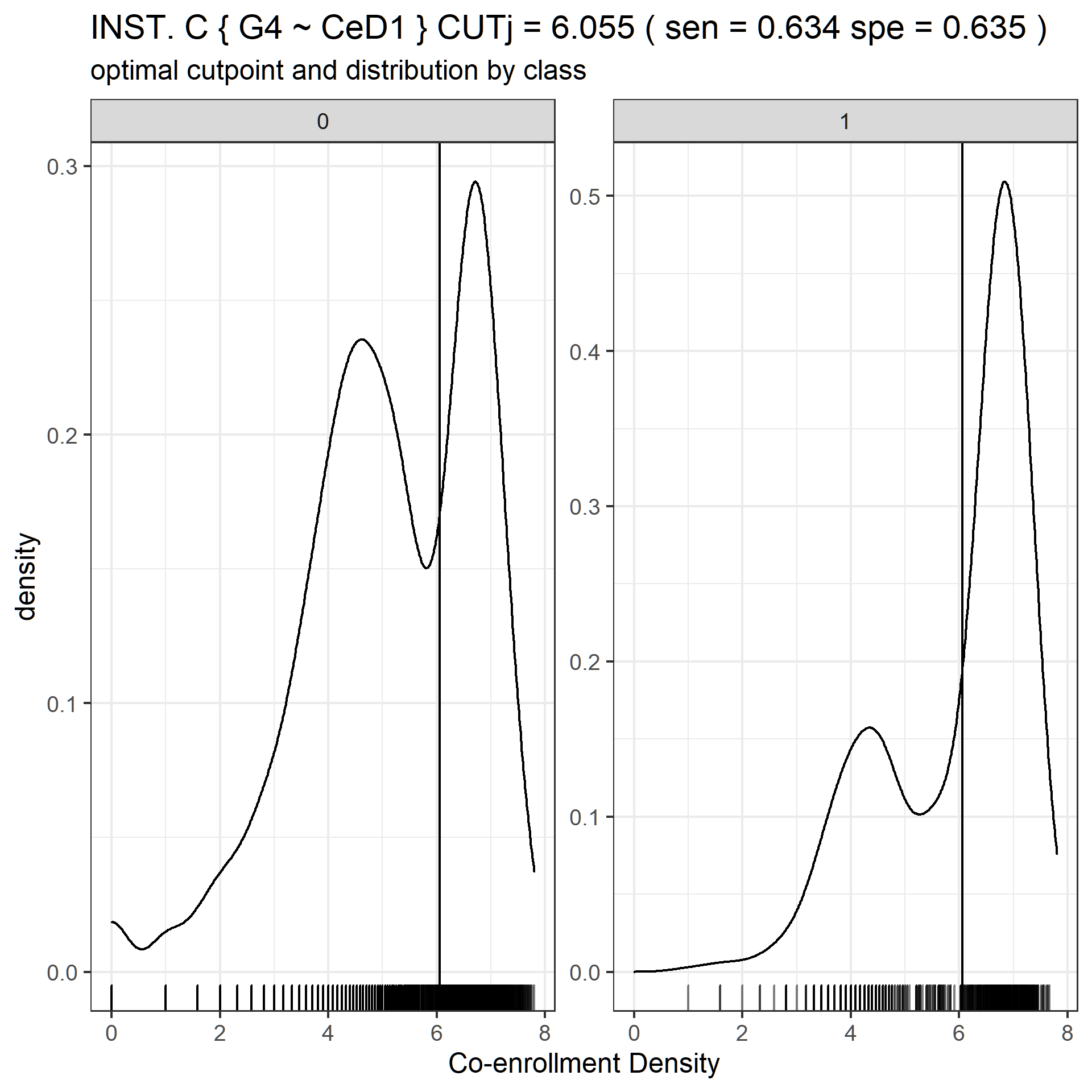

Supplement: Supplementary file 1 [file mmc1.zip › SupplementaryMaterials/216-ClassDen.png]

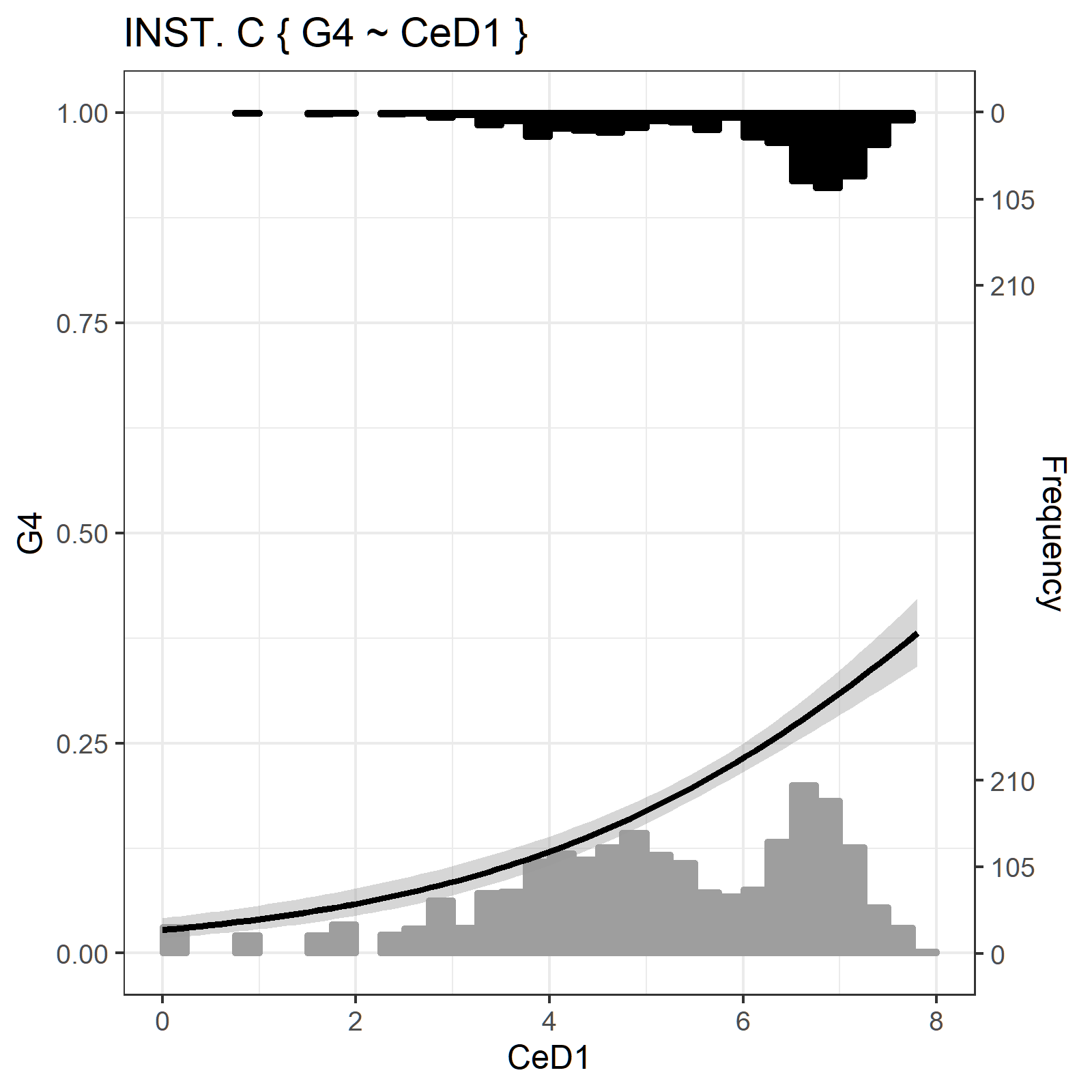

Supplement: Supplementary file 1 [file mmc1.zip › SupplementaryMaterials/216-LogitCurve.png]

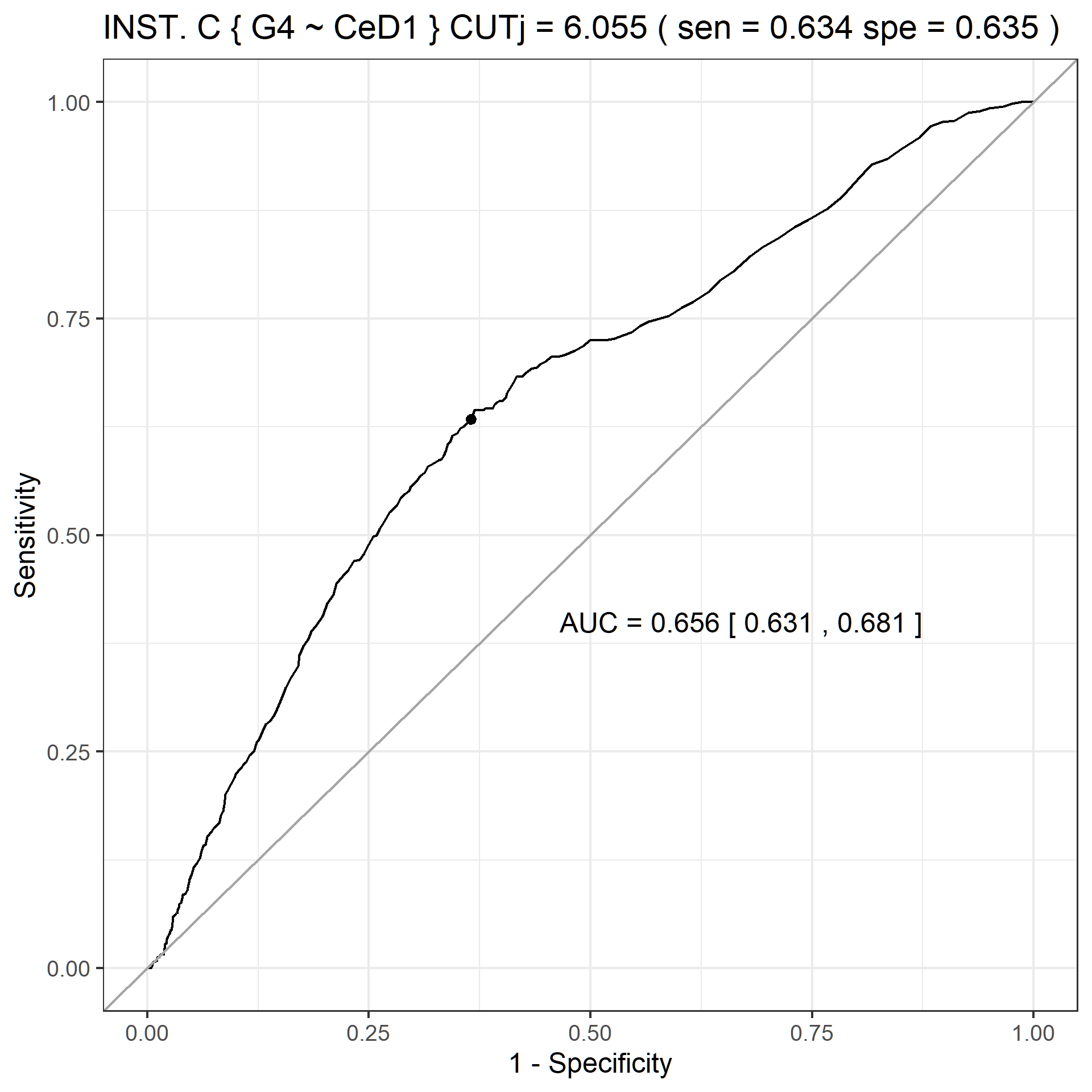

Supplement: Supplementary file 1 [file mmc1.zip › SupplementaryMaterials/216-ROCut.png]

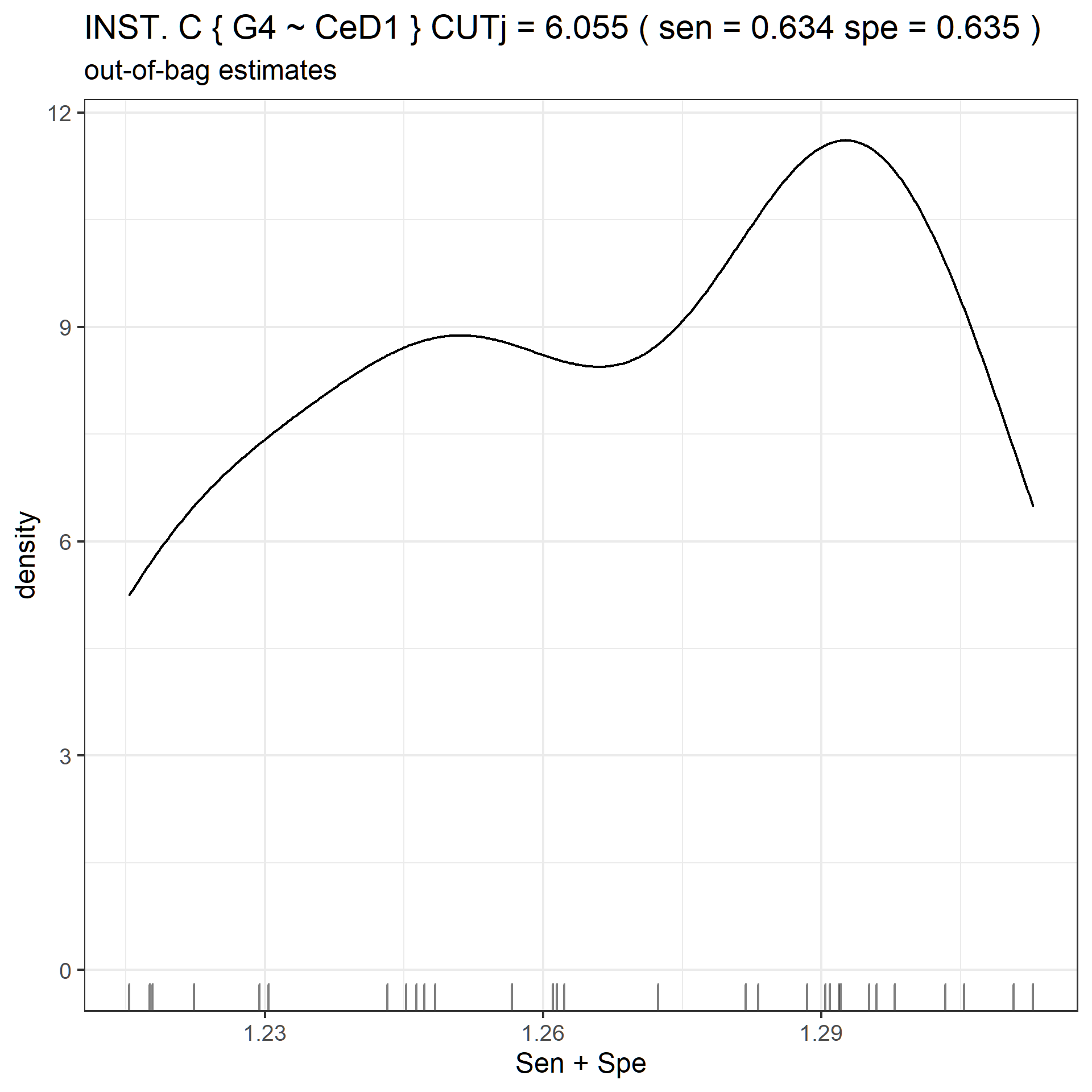

Supplement: Supplementary file 1 [file mmc1.zip › SupplementaryMaterials/216-SenSpe.png]

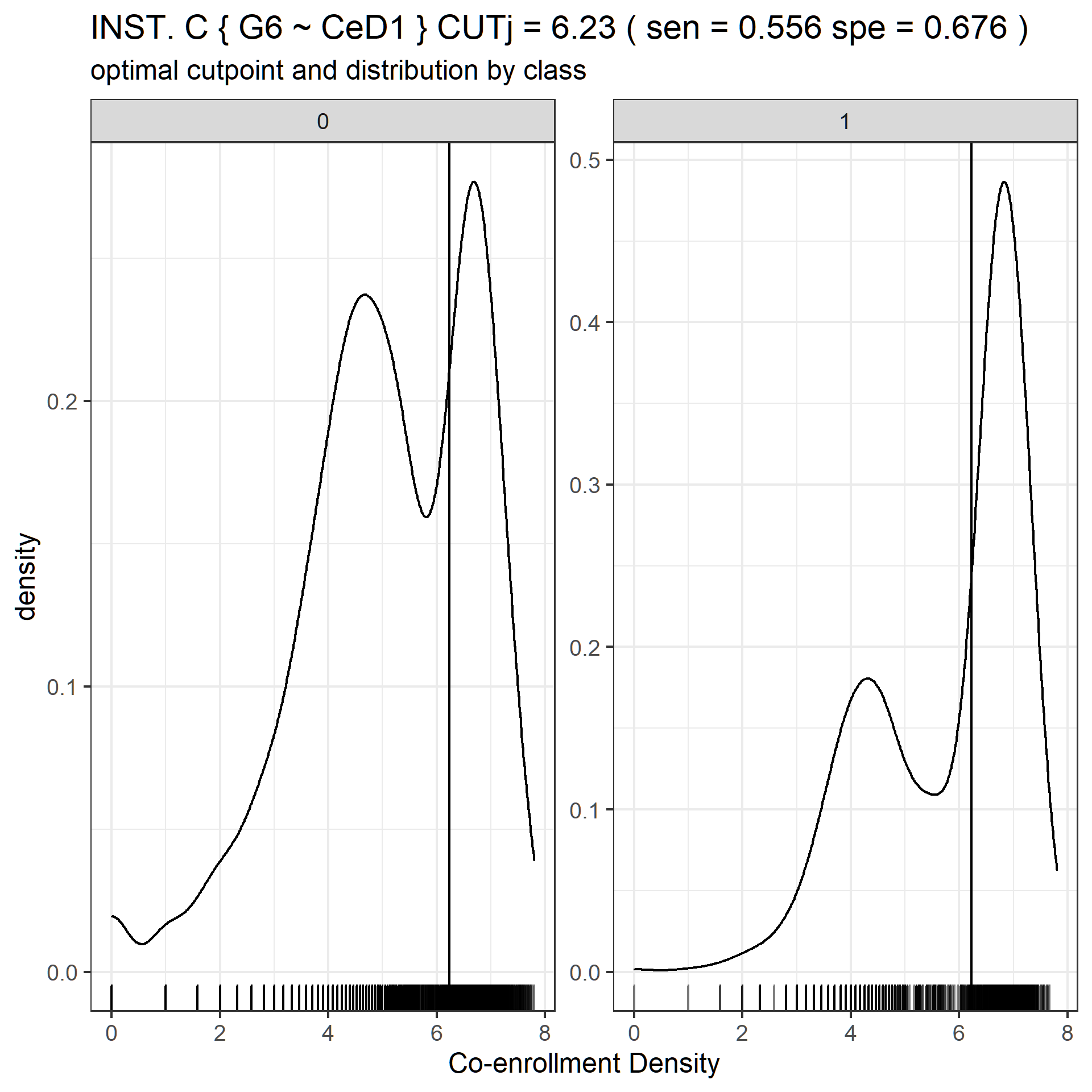

Supplement: Supplementary file 1 [file mmc1.zip › SupplementaryMaterials/217-ClassDen.png]

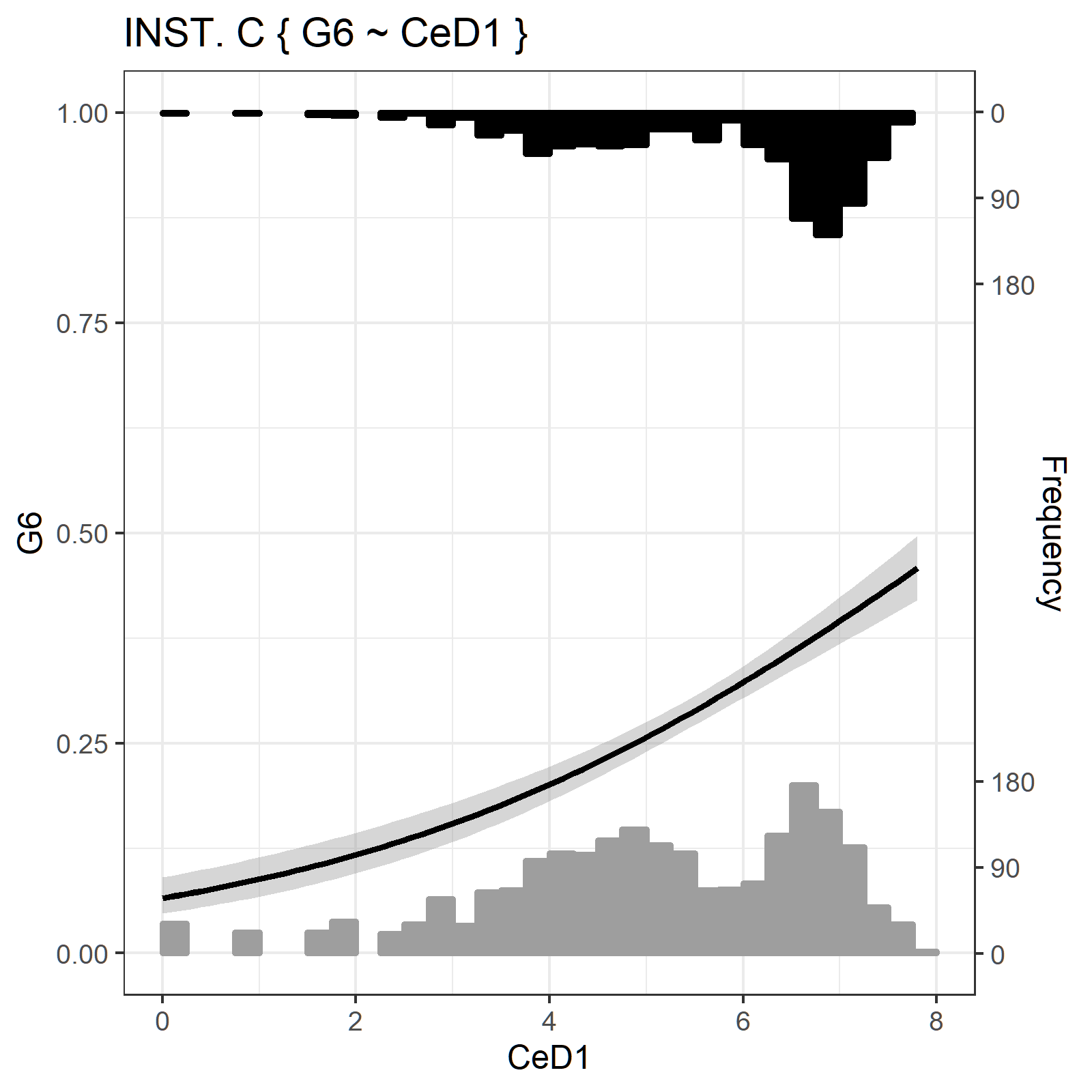

Supplement: Supplementary file 1 [file mmc1.zip › SupplementaryMaterials/217-LogitCurve.png]

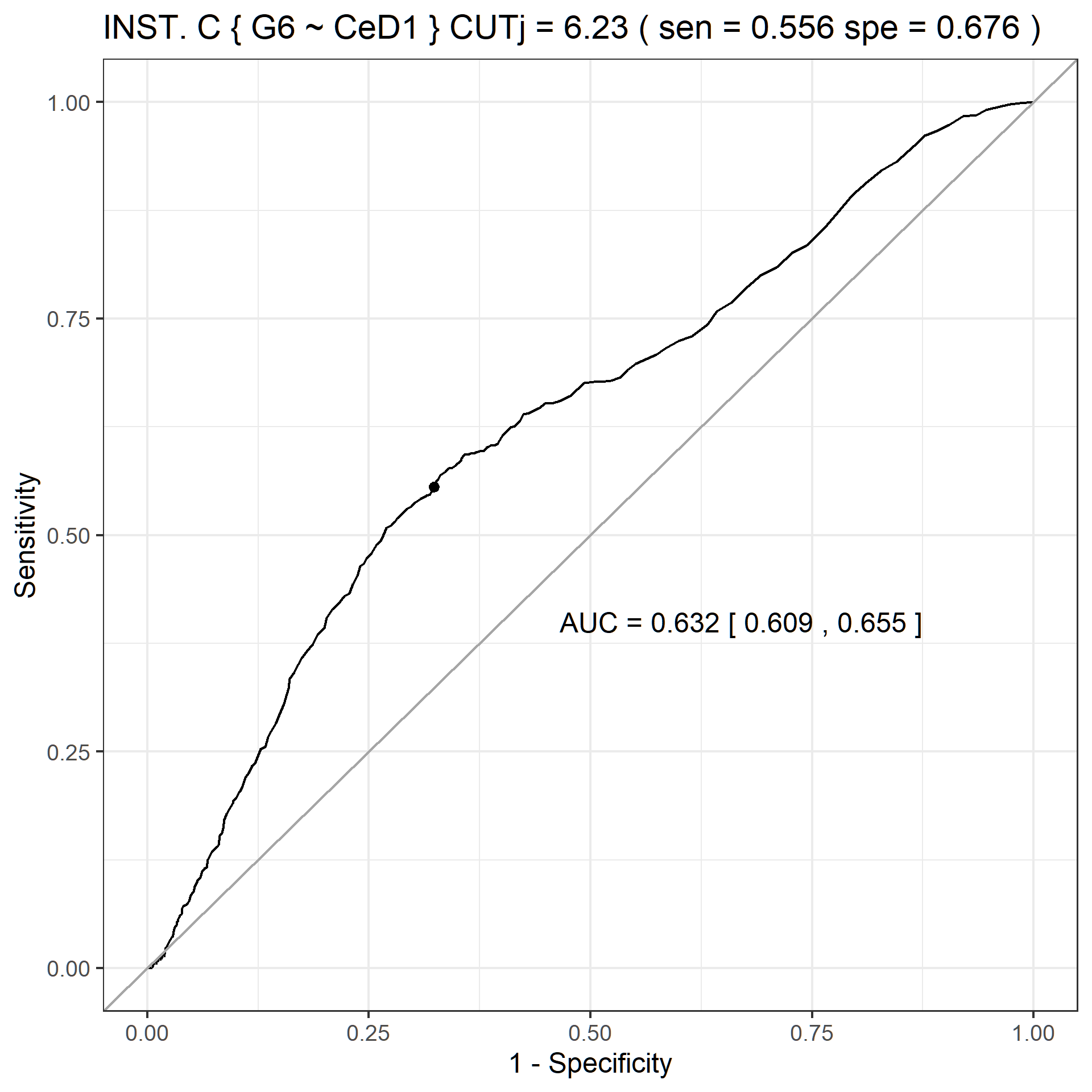

Supplement: Supplementary file 1 [file mmc1.zip › SupplementaryMaterials/217-ROCut.png]

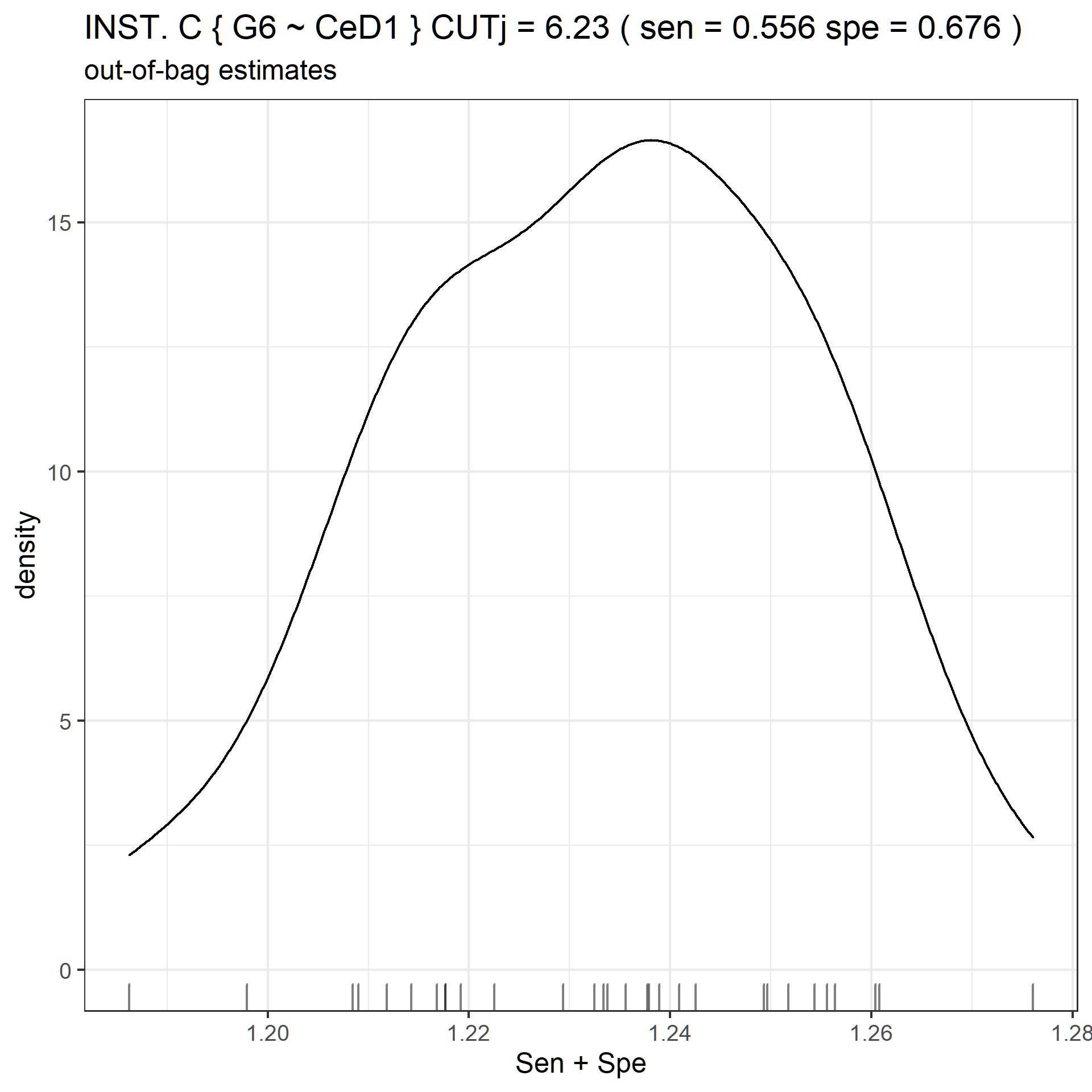

Supplement: Supplementary file 1 [file mmc1.zip › SupplementaryMaterials/217-SenSpe.png]

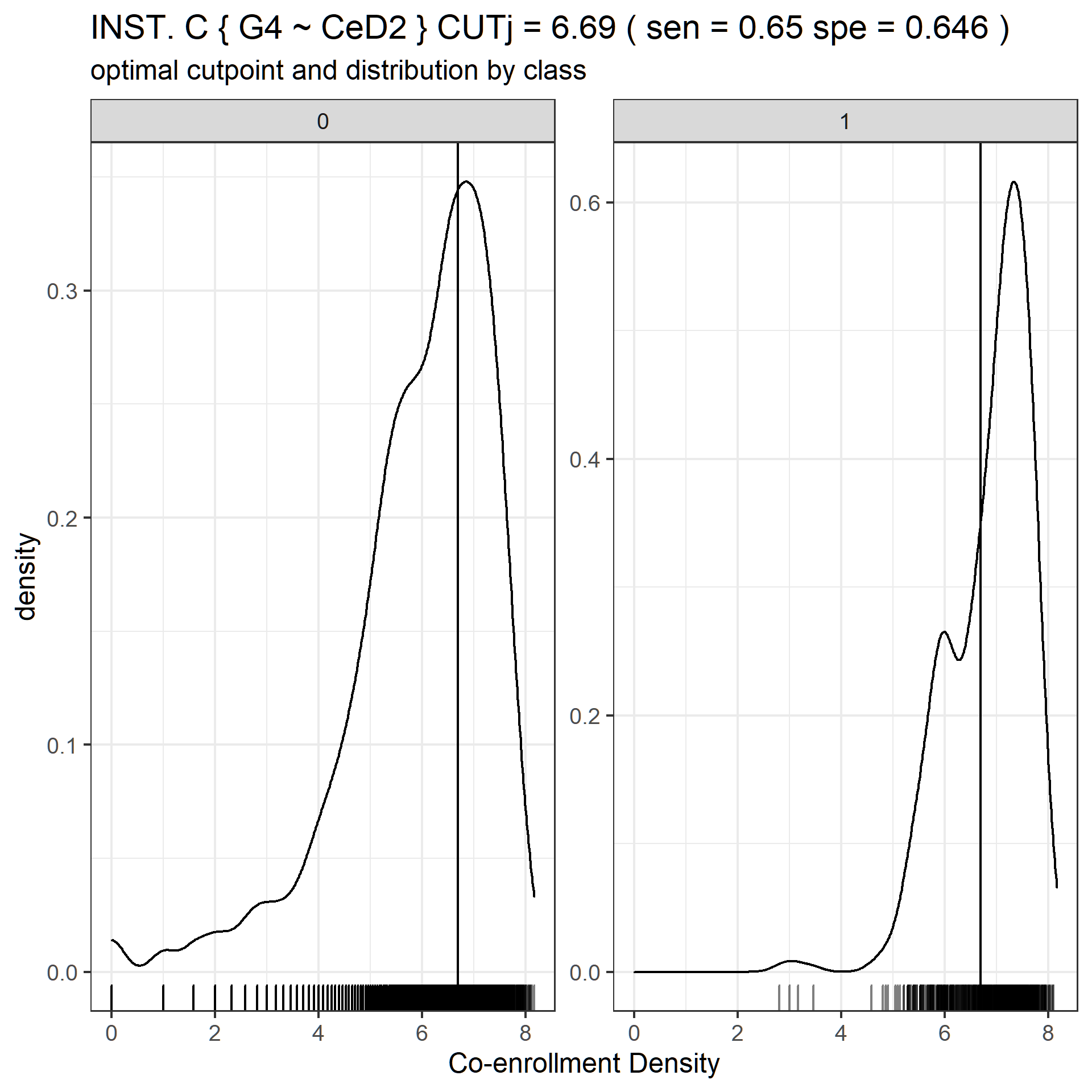

Supplement: Supplementary file 1 [file mmc1.zip › SupplementaryMaterials/226-ClassDen.png]

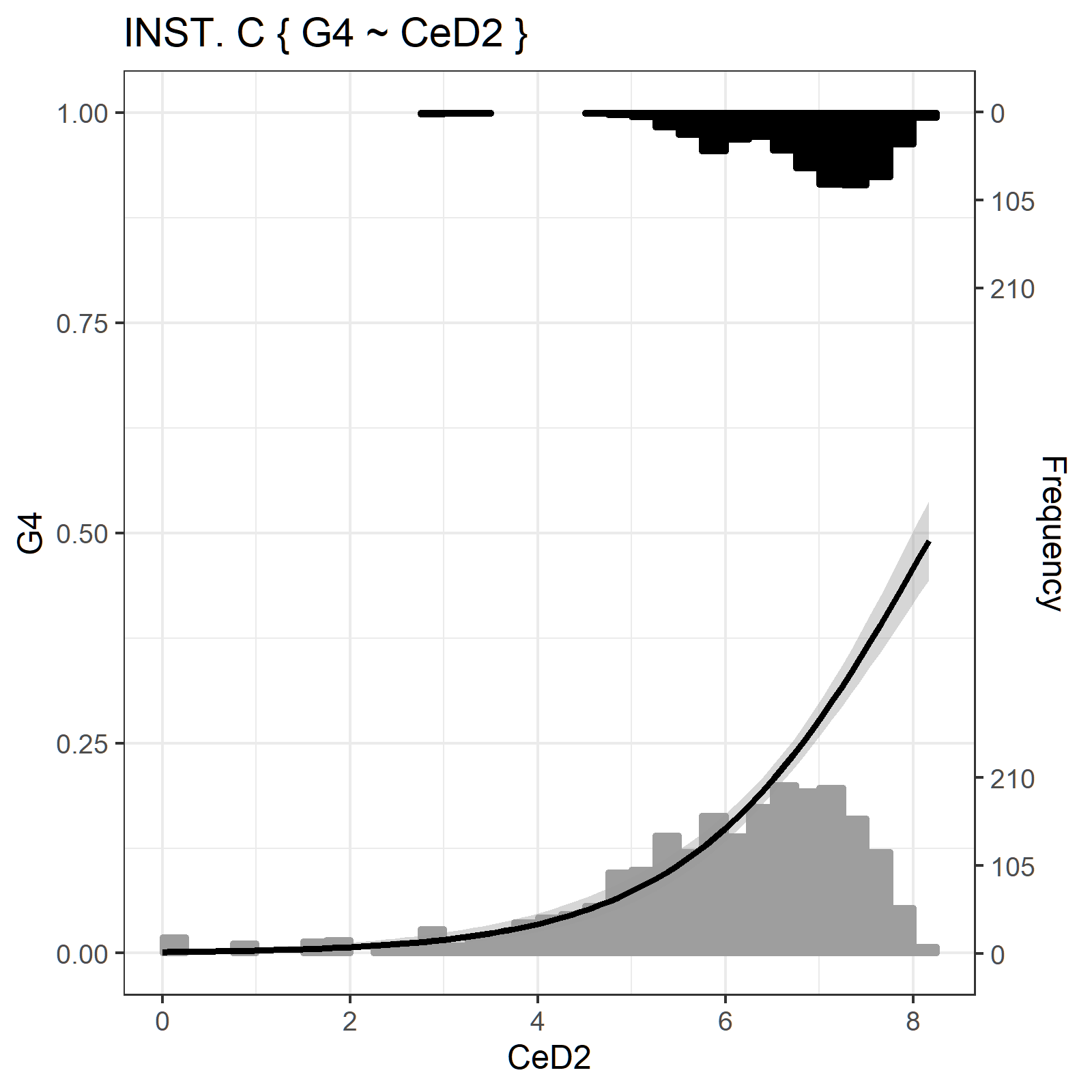

Supplement: Supplementary file 1 [file mmc1.zip › SupplementaryMaterials/226-LogitCurve.png]

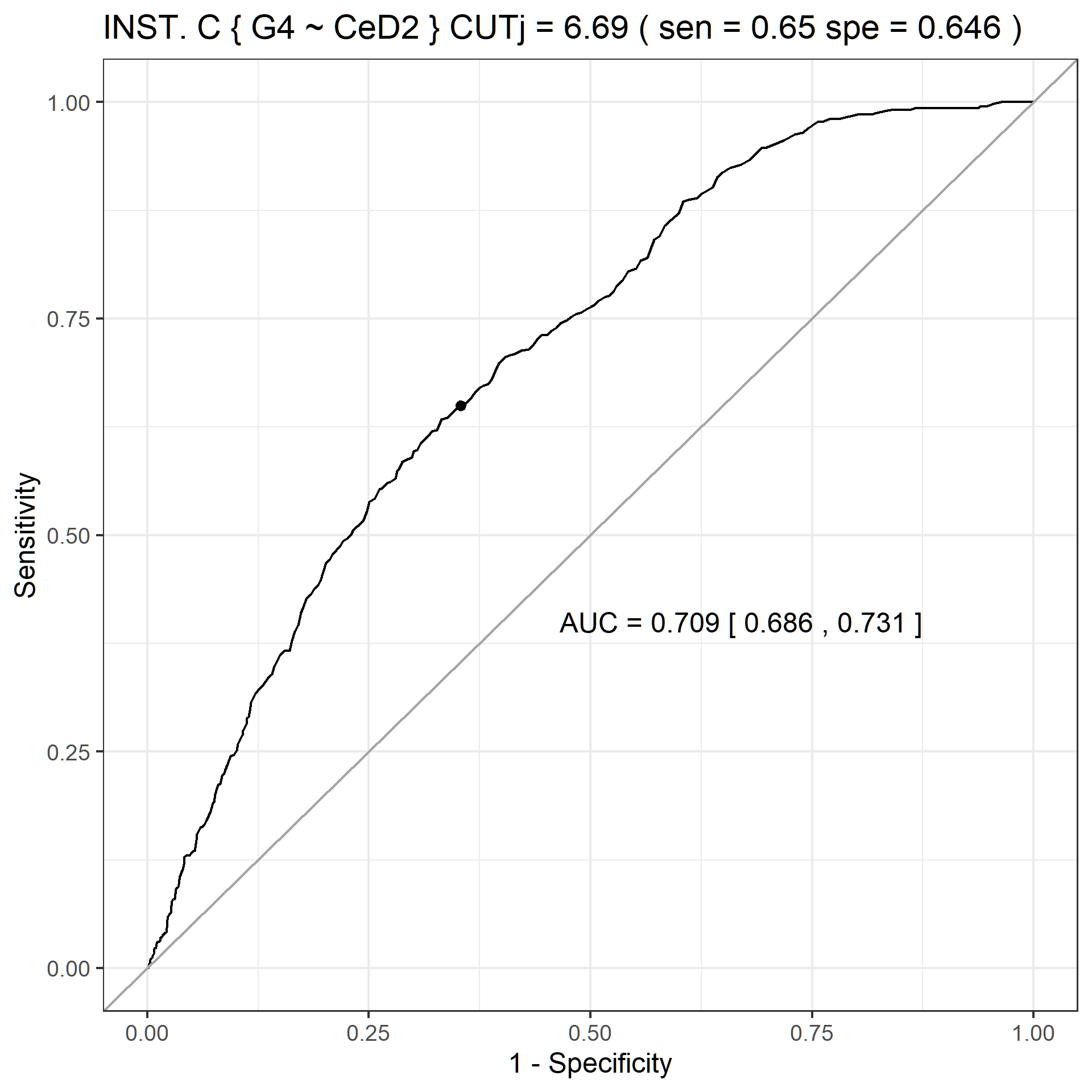

Supplement: Supplementary file 1 [file mmc1.zip › SupplementaryMaterials/226-ROCut.png]

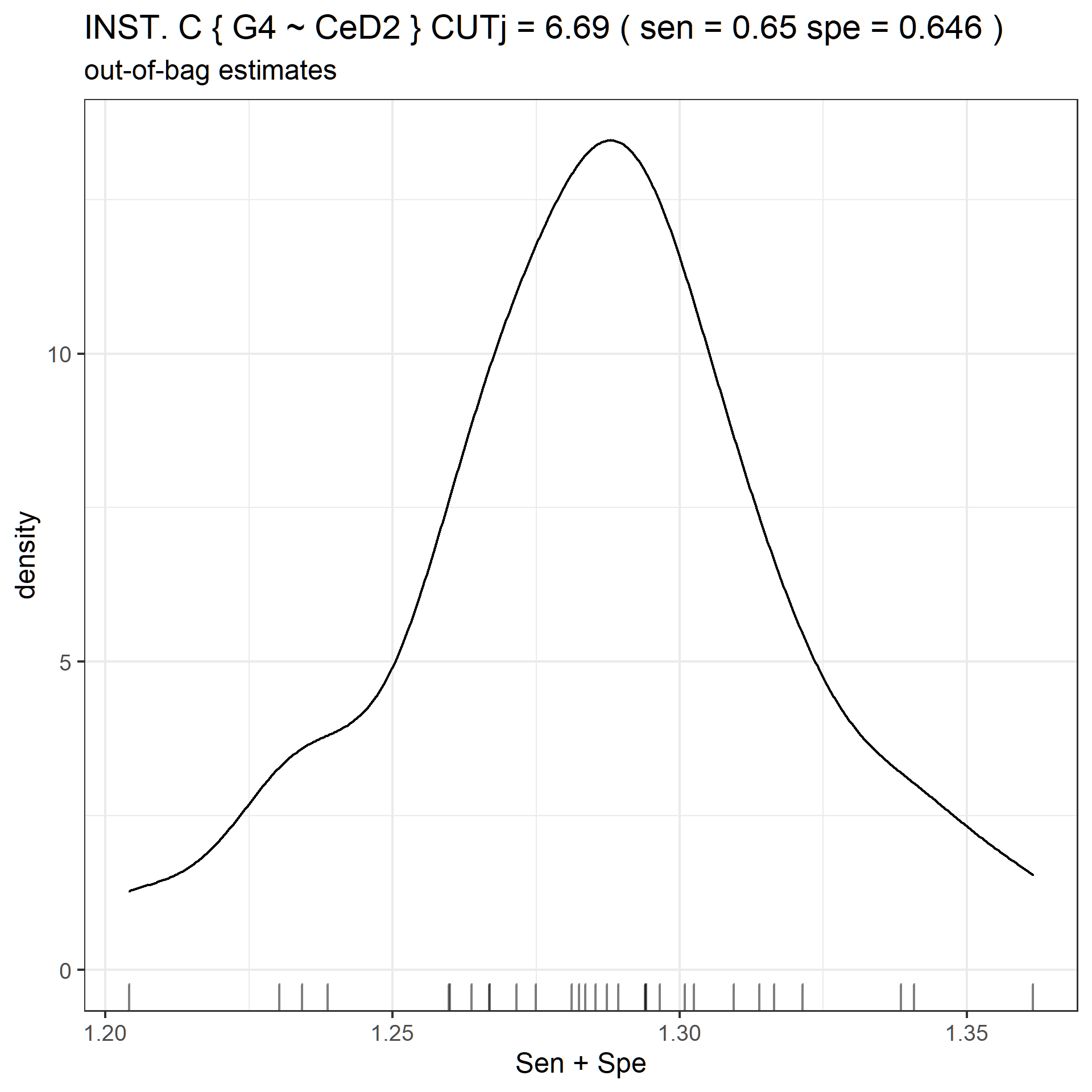

Supplement: Supplementary file 1 [file mmc1.zip › SupplementaryMaterials/226-SenSpe.png]

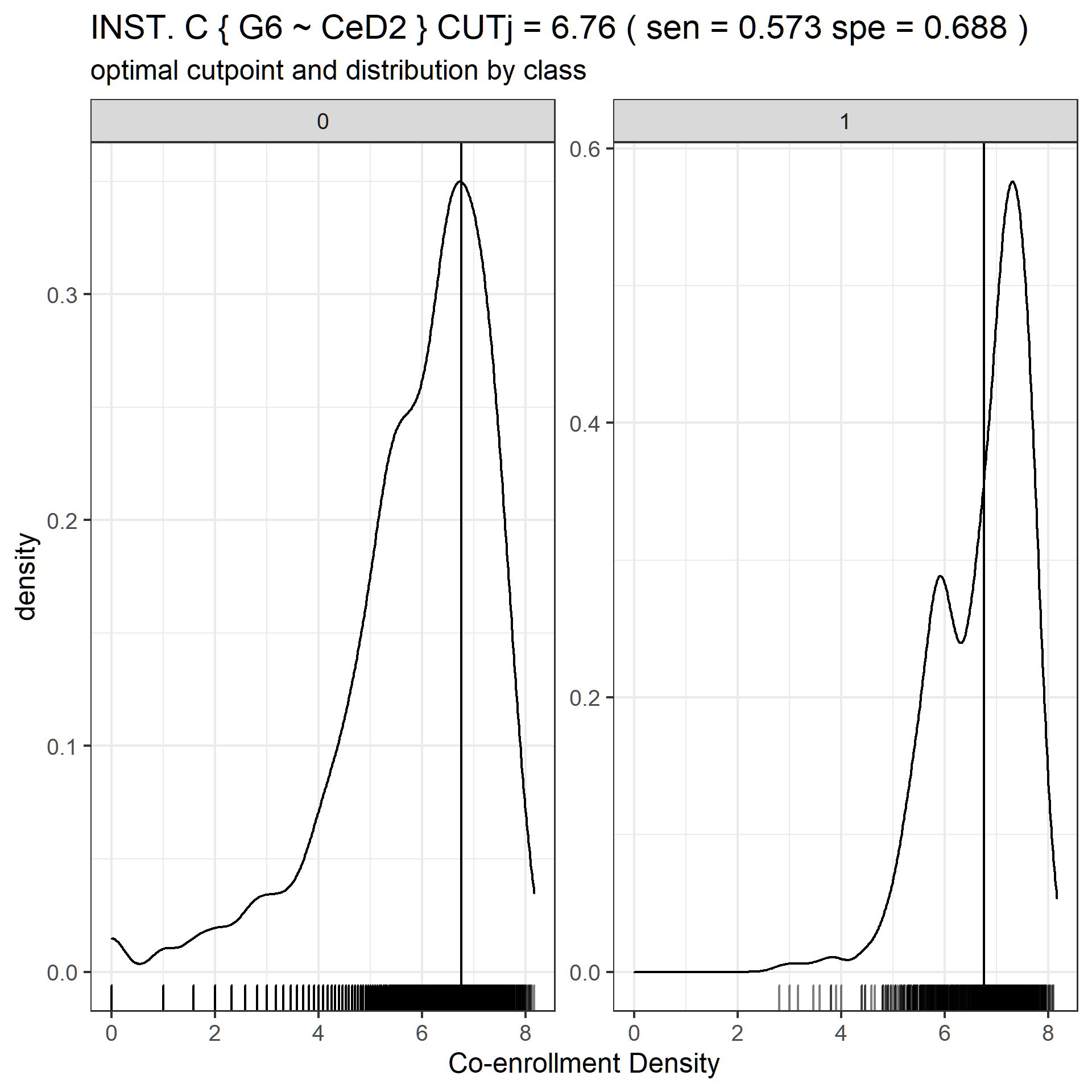

Supplement: Supplementary file 1 [file mmc1.zip › SupplementaryMaterials/227-ClassDen.png]

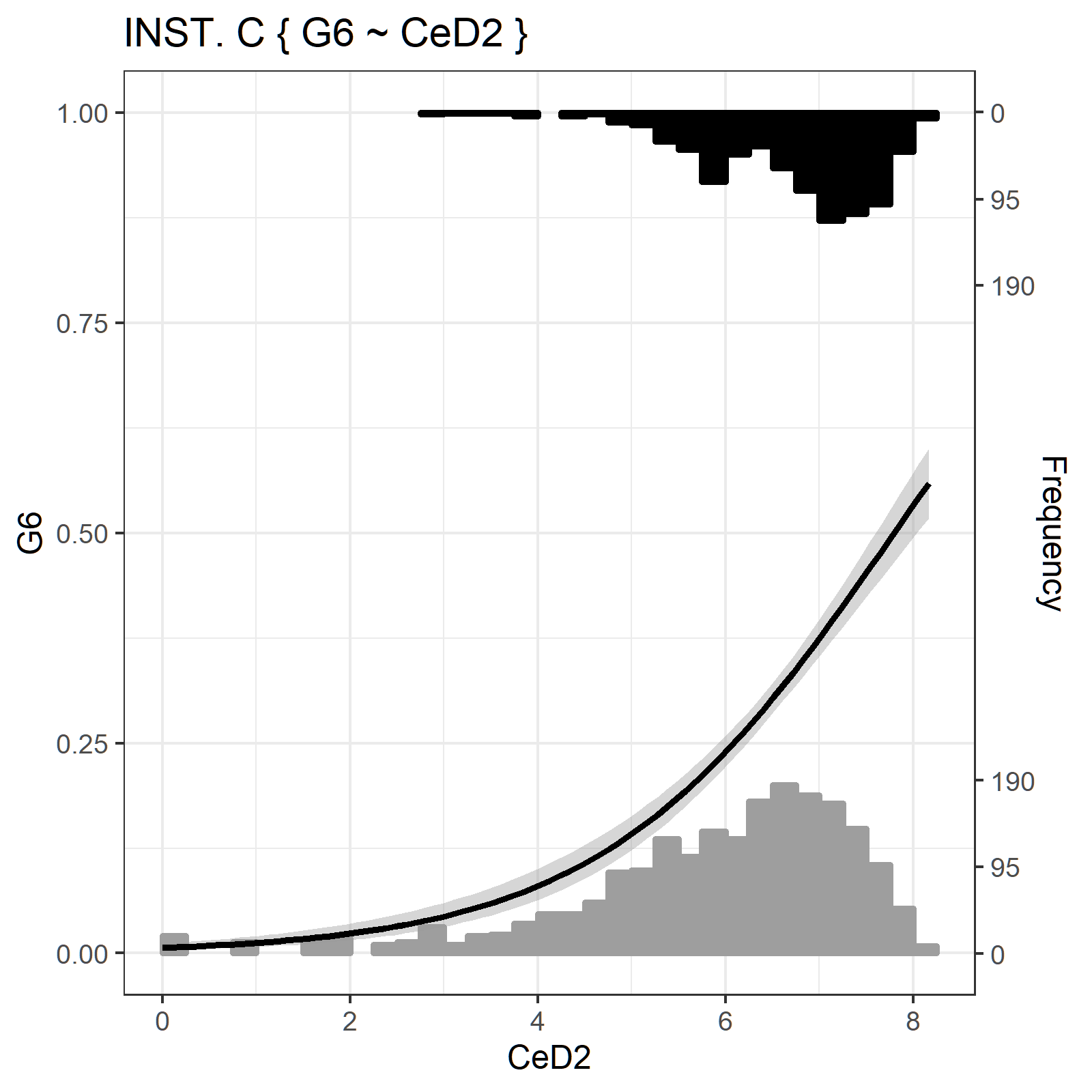

Supplement: Supplementary file 1 [file mmc1.zip › SupplementaryMaterials/227-LogitCurve.png]

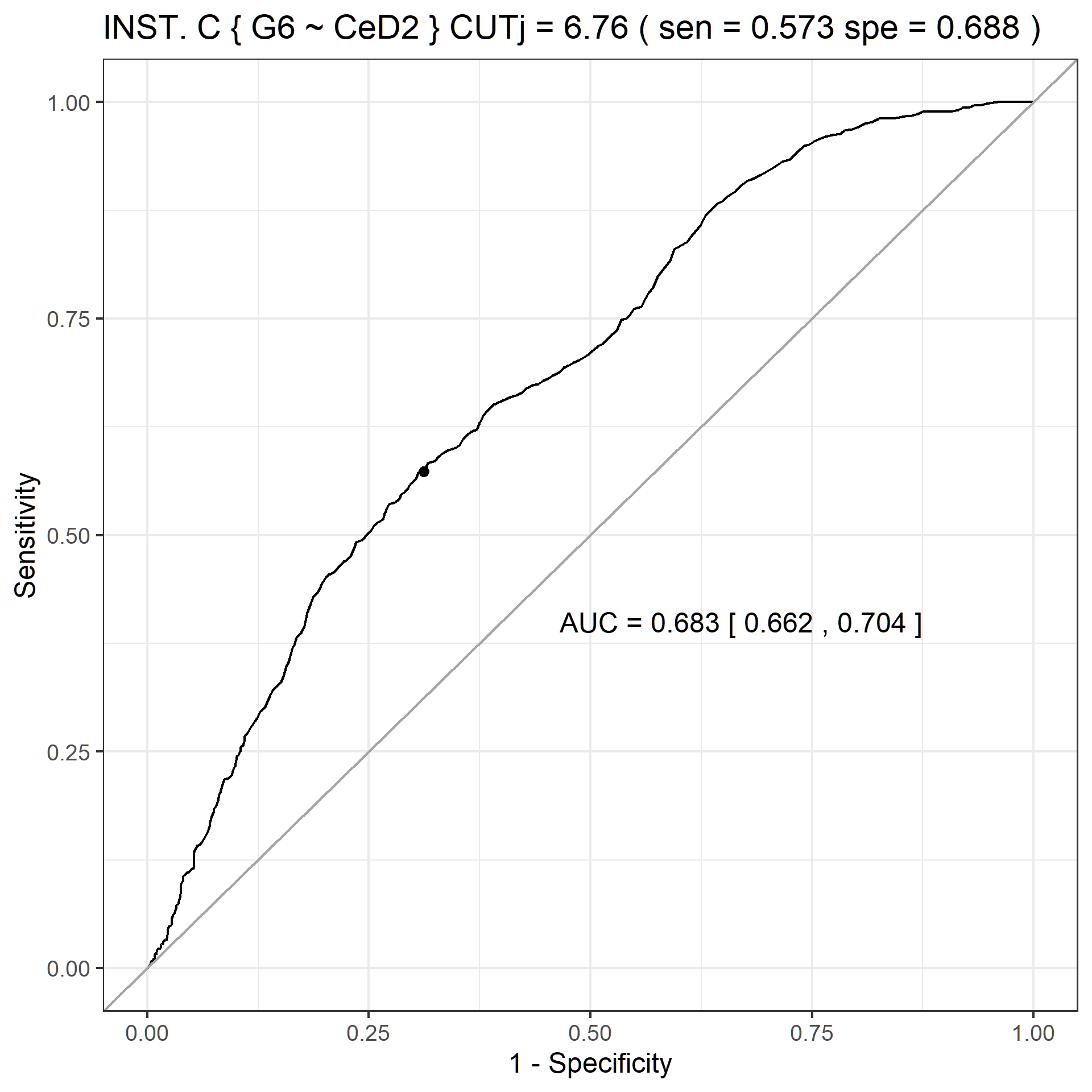

Supplement: Supplementary file 1 [file mmc1.zip › SupplementaryMaterials/227-ROCut.png]

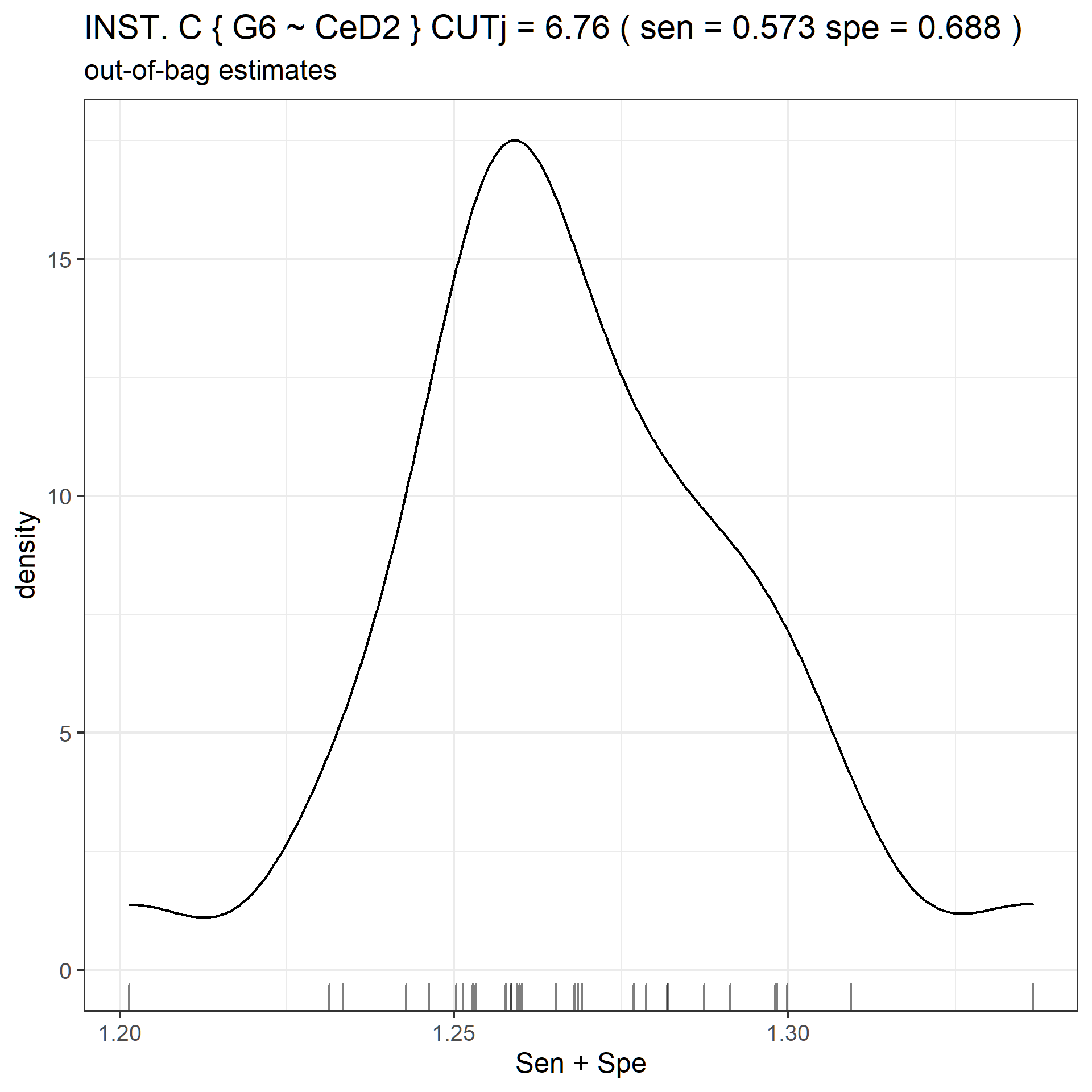

Supplement: Supplementary file 1 [file mmc1.zip › SupplementaryMaterials/227-SenSpe.png]

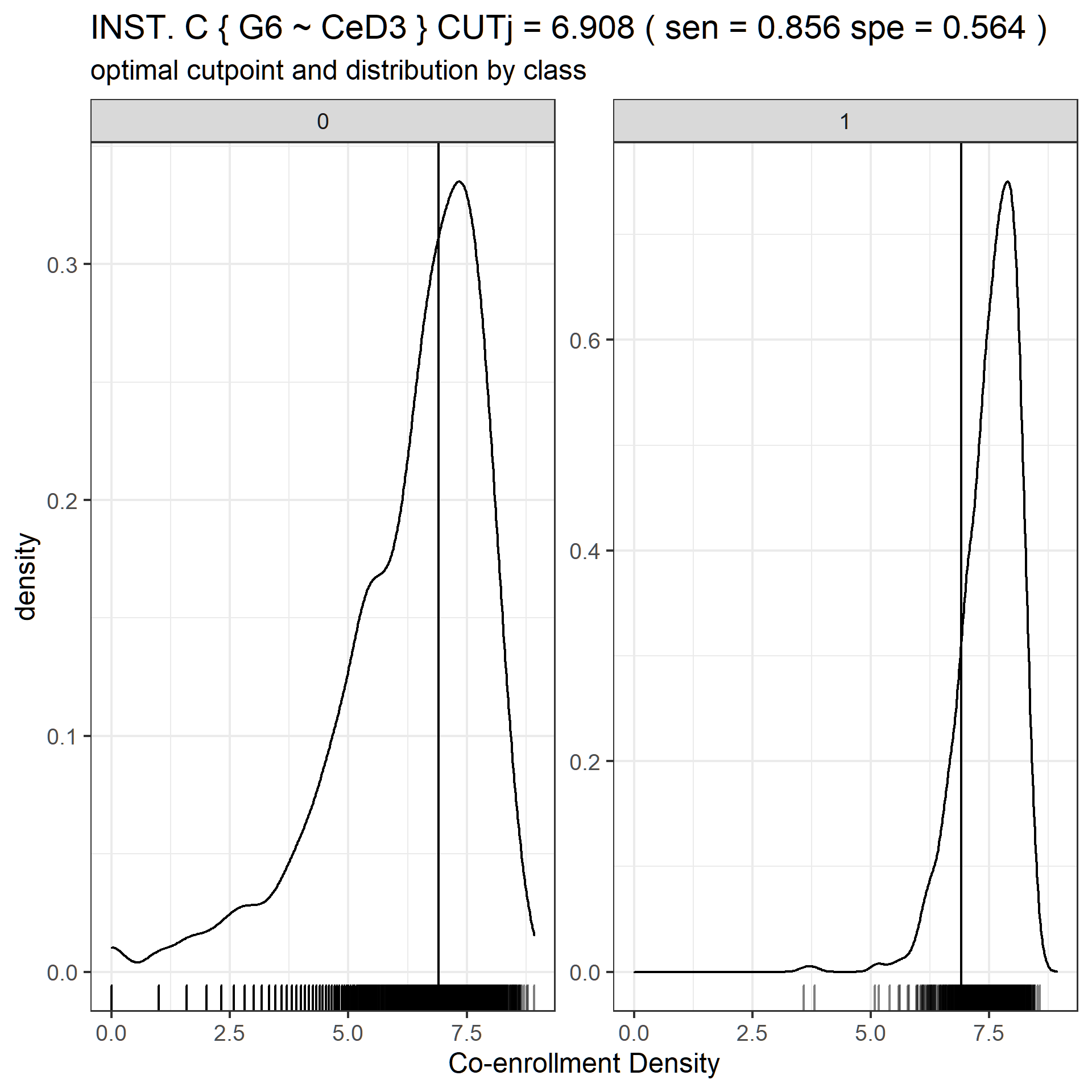

Supplement: Supplementary file 1 [file mmc1.zip › SupplementaryMaterials/237-ClassDen.png]

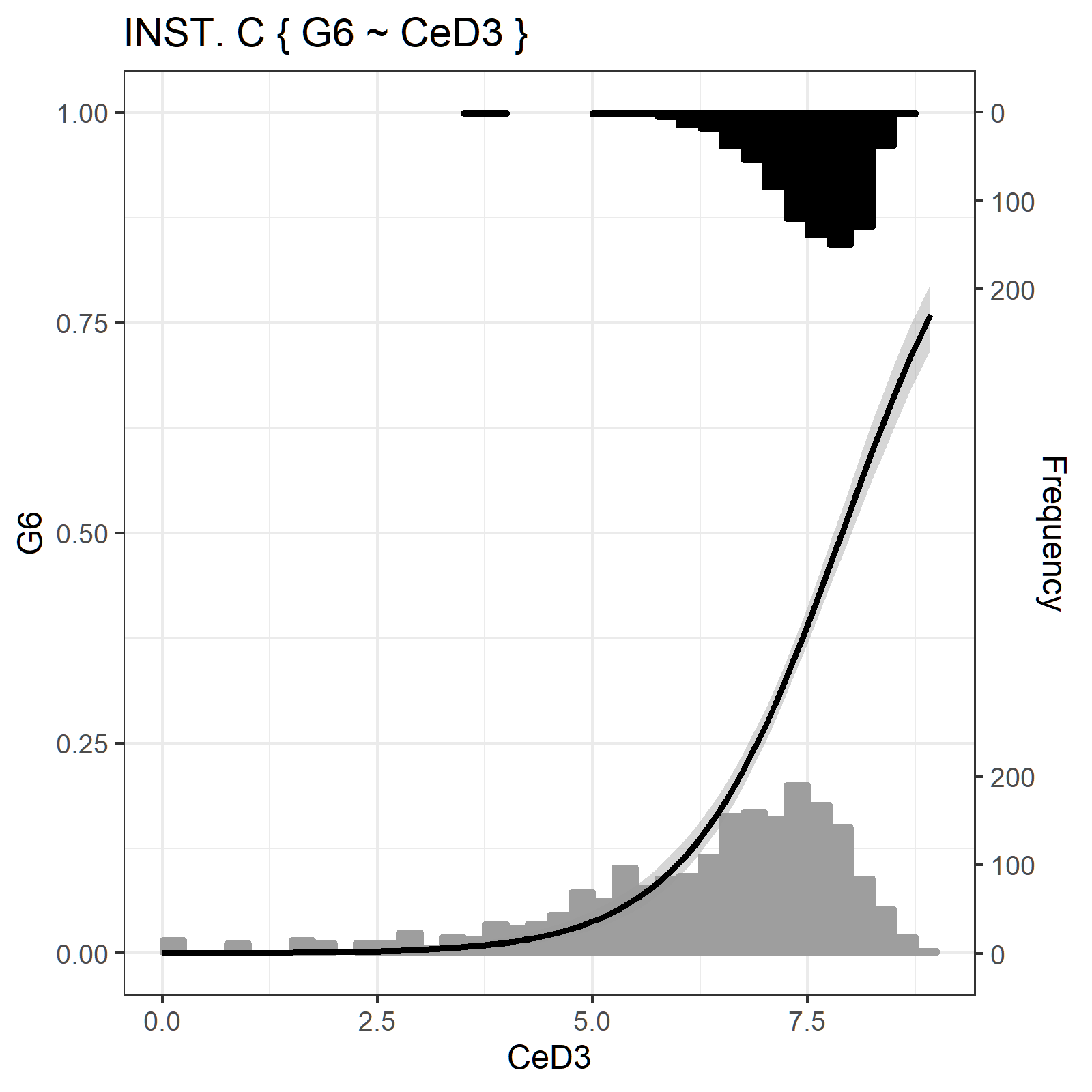

Supplement: Supplementary file 1 [file mmc1.zip › SupplementaryMaterials/237-LogitCurve.png]

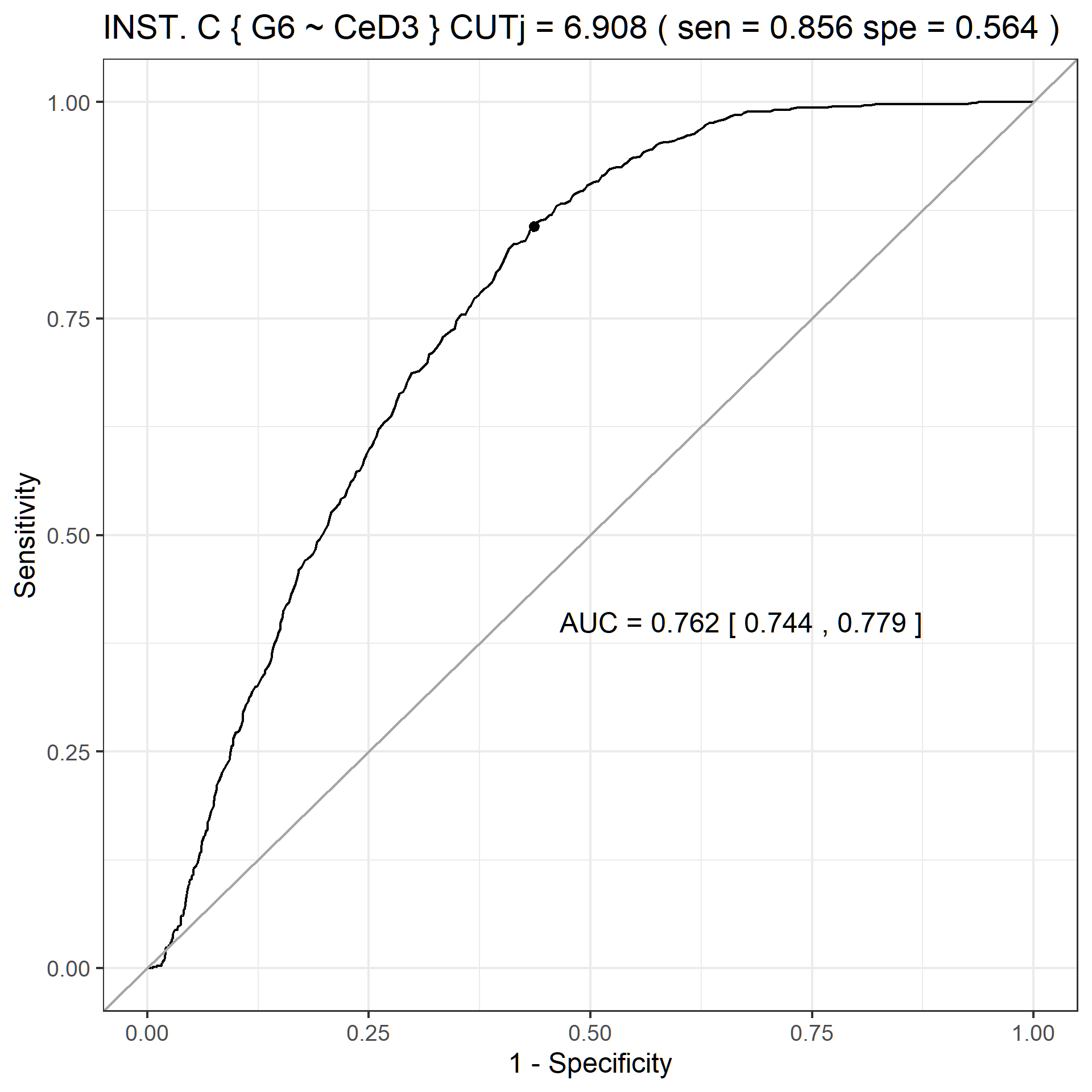

Supplement: Supplementary file 1 [file mmc1.zip › SupplementaryMaterials/237-ROCut.png]

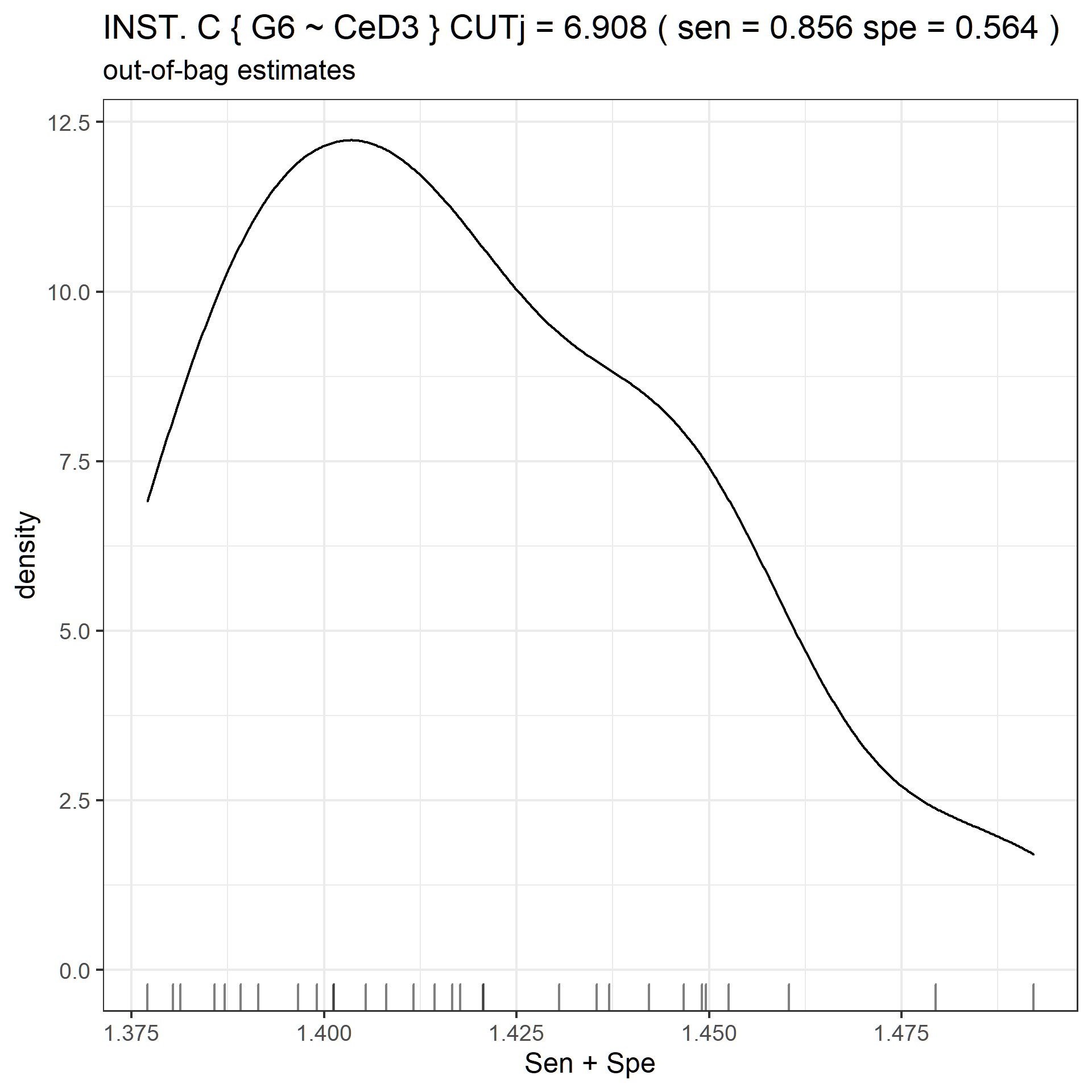

Supplement: Supplementary file 1 [file mmc1.zip › SupplementaryMaterials/237-SenSpe.png]

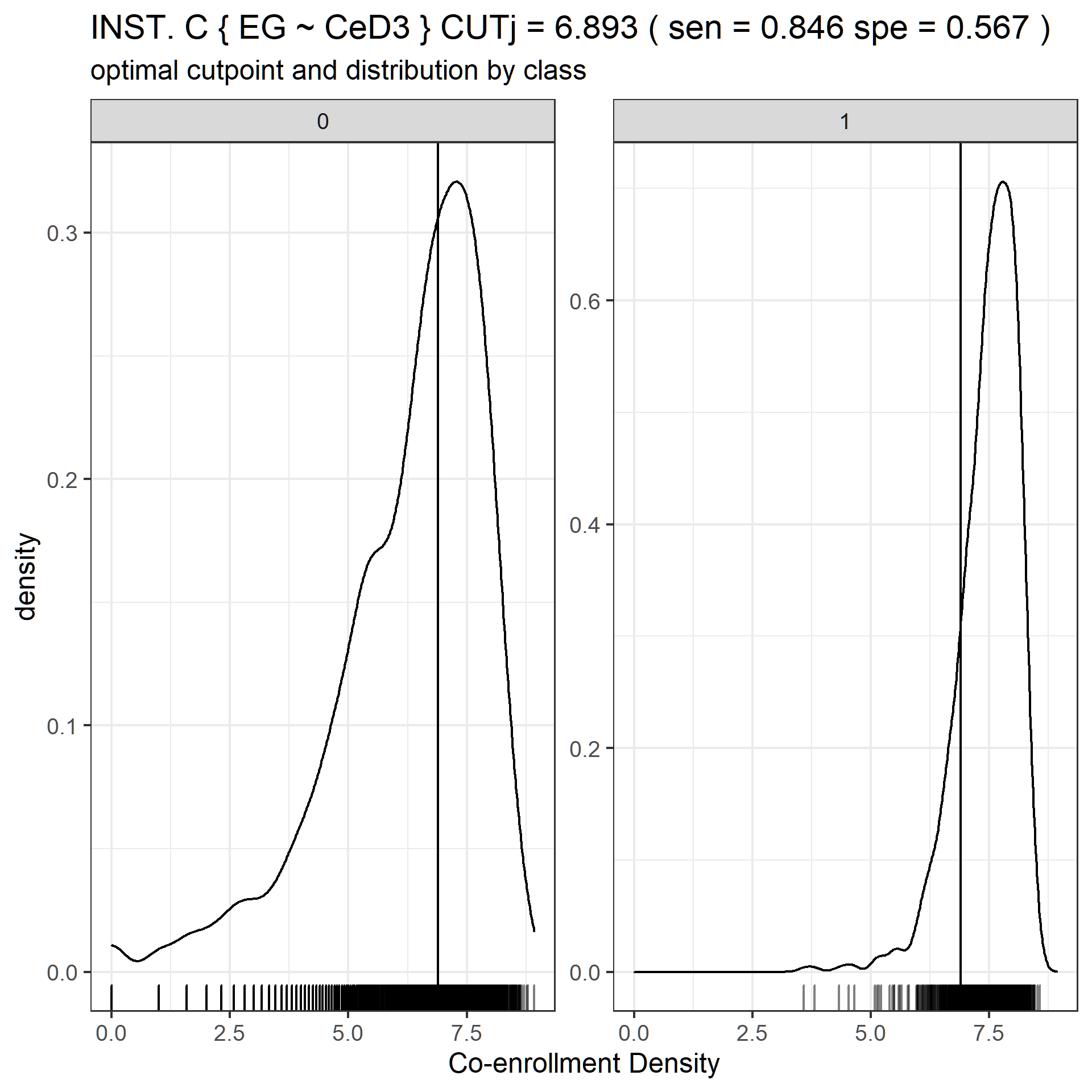

Supplement: Supplementary file 1 [file mmc1.zip › SupplementaryMaterials/238-ClassDen.png]

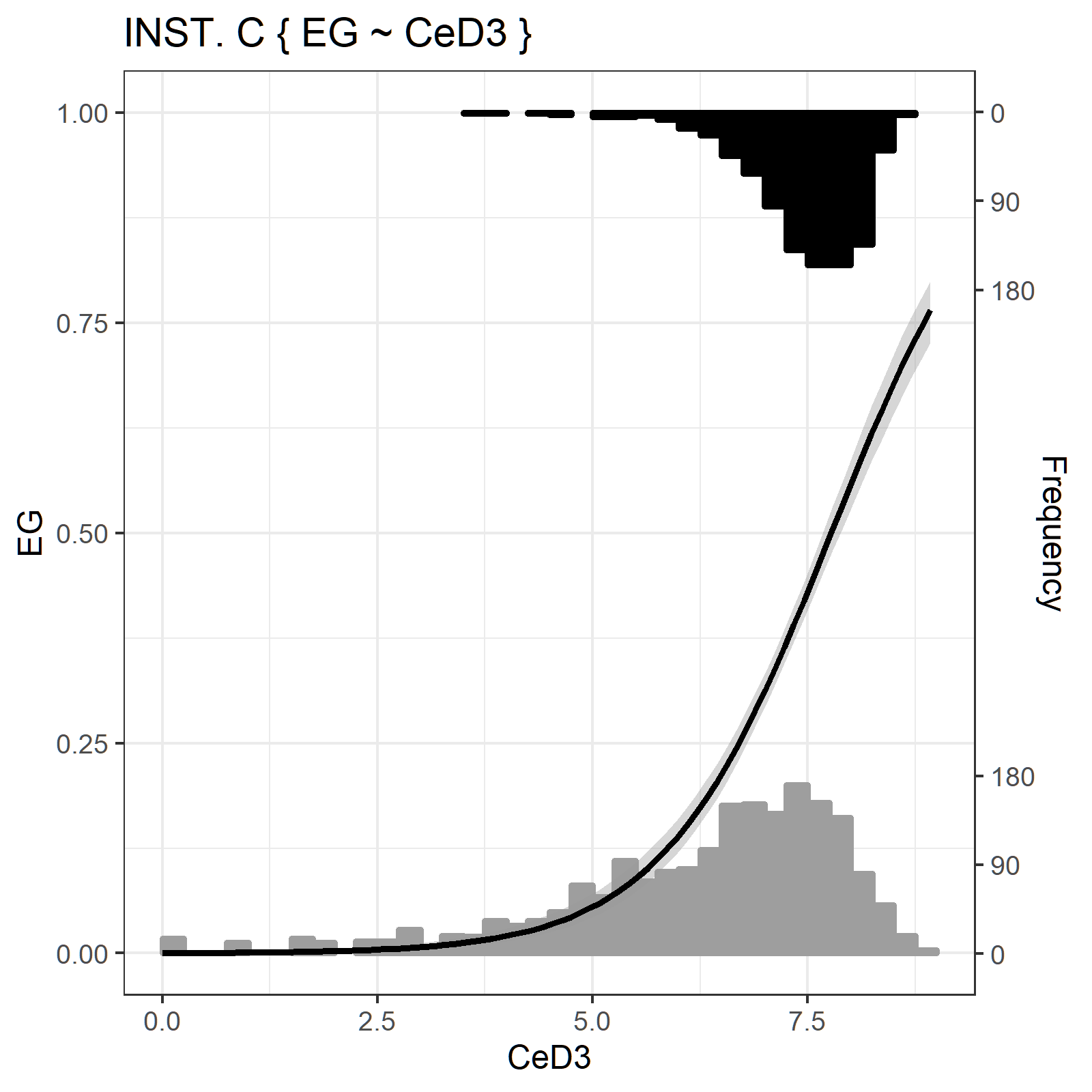

Supplement: Supplementary file 1 [file mmc1.zip › SupplementaryMaterials/238-LogitCurve.png]

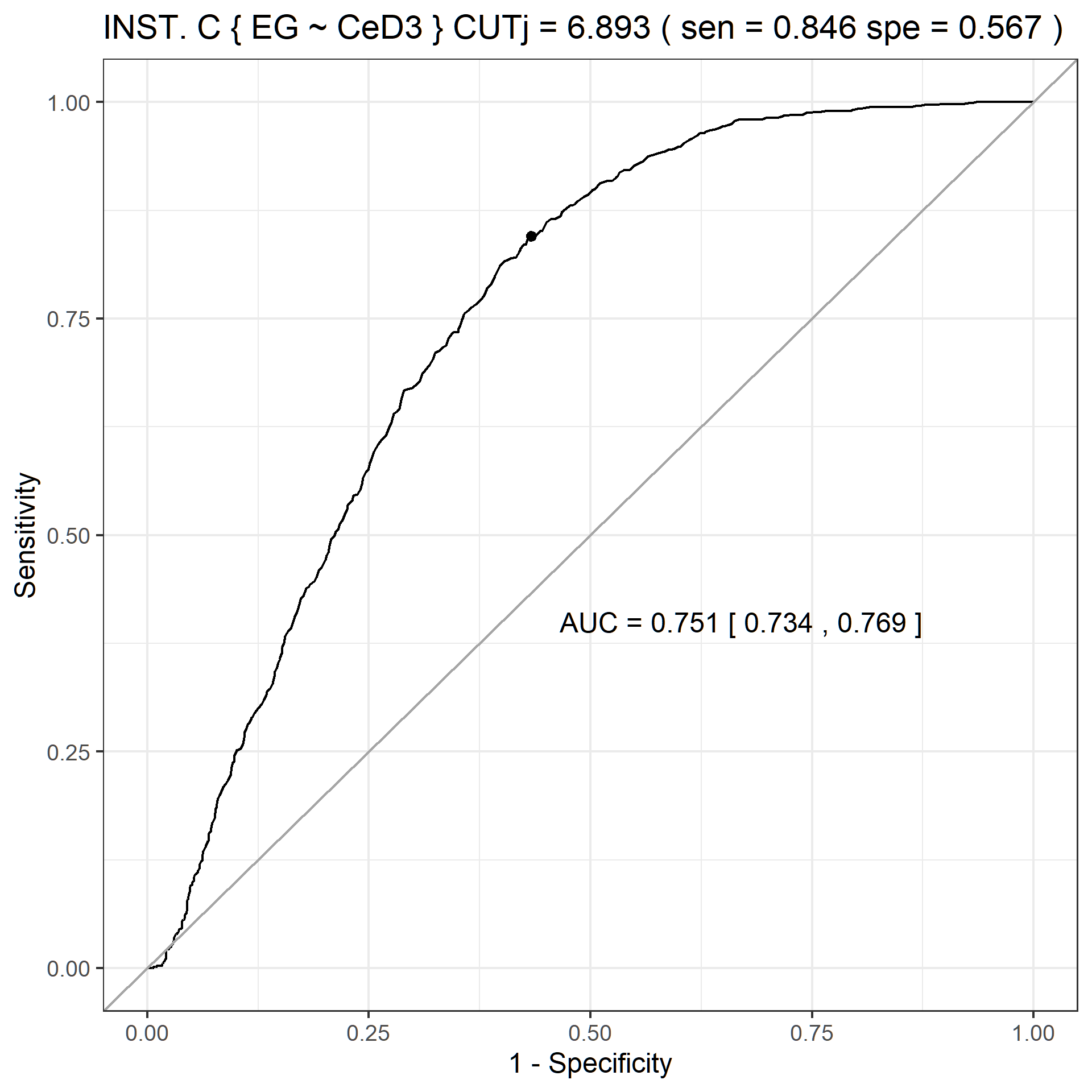

Supplement: Supplementary file 1 [file mmc1.zip › SupplementaryMaterials/238-ROCut.png]

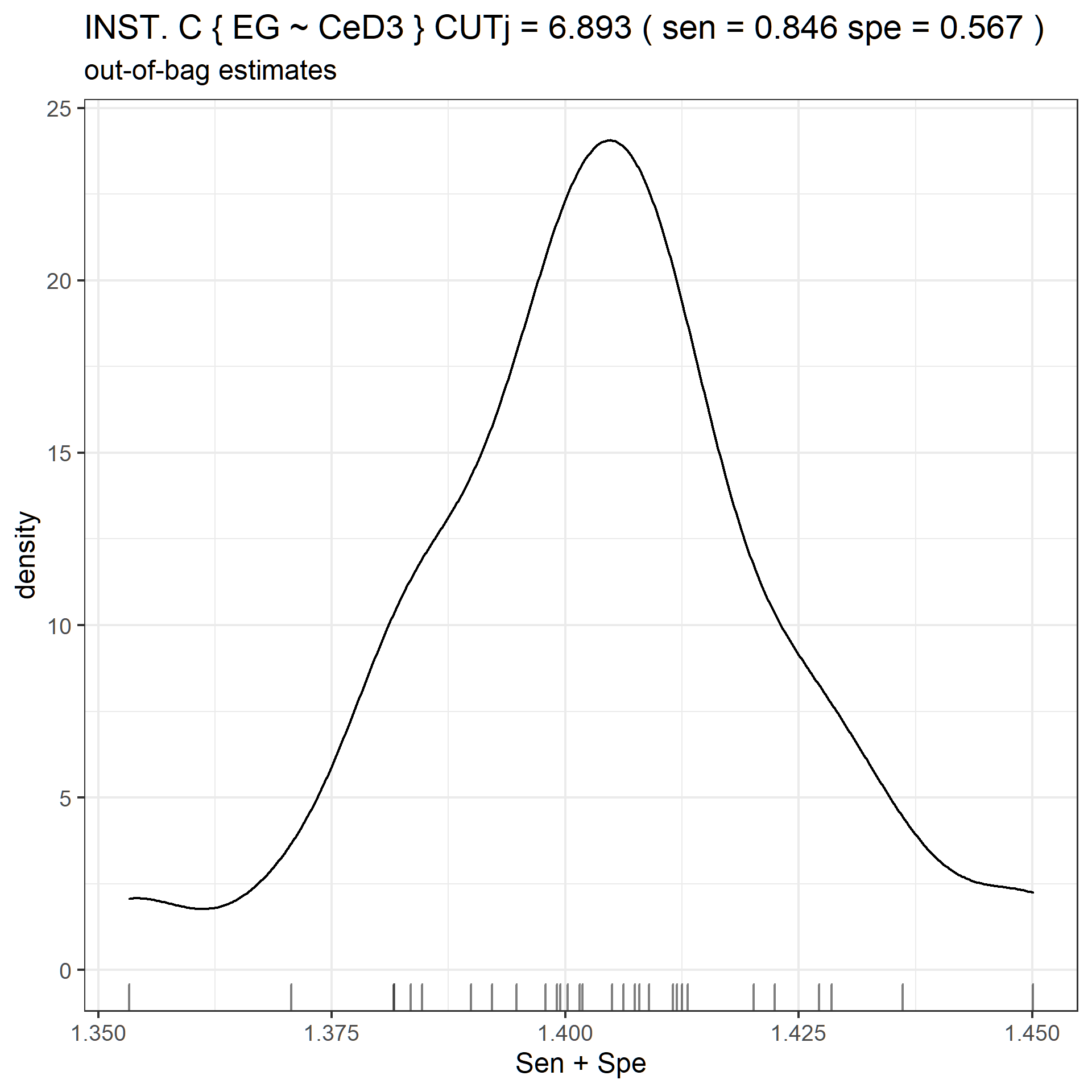

Supplement: Supplementary file 1 [file mmc1.zip › SupplementaryMaterials/238-SenSpe.png]

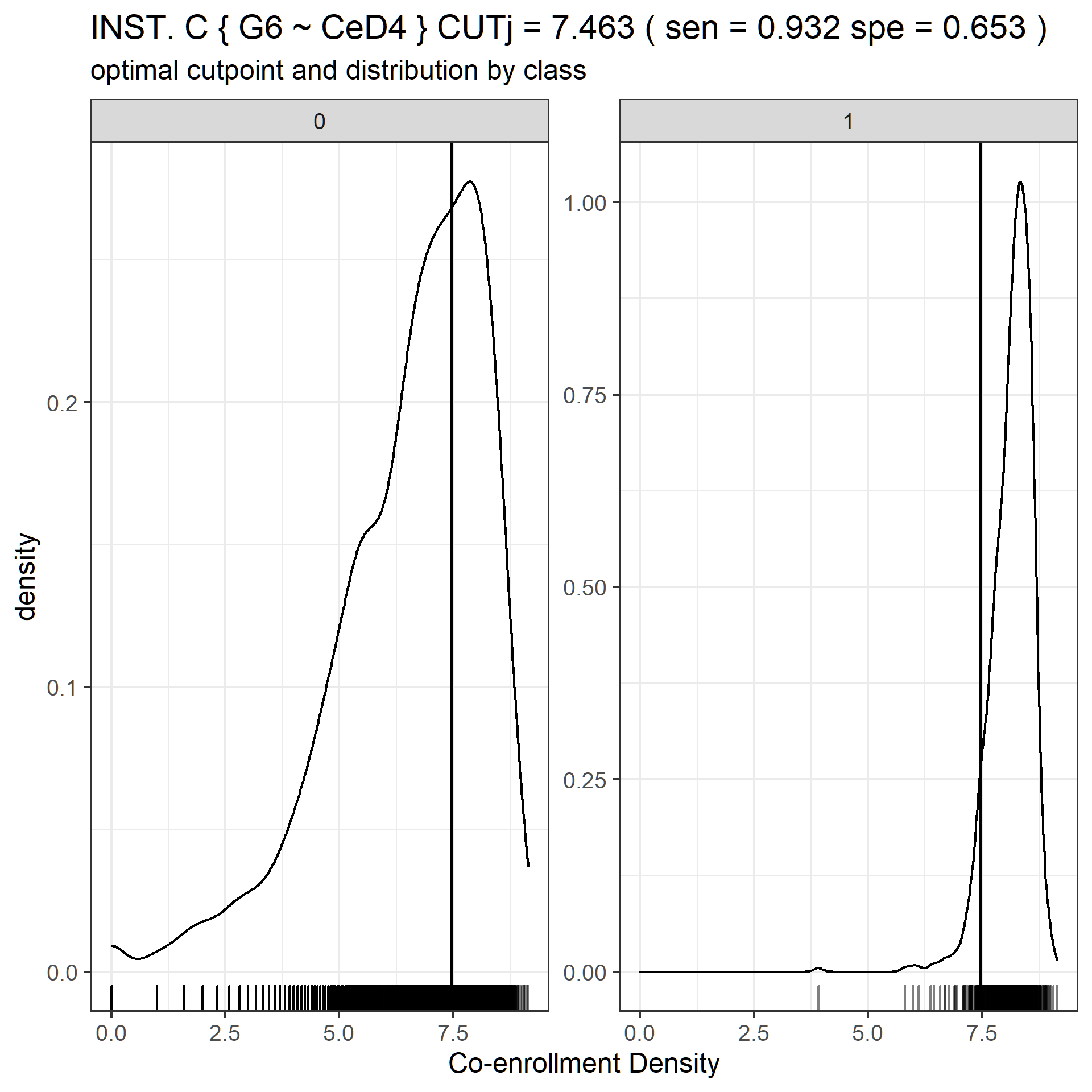

Supplement: Supplementary file 1 [file mmc1.zip › SupplementaryMaterials/247-ClassDen.png]

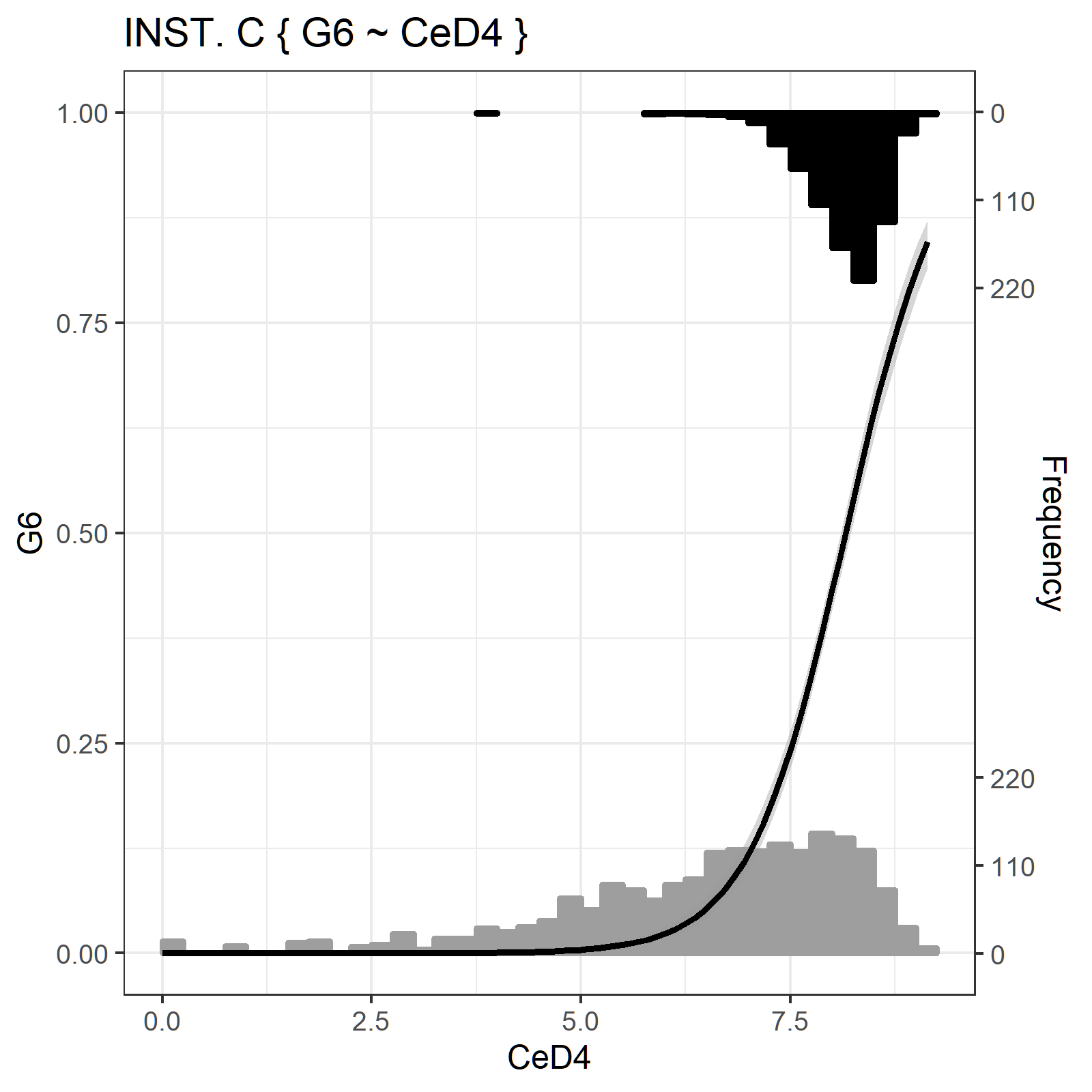

Supplement: Supplementary file 1 [file mmc1.zip › SupplementaryMaterials/247-LogitCurve.png]

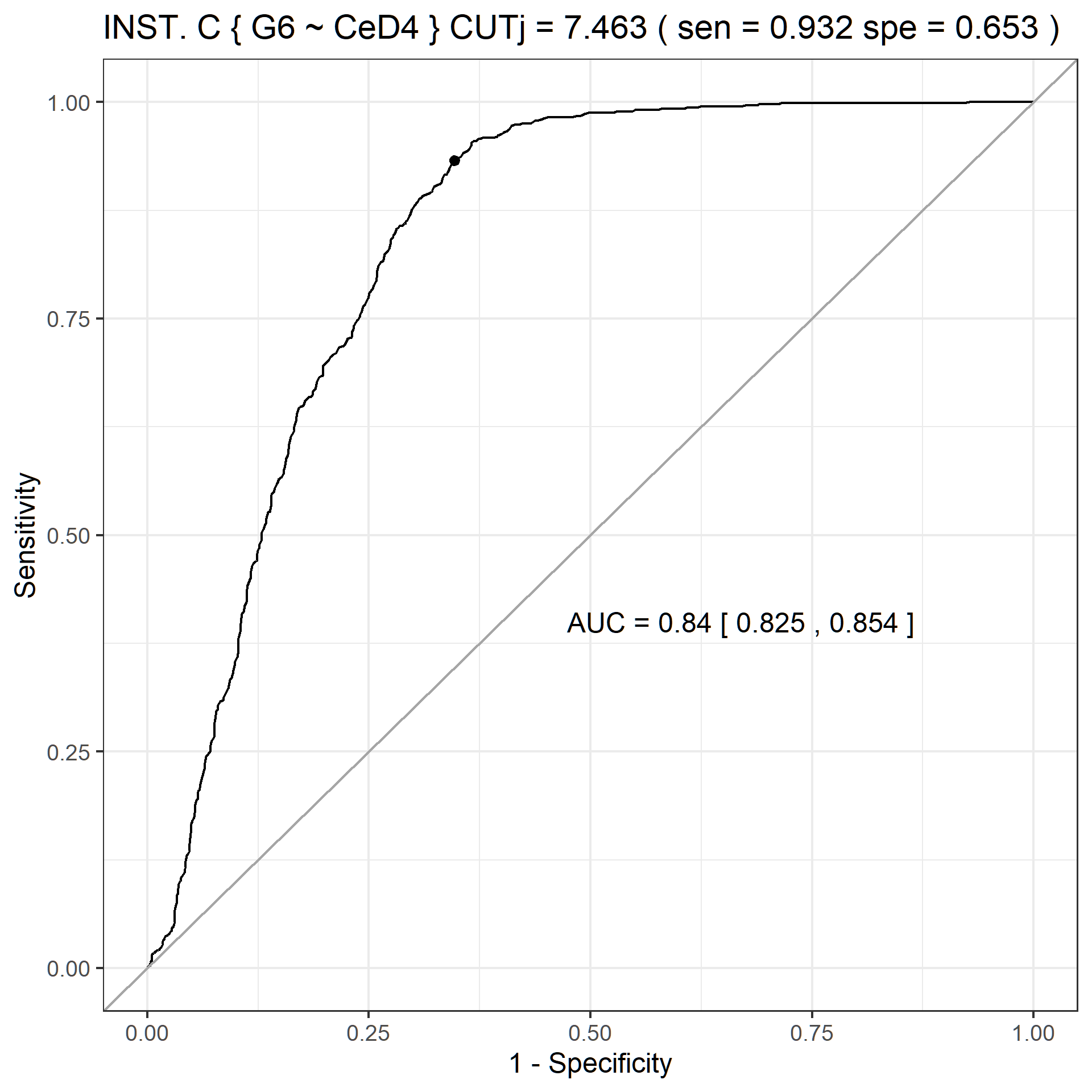

Supplement: Supplementary file 1 [file mmc1.zip › SupplementaryMaterials/247-ROCut.png]

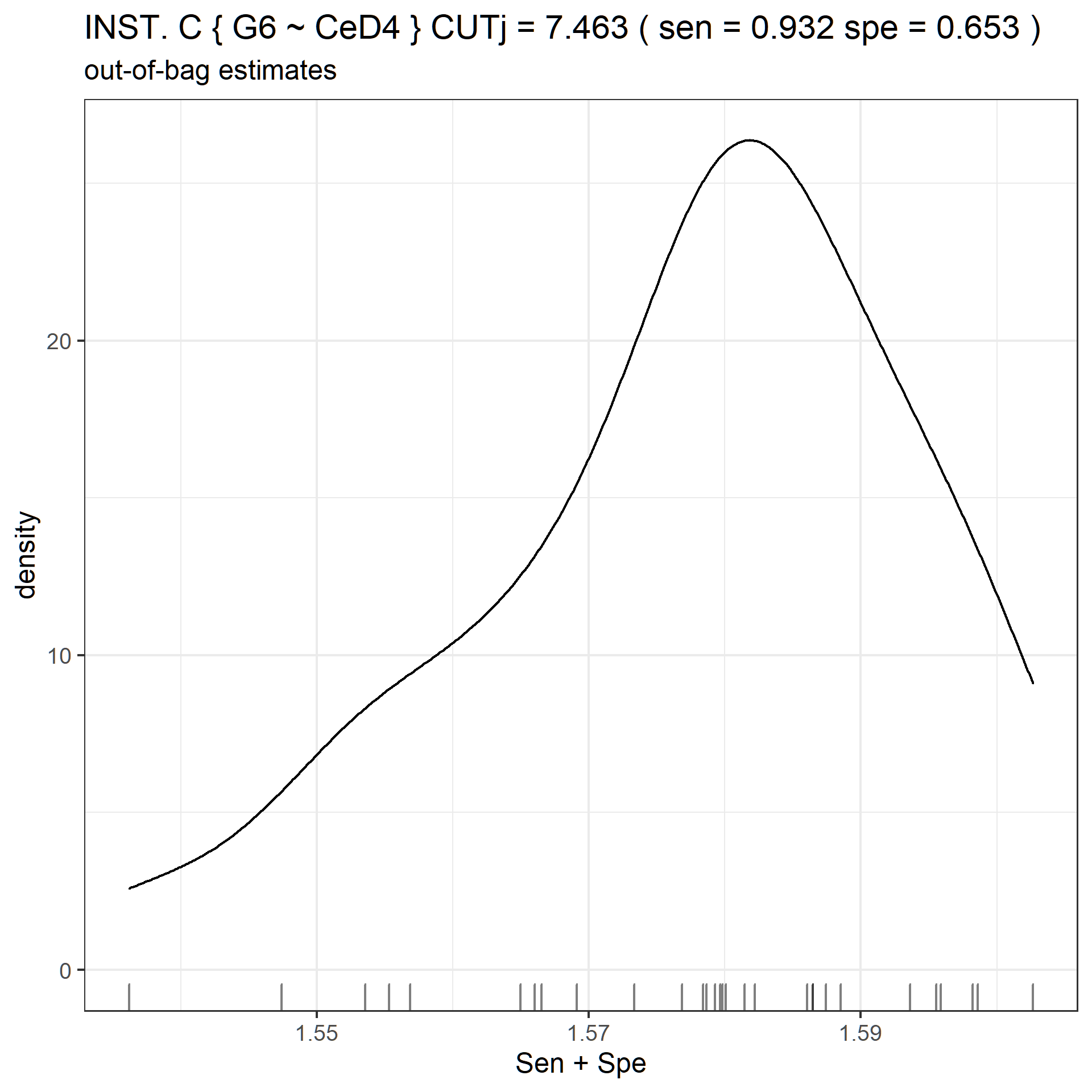

Supplement: Supplementary file 1 [file mmc1.zip › SupplementaryMaterials/247-SenSpe.png]

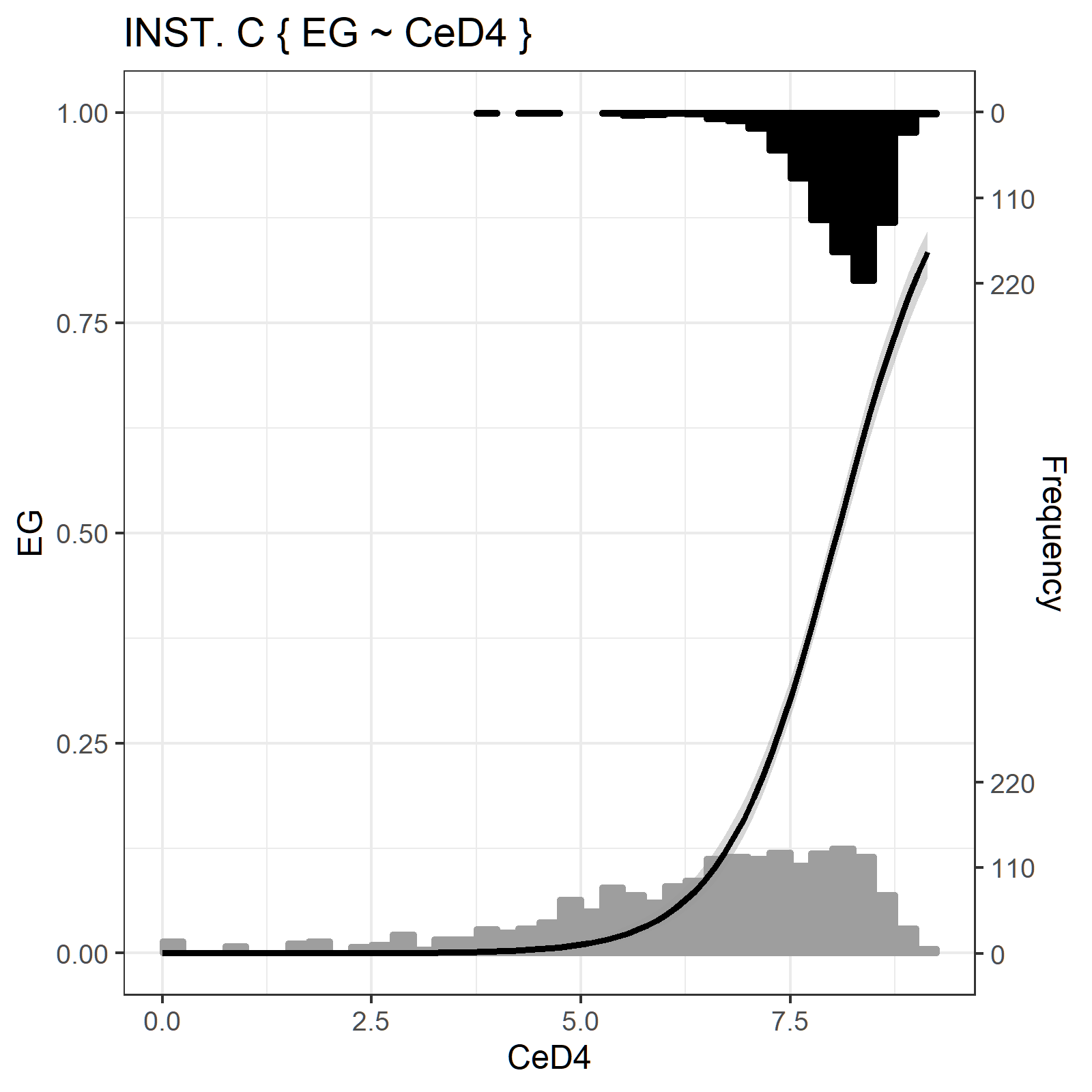

Supplement: Supplementary file 1 [file mmc1.zip › SupplementaryMaterials/248-LogitCurve.png]

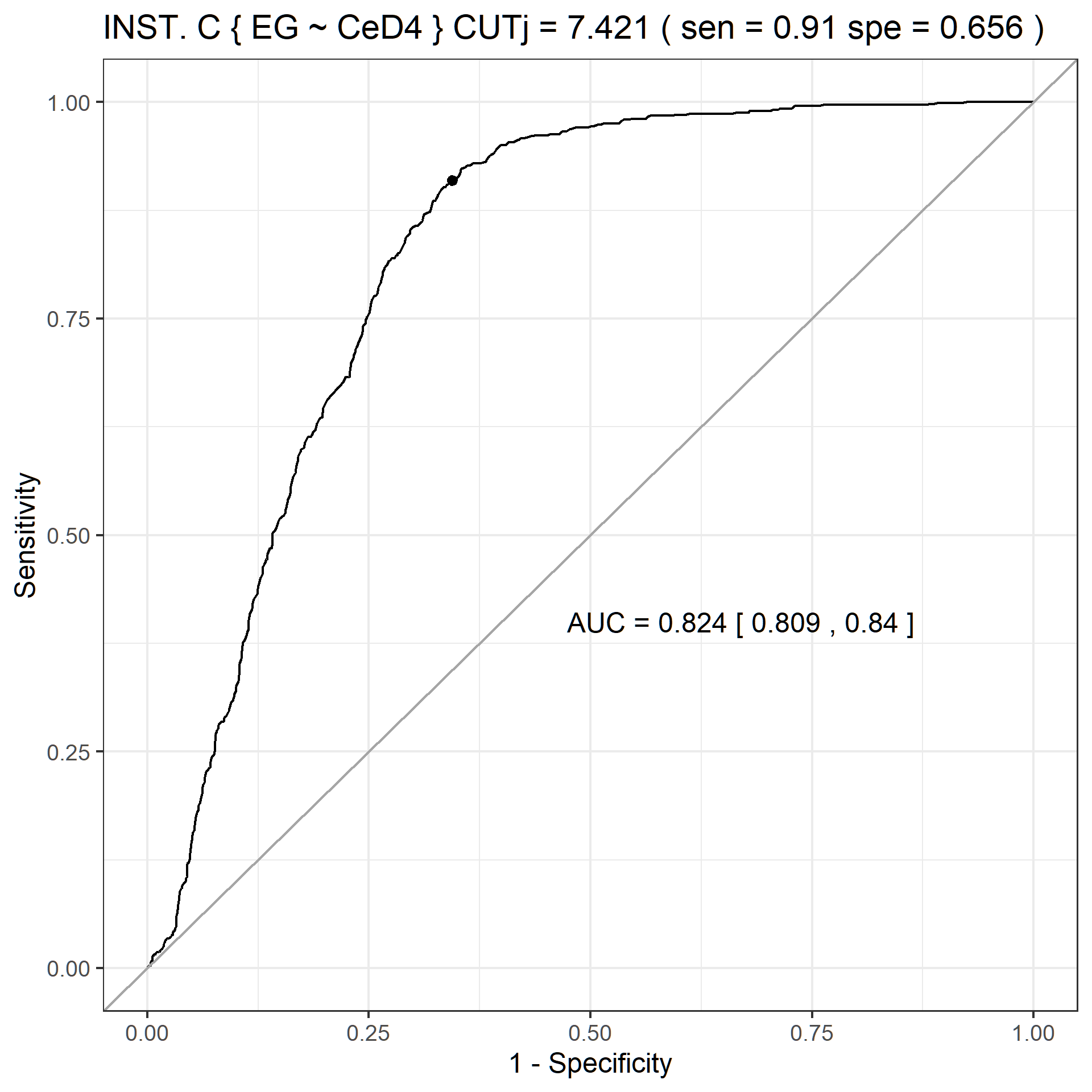

Supplement: Supplementary file 1 [file mmc1.zip › SupplementaryMaterials/248-ROCut.png]

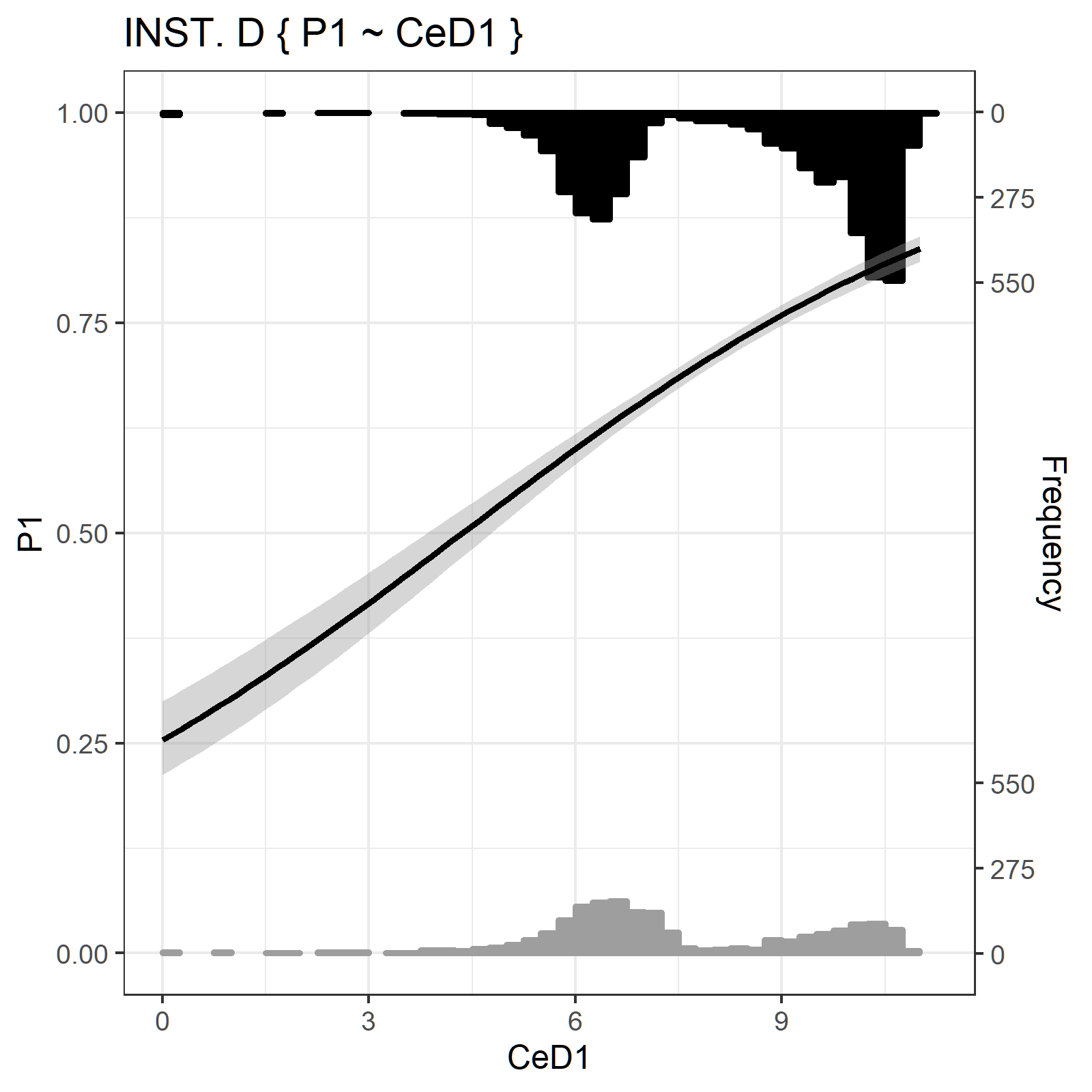

Supplement: Supplementary file 1 [file mmc1.zip › SupplementaryMaterials/315-LogitCurve.png]

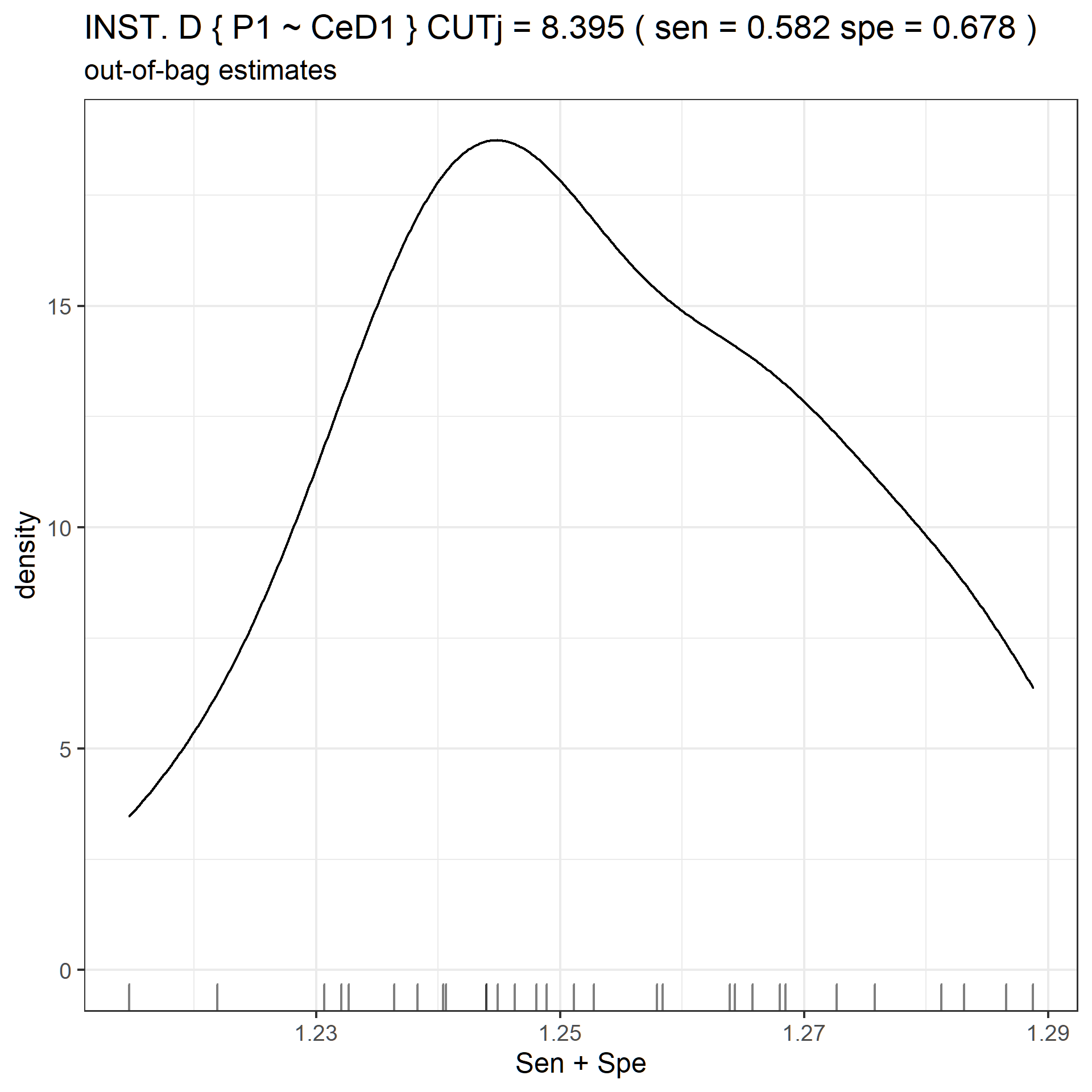

Supplement: Supplementary file 1 [file mmc1.zip › SupplementaryMaterials/315-SenSpe.png]

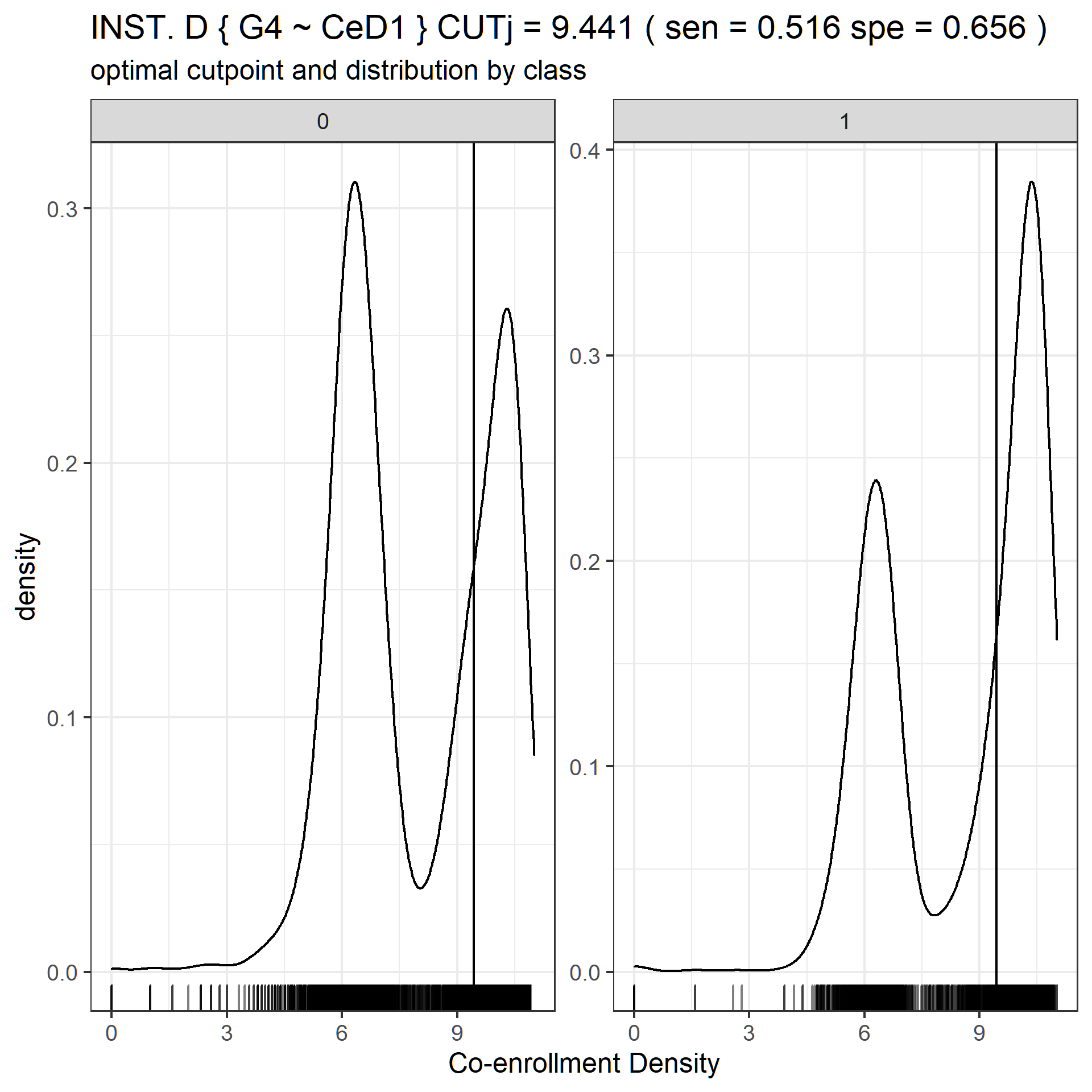

Supplement: Supplementary file 1 [file mmc1.zip › SupplementaryMaterials/316-ClassDen.png]

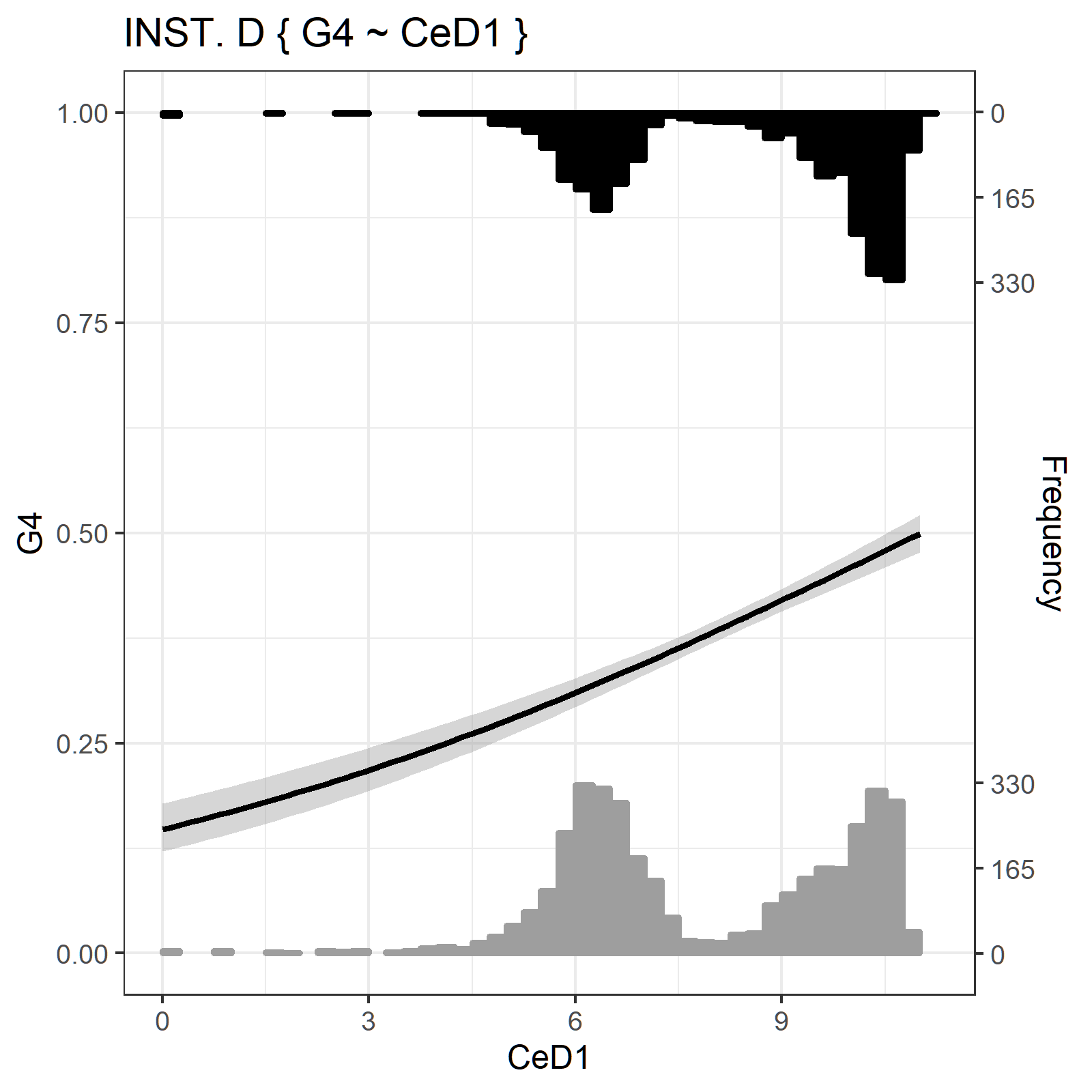

Supplement: Supplementary file 1 [file mmc1.zip › SupplementaryMaterials/316-LogitCurve.png]

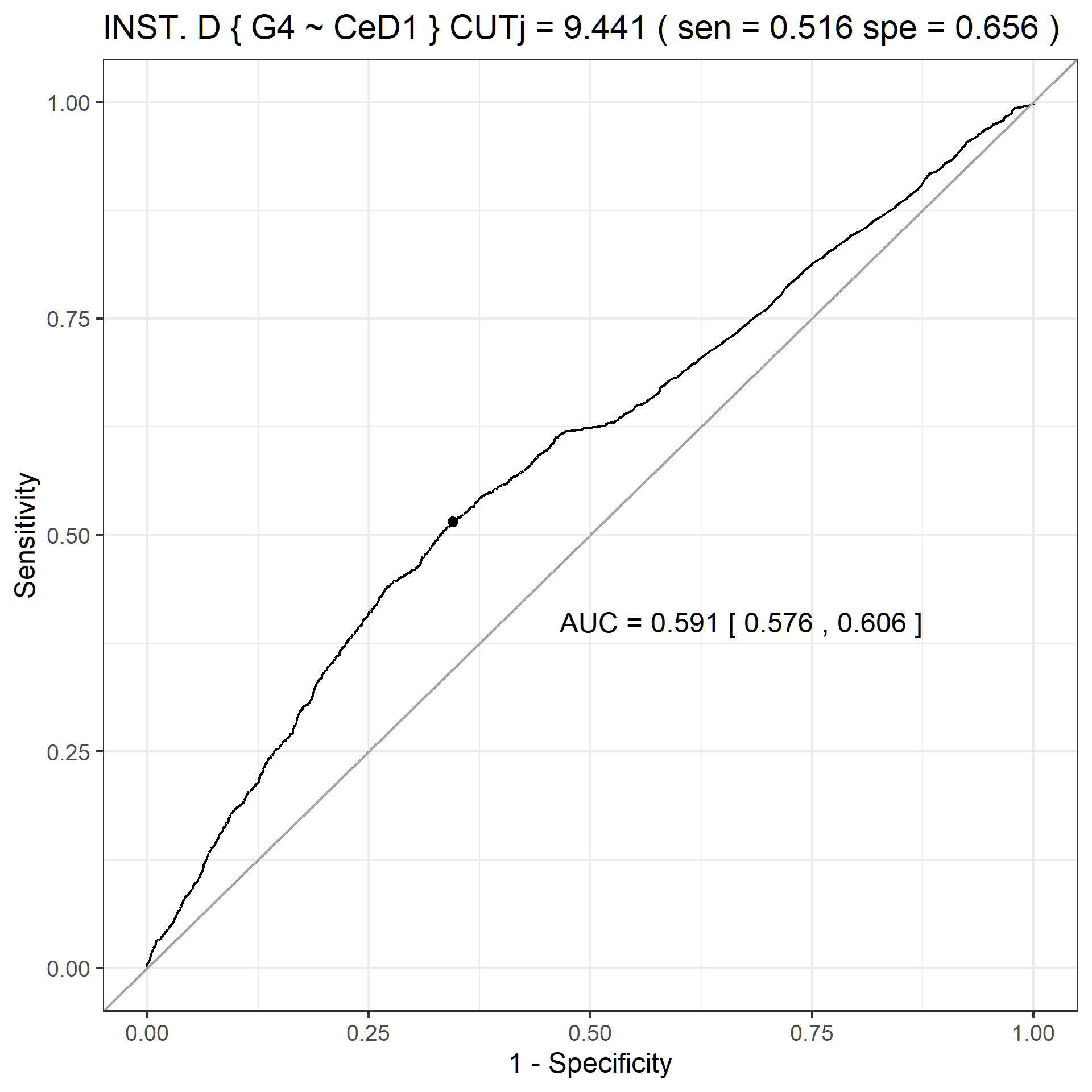

Supplement: Supplementary file 1 [file mmc1.zip › SupplementaryMaterials/316-ROCut.png]

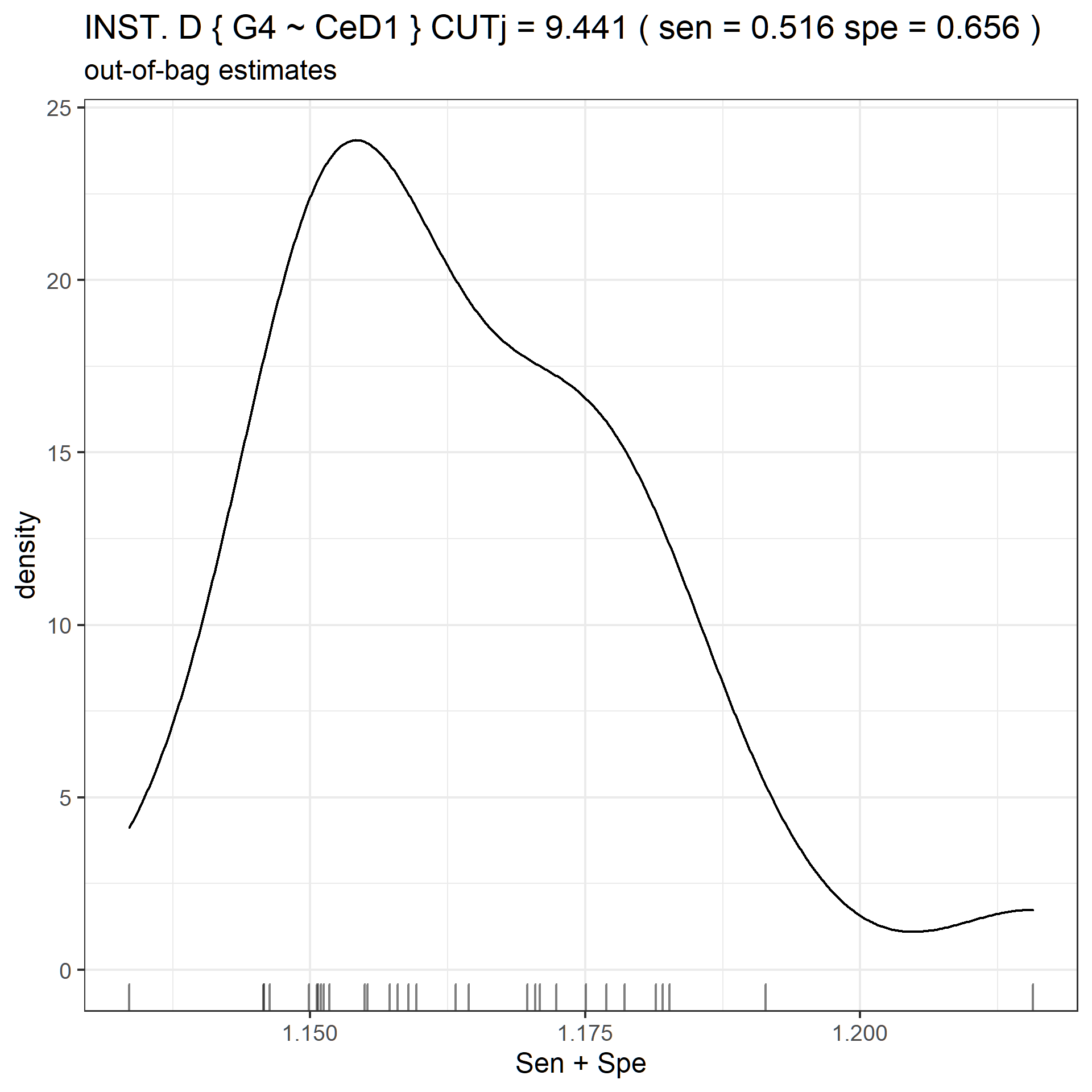

Supplement: Supplementary file 1 [file mmc1.zip › SupplementaryMaterials/316-SenSpe.png]

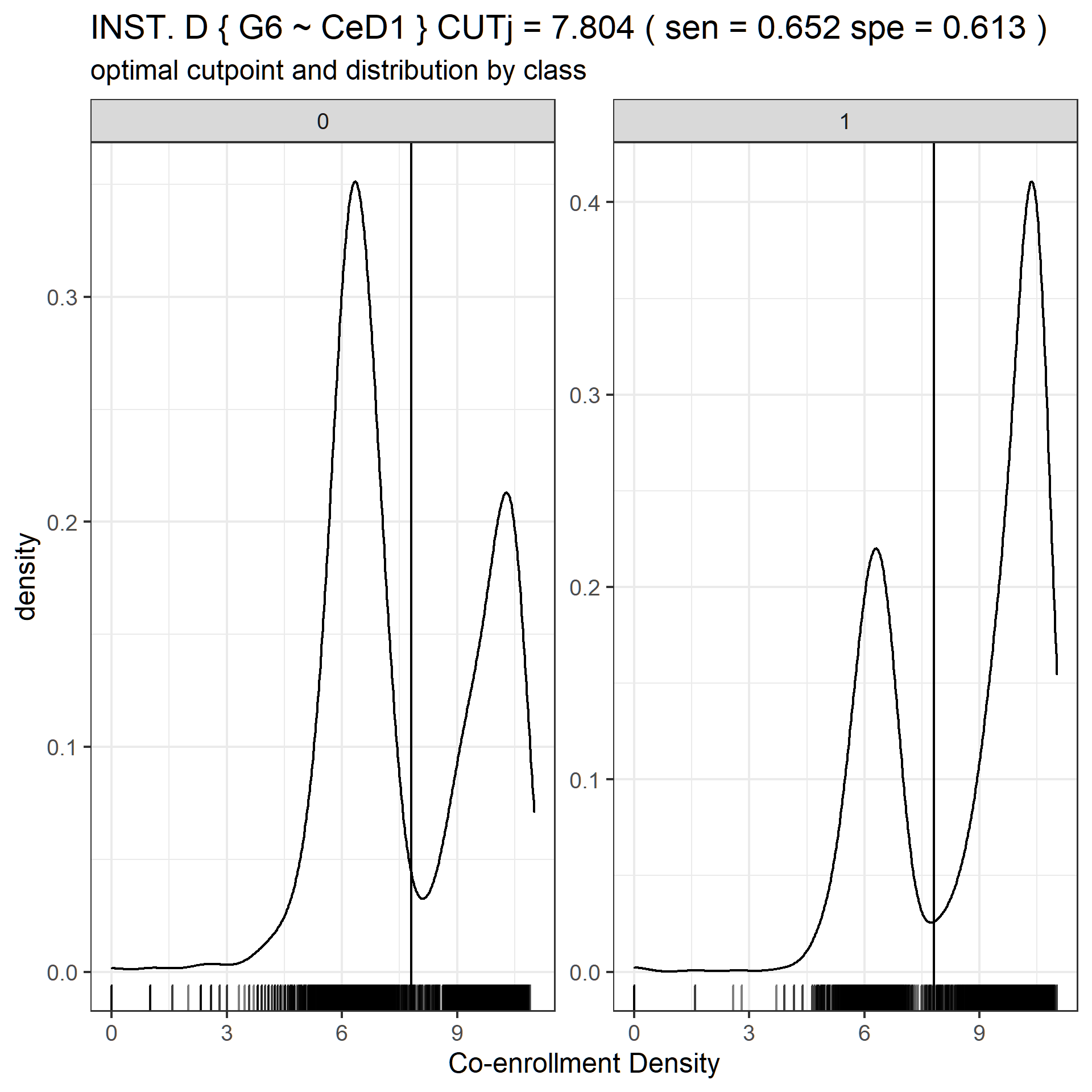

Supplement: Supplementary file 1 [file mmc1.zip › SupplementaryMaterials/317-ClassDen.png]

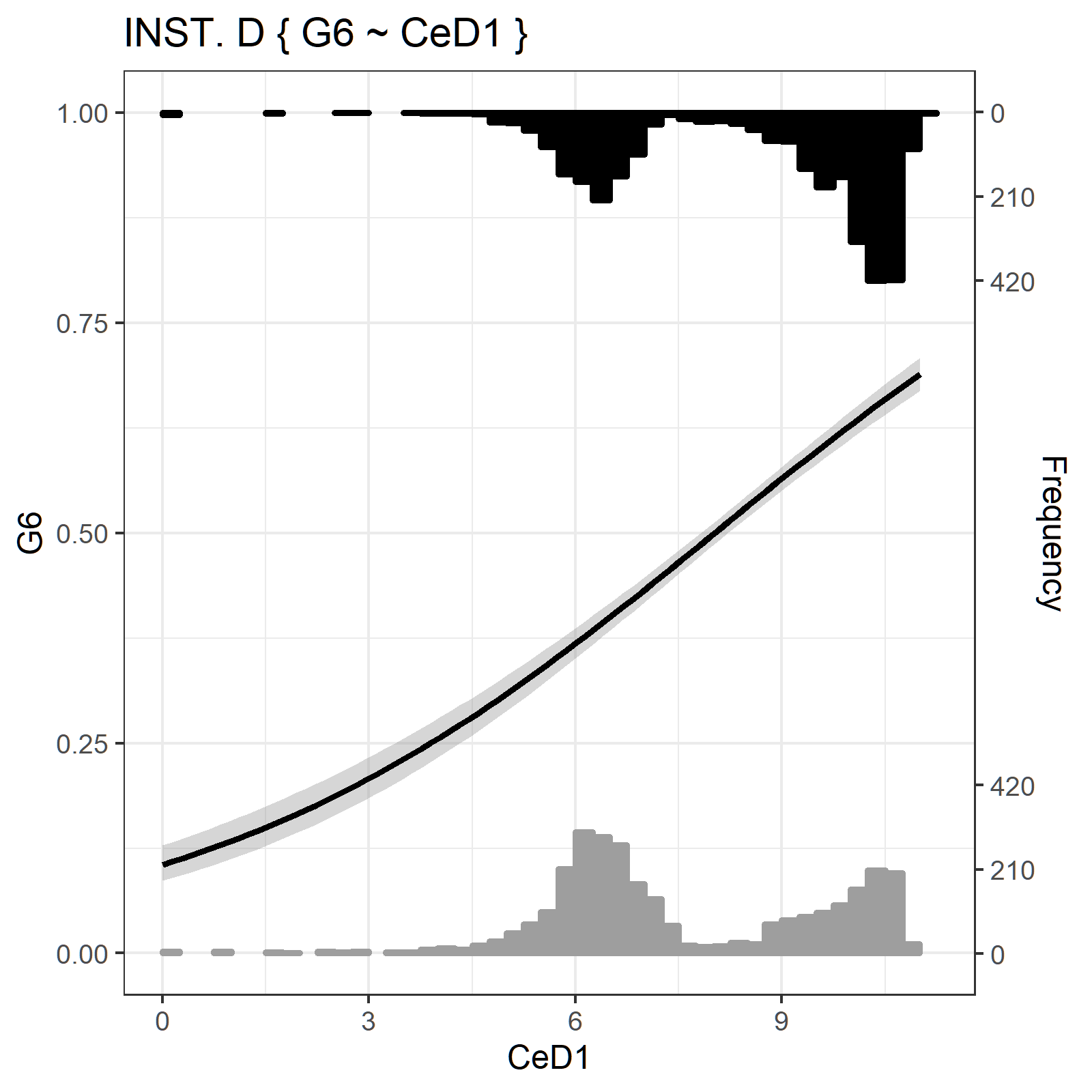

Supplement: Supplementary file 1 [file mmc1.zip › SupplementaryMaterials/317-LogitCurve.png]

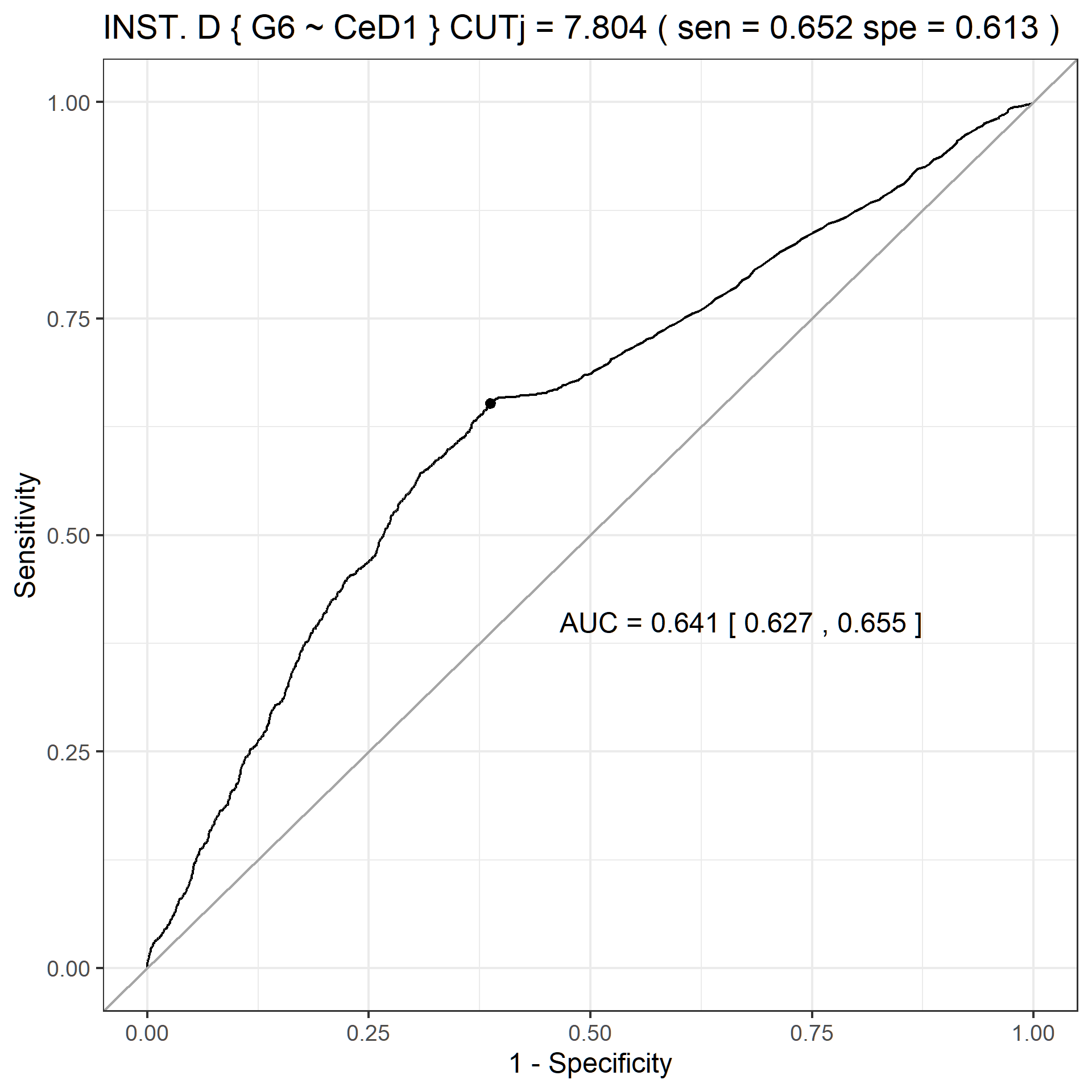

Supplement: Supplementary file 1 [file mmc1.zip › SupplementaryMaterials/317-ROCut.png]

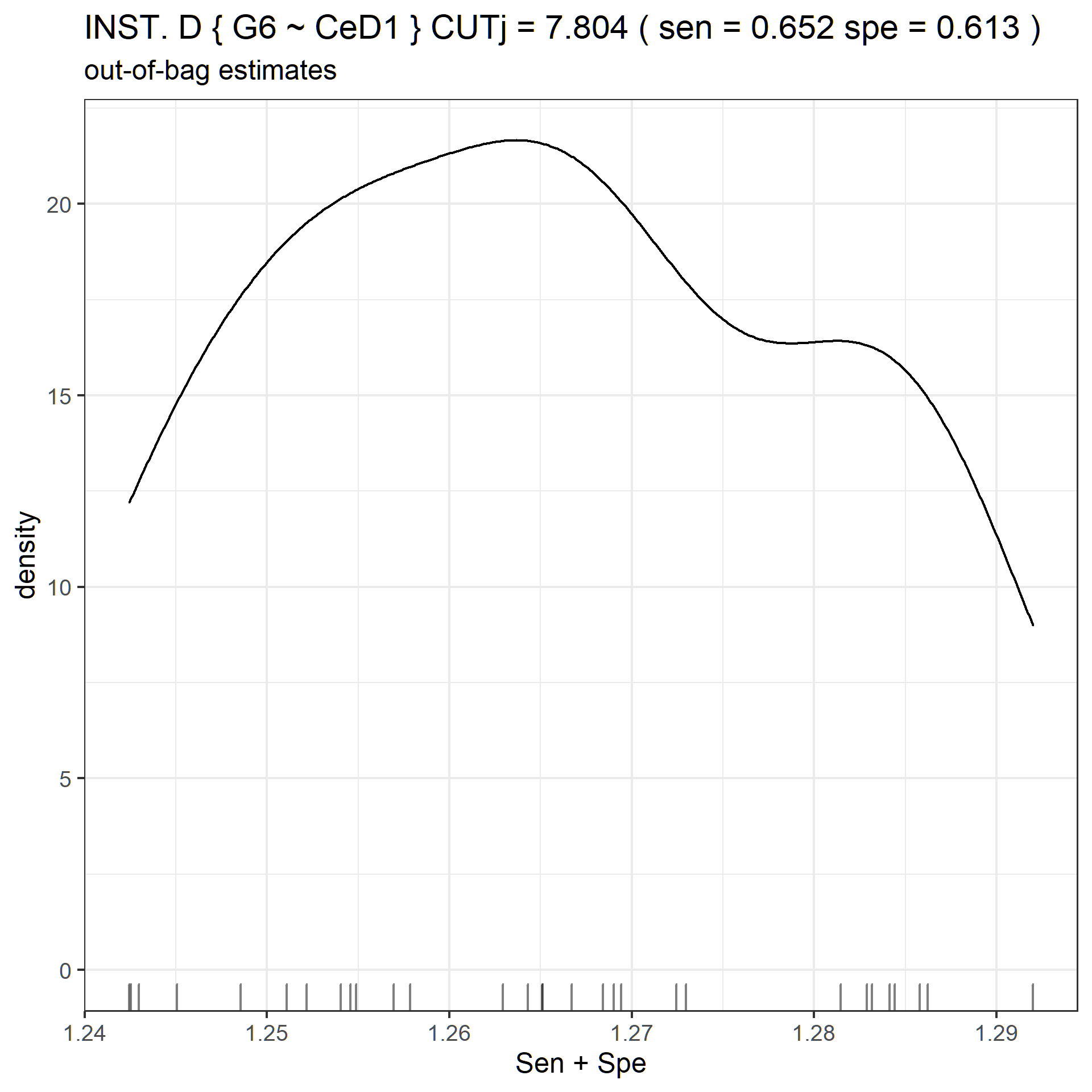

Supplement: Supplementary file 1 [file mmc1.zip › SupplementaryMaterials/317-SenSpe.png]

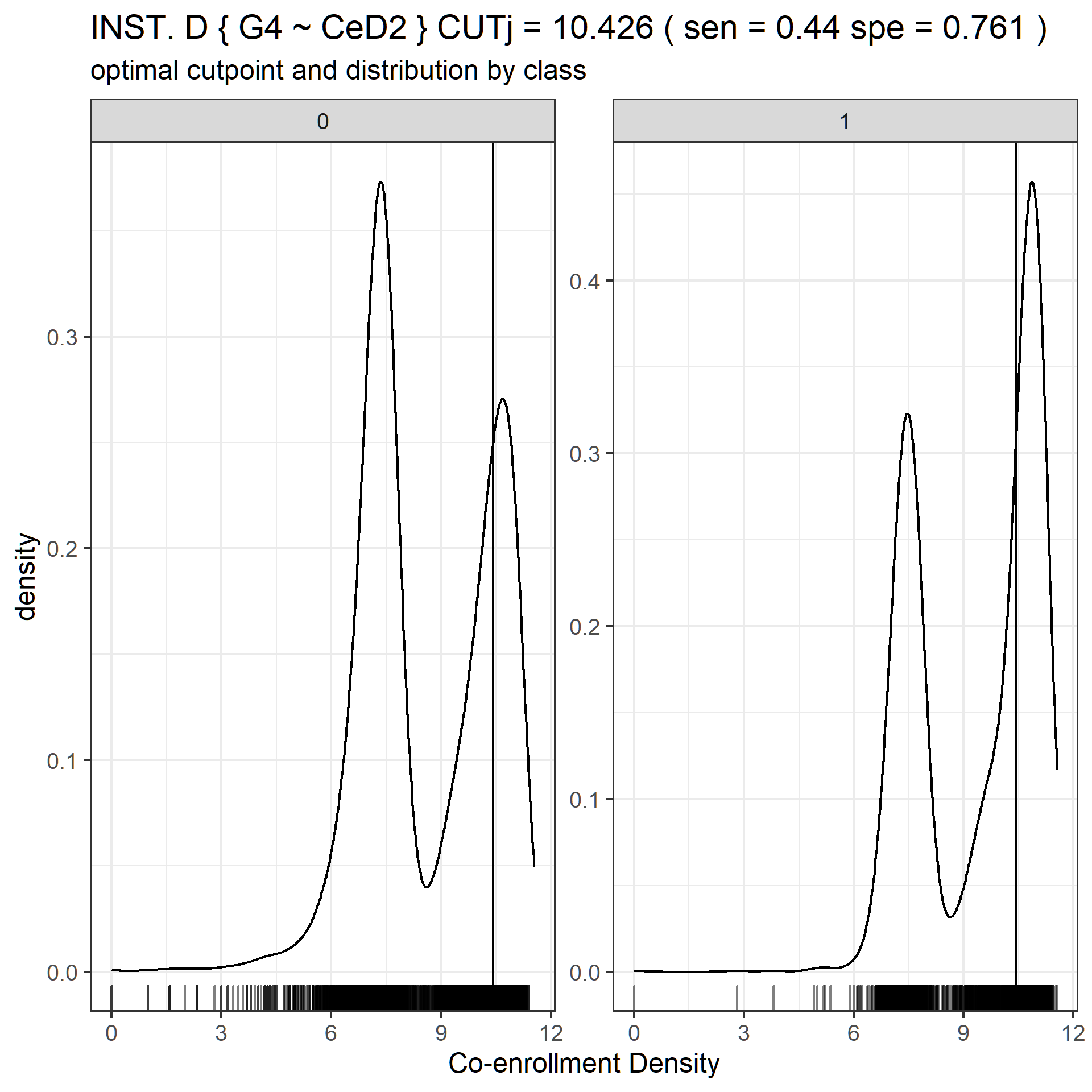

Supplement: Supplementary file 1 [file mmc1.zip › SupplementaryMaterials/326-ClassDen.png]

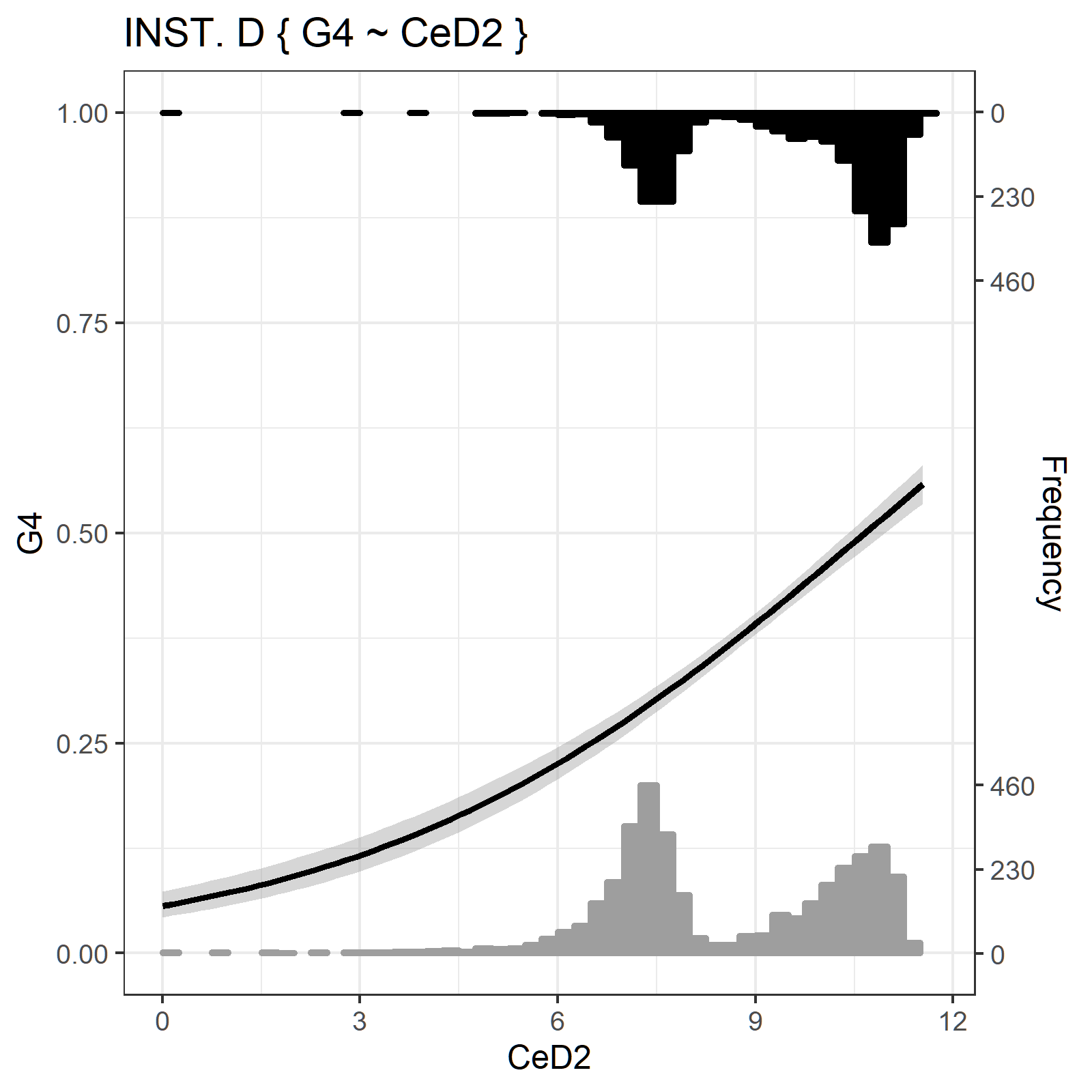

Supplement: Supplementary file 1 [file mmc1.zip › SupplementaryMaterials/326-LogitCurve.png]

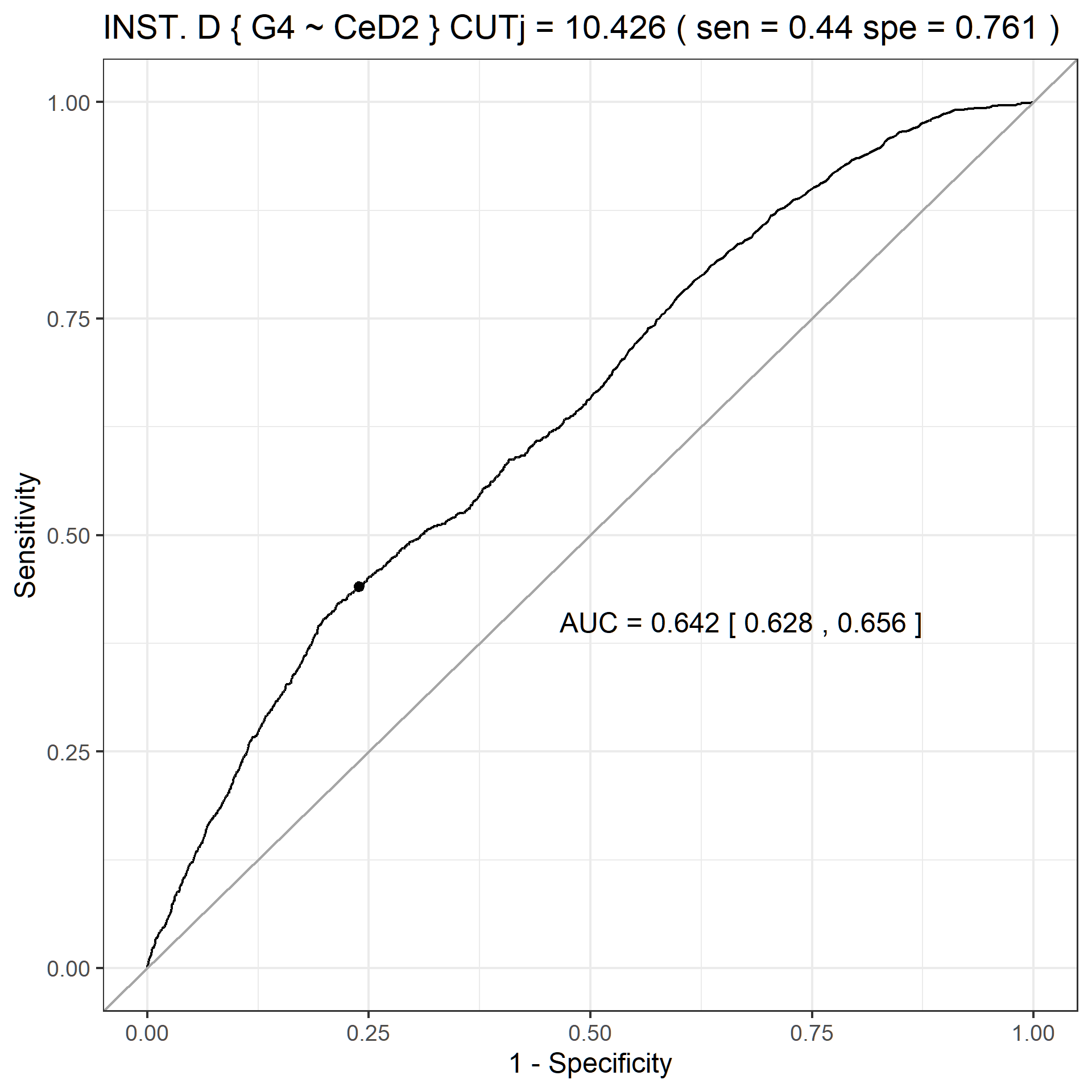

Supplement: Supplementary file 1 [file mmc1.zip › SupplementaryMaterials/326-ROCut.png]

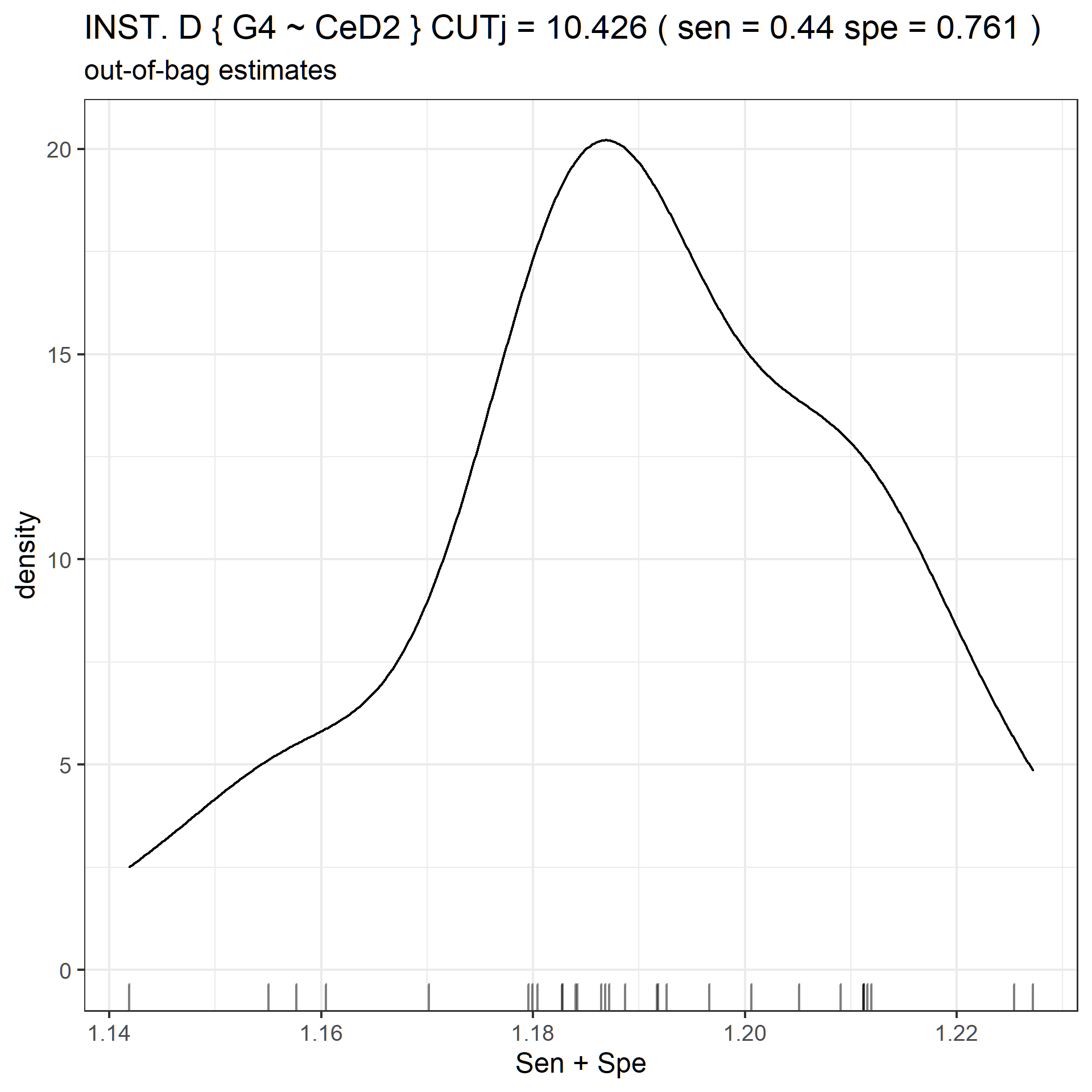

Supplement: Supplementary file 1 [file mmc1.zip › SupplementaryMaterials/326-SenSpe.png]

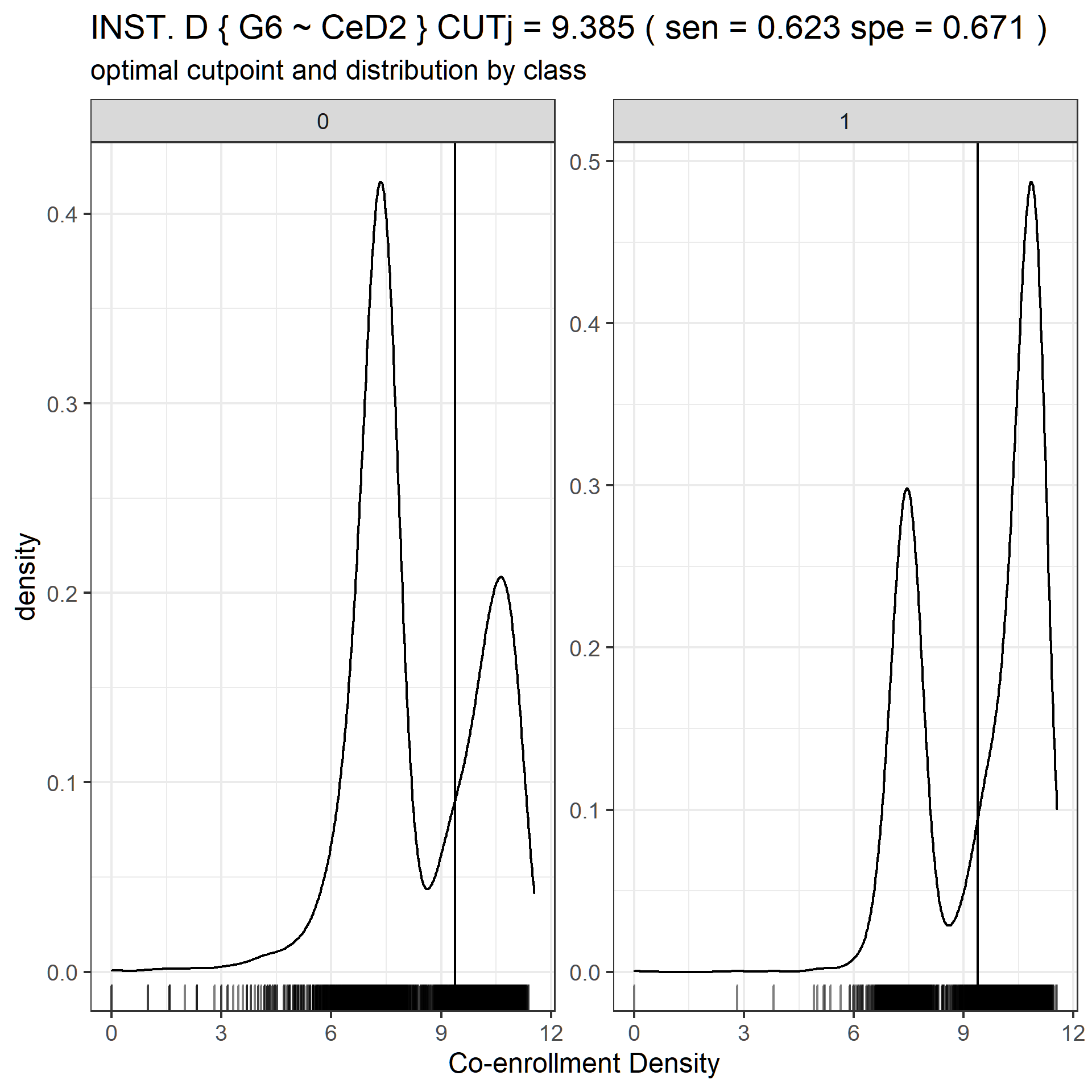

Supplement: Supplementary file 1 [file mmc1.zip › SupplementaryMaterials/327-ClassDen.png]

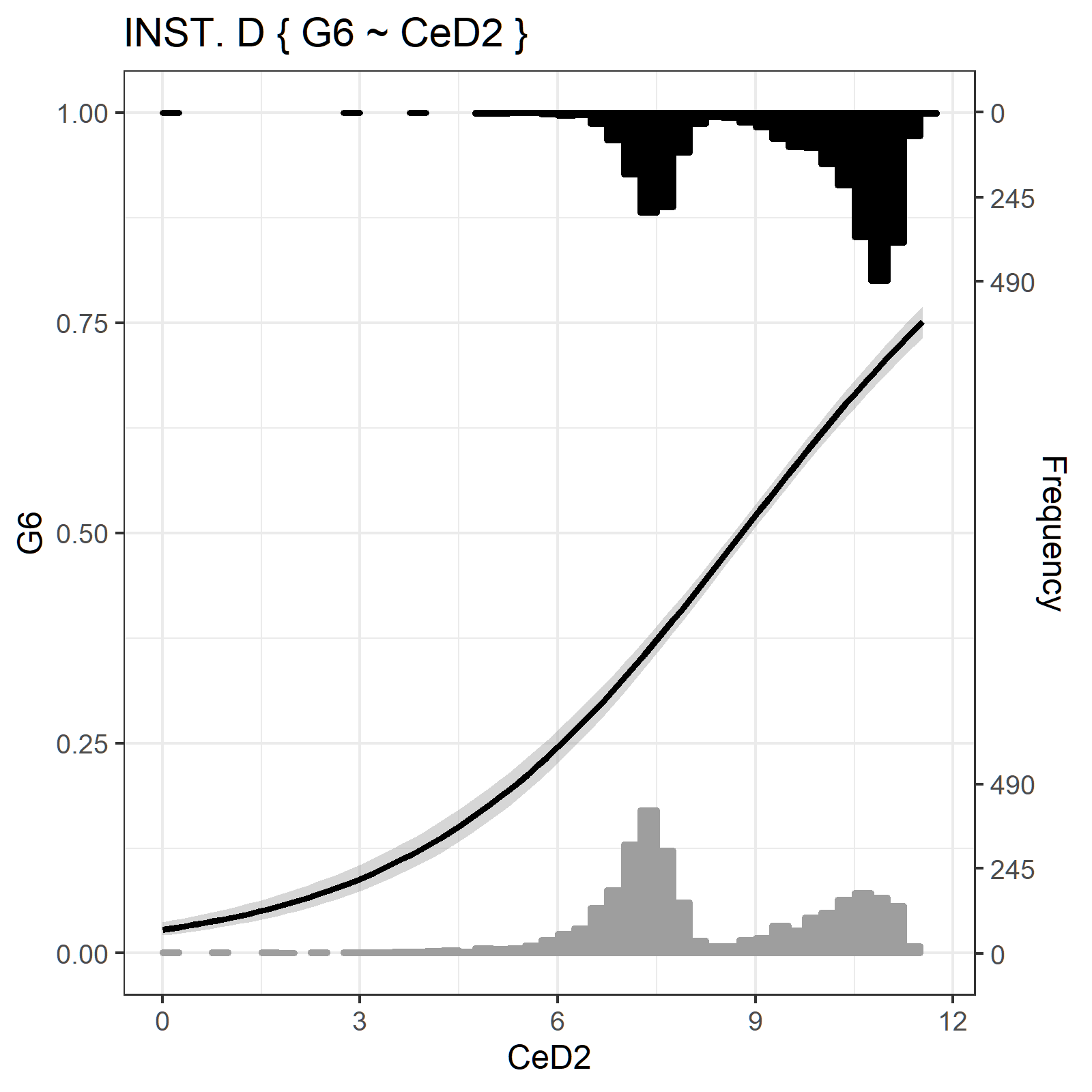

Supplement: Supplementary file 1 [file mmc1.zip › SupplementaryMaterials/327-LogitCurve.png]

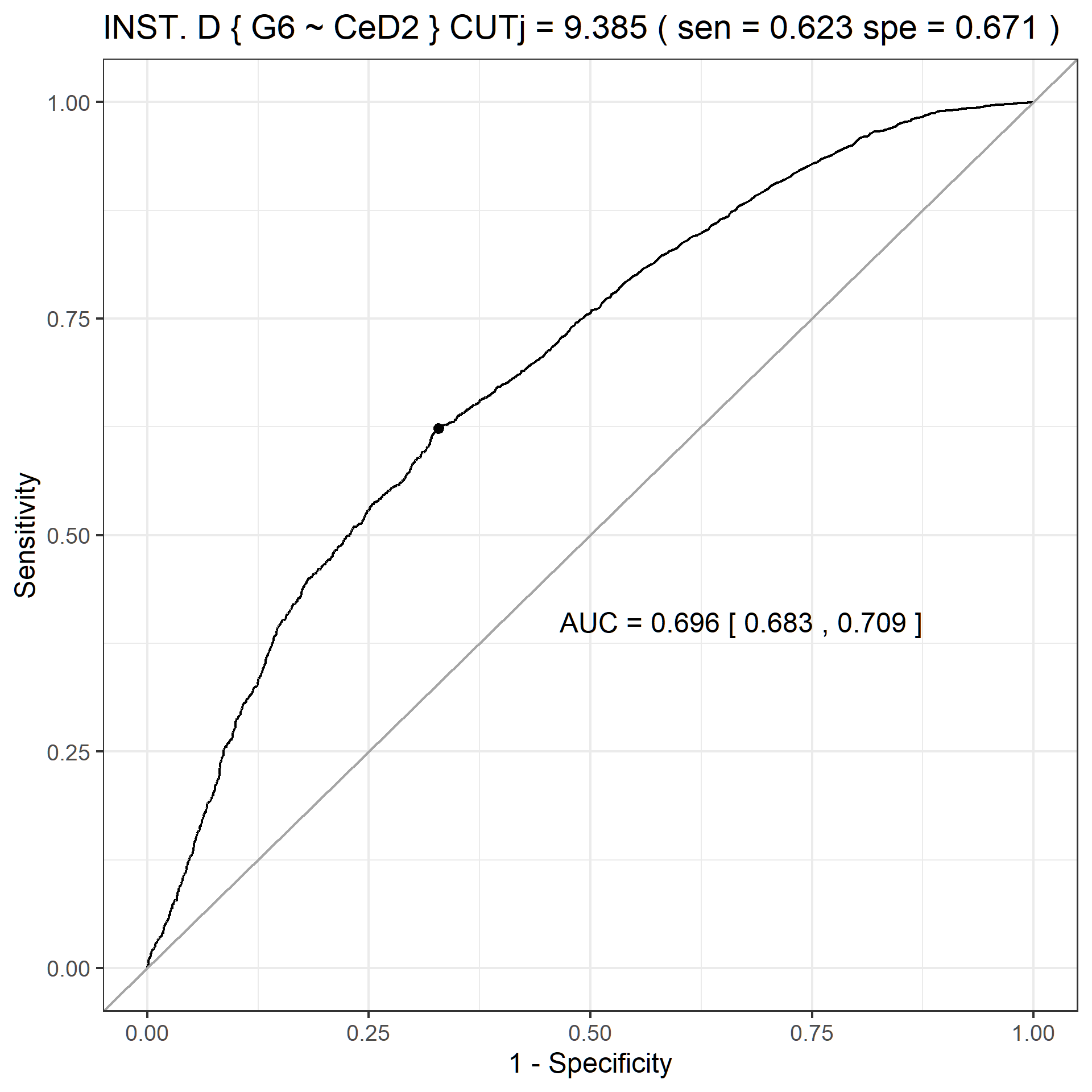

Supplement: Supplementary file 1 [file mmc1.zip › SupplementaryMaterials/327-ROCut.png]

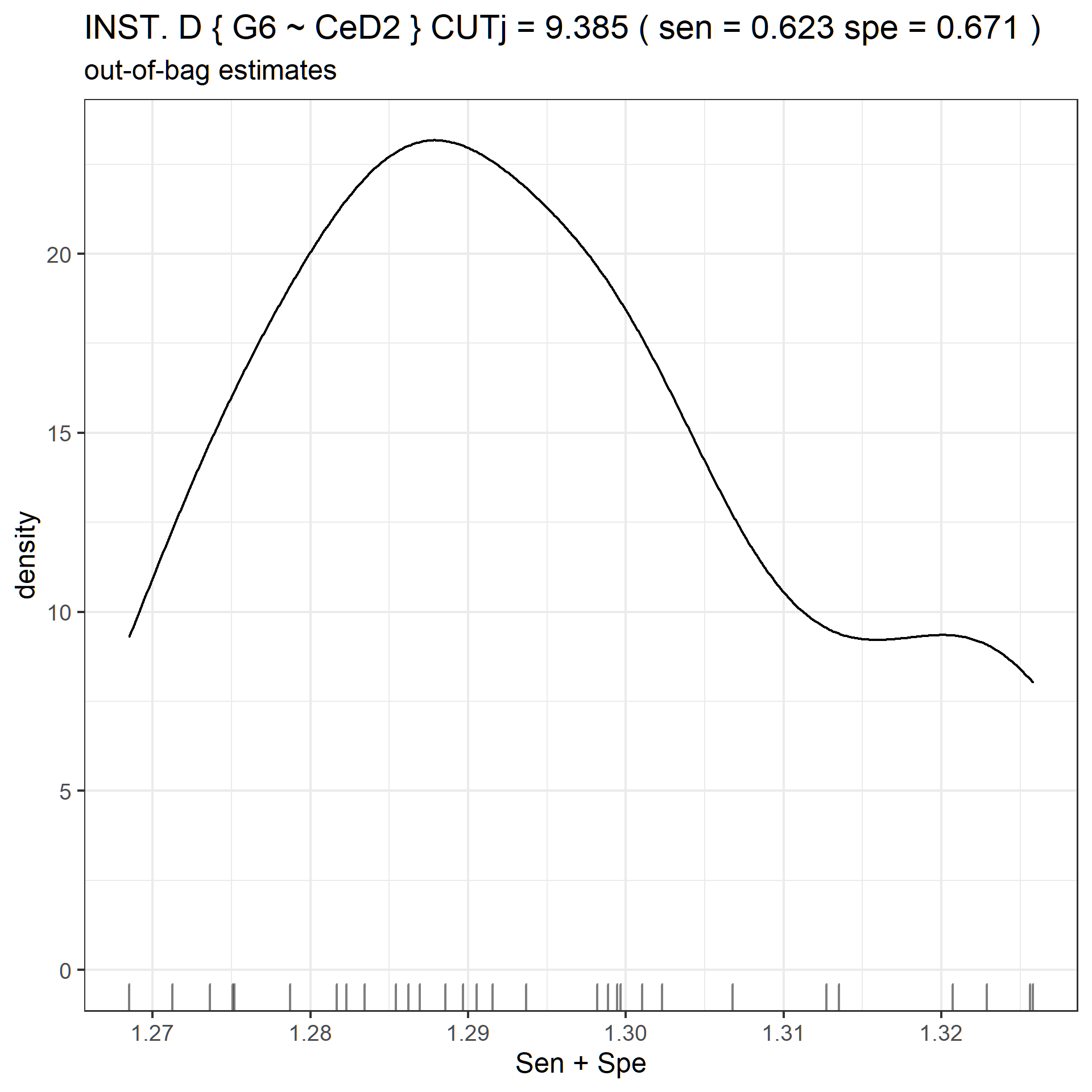

Supplement: Supplementary file 1 [file mmc1.zip › SupplementaryMaterials/327-SenSpe.png]

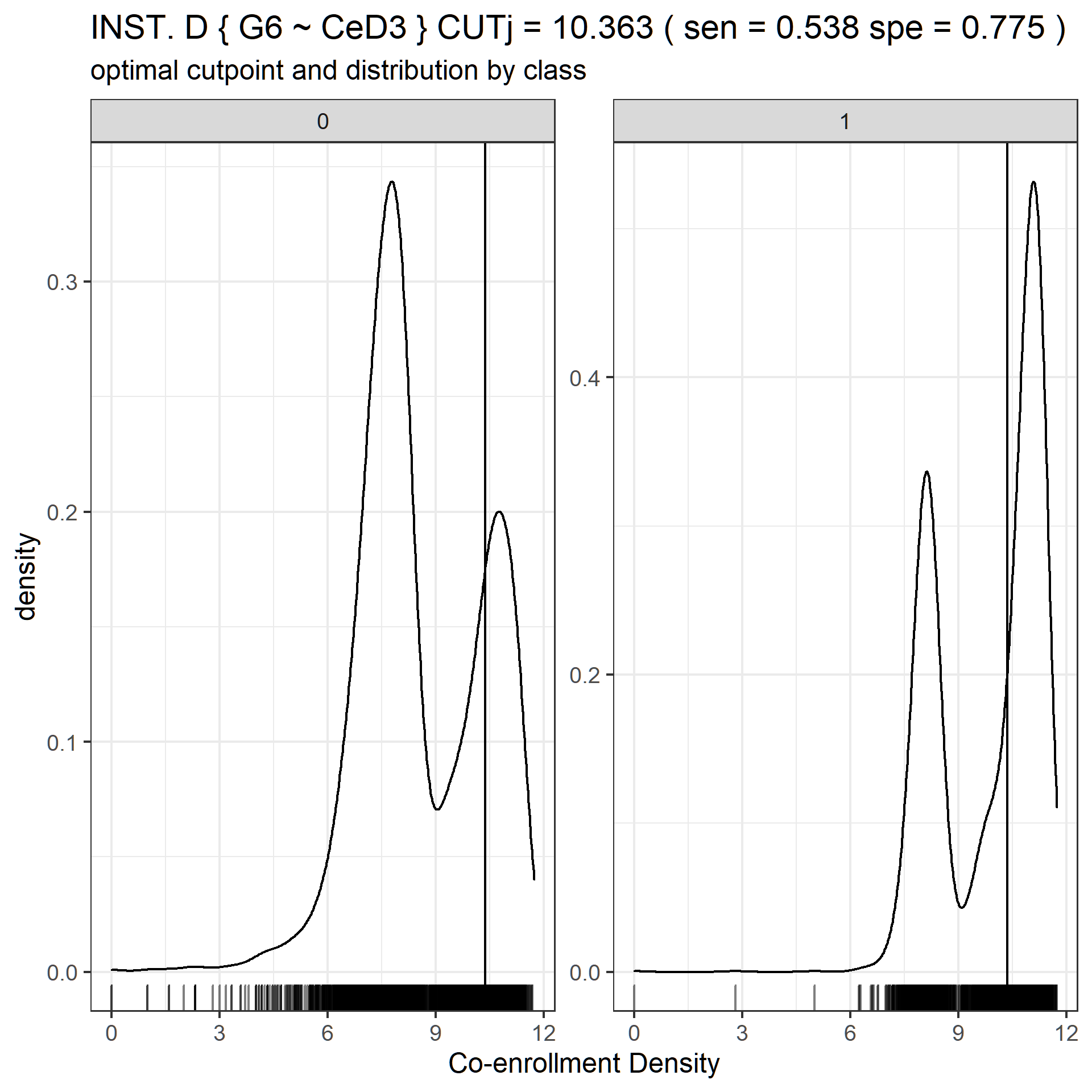

Supplement: Supplementary file 1 [file mmc1.zip › SupplementaryMaterials/337-ClassDen.png]

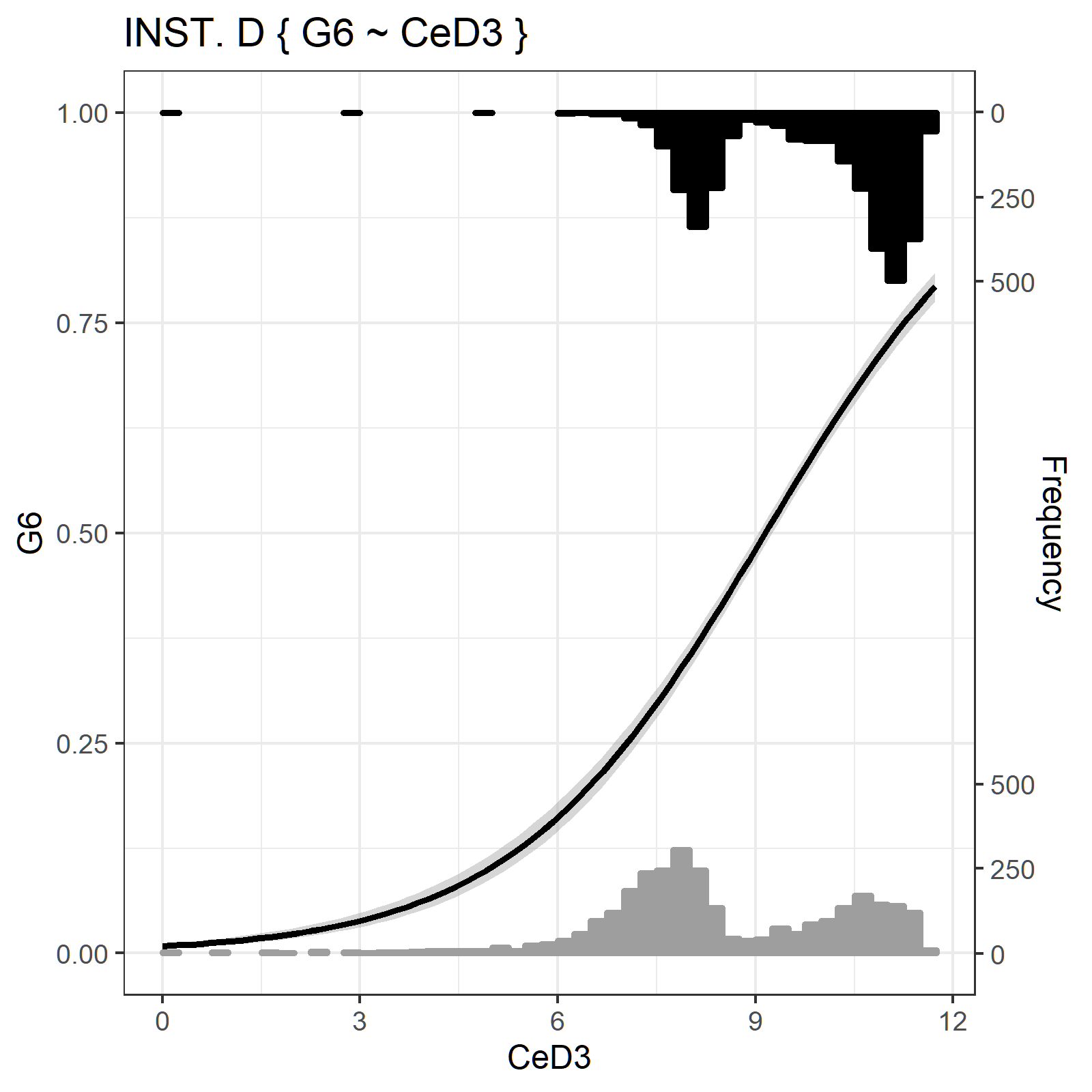

Supplement: Supplementary file 1 [file mmc1.zip › SupplementaryMaterials/337-LogitCurve.png]

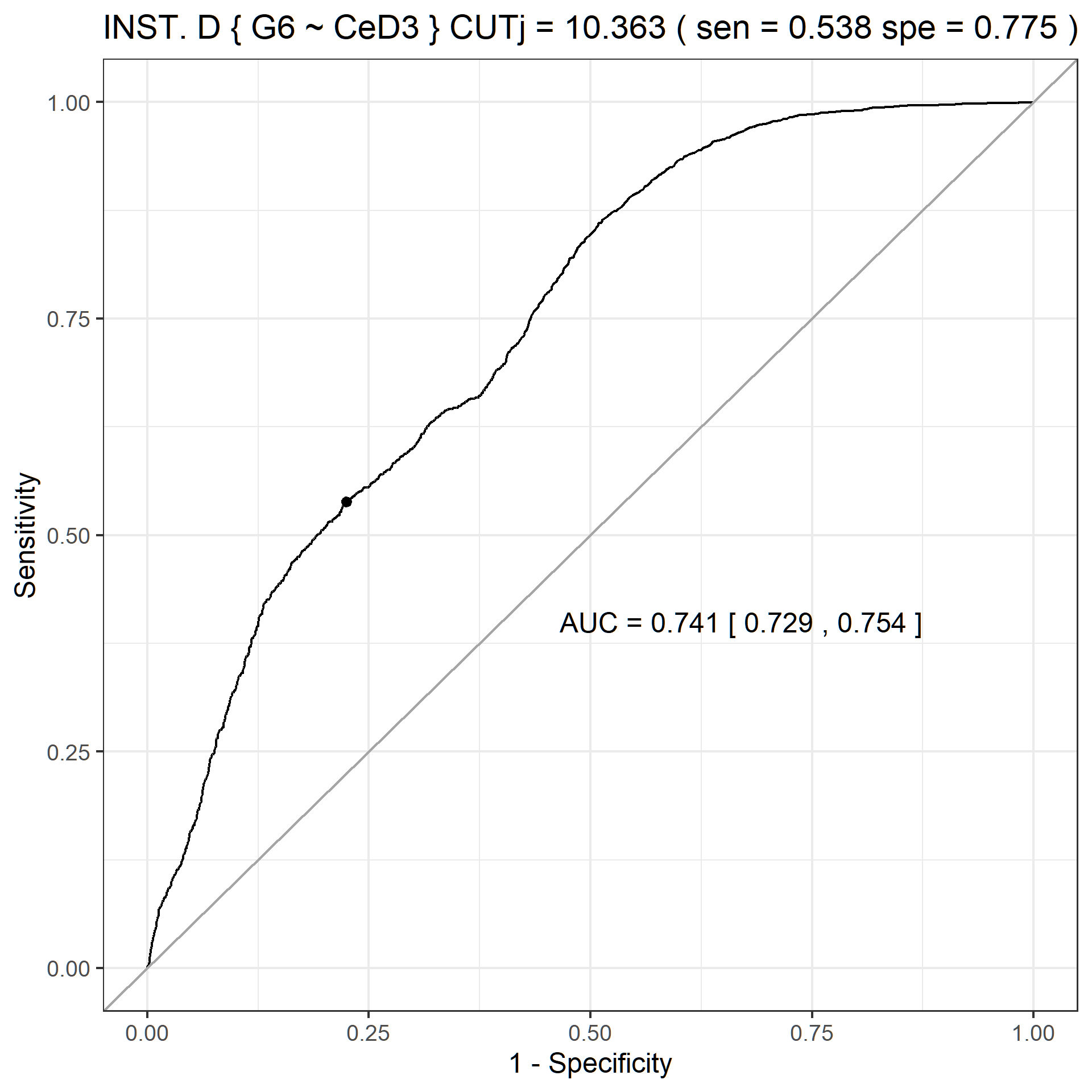

Supplement: Supplementary file 1 [file mmc1.zip › SupplementaryMaterials/337-ROCut.png]

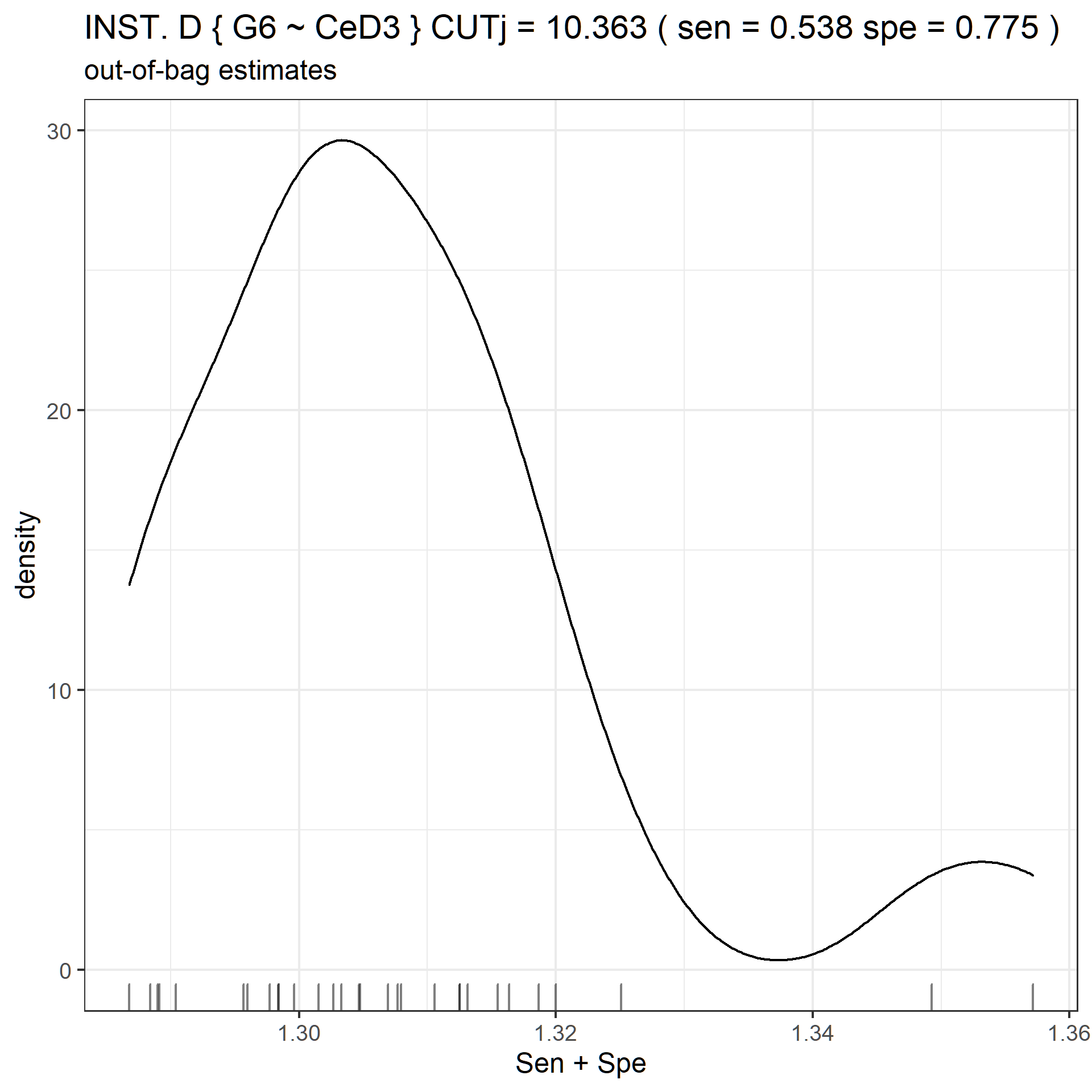

Supplement: Supplementary file 1 [file mmc1.zip › SupplementaryMaterials/337-SenSpe.png]

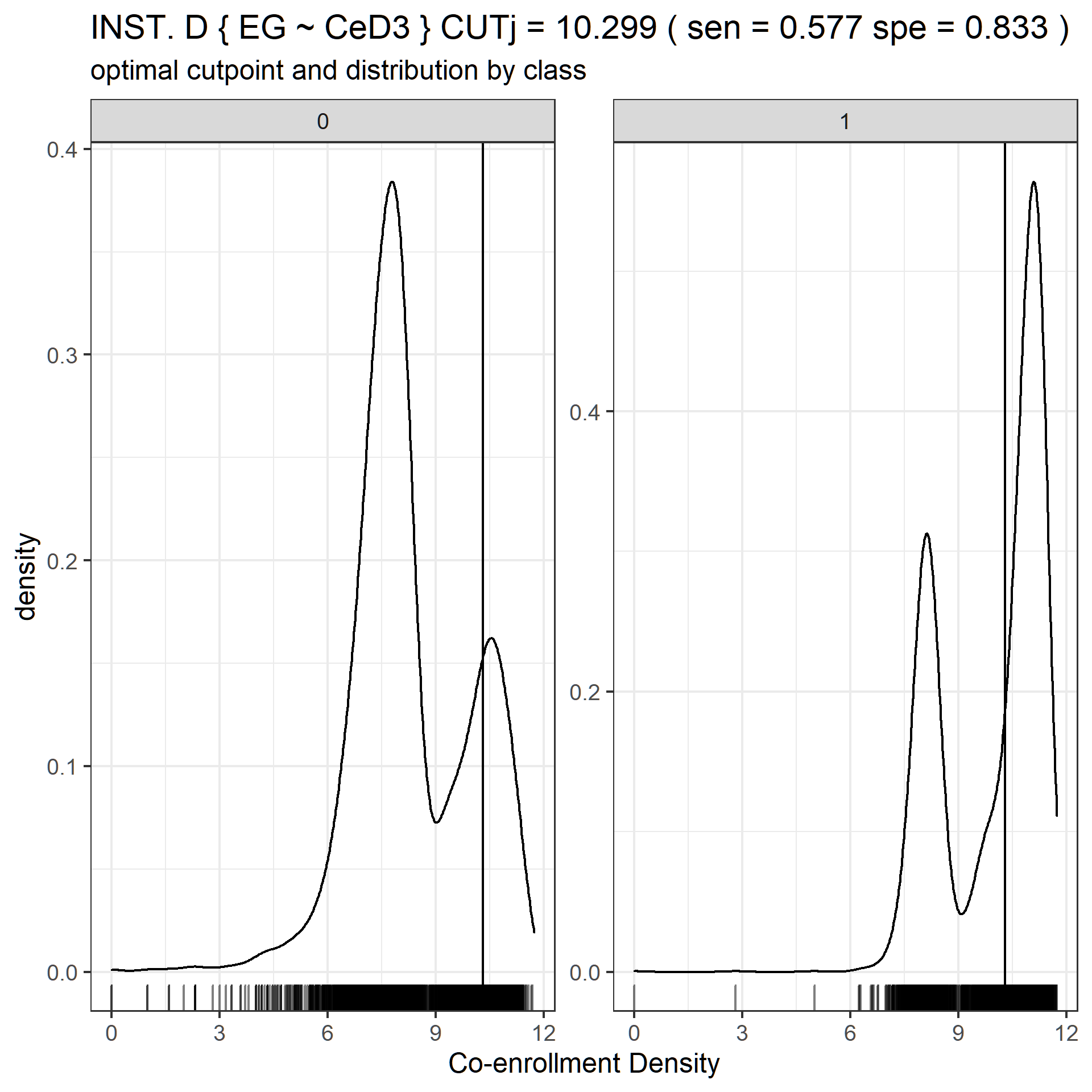

Supplement: Supplementary file 1 [file mmc1.zip › SupplementaryMaterials/338-ClassDen.png]

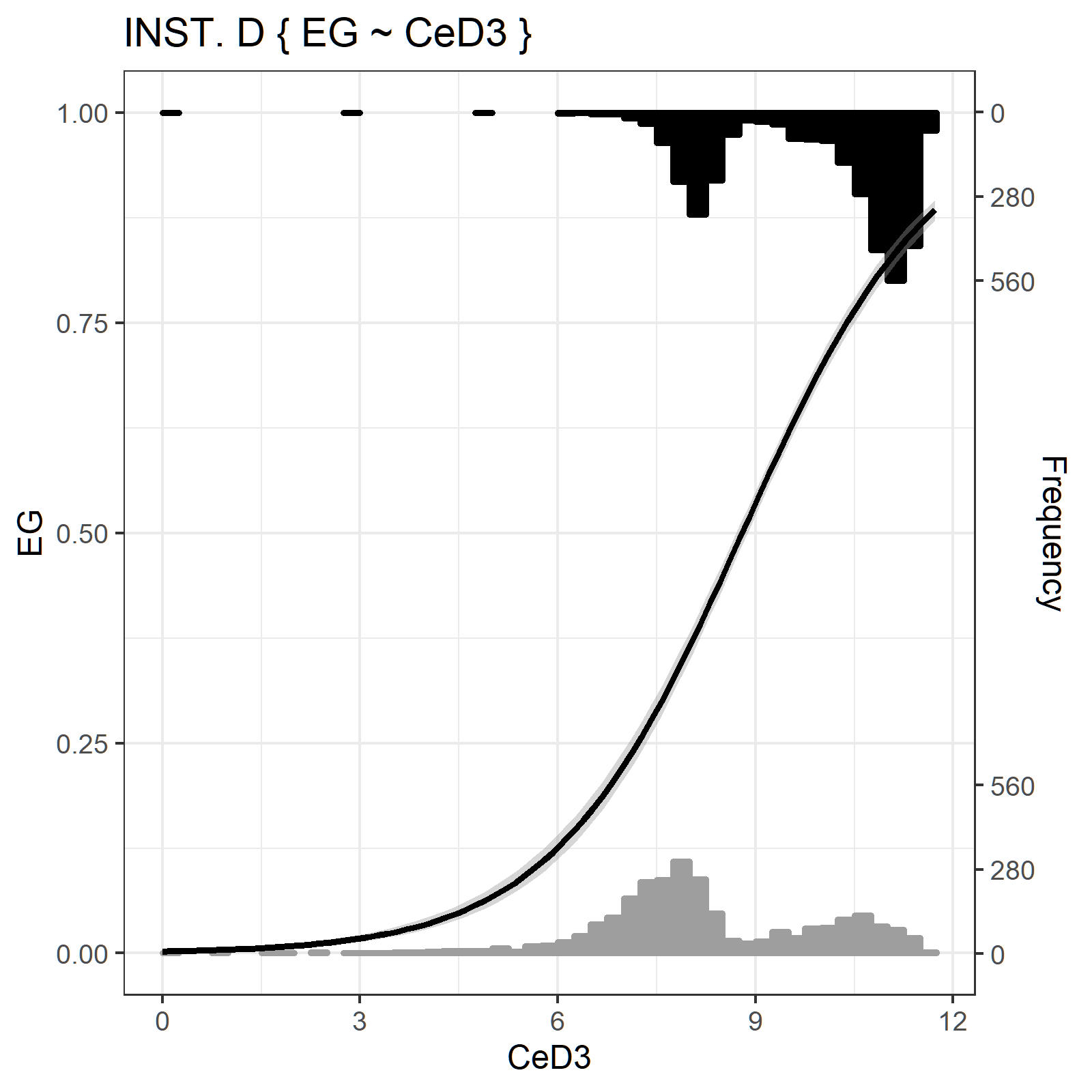

Supplement: Supplementary file 1 [file mmc1.zip › SupplementaryMaterials/338-LogitCurve.png]

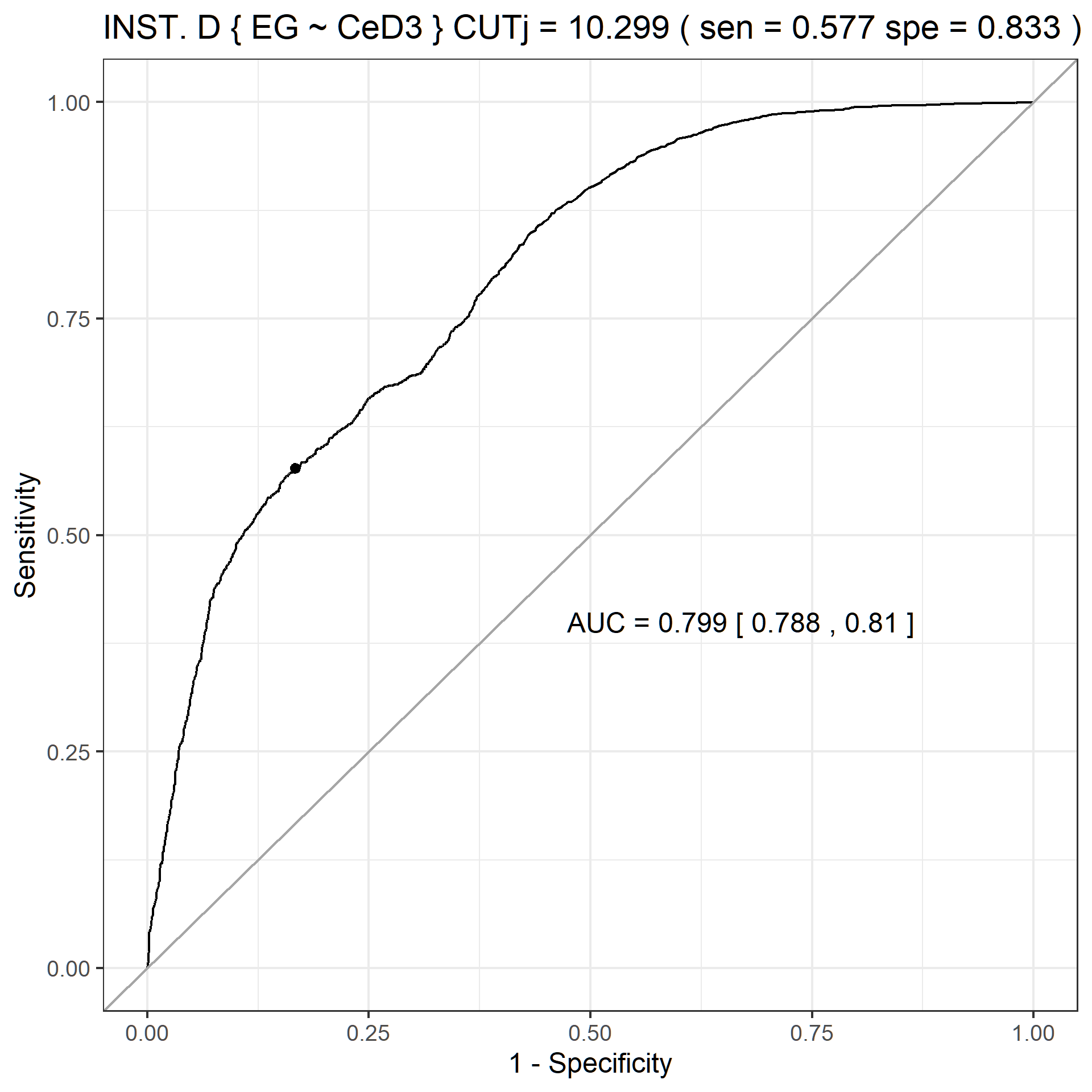

Supplement: Supplementary file 1 [file mmc1.zip › SupplementaryMaterials/338-ROCut.png]

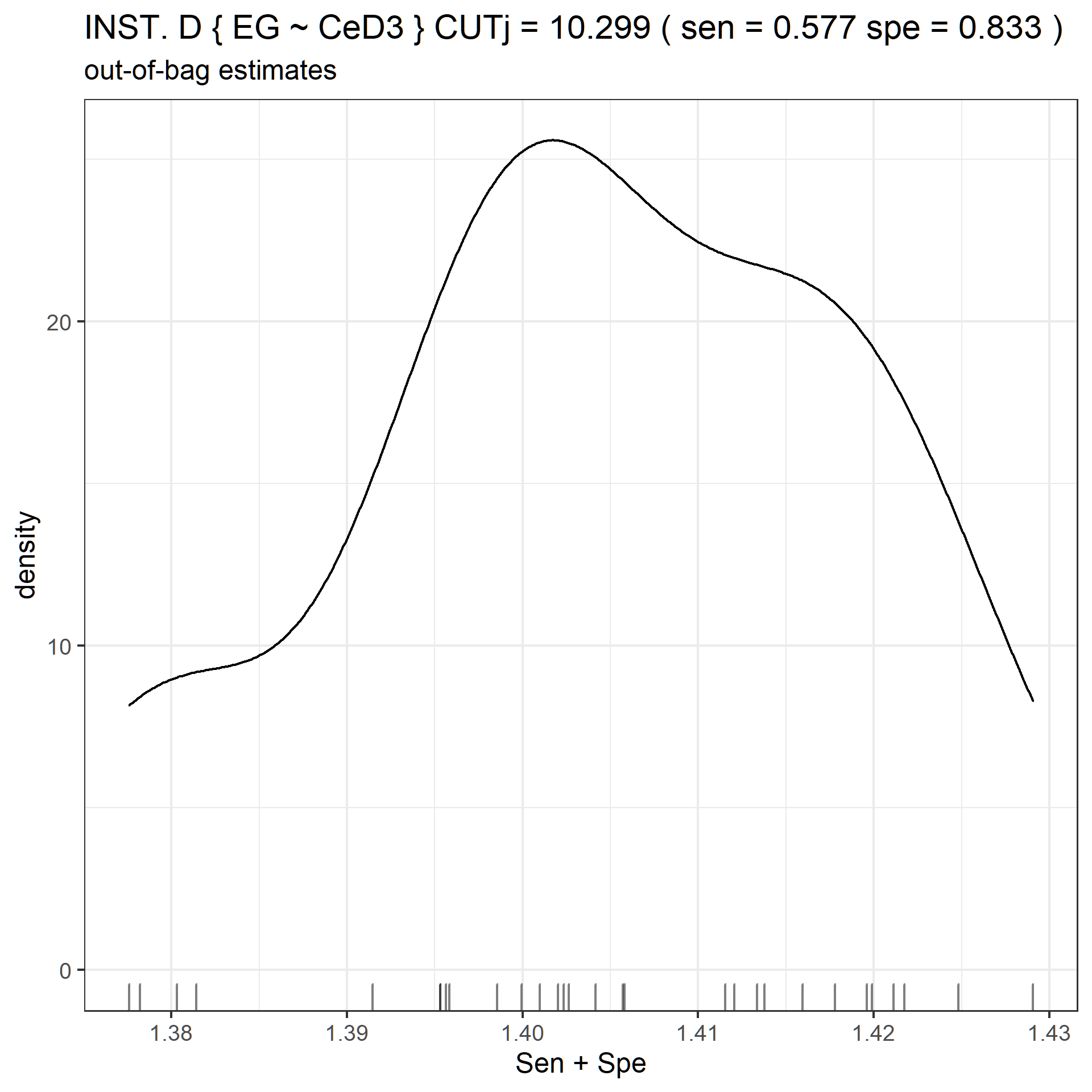

Supplement: Supplementary file 1 [file mmc1.zip › SupplementaryMaterials/338-SenSpe.png]

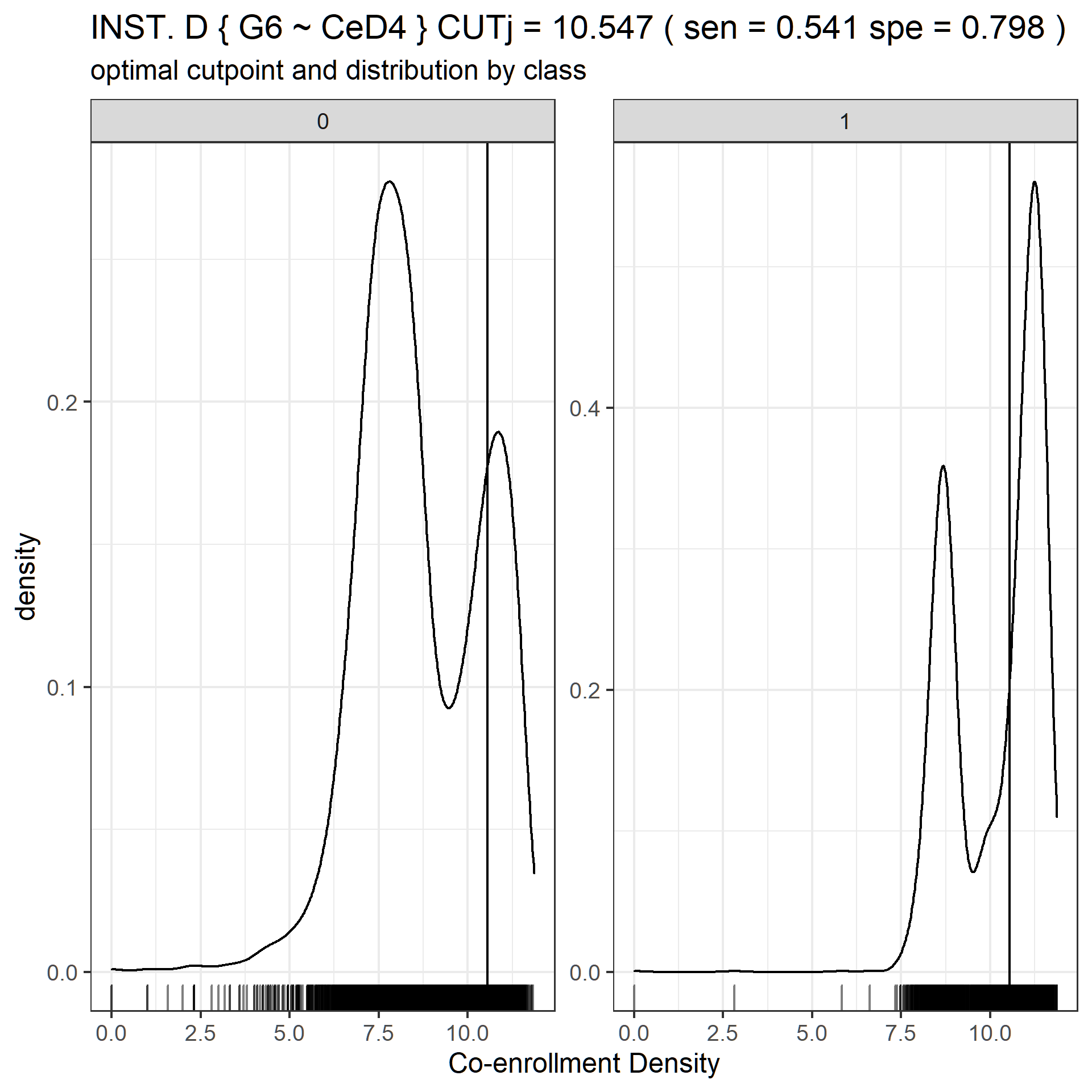

Supplement: Supplementary file 1 [file mmc1.zip › SupplementaryMaterials/347-ClassDen.png]

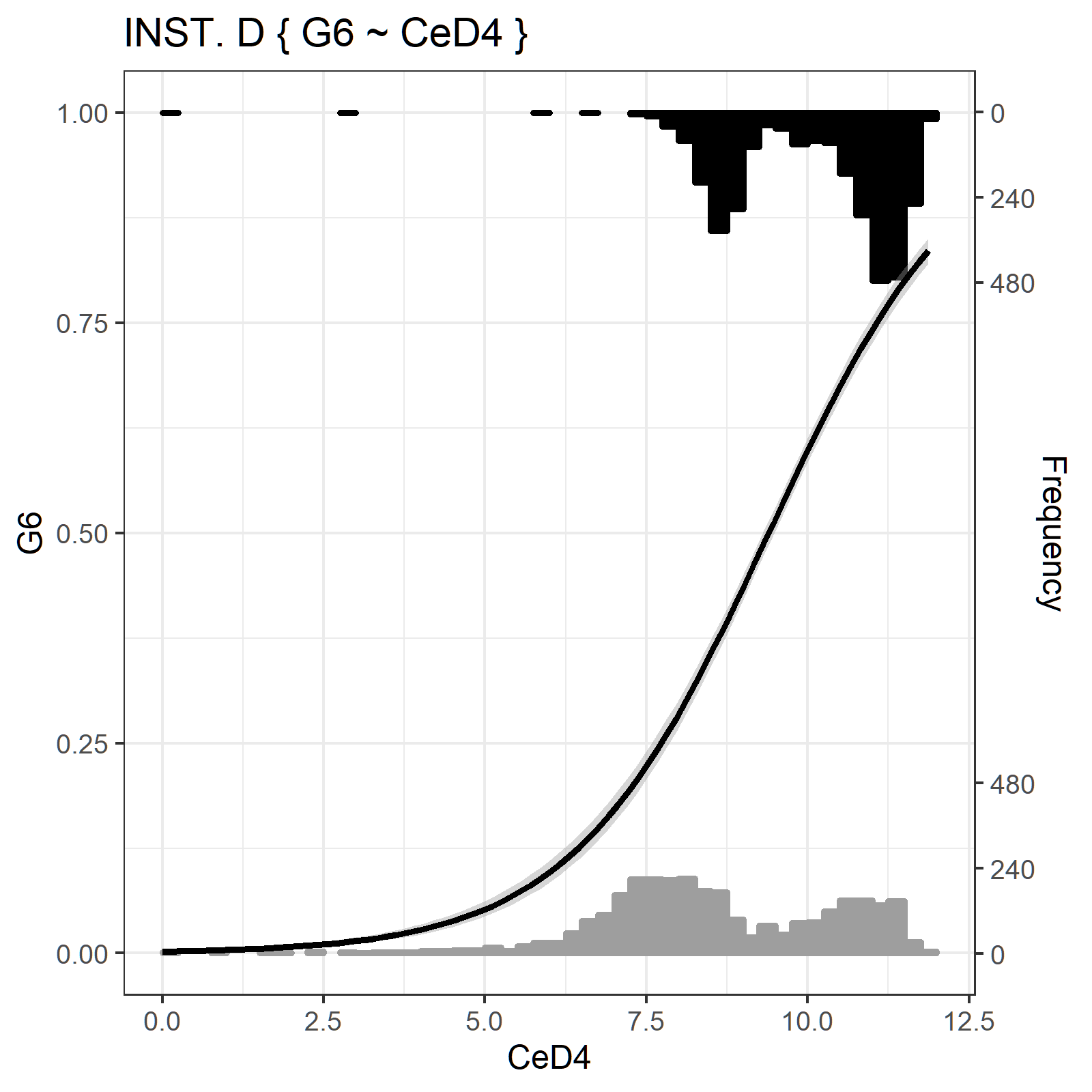

Supplement: Supplementary file 1 [file mmc1.zip › SupplementaryMaterials/347-LogitCurve.png]

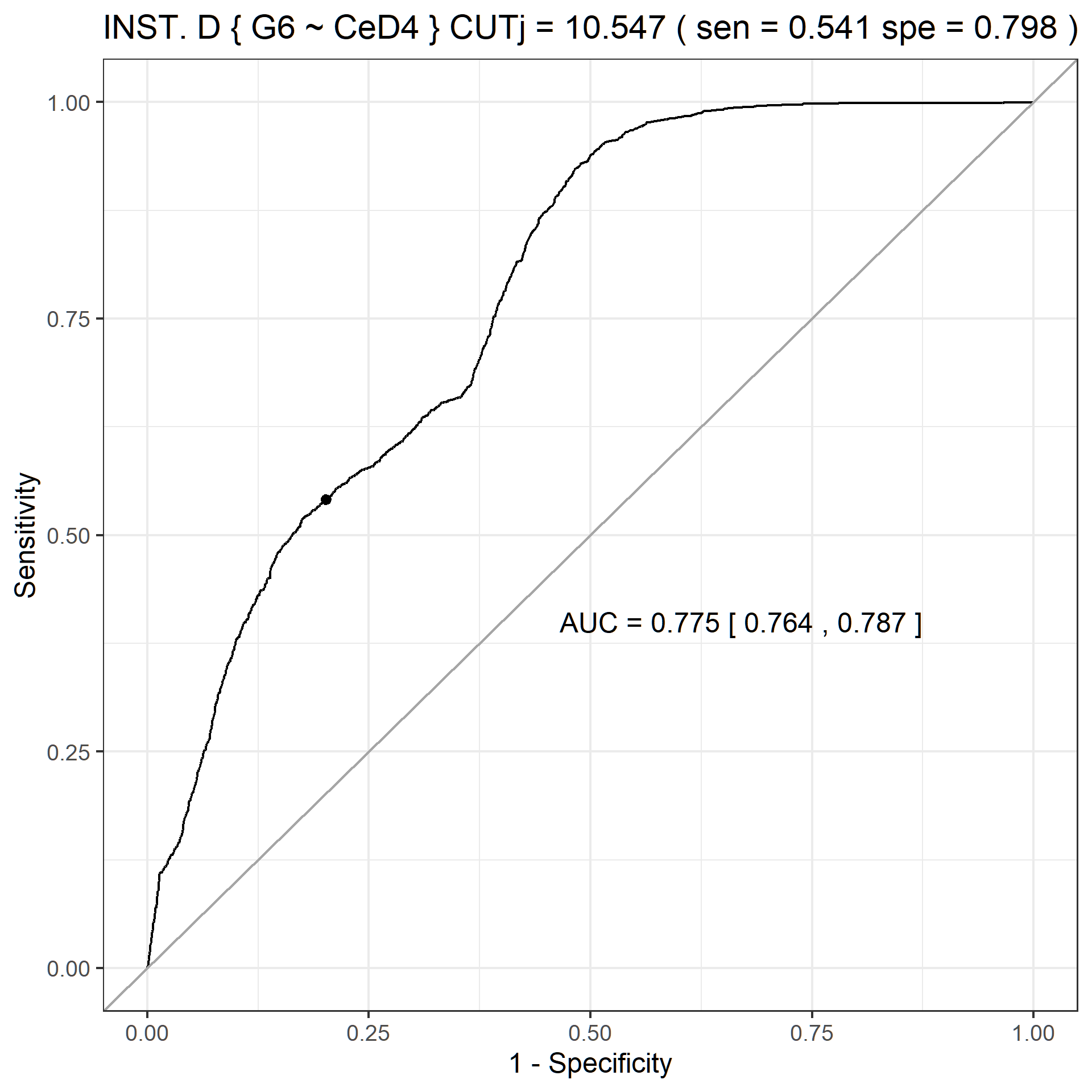

Supplement: Supplementary file 1 [file mmc1.zip › SupplementaryMaterials/347-ROCut.png]

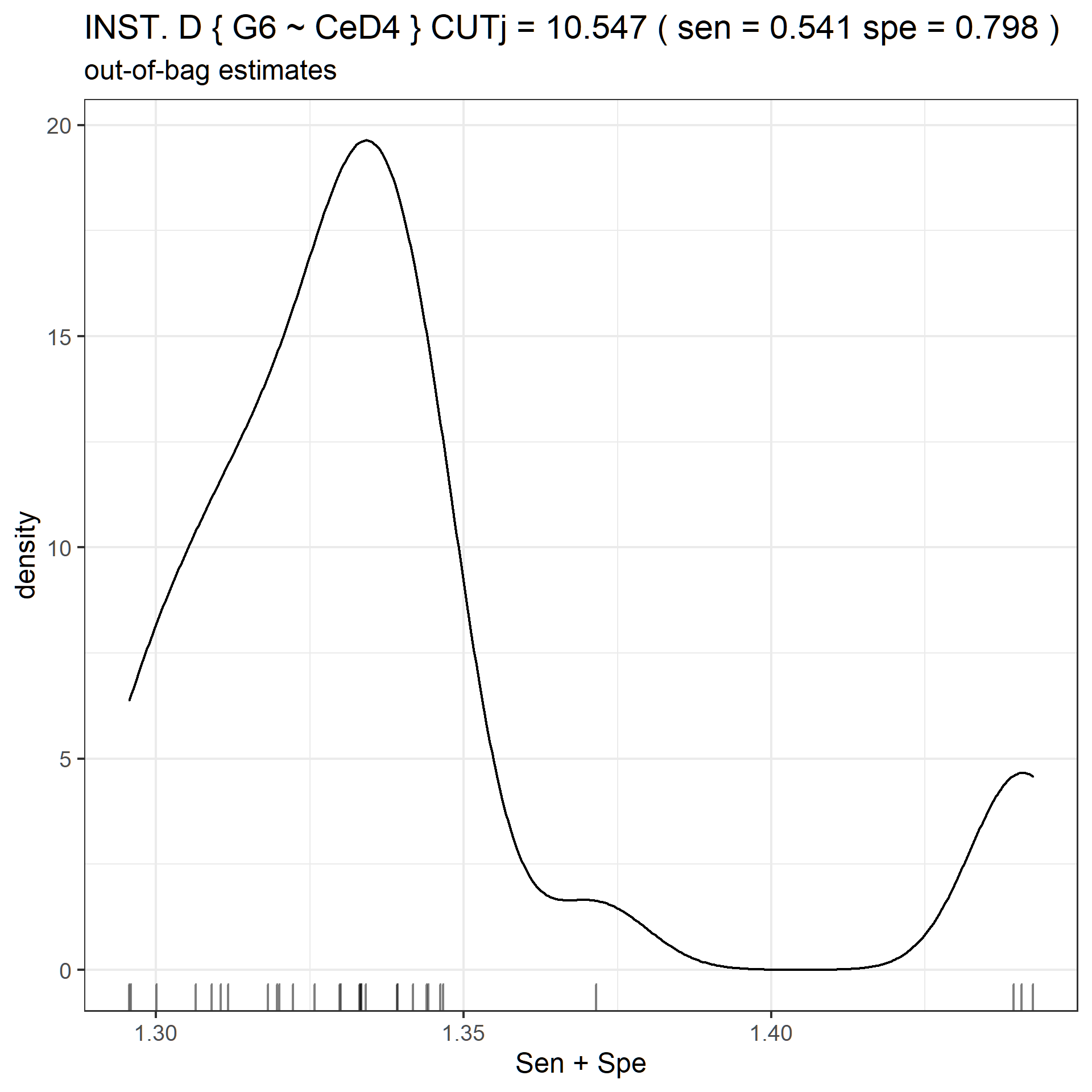

Supplement: Supplementary file 1 [file mmc1.zip › SupplementaryMaterials/347-SenSpe.png]

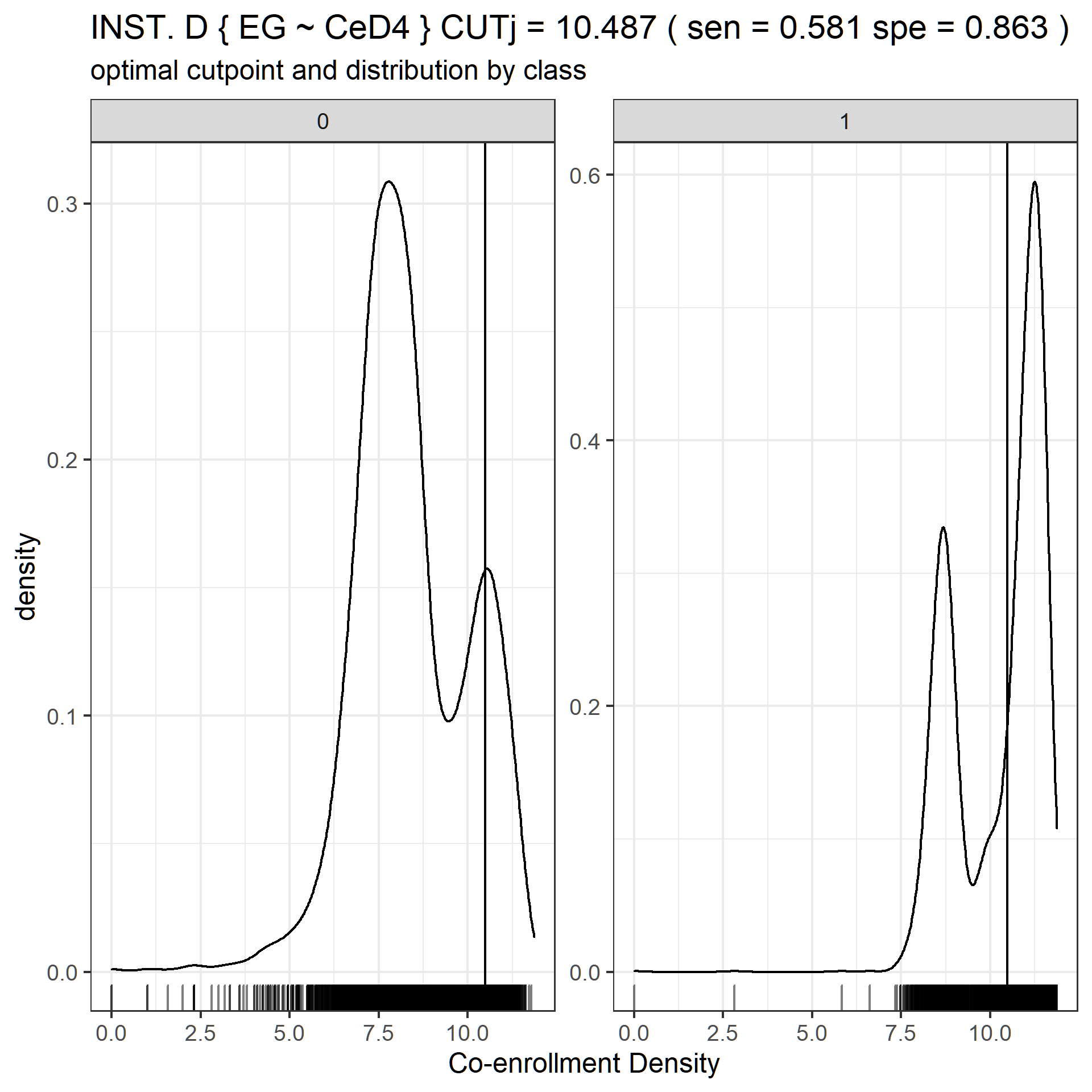

Supplement: Supplementary file 1 [file mmc1.zip › SupplementaryMaterials/348-ClassDen.png]

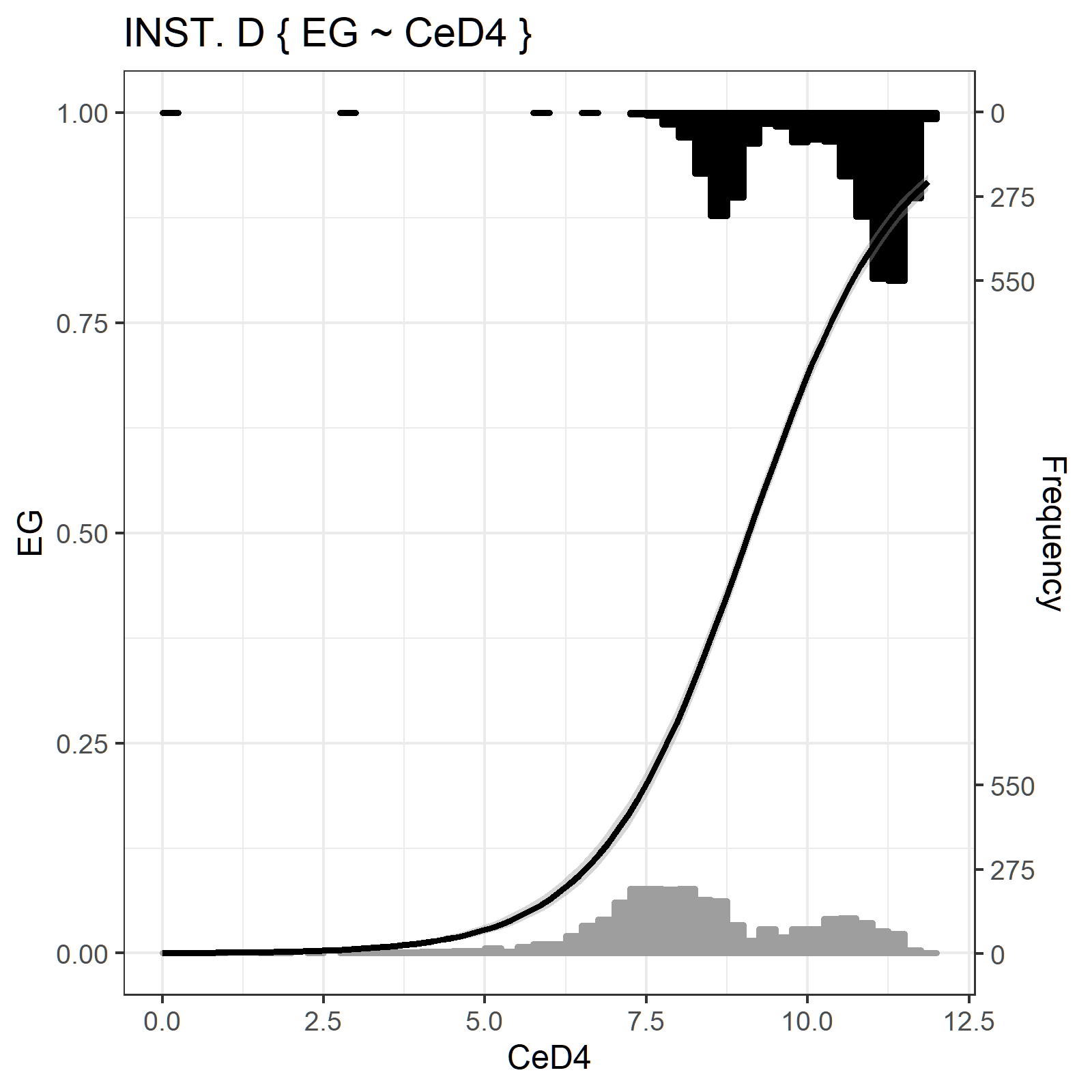

Supplement: Supplementary file 1 [file mmc1.zip › SupplementaryMaterials/348-LogitCurve.png]

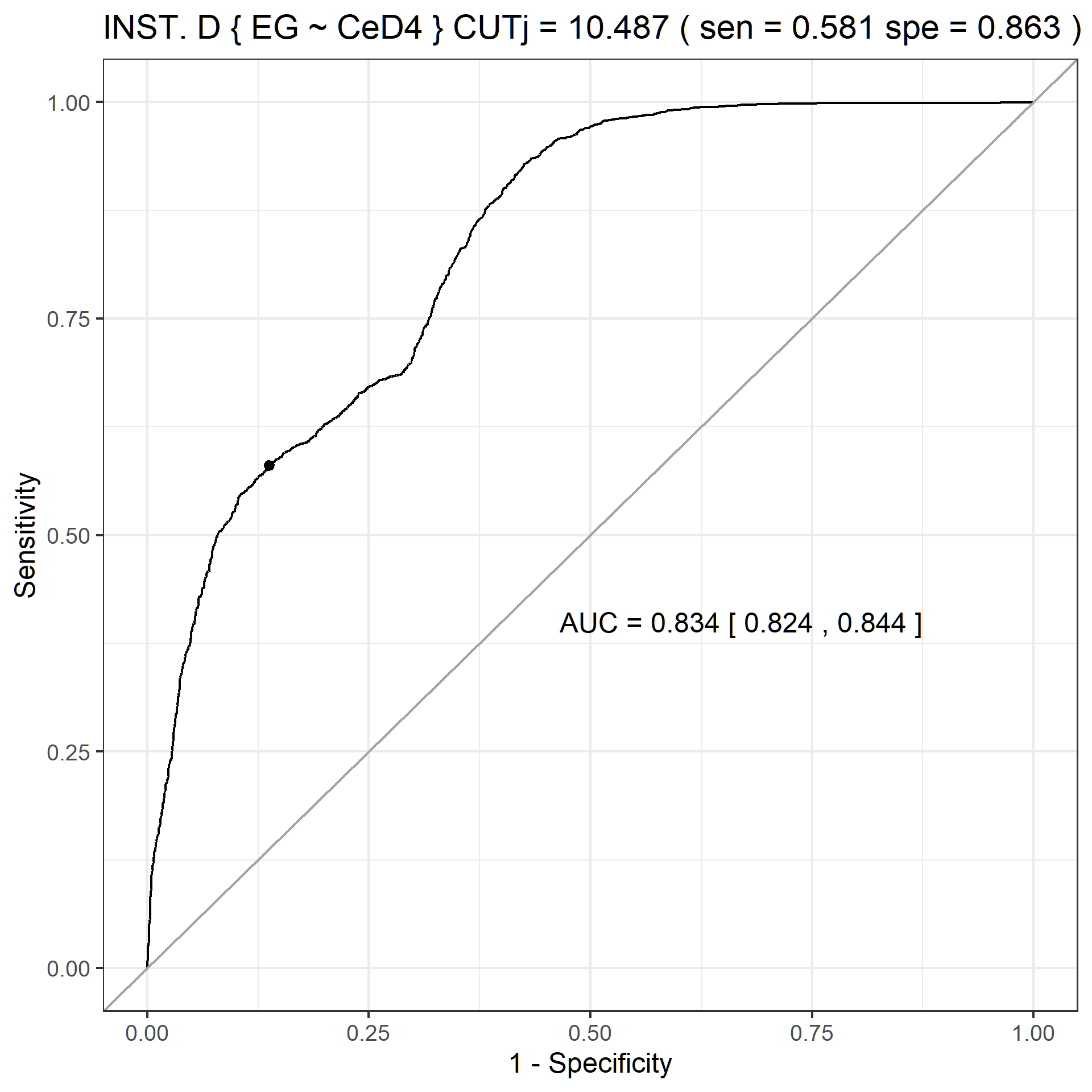

Supplement: Supplementary file 1 [file mmc1.zip › SupplementaryMaterials/348-ROCut.png]
